# Galactan synthesis in a single step via oligomerization of monosaccharides

**DOI:** 10.3762/bjoc.10.279

**Published:** 2014-11-13

**Authors:** Marius Dräger, Amit Basu

**Affiliations:** 1 Department of Chemistry, Box H, Brown University, Providence, RI 02912, USA

**Keywords:** arabinogalactan protein, glycosyl fluoride, glycosylation, oligosaccharides

## Abstract

Galactans ranging in length from one to five residues were prepared in a single step by treatment of the glycosyl donor 2,3,4-tri-O-benzoyl-β-D-galactopyranosyl fluoride with lanthanum perchlorate in the presence of an initiator alcohol. The product oligosaccharides were readily chromatographically separable. This oligomerization was used to synthesize a pentagalactan in a single step from monosaccharide building blocks in reasonable overall yields.

## Introduction

Despite numerous recent advances in the synthesis of complex oligosaccharides, unlike polypeptide or oligonucleotide assembly, their preparation remains far from a routine endeavor. The critical step in oligosaccharide assembly is the construction of the acetal or ketal glycosidic bond that links individual sugar residues together. The synthesis of an *n*-mer oligosaccharide generally requires at least *n−*1 separate glycosylation reactions, regardless of whether the molecule is assembled in a linear or convergent manner. Additionally, numerous other steps involving protecting group removal and installation are required to assure regio- and stereoselective glycan assembly. One-pot strategies for oligosaccharide synthesis can streamline the process by sequential iterative glycosylations, without the need for work-up and purification after the formation of each glycosidic bond, and have enabled the rapid preparation of complex glycans [1].

An alternative strategy for the efficient oligosaccharide formation involves the oligomerization of a single building block that functions both as a glycosyl donor and acceptor. This approach, which is essentially a polymerization of an AB-type monomer, has been used to prepare polysaccharides with varying molecular weight distributions. Glycosyl orthoesters [2–4], thiocyanates [5], anhydrosugars [6–8], and halides [9–10] have all successfully been oligomerized or polymerized. Glycosyl halides have also been polymerized to provide poly-orthoesters [11]. The products of these reactions are polydisperse and are isolated and treated as a mixture of oligosaccharides of varying length. While this polydispersity may not be a significant limitation in cases where the product polymers have interesting materials properties, it does not provide discrete compounds and is not useful for targeted oligosaccharide synthesis.

In contrast, there are several cases where the product mixture of an oligomerization reaction has been separated into discrete oligosaccharides of varying lengths. An early report describes the treatment of 2,3,4-tri-*O*-acetyl-glucopyranosyl bromide under Koenigs–Knorr conditions [12]. Products ranging from the β1→6 linked disaccharide to the hexasaccharide were obtained after separation, albeit in poor yields. A similar oligomerization of *N*-acetylglucosamine mediated by HF followed by chromatographic separation afforded chito-oligosaccharides as long as hexasaccharides in reasonable yields [13]. In most of these oligomerizations the reducing end of the oligomer is a hemi-acetal, or a reduced or protected derivative thereof, and both anomers are present [14]. An earlier attempt at the oligomerization of a 6-hydroxyglucosamine thioglycoside donor in the presence of an initiating primary alcohol resulted in a single glycosylation of the initiating alcohol to provide a glycoside product, and trace amounts of further oligomerization were detected in some cases [15]. Self-reaction of the donor, usually by cyclization, was a consistently significant side-reaction and even the source of the major product in several instances. To the best of our knowledge, there have not been any reports of monosaccharide oligmerization to provide stereochemically pure, discrete and separable oligosaccharide products that are functionalized as glycosides at their reducing termini [16]. We report here the oligomerization of a galactosyl donor in the presence of an initiating alcohol to prepare galactans ranging from mono- to pentasaccharides with concomitant control of stereochemistry and reducing end functionality.

## Results and Discussion

**Figure 1:**
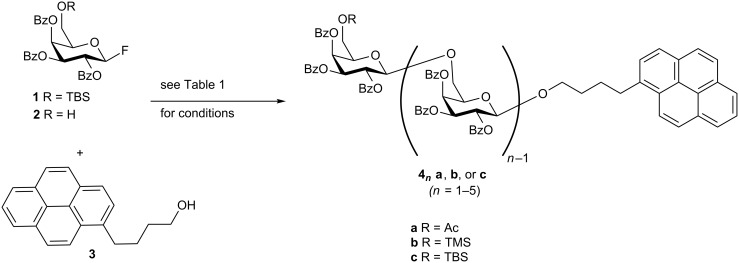
Pyrenebutanol (**3**) initiated oligomerization.

As part of a project on the synthesis of glycan fragments derived from arabinogalactan proteins (AGPs) [17–18], we sought to prepare a β1→6 linked galactan as a chemical probe. We elected to prepare the galactan with a pyrene-containing aglycone to aid detection in subsequent biochemical studies. We prepared the TBS-protected galactosyl fluoride **1** and carried out a glycosylation with 4-pyrenebutanol (**3**) as the acceptor (Figure 1), using conditions reported by Shibasaki (lanthanum perchlorate and potassium carbonate in acetonitrile) [19]. To our surprise, the reaction provided both the desired monosaccharide **4_1_a** as well as significant amounts of the disaccharide **4_2_a** and trisaccharide **4_3_a** (entry 1, Table 1) [20]. We surmised that the fluoride ion derived from the activated glycosyl donor was responsible for desilylating **1** during the course of the reaction. We observed no reaction when we used a TMS-protected version of **3** (entry 2, Table 1), even though silyl ethers have been reported as competent acceptors in this reaction [19]. The use of 2,6-di-*tert*-butylpyridine (DTBP) as the base resulted in a single glycosylation, and no oligomerization products were obtained (entry 3, Table 1). Moreover, loss of the silyl ether from the product monosaccharide was not observed. We believe that DTBP, which is soluble in acetonitrile, functioned as a more effective proton scavenger than the heterogeneous suspension of potassium carbonate. These observations suggest that desilylation is most likely the result of transient Brønsted acid formation during the reaction and not due to fluoride ion generation.

**Table 1:** Optimization of oligomerization reaction conditions.

Entry	Conditions	**4_1_** (*n* = 1) yield (%)	**4_2_** (*n* = 2) yield (%)	**4_3_** (*n* = 3) yield (%)	**4_4_** (*n* = 4) yield (%)	**4_5_** (*n* = 5) yield (%)	Overall yield^a^ (%)	Capping^b^

1	**1**, La(ClO_4_)_3_, K_2_CO_3_	50	21	12	–	–	83 (39)	a
2	**1**, La(ClO_4_)_3_, no base, TMS acceptor	–	–	–	–	–	N.R.^c^	a
3	**1**, La(ClO_4_)_3_, DTBP	99	–	–	–	–	99 (30)	–
4	**2**, La(ClO_4_)_3_, K_2_CO_3_	55	29	11	3	–	98 (50)	b_1_
5	**2**, La(ClO_4_)_3_, K_2_CO_3_ , syringe pump addition	50	29	9	4	–	92 (54)	b_2_
6	**2**, Yb(OTf)_3_, K_2_CO_3_ , syringe pump addition	40	19	4	–	–	63 (28)	b_1_
7	**2**, La(ClO_4_)_3_ + ZnCl_2_, syringe pump addition	58	29	10	3	–	100 (55)	b_1_
8	**2**, Hf(OTf)_4_, K_2_CO_3_	–	–	–	–	–	N.R	b_1_
9	**2**, La(ClO_4_)_3_ only, no base	39	33	18	4	–	94 (54)	b_1_
10	**2**, La(ClO_4_)_3_, K_2_CO_3_ , extra donor	40	35	19	5	–	99 (40)	b_1_
11	**2**, La(ClO_4_)_3_, K_2_CO_3_	46	32	14	5	2	99 (60)	c
12	**1**, La(ClO_4_)_3_, K_2_CO_3_	37	39	15	6	3	97 (60)	c

^a^The first number treats the glycosyl acceptor as the limiting reagent, and is the sum of the individual yields of the mono-, di-, trisaccharide etc. The value inside parentheses corresponds to the % yield of the donor summed across all the products. The second value is the yield of the reaction when the glycosyl donor is treated as the limiting reagent, and takes into account the fact that the reaction stoichiometry with respect to the donor varies for each oligosaccharide when summing up the total yield. The difference between the standard acceptor yield and the (donor yield) provides a sense of how efficiently/inefficiently the donor is incorporated into one of the products. Additional details of the donor yields for each of the oliogosaccharide products and a sample calculation are provided in Supporting Information File 1. ^b^Capping protocol for purification: a) Ac_2_O, pyridine; b_1_) TMSCl/HMDS; b_2_) TMSCN; c) TBSCl, imidazole. ^c^N.R. – no reaction;

In an attempt to tune silyl ether deprotection rates and promote formation of longer oligomers, we examined the glycosylation of a bulkier TBDPS-protected donor. While this donor underwent oligomerization, the products were a mixture of the TBDPS capped and desilylated oligosaccharides and purification and isolation of pure products was challenging, even after resilylation of the oligosaccharide mixture. However, to our surprise, we observed that the reaction using donor **2**, which lacks a silyl protecting group on the primary hydroxy group, proved to be remarkably clean, providing products ranging from the monosaccharide to the tetrasaccharide in isolable yield (Table 1, entry 4). In all of the reactions carried out thus far, we noticed that while the overall yields were very high with respect to the initial acceptor, the mass balance of the donor was low. The loss of donor could arise from linear and cyclic self-oligomerization competing with the desired oligomerization initiated with **3** [14–15,21]. We hypothesized that slow addition of the donor to a solution of **3** and La(ClO_4_)_3_ might reduce self-oligomerization. However, addition of **2** via a syringe pump did not significantly improve the yields, nor did it shift the product distribution to longer oligomers.

The use of La(ClO_4_)_3_ as the promoter appears to be critical in this reaction. The use of the more Lewis acidic [22] Yb(OTf)_3_ resulted in a reduction in both overall yield and in the degree of oligomerization (Table 1, entry 6). Shibasaki has previously reported improvements in both yield and reaction time using ZnCl_2_ as an additive [19,23], but its inclusion in the reaction did not result in any significant changes (Table 1, entry 7). Finally, the use of Hf(OTf)_4_ as a promoter [24] was completely ineffective and no glycosylation was observed (Table 1, entry 8). Carrying out the reaction in the absence of base did not have a significant effect, although it minimally shifted the product distribution towards longer oligomers (Table 1, entry 9). Somewhat surprisingly, the addition of another 0.5 equivalent of **2** after 120 minutes of reaction did not appear to significantly perturb either the overall yields or product distributions (Table 1, entry 10). We found that capping the product mixture as TBS ethers greatly improved product separation, resulting in higher overall yields for the process (Table 1, entry 11). Finally, we reexamined the use of the TBS protected glycosyl donor **1** under the optimized conditions of Table 1, entry 11, and confirmed that the presence/absence of a protecting group at the 6 position does not affect the product distribution (Table 1, entries 12 vs 11).

**Figure 2:**
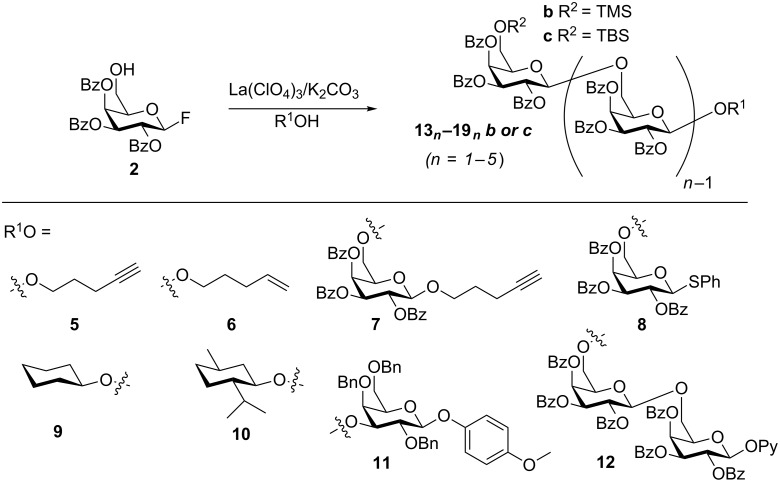
Scope of oligomerization.

With optimized conditions in hand, we explored the scope of the oligomerization reaction with other initiating acceptors (Figure 2). Reaction with 4-pentynol (**5**) (Table 2, entry 1) proceeded with a similar product distribution as **3**. Glycosylation with 4-pentenol (**6**) (Table 2, entry 2) also provided products up to the trisaccharide with good yield. Higher yields of trisaccharides could be obtained by using the monosaccharides **7** and **8** as the initial acceptors, which also provided isolable quantities of the tetrasaccharides (Table 2, entries 3–5). The successful use of **8** is notable, as the products can in turn be used as donors in subsequent glycosylation reactions upon activation of the thiophenyl group. When the glycosylation of **2** and **8** was carried out on a larger scale (>100 mg, Table 2, entry 5), followed by derivatization of the products as the TBS ethers, isolable quantities of the pentasaccharide **15_5_c** were generated.

**Table 2:** Oligomerizations with variations in initiating acceptors.

Entry	Acceptor	Product	(*n* = 1) yield (%)	(*n* = 2) yield (%)	(*n* = 3) yield (%)	(*n* = 4) yield (%)	(*n* = 5) yield (%)	Overall yield^a^ (%)	Capping^b^

1	**5**	**13**	53	24	9	6	–	92 (49)	b_2_
2	**6**	**14**	77	17	6	–	–	99 (50)	c
3	**7**	**13**	–	43	26	11	–	80 (40)	b_1_
4	**8**	**15**	–	50	21	8	–	79 (36)	b_2_
5	**8**	**15**	–	47	19	10	4	80 (44)	c
6	**9**	**16**	77	22	–	–	–	98 (49)	c
7	**10**	**17**	30	28	–	–	–	58 (27)	b_1_
8	**11**	**18**	33		–	–	–	33 (10)	b_1_
9	**12**	**4**	–	–	34	13	8	55 (27)	b_1_

^a^See caption under Table 1. ^b^Capping protocol for purification: b_1_) TMSCl/HMDS; b_2_) TMSCN; c) TBSCl, imidazole.

Secondary alcohols such as cyclohexanol or the sterically more hindered menthol successfully underwent glycosylation to provide mono- and disaccharides, but longer oligomers were not observed. Moreover, very little of the donor is incorporated into the products, suggesting that self-oligomerization competes effectively with the desired initiation. The use of *p*-methoxyphenyl 2,4,6-tri-*O*-benzyl-β-galactopyranoside (**11**) as the acceptor provided only the product derived from a single glycosylation in modest yield. When we used disaccharide **12** as the acceptor (Table 2, entry 12); a large amount of **12** was recovered (45%) and only a small portion of the donor **2** (27%) reacted with the remaining 55% of the acceptor.

## Conclusion

The results described here illustrate that oligogalactans as long as pentamers can be generated in a single reaction by the activation of a bifunctional galactosyl donor/acceptor in the presence of an initiating alcohol. While isolated yields of the tri-, tetra-, and pentasaccharides are modest, the obtained yields of 19%, 10%, and 4% (Table 2, entry 5) correspond to yields ranging from 57%, 63%, and 63%, respectively, per step of a linear iterative multistep sequence (i.e., glycosylation followed by deprotection) to assemble each of these products. As such, the overall oligomerization yields are competitive with many existing glycosylation methods, and the rapid access to glycans in a single step is a strength of this approach. All of the oligosaccharides reported here have successfully been separated using routine normal-phase silica gel columns on a commercially available flash chromatography system. The oligomerization described here is a highly efficient method for homo-oligosaccharide synthesis. We are currently investigating the extension of this approach to targets other than galactans and to the assembly of glycans linked via secondary hydroxy groups.

## Supporting Information

File 1 Experimental details for Tables 1 and 2, experimental protocols, and characterization data.

File 2 NMR spectra for all new compounds.
