# Supplementary material for: Galactan synthesis in a single step via oligomerization of monosaccharides
Source: Beilstein J Org Chem. 2014 Nov 13;10:2658–63. doi: 10.3762/bjoc.10.279 (PMC4273286; doi:10.3762/bjoc.10.279)
Supplement: File 1 — Experimental details for Tables 1 and 2, experimental protocols, and characterization data. [file Beilstein_J_Org_Chem-10-2658-s001.pdf]

Supporting Information File 1

for

Galactan synthesis in a single step via oligomerization of monosaccharides

Marius Dräger and Amit Basu*

Address: Department of Chemistry, Box H, Brown University, Providence, RI 02912, USA

Email: Amit Basu - abasu@brown.edu

*Corresponding author

Experimental details for Tables 1 and 2, experimental protocols, and characterization data

General Methods

Dry solvents were obtained from a commercially available solvent purification system based on protocols reported by Grubbs.[1] All reactions were carried out under nitrogen atmosphere unless otherwise stated. Compounds were purchased from standard suppliers and used without further . Thin layer chromatography was carried out on Merck silica gel 60 F254 precoated glass plates. Spots were detected by UV light and immersion in p-anisaldehyde stain. Column chromatography was performed on Teledyne ISCO CombiFlash® Rf purification system using pre-packed RediSep Rf Gold® Normal-Phase Silica Flash cartridges and gradient elution or with DAVISIL® Chromatographic Silica Media LC60A 35-70 micron. Evaporation of solvents was performed under reduced pressure at 30-40 °C. ^{1}H NMR spectra were recorded on a Bruker Avance III HD Ascend or Bruker Avance Ultra-Shield spectrometers at 600 MHz or 400 MHz, respectively. NMR spectra were referenced[2] to 4.79 ppm for D_2O and 7.26 ppm for $CHCl_3$ in 1H, COSY and HSQC and 77.16 ppm for ^{13}C and HSQC, ^{19}F NMR spectra were referenced to external trichlorofluoromethane at 0 ppm. Coupling constants (J) are given in [] and chemical shifts in [ppm]. Electrospray ionization (ESI) and fast atom bombardment (FAB) mass spectra were obtained using JEOL JMS-600H double focusing magnetic sector mass spectrometer for HRMS and Thermo LCQ Deca XP Max ion trap mass spectrometer with Shimadzu HPLC system for LRMS in positive mode.

Scheme S1

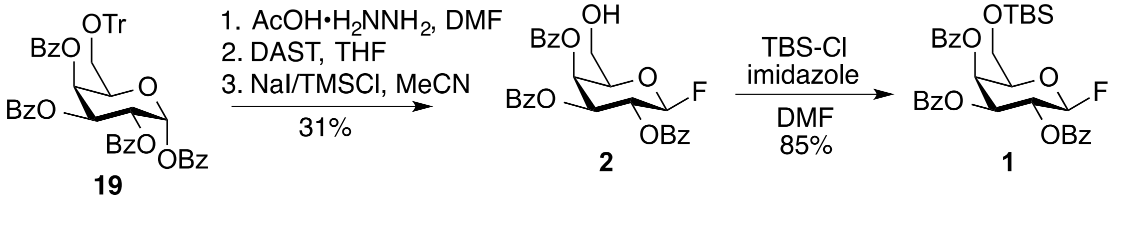

*Synthesis of glycosyl donors **1** and **2***

6-O-Triphenylmethyl-1,2,3,4-tetra-O-benzoyl-D-galactopyranose[3] (**19**) (250 mg, 0.30 mmol) and hydrazine acetate (56 mg, 0.61 mmol)) were dissolved in DMF (5 mL) and the solution was stirred overnight.[4] The solution was diluted with ethyl acetate and washed with water.

The aqueous phase was extracted with ethyl acetate 3 times and the combined organic layers were washed with water, brine and dried with magnesium sulfate. The organic solvent was removed under reduced pressure providing 6-O-triphenylmethyl-2,3,4-tri-O-benzoyl-D-galactopyranose as an off-white sticky solid which was used in the next step without further characterization.

6-O-Triphenylmethyl-2,3,4-tri-O-benzoyl-D-galactopyranose (150 mg, 0.20 mmol) was dissolved in anhydrous THF (2 mL) and cooled to -30 °C, followed by the addition of DAST (30 µL, 0.23 mmol).[5] The solution was slowly warmed but never allowed to exceed 0 °C and was stirred for 1 hour or until TLC showed completion. The reaction was quenched with methanol and taken up in dichloromethane and water. After phase separation the aqueous phase was extracted 3 times with dichloromethane and the combined organic layers were washed with water and brine, and dried using magnesium sulfate. The organic solvent was removed under reduced pressure providing 6-O-triphenylmethyl-2,3,4-tri-O-benzoyl-D-galactopyranosyl fluoride as a white solid in quantitative yield as a 1:1 mixture of anomers (^{19}F NMR (282 MHz, $CDCl_3$) δ -140.13 (dd, J = 52.5, 12.1), -150.05 (dd, J = 53.3, 24.0)).

2,3,4-Tri-O-benzoyl-β-D-galactopyranosyl fluoride (2)**.** 6-O-triphenylmethyl-2,3,4-tri-O-benzoyl-D-galactopyranosyl fluoride (2.00 g, 2.72 mmol) and sodium iodide (1.20 g, 8.00 mmol) were dissolved in acetonitrile (25 mL), cooled to 0°C, and treated with TMSCl (1.1 mL, 8.67 mmol).[6] The reaction was stirred for 30 minutes, followed by the addition of ice cold water (13 mL). Following an additional 30 minutes of stirring, a 10% (w/v) sodium thiosulfate solution was added, and the solution was stirred until the brown color disappeared. The solution was extracted with dichloromethane (3 times) and the combined organic layers were washed with water, brine and dried using magnesium sulfate. The solvent was removed under reduced pressure. Purification using automated flash column chromatography provided **2** as a white solid in 31 % yield (410 mg, 0.83 mmol). ^{1}H NMR (400 MHz, $CDCl_3$) δ 8.16 – 8.11 (m, 2H), 8.05 – 8.01 (m, 2H), 7.88 – 7.83 (m, 2H), 7.70 – 7.64 (m, 1H), 7.60 – 7.40 (m, 6H), 7.33 – 7.27 (m, 2H), 5.96 (ddd, J = 12.2, 10.3, 7.0 Hz, 1H), 5.92 – 5.89 (m, 1H), 5.68 – 5.63 (m, 1H), 5.64 (dd, J = 52.4, 7.1 Hz, 1H), 4.25 – 4.16 (m, 1H), 3.95 (dd, J = 11.9, 6.7 Hz, 1H), 3.75 (dd, J = 11.9, 6.5 Hz, 1H); ^{13}C NMR (101 MHz, $CDCl_3$) δ 166.5, 165.5, 165.3, 134.0, 133.63, 133.60, 133.56, 130.1, 130.0, 129.9, 129.8, 128.81, 128.78, 128.52, 128.46, 128.4, 107.4 (d, $J_{F,C}$ = 218.8 Hz), 74.5 (d, $J_{F,C}$ = 3.8 Hz), 70.9 (d, $J_{F,C}$ = 10.4 Hz), 70.0 (d, $J_{F,C}$ = 24.6 Hz), 68.1, 60.5; ^{19}F NMR (282 MHz, $CDCl_3$) δ -140.42 (dd, J = 52.2, 12.1 Hz); ESI-HRMS m/z calculated for $[M+H]^+$ $C_{27}H_{24}FO_8$ 495.1455; found 495.1469

6-O-tert-butyldimethylsilyl-2,3,4-Tri-O-benzoyl-β-D-galactopyranosyl fluoride (1)**.** Galactopyranosyl fluoride **2** (200 mg, 0.40 mmol) and imidazole (44 mg, 0.65 mmol) were dissolved in DMF (2 mL). TBSCl (180 mg, 1.20 mmol) was added and the solution was stirred overnight.[7] The solution was taken up in ethyl acetate and water. After phase separation the aqueous phase was extracted with ethyl acetate (3 times). The combined organic layers were washed with water, brine and dried using magnesium sulfate. The organic solvent was removed under reduced pressure. Purification using automated flash column chromatography provided **1** as a white solid in 85 % yield (207 mg, 0.34 mmol). ^{1}H NMR (400 MHz, $CDCl_3$) δ 8.12 – 8.08 (m, 2H), 8.03 – 7.98 (m, 2H), 7.85 – 7.80 (m, 2H), 7.68 – 7.63 (m, 1H), 7.58 – 7.39 (m, 6H), 7.32 – 7.30 (m, 1H), 7.28 – 7.26 (m, 1H), 6.02 – 5.99 (m, 1H), 5.85 (ddd, J = 12.1, 10.3, 7.0 Hz, 1H), 5.68 – 5.51 (m, 2H), 4.21 – 4.12 (m, 1H), 3.93 (dd, J = 9.9, 5.8 Hz, 1H), 3.86 (dd, J = 10.0, 7.8 Hz, 1H), 0.85 (s, 9H), 0.01 (s, 3H), -0.04 (s, 3H); ^{13}C NMR (101 MHz, $CDCl_3$) δ 166.0, 165.7, 165.6, 134.0, 133.8, 130.4, 130.3,

129.7, 129.4, 129.2, 129.1, 129.0, 128.9, 128.8, 108.0 (d, $J_{F,C}$ = 218.2 Hz), 74.9 (d, $J_{F,C}$ = 3.4 Hz), 71.5 (d, $J_{F,C}$ = 10.4 Hz), 70.6 (d, $J_{F,C}$ = 24.9 Hz) , 67.5, 61.1, 26.2, 18.6, -5.2, -5.3; ESI-HRMS m/z calculated for $[M+H]^+$ $C_{33}H_{38}FO_8Si$ 609.2320; found 609.2303

Scheme S2

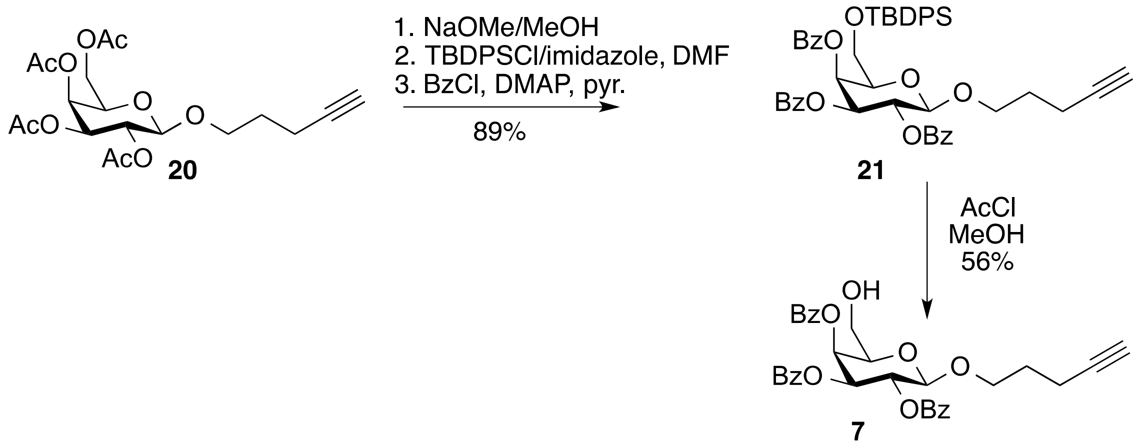

Synthesis of 7
4-pentynyl-2,3,4,6-tetra-O-acetyl-β-D-galactopyranoside[8] (**20**) (650 mg, 1.45 mmol) was suspended in methanol (5 mL) followed by addition of 1M sodium methoxide solution in methanol (65 µL, 0.07 mmol). The reaction was stirred until TLC showed completion (2 h) (dichloromethane / methanol 10:1 v/v) the solution was neutralized with washed Amberlite $120H^+$ ion-exchange resin. After filtration, the solvent was removed under reduced pressure and further concentration under high vacuum provided pentynyl-β-galactopyranoside[8] as a colorless amorphous solid in 98% yield (350 mg, 1.42 mmol) which was used directly in the next reaction without further characterization.
4-pentynyl-β-D-galactopyranoside (350 mg, 1.42 mmol) and imidazole (126 mg, 1.85 mmol) were dissolved in anhydrous DMF (5 mL) and the reaction mixture cooled to 0 °C. TBDPSCl (0.45 mL, 2.10 mmol) was slowly added and the reaction mixture was allowed to warm to room temperature.[9] After 16 h the reaction mixture was concentrated in vacuo and the resulting residue dissolved in dichloromethane (10 mL). The organic layer was washed with water (10 mL) and the aqueous layer extracted with dichloromethane (2 times, 10 mL). The combined organic layers were washed with brine (10 mL), dried with magnesium sulfate, filtered and concentrated under reduced pressure. The crude 4-pentynyl-6-O-tert-butyldiphenylsilyl-β-D-galactopyranoside was dried in high vacuum for 2 h and used in the next reaction without further purification.
4-pentynyl-6-O-tert-butyldiphenylsilyl-β-D-galactopyranoside (862 mg, 1.78 mmol) and DMAP (5 mg, 0.04 mmol) were dissolved in anhydrous pyridine (10 mL) and the solution was cooled to 0 °C. Benzoyl chloride (1.35 mL, 11.61 mmol) was added, the solution was allowed to warm to room temperature, and stirred overnight. After the addition of methanol to decompose excess benzoyl chloride, the solution was concentrated in vacuo. The residue was taken up in ethyl acetate (12 mL), washed with saturated ammonium chloride solution (12 mL) water (twice, 12 mL) and brine (12 mL). After drying with magnesium sulfate the solvent was removed under reduced pressure and the crude residue was dried under high vacuum. Purification using automated flash column chromatography provided 4-pentynyl-2,3,4-tri-O-benzoyl-6-O-tert-butyldiphenylsilyl-β-D-galactopyranoside **21** as an off white solid in 91 % yield (1.29 g, 1.62 mmol). ^1H NMR (400 MHz, $CDCl_3$) δ 8.05 – 8.01 (m, 2H), 7.98 – 7.93 (m, 2H), 7.83 – 7.78 (m, 2H), 7.68 – 7.64 (m, 2H), 7.61 (m, 1H), 7.53 – 7.34 (m,

10H), 7.30 – 7.22 (m, 4H), 7.15 – 7.09 (m, 2H), 6.03 (dd, J = 3.4, 1.2 Hz, 1H), 5.69 (dd, J = 10.4, 7.7 Hz, 1H), 5.61 (dd, J = 10.4, 3.4 Hz, 1H), 4.75 (d, J = 7.8 Hz, 1H), 4.06 (ddd, J = 7.6, 6.4, 1.3 Hz, 1H), 3.98 (dt, J = 9.8, 5.6 Hz, 1H), 3.89 – 3.79 (m, 2H), 3.64 (m, J = 9.8, 8.0, 5.2 Hz, 1H), 2.12 (ddd, J = 7.4, 6.5, 2.6 Hz, 2H), 1.79 (t, J = 2.6 Hz, 1H), 1.78 – 1.61 (m, 2H), 1.00 (s, 9H); ^{13}C NMR (101 MHz, $CDCl_3$) δ 165.7, 165.5, 165.3, 135.61, 135.59, 135.5, 133.3, 133.1, 133.0, 132.6, 130.03, 129.98, 129.9, 129.8, 129.73, 129.69, 129.5, 129.0, 128.5, 128.40, 128.36, 128.30, 128.25, 127.82, 127.80, 127.7, 127.63, 127.58, 101.8, 83.7, 83.4, 77.4, 73.9, 72.0, 70.1, 68.6, 68.4, 67.9, 61.4, 28.3, 26.7, 19.0, 14.8.

4-Pentynyl-2,3,4-tri-O-benzoyl-β-D-galactopyranoside (7). A solution of acetyl chloride (84 µL 1.18 mmol) in methanol (2.1 mL) was cooled in a water bath at 22 °C.[10] To this solution was added dropwise a solution of **21** (50 mg, 0.06 mmol) dissolved in diethylether (2.1 mL). The mixture was allowed to stir overnight at room temperature. The reaction was neutralized with solid sodium bicarbonate, filtered over Celite, and the solvent removed under reduced pressure. The crude product was purified using automated column chromatography providing the desired product **7** as a white solid in 56% yield (20 mg, 0.035 mmol). ^{1}H NMR (400 MHz, $CDCl_3$) δ δ 8.17 – 8.13 (m, 2H), 8.04 – 8.00 (m, 2H), 7.88 – 7.83 (m, 2H), 7.69 – 7.63 (m, 1H), 7.59 – 7.40 (m, 6H), 7.34 – 7.24 (m, 2H), 5.91 – 5.83 (m, 2H), 5.62 (dd, J = 10.4, 3.4 Hz, 1H), 4.85 (d, J = 8.0 Hz, 1H), 4.12 – 4.04 (m, 2H), 3.88 (dd, J = 12.1, 6.6 Hz, 1H), 3.81 – 3.73 (m, 1H), 3.70 (dd, J = 11.9, 6.7 Hz, 1H), 2.67 (s, 1H), 2.29 – 2.14 (m, 2H), 1.88 (t, J = 2.6 Hz, 1H), 1.87 – 1.70 (m, 2H); ^{13}C NMR (101 MHz, $CDCl_3$) δ 166.9, 165.6, 165.4, 133.9, 133.4, 133.3, 130.24, 130.16, 130.1, 129.79, 129.75, 129.4, 128.81, 128.76, 128.73, 128.68, 128.6, 128.5, 128.43, 128.37, 101.9, 83.5, 74.0, 71.9, 70.0, 69.0, 68.8, 68.6, 60.7, 28.3, 14.8; ESI-HRMS m/z calculated for $[M+H]^+$ $C_{32}H_{31}O_9$ 559.1968; found 559.1991

Scheme S3

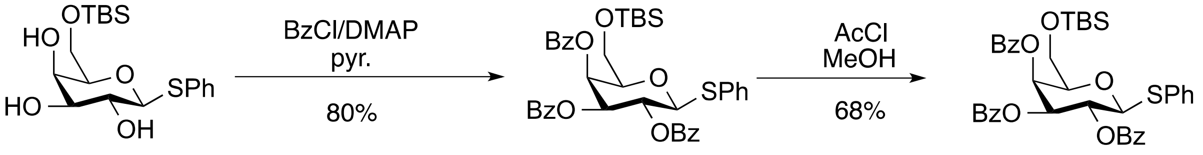

*Synthesis of **8***

Phenyl-2,3,4-tri-O-benzoyl-6-O-tert-butyldimethylsilyl-1-thio-β-D-galactopyranoside (***23***). Phenyl-6-O-tert-butyldimethylsilyl-1-thio-β-D-galactopyranoside[11] (5.03 g, 13.00 mmol) and DMAP (25 mg, 0.21 mmol) were dissolved in pyridine (60 mL) and cooled to 0 °C. Benzoyl chloride (8.9 mL, 76.54 mmol) was added and the solution was allowed to warm to room temperature and stirred overnight. After the addition of methanol to decompose excess benzoyl chloride, the solution was concentrated in vacuo and the residue was taken up in ethyl acetate (120 mL) and washed with saturated ammonium chloride solution (60 mL) followed by washing with water (2x, 120 mL) and brine (120 mL). After drying with magnesium sulfate, the solvent was removed under reduced pressure. The crude residue was dried in high vacuum. Purification using automated flash column chromatography provided **23** as an off-white solid in 80 % yield (6.71 g, 9.60 mmol). ^{1}H NMR (400 MHz, $CDCl_3$) δ 8.03 – 7.98 (m, 2H), 7.94 – 7.89 (m, 2H), 7.81 – 7.76 (m, 2H), 7.66 – 7.61 (m, 3H), 7.59 – 7.53 (m, 1H), 7.51 – 7.45 (m, 2H), 7.45 – 7.38 (m, 6H), 7.26 (m, 2H), 6.06 – 5.91 (m, 1H), 5.74 (t, J = 9.9 Hz, 1H), 5.61 (dd, J = 9.9, 3.2 Hz, 1H), 5.04 (d, J = 9.9 Hz, 1H), 4.11 (ddd, J = 7.3, 5.8, 1.1 Hz, 1H), 3.91 (dd, J = 10.0, 6.0 Hz, 1H), 3.77 (dd, J = 10.1, 7.3 Hz, 1H), 0.88 (s, 9H), 0.04 (s, 3H), -0.01 (s, 3H).

Phenyl-2,3,4-tri-O-benzoyl-1-thio-β-D-galactopyranoside (8). A solution of acetyl chloride (0.2 mL, 2.80 mmol) in methanol (5 mL) was cooled in a water bath at 22 °C. To this solution a solution of **23** (100 mg 0.14 mmol) dissolved in diethylether (5 mL) was added dropwise. The mixture was allowed to stir for 4 h at room temperature and afterwards neutralized with triethylamine and the solvent removed under reduced pressure. The crude product was purified using automated column chromatography providing **8**[12] as a white solid in 68% yield (56.8 mg, 0.097 mmol). ^{1}H NMR (600 MHz, $CDCl_3$) δ 8.04 – 7.99 (m, 2H), 7.94 – 7.91 (m, 2H), 7.82 – 7.79 (m, 2H), 7.67 – 7.63 (m, 1H), 7.61 – 7.55 (m, 3H), 7.51 – 7.46 (m, 2H), 7.46 – 7.40 (m, 4H), 7.40 – 7.35 (m, 2H), 7.28 – 7.24 (m, 2H), 5.86 (dd, *J* = 3.3, 0.9 Hz, 1H), 5.82 (t, *J* = 9.9 Hz, 1H), 5.61 (dd, *J* = 9.9, 3.3 Hz, 1H), 5.05 (d, *J* = 9.9 Hz, 1H), 4.13 (td, *J* = 6.7, 1.0 Hz, 1H), 3.89 (dt, *J* = 12.4, 6.5 Hz, 1H), 3.65 (dt, *J* = 12.7, 6.5 Hz, 1H), 2.62 (t, *J* = 7.1 Hz, 1H); ^{13}C NMR (151 MHz, $CDCl_3$) δ 166.6, 165.5, 165.2, 134.1, 133.8, 133.41, 133.36, 130.9, 130.1, 129.84, 129.78, 129.3, 128.70, 128.65, 128.61, 128.58, 128.5, 128.3, 85.6, 77.9, 77.3, 77.0, 76.8, 73.1, 69.0, 68.0, 60.8; ESI-HRMS m/z calculated for $[M+H]^+$ $C_{33}H_{29}O_8S$ 585.1583; found 585.1570

Scheme S4

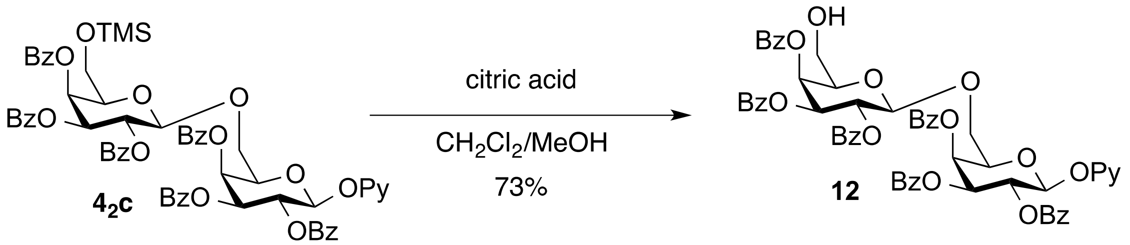

4-(Pyren-1-yl)butyl 2,3,4-tri-O-benzoyl-β-D-galactopyranosyl-(1→6)-2,3,4-tri-O-benzoyl-β-D-galactopyranoside (12). The TMS-protected disaccharide (**4$_2$b)** (43 mg, 0.03 mmol) was dissolved in a mixture of methanol and dichloromethane (2:1 v/v) (2 mL) followed by the addition of citric acid until pH paper indicated a pH-value of approximately 2.[13] The solution was stirred until TLC indicated completion of the reaction. To the solution dichloromethane (10 mL) was added and the organic phase was washed with saturated sodium bicarbonate, water and brine, followed by drying of the organic phase with sodium sulfate and removal of the solvent under reduced pressure. Purification using automated flash column chromatography (hexanes / ethyl acetate gradient) provided **12** as a slightly yellow solid in 73% yield (30 mg, 0.02 mmol). ^{1}H NMR (400 MHz, $CDCl_3$) δ 8.21 – 8.11 (m, 5H), 8.10 – 8.00 (m, 7H), 7.96 (m, 1H), 7.82 – 7.77 (m, 5H), 7.71 – 7.67 (m, 1H), 7.66 – 7.60 (m, 2H), 7.54 – 7.40 (m, 9H), 7.40 – 7.34 (m, 1H), 7.28 – 7.19 (m, 6H), 7.07 (m, 2H), 5.95 (d, *J* = 3.5 Hz, 1H), 5.82 (dd, *J* = 10.5, 7.8 Hz, 1H), 5.78 – 5.71 (m, 2H), 5.57 – 5.50 (m, 2H), 4.79 (d, *J* = 7.9 Hz, 1H), 4.67 (d, *J* = 7.9 Hz, 1H), 4.17 – 4.08 (m, 2H), 3.90 – 3.83 (m, 2H), 3.80 (dt, *J* = 10.5, 5.4 Hz, 1H), 3.65 (dd, *J* = 12.0, 6.6 Hz, 1H), 3.50 – 3.43 (m, 1H), 3.42 – 3.33 (m, 1H), 3.22 – 3.13 (m, 2H), 1.87 – 1.72 (m, 1H), 1.73 – 1.58 (m, 2H), 1.60 – 1.49 (m, 1H); ^{13}C NMR (101 MHz, $CDCl_3$) δ 166.7, 165.63, 165.56, 165.5, 165.3, 136.6, 133.8, 133.6, 133.3, 133.24, 133.20, 132.9, 131.4, 130.9, 130.2, 130.1, 129.9, 129.78, 129.75, 129.7, 129.5, 129.3, 129.2, 129.1, 128.9, 128.8, 128.73, 128.68, 128.6, 128.5, 128.43, 128.38, 128.33, 128.30, 128.25, 128.1, 127.5, 127.13, 127.09, 126.6, 126.3, 125.8, 125.0, 124.8, 124.74, 124.70, 123.4, 101.6, 101.4, 74.0, 73.0, 71.8, 71.7, 70.02, 70.00, 69.88, 68.87, 68.7, 68.2, 60.6, 33.0, 29.4, 28.1.

General procedure for glycosylations

For x mmol of the glycosyl fluoride, 1.30 equiv of the lanthanide catalyst, and 60 mg per mL of solvent of 5 Å molecular sieves were combined in a schlenk tube and heated under vacuum at 180 °C for 2 hours. In a separate schlenk flask the fluoride and 0.33 equiv of the glycosyl acceptor was combined, azeotroped three times using benzene and then left under vacuum for 2 hours.

After cooling the lanthanide mixture to 0 °C, the glycosyl donor / acceptor mixture was dissolved in acetonitrile (0.075M) and transferred to the schlenk tube containing the lanthanide, to which the identical amount of acetonitrile was added previously (diluting the solution to 0.037M). The reaction was left to stir for 4 hours and checked via TLC for progress (hexanes / ethyl acetate 1:1 v/v).

The reaction was quenched using saturated bicarbonate solution and stirred for 5 minutes, followed by the addition of ethyl acetate and water. The phases were separated and the aqueous phase was extracted three times using ethyl acetate. The combined organic phases were washed with brine and dried with sodium sulfate. The solvent was evaporated under reduced pressure and the crude product was treated according to the end-capping procedures shown below. After end-capping, the crude protected product was taken up in dichloromethane and loaded onto an appropriately sized ISCO cartridge packed with Celite, on which the solvent was again evaporated to prepare the crude product dry loaded on Celite. The crude product was then purified using automated column chromatography (hexanes / ethylacetate gradient).

Acetyl end capping:

The crude reaction mixture was taken up in pyridine (0.1 M) and acetic anhydride was added (114 equiv. based on donor) and the reaction was stirred overnight and the solvents were removed under reduced pressure.

TMS-Trimix end capping (pyridine, HMDS, TMSCl):[14]

The crude reaction mixture was taken up in pyridine (0.2 M) under nitrogen atmosphere, HMDS (13 equiv) and TMSCl (10.5 equiv) were added, the reaction was stirred overnight and solvents were removed under reduced pressure.

TMS-TMSCN end capping:[15]

The crude reaction mixture was taken up in acetonitrile (0.2 M) and TMSCN (11 equiv) was added. The reaction was stirred overnight and the solvents were removed under reduced pressure.

TBS end capping:[16]

The crude reaction mixture was taken up in DMF (0.2 M) followed by the addition of TBSCl (3 equiv) and imidazole (4 equiv). The reaction was stirred overnight and diluted with ethyl acetate (10 mL) followed by washing of the organic phase with water (twice) and brine (once). After separation, the organic phase was dried using sodium sulfate and the solvent was removed under reduced pressure.

Capping protocol for purification:

a) Ac_2O, pyridine; b_1) TMSCl/HMDS; b_2) TMSCN; c) TBSCl, imidazole.

Experimental details for entries in Tables 1 and 2

Table S1 – Reaction details for entries in Table 1

Entry	Acceptor	Donor	Solvent	Base	Promoter
1	**3** (8 mg, 0.029 mmol)	**1** (59 mg, 0.0967 mmol)	MeCN (2 mL) / DCM (0.5 mL)	15 mg K_2CO_3	62 mg $La(ClO_4)_3$ (0.114 mmol)
2	**3-TMS** (10 mg, 0.029 mmol)	**1** (59 mg, 0.0967 mmol)	MeCN (2 mL) / DCM (0.5 mL)	No base	19 mg $La(ClO_4)_3$ (0.034 mmol)
3	**3** (8 mg, 0.029 mmol)	**1** (59 mg, 0.0967 mmol)	MeCN (2 mL) / DCM (0.5 mL)	46 mg DTBP	62 mg $La(ClO_4)_3$ (0.114 mmol)
4	**3** (33 mg, 0.12 mmol)	**2** (182 mg, 0.367 mmol)	MeCN (10 mL)	60 mg K_2CO_3	248 mg $La(ClO_4)_3$ (0.454 mmol)
5	**3** (36 mg, 0.132 mmol)	**2** (181 mg, 0.366 mmol)	MeCN (10 mL)	60 mg K_2CO_3	248 mg $La(ClO_4)_3$ (0.454 mmol)
6	**3** (32 mg, 0.118 mmol)	**2** (179 mg, 0.362 mmol)	MeCN (10 mL)	60 mg K_2CO_3	282 mg $Yb(OTf)_3$ (0.454 mmol)
7	**3** (18 mg, 0.066 mmol)	**2** (94 mg, 0.190 mmol)	MeCN (5 mL)	30 mg K_2CO_3	124 mg $La(ClO_4)_3$ (0.227 mmol) + 15 mg $ZnCl_2$ (0.112 mmol)
8	**3** (16 mg, 0.058 mmol)	**2** (92 mg, 0.186 mmol)	MeCN (5 mL)	30 mg K_2CO_3	176 mg $Hf(OTf)_4$ (0.227 mmol)
9	**3** (16 mg, 0.058 mmol)	**2** (94 mg, 0.190 mmol)	MeCN (5 mL)	No base	124 mg $La(ClO_4)_3$ (0.227 mmol)
10	**3** (17 mg, 0.060 mmol)	**2** (92 mg, 0.186 mmol)	MeCN (5 mL)	30 mg K_2CO_3	124 mg $La(ClO_4)_3$ (0.227 mmol)
11	**3** (17 mg, 0.062 mmol)	**2** (94 mg, 0.191 mmol)	MeCN (5 mL)	30 mg K_2CO_3	124 mg $La(ClO_4)_3$ (0.227 mmol)
12	**3** (17 mg, 0.062 mmol)	**1** (117 mg, 0.192 mmol)	MeCN (5 mL)	30 mg K_2CO_3	124 mg $La(ClO_4)_3$ (0.227 mmol)

Table S2 – Product amounts for entries in Table 1

**Entry**	$\mathbf{4}_1$ (n=1) yield %, (mmol, mg)	$\mathbf{4}_2$ (n=2) %, (mmol, mg)	$\mathbf{4}_3$ (n=3) %, (mmol, mg)	$\mathbf{4}_4$ (n=4) %, (mmol, mg)	$\mathbf{4}_5$ (n=5) %, (mmol, mg)	**Overall yield**	**Capping**
1	50 [15] (0.015, 12)	21 [13] (0.006, 8)	12 [11] (0.004, 6)	-	-	83 [39]	a
2	-	-	-	-	-	N.R	a
3	99 [30] (0.029, 25)	-	-	-	-	99 [30]	-
4	55 [18] (0.066, 54)	29 [18] (0.033, 43)	11 [10] (0.013, 22)	3 [4] (0.0045, 11)	-	98 [50]	b_1
5	50 [18] (0.066, 54)	29 [20] (0.038, 49)	9 [10] (0.012, 21)	4 [6] (0.005, 12)	-	92 [54]	b_2
6	40 [13] (0.047, 39)	19 [12] (0.022, 29)	4 [3] (0.005, 8)	-	-	63 [28]	b_1
7	58 [20] (0.038, 32)	29 [20] (0.022, 29)	10 [11] (0.005, 8)	3 [4] (0.002, 2)	-	100 [55]	b_1
8	-	-	-	-	-	N.R	b_1
9	39 [12] (0.023, 19)	33 [20] (0.019, 25)	18 [17] (0.011, 19)	4 [5] (0.002, 5)	-	94 [54]	b_1
10	40 [9] (0.024, 20)	35 [15] (0.021, 27)	19 [12] (0.011, 20)	5 [4] (0.003, 7)	-	99 [40]	b_1
11	46 [15] (0.029, 25)	32 [21] (0.020, 27)	14 [14] (0.009, 16)	5 [7] (0.003, 7)	2 [3] (0.001, 3)	99 [60]	c
12	37 [12] (0.023, 20)	39 [25] (0.024, 32)	15 [15] (0.010, 17)	6 [8] (0.004, 9)	3 [4] (0.002, 4)	97 [60]	c

Capping protocol for purification: a) Ac_2O, pyridine; b_1) TMSCl/HMDS; b_2) TMSCN; c) TBSCl, imidazole.

Yields in [ ] correspond to yields based on the donor as the limiting reagent, calculated for each product of the glycosylation. A sample calculation is found after Table S4.

Table S3 – Reaction details for entries in Table 2

Entry	Acceptor	Donor	Solvent	Base	Promoter
1	**5** (5 μL, 0.059 mmol)	**2** (90 mg, 0.182 mmol)	MeCN (5 mL)	30 mg K_2CO_3	124 mg $La(ClO_4)_3$ (0.227 mmol)
2	**6** (7 μL, 0.066 mmol)	**2** (94 mg, 0.189 mmol)	MeCN (5 mL)	30 mg K_2CO_3	124 mg $La(ClO_4)_3$ (0.227 mmol)
3	**7** (32 mg, 0.058 mmol)	**2** (92 mg, 0.186 mmol)	MeCN (5 mL)	30 mg K_2CO_3	124 mg $La(ClO_4)_3$ (0.227 mmol)
4	**8** (34 mg, 0.058 mmol)	**2** (92 mg, 0.186 mmol)	MeCN (5 mL)	30 mg K_2CO_3	124 mg $La(ClO_4)_3$ (0.227 mmol)
5	**8** (144 mg, 0.247 mmol)	**2** (368 mg, 0.744 mmol)	MeCN (20 mL)	120 mg K_2CO_3	496 mg $La(ClO_4)_3$ (0.908 mmol)
6	**9** (7 μL, 0.063 mmol)	**2** (93 mg, 0.187 mmol)	MeCN (5 mL)	30 mg K_2CO_3	124 mg $La(ClO_4)_3$ (0.227 mmol)
7	**10** (9 mg, 0.057 mmol)	**2** (94 mg, 0.191 mmol)	MeCN (5 mL)	30 mg K_2CO_3	124 mg $La(ClO_4)_3$ (0.227 mmol)
8	**11** (65 mg, 0.116 mmol)	**2** (184 mg, 0.372 mmol)	MeCN (10 mL)	60 mg K_2CO_3	248 mg $La(ClO_4)_3$ (0.454 mmol)
9	**12** (33 mg, 0.025 mmol)	**2** (46 mg, 0.093 mmol)	MeCN (5 mL)	15 mg K_2CO_3	62 mg $La(ClO_4)_3$ (0.114 mmol)

Table S4 – Product amounts for entries in Table 3

Entry	Acceptor	Product	Monosacch. ($n = 1$) yield % (mmol, mg)	Disacch ($n = 2$) yield % (mmol, mg)	Trisacch ($n = 3$) yield % (mmol, mg)	Tetrasacch ($n = 4$) yield % (mmol, mg)	Pentasacch ($n = 5$) yield % (mmol, mg)	Overall yield (%)	Capping
1	**5**	**13**	53 [17] (0.031, 20)	24 [15] (0.014, 15)	9 [9] (0.005, 8)	6 [8] (0.004, 8)	-	92 [49]	b_2
2	**6**	**14**	77 [27] (0.051 (34)	17 [12] (0.012, 13)	6 [6] (0.004, 6)	-	-	99 [50]	c
3	**7**	**13**	-	43 [13] (0.025, 28)	26 [17] (0.015, 24)	11 [10] (0.007, 13)	-	80 [40]	b_1
4	**8**	**15**	-	50 [16] (0.029, 33)	21 [13] (0.012, 19)	8 [7] (0.004, 9)*	-	79 [36]	b_2
5	**8**	**15**	-	47 [16] (0.116, 137)	19 [13] (0.047, 78)	10 [10] (0.025, 53)	4 [5] (0.009, 22)	80 [44]	c
6	**9**	**16**	77 [26] (0.049, 33)	22 [15] (0.014, 16)	-	-	-	98 [49]	c
7	**10**	**17**	30 [9] (0.017, 12)	28 [18] (0.017, 19)	-	-	-	58 [27]	b_1
8	**11**	**18**	33 [10] (0.038, 42)	-	-	-	-	33/10	b_2
9	**12**	**4**	-	-	34 [11] (0.008, 15)	13 [9] (0.003, 7)	8 [7] (0.002 5)**	55 [27]	b_1

Capping protocol for purification: a) Ac_2O, pyridine; b_1) TMSCl/HMDS; b_2) TMSCN; c) TBSCl, imidazole; *) corrected for DCM contamination
**) corrected for Hexanes contamination

Yields in [ ] correspond to yields based on the donor as the limiting reagent, calculated for each product of the glycosylation. A sample calculation is found below.

Comment of calculation of yields

The acceptor based yields are calculated normally – i.e. % yield = mmol of product/mmol acceptor (0.029).

Since the donor gets incorporated into products longer than **4_1a** multiple times, the calculations for the yields factor in the number of equivalents of donor in the product.. For example, the product **4_2a** contains one equivalent of pyrene butanol and two equivalents of the glycosyl donor.

Sample yield calculation for Entry 1 Table 1

The reaction was run using 0.029 mmol of acceptor and 0.0967 mmol of donor.

The reaction yielded 7.9 mg of **4_2a**. Dividing 7.9 mg by the molecular weight of **4_2a** (1265.31 g/mol) gives a value of 0.006 mmols. This value is multiplied by two, since there are two equivalents of donor incorporated into **4_2a**, providing 0.012 mmols of donor in the product.

Dividing 0.012 by the initial amount of donor (0.0967 mmols) generates a percent yield of 13% when the donor is considered the limiting reagent.
Similarly, for **4_3a**, we obtained 6.1 mg or 0.004 mmols. Since **4_3a** contains three equivalents of the donor. 0.004 mmols was multiplied by three, providing 0.012 mmols of donor in the product. Division of 0.012 mmols by 0.0967 mmols provides a 11% yield

Characterization data for all oligomerization products

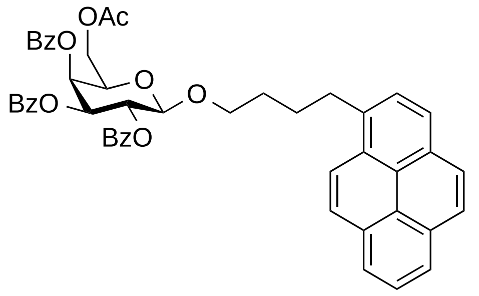

4-(Pyren-1-yl)butyl 6-O-acetyl-2,3,4-tri-O-benzoyl-β-D-galactopyranoside **4_1a**. ^{1}H NMR (600 MHz, $CDCl_3$) δ 8.20 – 8.15 (m, 3H), 8.11 – 8.06 (m, 3H), 8.05 – 7.99 (m, 4H), 7.84 – 7.78 (m, 4H), 7.71 (m, 1H), 7.62 (m, 1H), 7.50 – 7.43 (m, 3H), 7.28 – 7.20 (m, 3H), 7.09 (m, 2H), 5.90 (dd, J = 3.5, 1.2 Hz, 1H), 5.80 (dd, J = 10.4, 7.9 Hz, 1H), 5.56 (dd, J = 10.4, 3.5 Hz, 1H), 4.79 (d, J = 8.0 Hz, 1H), 4.30 (m, 2H), 4.17 (td, J = 6.5, 1.2 Hz, 1H), 4.10 (m, 1H), 3.63 (m, 1H), 3.33 – 3.18 (m, 2H), 2.06 (s, 3H), 1.96 – 1.71 (m, 4H); ^{13}C NMR (101 MHz, $CDCl_3$) δ 170.5, 165.7, 165.6, 165.3, 136.6, 133.6, 133.3, 133.0, 131.4, 130.0, 129.8, 129.7, 129.5, 129.2, 128.8, 128.6, 128.5, 128.3, 128.1, 127.5, 127.2, 127.1, 126.6, 125.8, 125.0, 124.81, 124.75, 124.7, 123.4, 101.8, 71.8, 71.2, 70.4, 69.7, 68.0, 61.8, 33.1, 29.5, 28.1, 20.8; FAB-HRMS m/z calculated for $[M+Na]^+$ $C_{49}H_{42}O_{10}Na$ 813.2675; found 813.2650

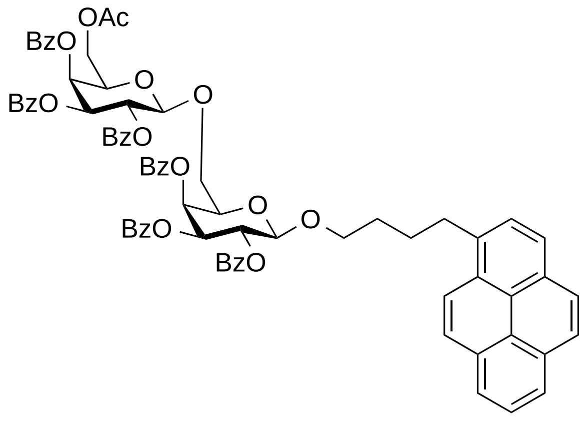

4-(Pyren-1-yl)butyl 6-O-acetyl-2,3,4-tri-O-benzoyl-β-D-galactopyranosyl-(1→6)-2,3,4-tri-O-benzoyl-β-D-galactopyranoside **4_2a**. ^{1}H NMR (600 MHz, $CDCl_3$) δ 8.17 – 8.11 (m, 3H), 8.09 – 8.00 (m, 9H), 8.00 – 7.96 (m, 1H), 7.91 – 7.88 (m, 2H), 7.78 – 7.73 (m, 6H), 7.68 – 7.65 (m,

1H), 7.62 – 7.55 (m, 3H), 7.47 – 7.38 (m, 5H), 7.24 – 7.20 (m, 7H), 7.07 – 7.03 (m, 2H), 5.88 – 5.84 (m, 1H), 5.78 (dd, *J* = 3.5, 1.1 Hz, 1H), 5.76 – 5.69 (m, 2H), 5.49 (m, 2H), 4.78 (d, *J* = 7.9 Hz, 1H), 4.64 (d, *J* = 7.9 Hz, 1H), 4.16 – 4.01 (m, 4H), 3.99 (td, *J* = 6.4, 1.2 Hz, 1H), 3.82 (dd, *J* = 10.5, 7.2 Hz, 1H), 3.77 – 3.71 (m, 1H), 3.31 (m, 5.0 Hz, 1H), 3.14 (m, 2H), 1.91 (s, 3H), 1.82 – 1.46 (m, 4H); ^{13}C NMR (101 MHz, $CDCl_3$) δ 165.6, 165.5, 136.63, 133.58, 133.3, 133.2, 131.4, 130.1, 129.78, 129.76, 129.7, 129.5, 129.1, 128.6, 128.5, 128.4, 128.29, 128.26, 128.1, 127.5, 127.13, 127.10, 126.5, 125.8, 125.0, 124.8, 124.7, 124.7, 123.4, 101.5, 101.2, 77.4, 77.3, 77.1, 76.7, 73.3, 71.7, 71.6, 71.1, 70.0, 69.9, 69.7, 68.8, 68.2, 67.8, 61.5, 33.0, 29.7, 29.4, 28.1, 20.6; ESI-HRMS m/z calculated for $[M+H]^+$ $C_{76}H_{65}O_{18}$ 1265.4170; found 1265.4153

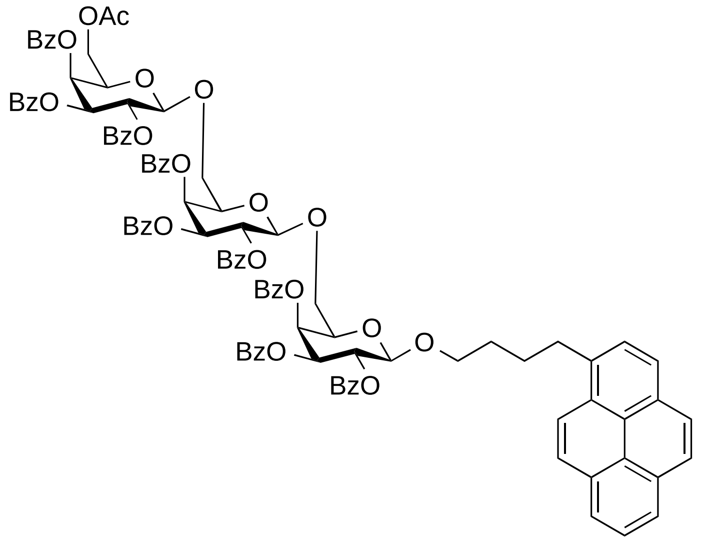

***4-(Pyren-1-yl)butyl 6-O-acetyl-2,3,4-tri-O-benzoyl-β-D-galactopyranosyl-(1→6)-2,3,4-tri-O-benzoyl-β-D-galactopyranosyl-(1→6)-2,3,4-tri-O-benzoyl-β-D-galactopyranoside* $\mathbf{4_3a}$**. ^{1}H NMR (600 MHz, $CDCl_3$) δ 8.19 – 8.11 (m, 3H), 8.10 – 7.98 (m, 10H), 7.94 – 7.89 (m, 4H), 7.81 – 7.75 (m, 8H), 7.68 – 7.64 (m, 1H), 7.60 – 7.53 (m, 3H), 7.51 – 7.38 (m, 11H), 7.38 – 7.34 (m, 2H), 7.28 – 7.24 (m, 9H), 7.09 – 7.04 (m, 2H), 5.92 – 5.89 (m, 1H), 5.88 – 5.86 (m, 1H), 5.79 (dd, *J* = 3.5, 1.1 Hz, 1H), 5.72 (dd, *J* = 10.4, 7.9 Hz, 1H), 5.70 – 5.66 (m, 1H), 5.66 – 5.63 (m, 1H), 5.53 – 5.46 (m, 3H), 4.72 (d, *J* = 7.9 Hz, 1H), 4.66 (d, *J* = 8.0 Hz, 1H), 4.62 (d, *J* = 7.9 Hz, 1H), 4.13 – 4.09 (m, 1H), 4.08 (dd, *J* = 10.0, 4.7 Hz, 1H), 4.04 – 4.00 (m, 2H), 3.96 – 3.91 (m, 2H), 3.87 (dd, *J* = 11.4, 6.5 Hz, 1H), 3.79 – 3.74 (m, 2H), 3.60 (dd, *J* = 10.0, 5.8 Hz, 1H), 3.37 – 3.31 (m, 1H), 3.21 – 3.10 (m, 3H), 1.92 (s, 3H), 1.82 – 1.47 (m, 4H); ESI-HRMS m/z calculated for $[M+NH_4]^+$ $C_{103}H_{90}O_{26}N$ 1756.5751; found 1756.5673

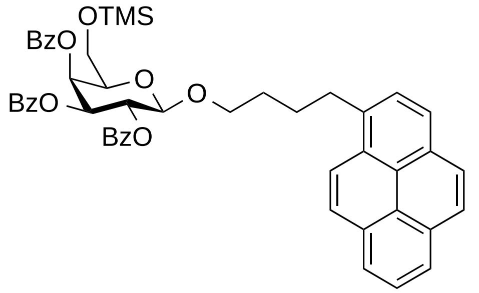

4-(Pyren-1-yl)butyl 6-O-trimethylsilyl-2,3,4-tri-O-benzoyl-β-D-galactopyranoside **4_1b**. ^{1}H NMR (600 MHz, $CDCl_3$) δ 8.20 – 8.16 (m, 3H), 8.12 – 8.06 (m, 3H), 8.04 (s, 2H), 8.04 – 8.00 (m, 2H), 7.85 – 7.80 (m, 4H), 7.72 – 7.69 (m, 1H), 7.65 – 7.60 (m, 1H), 7.51 – 7.46 (m, 2H), 7.47 – 7.42 (m, 1H), 7.28 – 7.25 (m, 2H), 7.25 – 7.20 (m, 1H), 7.11 – 7.06 (m, 2H), 6.01 – 5.96 (m, 1H), 5.78 (dd, *J* = 10.5, 7.9 Hz, 1H), 5.58 (dd, *J* = 10.4, 3.5 Hz, 1H), 4.79 (d, *J* = 7.9 Hz, 1H), 4.12 (dt, *J* = 9.6, 5.5 Hz, 1H), 4.02 (ddd, *J* = 7.3, 5.7, 1.1 Hz, 1H), 3.87 – 3.75 (m, 2H), 3.69 – 3.60 (m, 1H), 3.28 – 3.18 (m, 2H), 1.95 – 1.71 (m, 4H), 0.04 (s, 9H); ^{13}C NMR (101 MHz, $CDCl_3$) δ 165.7, 165.5, 165.3, 136.6, 133.3, 133.1, 132.9, 131.4, 130.9, 130.0, 129.8, 129.7, 129.5, 129.3, 129.0, 128.7, 128.6, 128.5, 128.2, 128.1, 127.5, 127.13, 127.07, 127.0, 126.5, 125.8, 125.0, 124.8, 124.74, 124.68, 123.4, 101.8, 74.1, 72.1, 70.2, 70.0, 68.0, 60.4, 33.1, 29.7, 29.6, 28.1, -0.75; FAB-HRMS m/z calculated for $[M+Na]^+$ $C_{50}H_{48}O_9SiNa$ 843.2965; found 843.2988

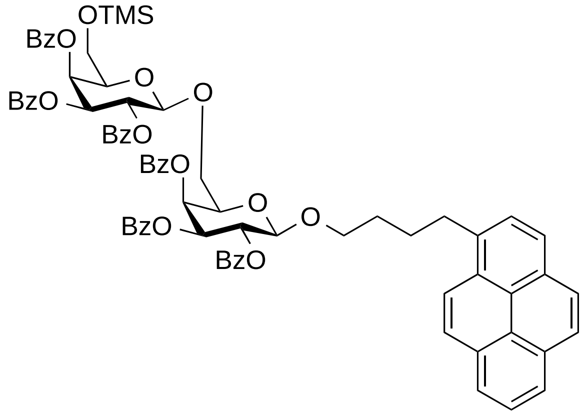

4-(Pyren-1-yl)butyl 6-O-trimethylsilyl-2,3,4-tri-O-benzoyl-β-D-galactopyranosyl-(1→6)-2,3,4-tri-O-benzoyl-β-D-galactopyranoside **4_2b**. ^{1}H NMR (600 MHz, $CDCl_3$) δ 8.20 – 8.16 (m, 2H), 8.16 – 8.13 (m, 2H), 8.11 – 8.07 (m, 5H), 8.05 – 8.04 (m, 2H), 8.04 – 7.99 (m, 1H), 7.94 – 7.91 (m, 2H), 7.81 – 7.76 (m, 6H), 7.68 – 7.66 (m, 1H), 7.65 – 7.58 (m, 2H), 7.50 – 7.45 (m, 6H), 7.45 – 7.40 (m, 3H), 7.27 – 7.22 (m, 5H), 7.08 – 7.04 (m, 2H), 5.93 – 5.89 (m, 2H), 5.76 – 5.70 (m, 2H), 5.56 – 5.49 (m, 2H), 4.81 (d, *J* = 7.9 Hz, 1H), 4.66 (d, *J* = 7.9 Hz, 1H), 4.19 – 4.12 (m, 2H), 3.90 (ddd, *J* = 7.1, 5.8, 1.3 Hz, 1H), 3.88 – 3.82 (m, 1H), 3.74 (dt, *J* = 10.6, 5.5 Hz, 1H), 3.64 – 3.52 (m, 2H), 3.33 – 3.26 (m, 1H), 3.23 – 3.10 (m, 2H), 1.82 – 1.45 (m, 4H), -0.04 (s, 9H); ^{13}C NMR (101 MHz, $CDCl_3$) δ 165.6, 165.51, 165.45, 165.3, 165.2, 136.6, 133.5, 133.3, 133.2, 133.13, 133.12, 132.9, 131.4, 130.9, 130.1, 130.0, 129.82, 129.79, 129.77, 129.7, 129.4, 129.2, 129.1, 129.0, 128.9, 128.59, 128.56, 128.5, 128.4, 128.3, 128.2, 128.1, 127.5, 127.12, 127.07, 126.5, 126.3, 125.8, 125.0, 124.8, 124.73, 124.69, 123.4, 101.5, 101.3, 76.8, 73.8, 73.2,

71.9, 71.7, 70.1, 69.94, 69.87, 68.8, 68.3, 67.7, 59.8, 33.0, 29.3, 28.1; ESI-HRMS m/z calculated for $[M+NH_4]^+$ $C_{77}H_{74}O_{17}SiN$ 1312.4726; found 1312.4706

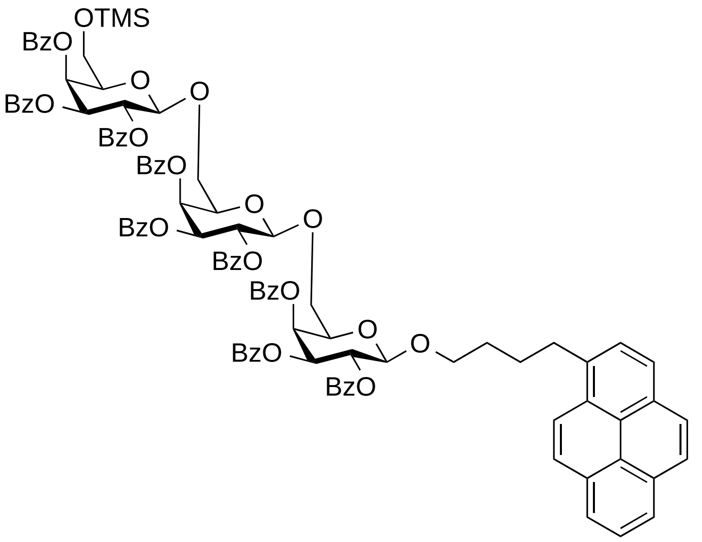

4-(Pyren-1-yl)butyl 6-O-trimethylsilyl-2,3,4-tri-O-benzoyl-β-D-galactopyranosyl-(1→6)-2,3,4-tri-O-benzoyl-β-D-galactopyranosyl-(1→6)-2,3,4-tri-O-benzoyl-β-D-galactopyranoside **4_3b**. ^{1}H NMR (600 MHz, $CDCl_3$) δ 8.19 – 8.12 (m, 4H), 8.09 – 8.05 (m, 6H), 8.06 – 8.02 (m, 4H), 8.02 – 7.98 (m, 2H), 7.95 – 7.89 (m, 4H), 7.82 – 7.77 (m, 10H), 7.67 – 7.64 (m, 1H), 7.59 – 7.54 (m, 3H), 7.49 – 7.39 (m, 8H), 7.38 – 7.34 (m, 2H), 7.28 – 7.23 (m, 8H), 7.09 – 7.05 (m, 2H), 5.92 (dd, *J* = 3.5, 1.2 Hz, 1H), 5.88 (dd, *J* = 3.5, 1.2 Hz, 1H), 5.87 (dd, *J* = 3.7, 1.1 Hz, 1H), 5.72 (dd, *J* = 10.4, 7.9 Hz, 1H), 5.68 (dd, *J* = 10.4, 7.9 Hz, 1H), 5.63 (dd, *J* = 10.4, 7.9 Hz, 1H), 5.55 – 5.46 (m, 3H), 4.72 (d, *J* = 7.9 Hz, 1H), 4.67 (d, *J* = 8.0 Hz, 1H), 4.59 (d, *J* = 7.9 Hz, 1H), 4.15 – 4.10 (m, 1H), 4.06 (dd, *J* = 10.3, 4.7 Hz, 1H), 4.02 (ddd, *J* = 7.1, 5.7, 1.3 Hz, 1H), 3.93 (dd, *J* = 10.0, 7.0 Hz, 1H), 3.84 (ddd, *J* = 8.4, 5.4, 1.2 Hz, 1H), 3.80 – 3.74 (m, 2H), 3.61 (dd, *J* = 9.9, 5.6 Hz, 1H), 3.40 (dd, *J* = 10.0, 5.5 Hz, 1H), 3.37 – 3.31 (m, 2H), 3.20 – 3.12 (m, 2H), 1.84 – 1.47 (m, 4H), -0.07 (s, 9H); ^{13}C NMR (151 MHz, $CDCl_3$) δ 165.6, 165.5, 165.33, 165.29, 165.11, 165.06, 136.6, 133.34, 133.27, 133.2, 133.12, 133.07, 132.9, 131.4, 130.9, 130.2, 130.1, 130.0, 129.9, 129.82, 129.80, 129.77, 129.72, 129.67, 129.53, 129.47, 129.34, 129.28, 129.05, 128.98, 128.7, 128.6, 128.5, 128.4, 128.34, 128.30, 128.20, 128.17, 128.13, 128.07, 127.5, 127.10, 127.06, 126.5, 125.8, 125.0, 124.8, 124.71, 124.68, 123.4, 101.6, 101.0, 100.9, 73.7, 72.9, 72.4, 72.1, 71.8, 71.6, 70.0, 69.9, 68.6, 67.9, 67.7, 67.5, 66.4, 59.3, 33.0, 29.4, 28.1, -0.9; ESI-HRMS m/z calculated for $[M+NH_4]^+$ $C_{104}H_{96}O_{25}SiN$ 1786.6041; found 1787.6024

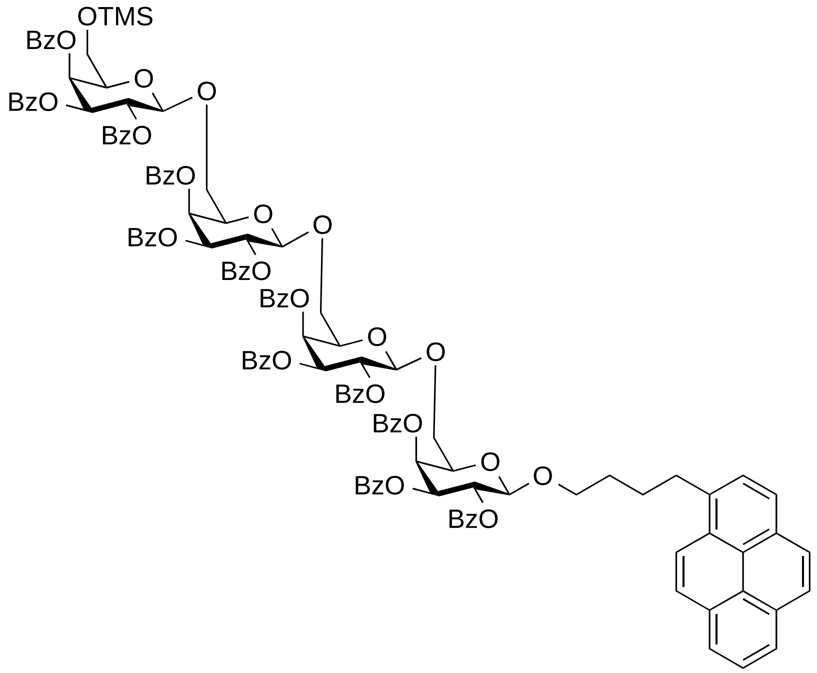

4-(Pyren-1-yl)butyl 6-O-trimethylsilyl-2,3,4-tri-O-benzoyl-β-D-galactopyranosyl-(1→6)-2,3,4-tri-O-benzoyl-β-D-galactopyranosyl-(1→6)-2,3,4-tri-O-benzoyl-β-D-galactopyranosyl-(1→6)-2,3,4-tri-O-benzoyl-β-D-galactopyranoside **4_4b**. ^{1}H NMR (600 MHz, $CDCl_3$) δ 8.19 – 8.11 (m, 3H), 8.08 – 7.99 (m, 13H), 7.96 – 7.93 (m, 5H), 7.91 (dd, J = 8.4, 1.3 Hz, 3H), 7.82 – 7.74 (m, 13H), 7.64 (d, J = 7.8 Hz, 1H), 7.55 – 7.49 (m, 2H), 7.49 – 7.41 (m, 7H), 7.39 – 7.34 (m, 9H), 7.28 – 7.23 (m, 10H), 7.22 – 7.17 (m, 1H), 7.05 (dd, J = 8.5, 7.6 Hz, 2H), 5.90 – 5.86 (m, 3H), 5.83 (dd, J = 3.6, 1.2 Hz, 1H), 5.71 – 5.64 (m, 2H), 5.63 – 5.56 (m, 2H), 5.54 – 5.44 (m, 4H), 4.75 (d, J = 7.9 Hz, 1H), 4.63 (d, J = 7.9 Hz, 1H), 4.52 (d, J = 7.8 Hz, 1H), 4.43 (d, J = 7.8 Hz, 1H), 4.12 (t, J = 6.1 Hz, 1H), 4.06 (dd, J = 10.4, 4.7 Hz, 1H), 4.02 (t, J = 6.7 Hz, 1H), 3.93 (t, J = 6.4 Hz, 1H), 3.86 – 3.76 (m, 2H), 3.74 – 3.67 (m, 2H), 3.57 – 3.50 (m, 1H), 3.36 (dd, J = 10.0, 5.5 Hz, 1H), 3.32 – 3.23 (m, 2H), 3.18 – 3.08 (m, 2H), 1.58 – 1.52 (m, 4H), -0.07 (s, 9H); ^{13}C NMR (151 MHz, $CDCl_3$) δ 165.6, 165.49, 165.47, 165.42, 165.38, 165.3, 165.11, 165.05, 165.0, 164.9, 136.6, 133.3, 133.2, 133.14, 133.13, 133.10, 133.05, 132.9, 131.4, 130.9, 130.2, 130.09, 130.05, 130.0, 129.93, 129.86, 129.82, 129.78, 129.74, 129.70, 129.66, 129.6, 129.5, 129.29, 129.25, 129.09, 129.06, 129.0, 128.5, 128.43, 128.38, 128.3, 128.21, 128.17, 128.14, 128.06, 127.5, 127.10, 127.06, 126.5, 125.8, 125.0, 124.8, 124.71, 124.69, 123.4, 101.5, 101.1, 100.6, 73.7, 72.8, 72.1, 72.0, 71.73, 71.69, 70.0, 69.9, 69.8, 68.6, 67.7, 67.4, 66.5, 59.2, 33.0, 29.7, 29.3, 28.0, -0.8; ESI-HRMS m/z calculated for $[M+Na]^+$ $C_{131}H_{114}O_{33}Si$ 2265.6909; found 2265.6895

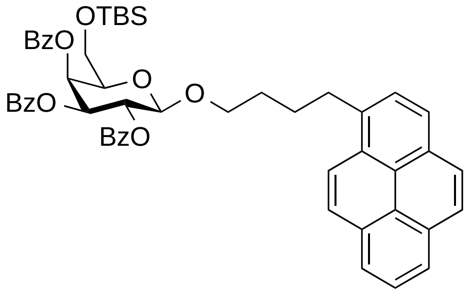

4-(Pyren-1-yl)butyl 6-O-tert-butyldimethylsilyl-2,3,4-tri-O-benzoyl-β-D-galactopyranoside **4_1c**. ^{1}H NMR (600 MHz, $CDCl_3$) δ 8.20 – 8.14 (m, 4H), 8.13 – 8.05 (m, 4H), 8.04 (s, 2H), 8.04 – 7.98 (m, 2H), 7.83 – 7.79 (m, 4H), 7.71 – 7.69 (m, 1H), 7.64 – 7.60 (m, 1H), 7.55 – 7.51 (m, 2H), 7.50 – 7.46 (m, 2H), 7.46 – 7.42 (m, 1H), 7.28 – 7.23 (m, 2H), 7.24 – 7.20 (m, 1H), 7.11 – 7.06 (m, 2H), 5.96 (dd, J = 3.5, 1.2 Hz, 1H), 5.77 (dd, J = 10.5, 8.0 Hz, 1H), 5.60 (dd, J = 10.4, 3.5 Hz, 1H), 4.78 (d, J = 8.0 Hz, 1H), 4.11 (dt, J = 9.5, 5.5 Hz, 1H), 4.02 (ddd, J = 7.3, 5.9, 1.4 Hz, 1H), 3.89 – 3.79 (m, 2H), 3.66 – 3.60 (m, 1H), 3.31 – 3.16 (m, 2H), 1.94 – 1.72 (m, 4H), 0.85 (s, 9H), -0.01 (s, 3H), -0.05 (s, 3H); ^{13}C NMR (151 MHz, $CDCl_3$) δ 165.7, 165.5, 165.3, 165.0, 136.6, 133.7, 133.6, 133.5, 133.3, 133.1, 132.9, 131.4, 130.9, 130.0, 129.9, 129.8, 129.7, 129.5, 129.3, 129.0, 128.7, 128.58, 128.55, 128.52, 128.49, 128.4, 128.22, 128.16, 128.1, 127.5, 127.13, 127.07, 126.5, 125.8, 125.0, 124.8, 124.73, 124.67, 123.4, 101.8, 99.3, 76.8, 74.1, 72.3, 72.0, 71.7, 70.2, 70.1, 68.0, 67.9, 65.9, 64.9, 61.0, 33.1, 29.7, 29.53, 29.45, 28.1, 25.75, 25.71, -5.6; ESI-HRMS m/z calculated for $[M+NH_4]^+$ $C_{53}H_{58}O_9SiN$ 880.3881; found 880.3871

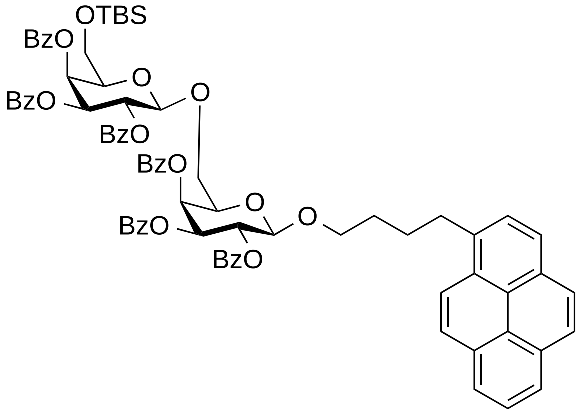

4-(Pyren-1-yl)butyl 6-O-tert-butyldimethylsilyl-2,3,4-tri-O-benzoyl-β-D-galactopyranosyl-(1→6)-2,3,4-tri-O-benzoyl-β-D-galactopyranoside **4_2c**. ^{1}H NMR (600 MHz, $CDCl_3$) δ 8.20 – 8.12 (m, 2H), 8.12 – 8.07 (m, 6H), 8.07 – 7.99 (m, 5H), 7.94 – 7.91 (m, 2H), 7.82 – 7.75 (m, 6H), 7.68 – 7.66 (m, 1H), 7.65 – 7.59 (m, 2H), 7.51 – 7.46 (m, 4H), 7.46 – 7.40 (m, 2H), 7.28 – 7.17 (m, 7H), 7.07 – 7.03 (m, 2H), 5.92 – 5.89 (m, 2H), 5.75 – 5.72 (m, 2H), 5.73 – 5.70 (m, 1H), 5.57 (dd, J = 10.4, 3.4 Hz, 1H), 5.52 (dd, J = 10.4, 3.6 Hz, 1H), 4.80 (d, J = 7.9 Hz, 1H), 4.65 (d, J = 7.9 Hz, 1H), 4.20 – 4.12 (m, 2H), 3.91 (ddd, J = 8.1, 5.8, 1.3 Hz, 1H), 3.83 (dd, J = 11.4, 8.2 Hz, 1H), 3.74 – 3.58 (m, 2H), 3.32 – 3.22 (m, 1H), 3.20 – 3.09 (m, 2H), 1.80 – 1.71 (m, 2H), 1.65 – 1.42 (m, 2H), 0.79 (s, 9H), -0.09 (s, 3H), -0.12 (s, 3H); ^{13}C NMR (151 MHz, $CDCl_3$) δ 165.6, 165.51, 165.46, 165.4, 165.3, 165.2, 136.6, 133.5, 133.3, 133.2, 133.1, 132.9, 131.4,

130.9, 130.1, 130.0, 129.84, 129.79, 129.7, 129.5, 129.44, 129.37, 129.2, 129.0, 128.9, 128.59, 128.56, 128.5, 128.3, 128.24, 128.22, 128.1, 127.5, 127.12, 127.07, 126.5, 125.8, 125.0, 124.8, 124.72, 124.70, 123.4, 101.5, 101.4, 73.9, 73.2, 71.9, 71.7, 70.1, 69.93, 69.87, 68.8, 68.4, 67.7, 60.4, 33.0, 29.7, 29.5, 29.3, 28.1, 25.7, -5.66, -5.72; ESI-HRMS m/z calculated for $[M+NH_4]^+$ $C_{80}H_{80}O_{17}SiN$ 1354.5195; found 1354.5168

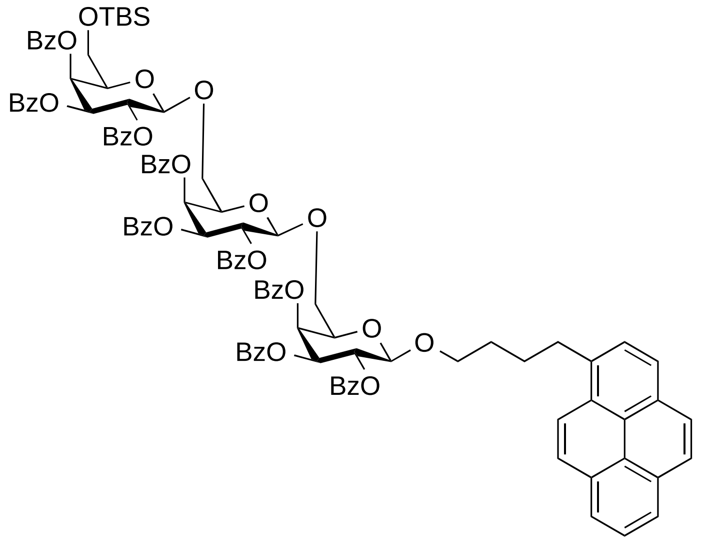

4-(Pyren-1-yl)butyl 6-O-tert-butyldimethylsilyl-2,3,4-tri-O-benzoyl-β-D-galactopyranosyl-(1→6)-2,3,4-tri-O-benzoyl-β-D-galactopyranosyl-(1→6)-2,3,4-tri-O-benzoyl-β-D-galactopyranoside **4_3c**. ^{1}H NMR (600 MHz, $CDCl_3$) δ 8.19 – 8.11 (m, 3H), 8.10 – 8.05 (m, 6H), 8.05 – 7.98 (m, 8H), 7.94 – 7.89 (m, 4H), 7.80 – 7.77 (m, 6H), 7.68 – 7.64 (m, 1H), 7.60 – 7.54 (m, 3H), 7.49 – 7.39 (m, 9H), 7.40 – 7.31 (m, 3H), 7.28 – 7.22 (m, 8H), 7.22 – 7.18 (m, 1H), 7.08 – 7.03 (m, 2H), 5.91 (dd, *J* = 3.6, 1.2 Hz, 1H), 5.87 – 5.85 (m, 2H), 5.71 (dd, *J* = 10.4, 8.0 Hz, 1H), 5.66 (dd, *J* = 10.5, 7.9 Hz, 1H), 5.61 (dd, *J* = 10.4, 7.8 Hz, 1H), 5.54 – 5.47 (m, 3H), 4.71 (d, *J* = 7.9 Hz, 1H), 4.65 (d, *J* = 8.0 Hz, 1H), 4.57 (d, *J* = 7.8 Hz, 1H), 4.10 (ddd, *J* = 6.8, 4.9, 1.3 Hz, 1H), 4.05 (dd, *J* = 10.2, 4.8 Hz, 1H), 4.01 (td, *J* = 5.6, 2.7 Hz, 1H), 3.93 (dd, *J* = 9.9, 7.0 Hz, 1H), 3.85 – 3.80 (m, 1H), 3.79 – 3.72 (m, 2H), 3.58 (dd, *J* = 9.9, 5.6 Hz, 1H), 3.44 (dd, *J* = 9.9, 5.4 Hz, 1H), 3.39 – 3.31 (m, 2H), 3.20 – 3.11 (m, 2H), 1.84 – 1.46 (m, 4H), 0.78 (s, 9H), -0.13 (s, 3H), -0.18 (s, 3H); ^{13}C NMR (151 MHz, $CDCl_3$) δ 165.6, 165.48, 165.45, 165.4, 165.34, 165.29, 165.2, 165.11, 165.08, 136.6, 133.4, 133.3, 133.19, 133.16, 133.13, 133.10, 133.07, 132.9, 131.4, 130.9, 130.1, 130.0, 129.82, 129.80, 129.73, 129.68, 129.50, 129.45, 129.3, 129.2, 129.03, 128.97, 128.5, 128.4, 128.3, 128.22, 128.19, 128.1, 127.5, 127.11, 127.07, 126.5, 125.8, 125.0, 124.8, 124.72, 124.68, 123.4, 101.6, 101.0, 100.9, 73.7, 72.9, 72.4, 71.9, 71.7, 71.6, 70.1, 70.0, 69.9, 68.6, 67.9, 67.6, 67.4, 59.9, 33.0, 29.7, 29.5, 29.4, 28.0, 25.7, -5.73, -5.77; ESI-HRMS m/z calculated for $[M+NH_4]^+$ $C_{107}H_{102}O_{25}SiN$ 1828.6510; found 1828.6497

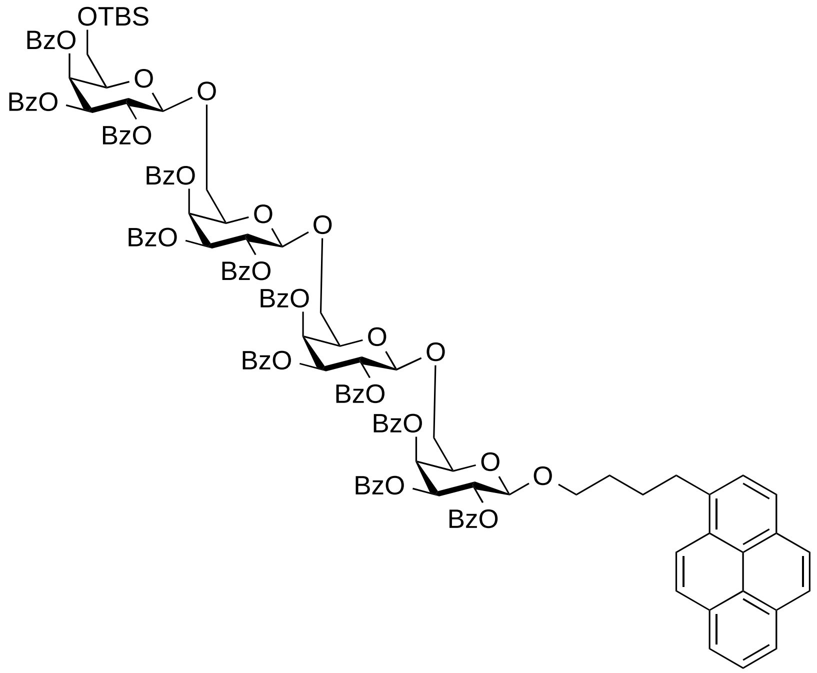

4-(Pyren-1-yl)butyl 6-O-tert-butyldimethylsilyl-2,3,4-tri-O-benzoyl-β-D-galactopyranosyl-(1→6)-2,3,4-tri-O-benzoyl-β-D-galactopyranosyl-(1→6)-2,3,4-tri-O-benzoyl-β-D-galactopyranosyl-(1→6)-2,3,4-tri-O-benzoyl-β-D-galactopyranoside **4_4c**. ^{1}H NMR (600 MHz, $CDCl_3$) δ 8.19 – 8.11 (m, 3H), 8.09 – 8.05 (m, 7H), 8.03 (s, 2H), 8.03 – 7.99 (m, 6H), 7.95 – 7.89 (m, 6H), 7.80 – 7.76 (m, 12H), 7.66 – 7.64 (m, 1H), 7.58 – 7.50 (m, 3H), 7.49 – 7.41 (m, 9H), 7.39 – 7.34 (m, 7H), 7.28 – 7.22 (m, 11H), 7.22 – 7.18 (m, 1H), 7.07 – 7.03 (m, 2H), 5.91 – 5.82 (m, 4H), 5.70 (dd, *J* = 10.4, 7.9 Hz, 1H), 5.66 (dd, *J* = 10.4, 7.9 Hz, 1H), 5.63 – 5.54 (m, 2H), 5.52 – 5.48 (m, 3H), 5.44 (dd, *J* = 10.4, 3.4 Hz, 1H), 4.72 (d, *J* = 7.9 Hz, 1H), 4.63 (d, *J* = 8.0 Hz, 1H), 4.50 (d, *J* = 7.8 Hz, 1H), 4.40 (d, *J* = 7.9 Hz, 1H), 4.11 (dd, *J* = 6.8, 5.1 Hz, 1H), 4.05 (dd, *J* = 10.4, 4.5 Hz, 1H), 3.97 (t, *J* = 6.5 Hz, 1H), 3.91 (t, *J* = 6.3 Hz, 1H), 3.87 – 3.81 (m, 1H), 3.80 – 3.74 (m, 2H), 3.74 – 3.68 (m, 2H), 3.51 (dd, *J* = 9.6, 5.6 Hz, 1H), 3.40 (dd, *J* = 9.9, 5.4 Hz, 1H), 3.35 – 3.24 (m, 2H), 3.20 – 3.08 (m, 2H), 1.84 – 1.69 (m, 2H), 1.54 – 1.41 (m, 2H), 0.78 (s, 9H), -0.14 (s, 3H), -0.19 (s, 3H); ^{13}C NMR (151 MHz, $CDCl_3$) δ 165.6, 165.4, 165.33, 165.27, 165.13, 165.05, 165.0, 164.9, 136.6, 133.4, 133.3, 133.2, 133.14, 133.06, 132.9, 131.4, 130.9, 130.2, 130.1, 130.0, 129.9, 129.82, 129.78, 129.73, 129.68, 129.6, 129.51, 129.49, 129.44, 129.35, 129.3, 129.2, 129.1, 129.03, 128.97, 128.6, 128.52, 128.48, 128.42, 128.38, 128.35, 128.3, 128.20, 128.16, 128.1, 127.5, 127.10, 127.06, 126.5, 125.8, 125.0, 124.8, 124.71, 124.68, 123.4, 101.5, 101.1, 101.0, 100.6, 73.7, 72.9, 72.2, 72.1, 71.9, 71.71, 71.65, 70.1, 69.9, 68.6, 67.7, 67.4, 59.7, 34.1, 33.0, 29.7, 29.3, 28.0, -5.74, -5.77; ESI-HRMS m/z calculated for $[M+NH_4]^+$ $C_{134}H_{124}O_{33}SiN$ 2302.7824; found 2302.7797

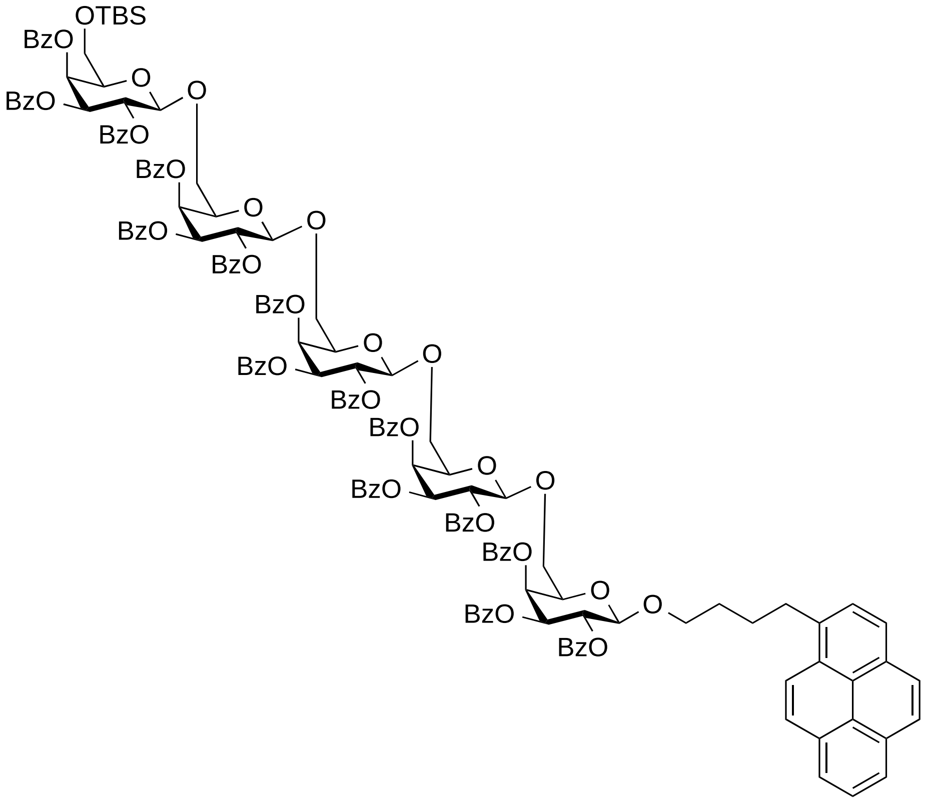

4-(Pyren-1-yl)butyl 6-O-tert-butyldimethylsilyl-2,3,4-tri-O-benzoyl-β-D-galactopyranosyl-(1→6)-2,3,4-tri-O-benzoyl-β-D-galactopyranosyl-(1→6)-2,3,4-tri-O-benzoyl-β-D-galactopyranosyl-(1→6)-2,3,4-tri-O-benzoyl-β-D-galactopyranosyl-(1→6)-2,3,4-tri-O-benzoyl-β-D-galactopyranoside **4_5c**. ^{1}H NMR (600 MHz, $CDCl_3$) δ 8.18 – 8.11 (m, 3H), 8.09 – 8.05 (m, 6H), 8.03 (s, 2H), 8.03 – 7.99 (m, 6H), 7.99 – 7.95 (m, 4H), 7.94 – 7.89 (m, 8H), 7.81 – 7.75 (m, 15H), 7.71 – 7.68 (m, 1H), 7.66 – 7.64 (m, 1H), 7.50 – 7.40 (m, 12H), 7.37 – 7.31 (m, 10H), 7.27 – 7.22 (m, 13H), 7.07 – 7.03 (m, 3H), 5.88 – 5.82 (m, 5H), 5.69 (dd, J = 10.4, 8.0 Hz, 1H), 5.64 (dd, J = 10.4, 7.9 Hz, 1H), 5.59 – 5.52 (m, 3H), 5.51 – 5.42 (m, 5H), 4.71 (d, J = 7.9 Hz, 1H), 4.62 (d, J = 7.9 Hz, 1H), 4.50 (d, J = 7.9 Hz, 1H), 4.40 (d, J = 7.8 Hz, 1H), 4.36 (d, J = 7.8 Hz, 1H), 4.10 – 4.06 (m, 1H), 4.03 (dd, J = 10.3, 4.7 Hz, 1H), 3.97 (t, J = 6.8 Hz, 1H), 3.91 – 3.86 (m, 1H), 3.87 – 3.81 (m, 2H), 3.77 (t, J = 7.3 Hz, 1H), 3.73 (dd, J = 10.6, 7.0 Hz, 2H), 3.67 – 3.63 (m, 1H), 3.63 – 3.59 (m, 1H), 3.50 (dd, J = 10.0, 6.0 Hz, 1H), 3.35 (dd, J = 9.9, 5.3 Hz, 1H), 3.30 – 3.25 (m, 2H), 3.24 – 3.19 (m, 2H), 3.17 – 3.09 (m, 2H), 1.80 – 1.64 (m, 2H), 1.52 – 1.39 (m, 2H), 0.77 (s, 9H), -0.16 (s, 3H), -0.21 (s, 3H); ESI-HRMS m/z calculated for $[M+NH_4]^+$ $C_{161}H_{146}O_{41}SiN$ 2776.9140; found 2776.9135

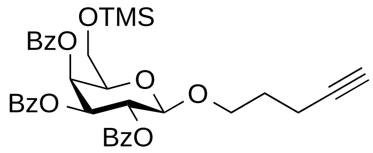

***4-Pentynyl 6-O-trimethylsilyl-2,3,4-tri-O-benzoyl-β-D-galactopyranoside* 13$_1$b**. ^{1}H NMR (600 MHz, $CDCl_3$) δ 8.12 – 8.08 (m, 2H), 8.02 – 7.98 (m, 2H), 7.85 – 7.81 (m, 2H), 7.66 – 7.62 (m, 1H), 7.56 – 7.52 (m, 2H), 7.52 – 7.48 (m, 2H), 7.48 – 7.44 (m, 1H), 7.43 – 7.39 (m, 2H), 7.29 – 7.26 (m, 1H), 5.98 (d, J = 3.5 Hz, 1H), 5.76 (dd, J = 10.4, 7.8 Hz, 1H), 5.59 (dd, J = 10.4, 3.5 Hz, 1H), 4.81 (d, J = 7.9 Hz, 1H), 4.10 (dt, J = 9.6, 5.5 Hz, 1H), 4.05 (t, J = 6.7 Hz, 1H), 3.84 (dd, J = 10.1, 5.9 Hz, 1H), 3.78 (dd, J = 10.1, 7.3 Hz, 1H), 3.76 – 3.72 (m, 1H), 2.19 (td, J = 7.0, 2.6 Hz, 2H), 1.88 – 1.86 (m, 1H), 1.89 – 1.81 (m, 1H), 1.80 – 1.72 (m, 1H), 0.06 (s, 9H); ^{13}C NMR (151 MHz, $CDCl_3$) δ 165.7, 165.5, 165.4, 133.4, 133.18, 133.15, 130.00, 129.95, 129.8, 129.7, 129.5, 129.0, 128.8, 128.56, 128.55, 128.4, 128.3, 101.9, 83.5, 74.13, 72.09, 70.0, 68.7, 68.6, 68.0, 60.4, 28.3, 14.8; ESI-HRMS m/z calculated for $[M+Na]^+$ $C_{35}H_{38}O_9SiNa$ 653.2183; found 653.2185

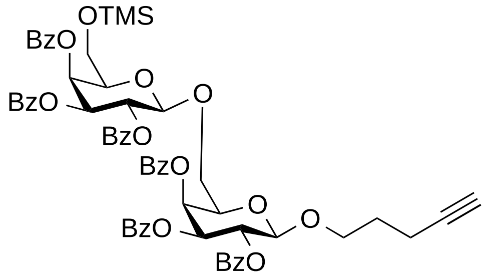

***4-Pentynyl 6-O-trimethylsilyl-2,3,4-tri-O-benzoyl-β-D-galactopyranosyl-(1→6)-2,3,4-tri-O-benzoyl-β-D-galactopyranoside* 13$_2$b**. ^{1}H NMR (600 MHz, $CDCl_3$) δ 8.11 – 8.08 (m, 4H), 8.00 – 7.95 (m, 4H), 7.82 – 7.79 (m, 2H), 7.66 – 7.61 (m, 2H), 7.55 – 7.51 (m, 3H), 7.52 – 7.48 (m, 4H), 7.47 – 7.44 (m, 1H), 7.42 – 7.38 (m, 5H), 7.28 – 7.25 (m, 5H), 5.94 (dd, J = 3.5, 1.1 Hz, 1H), 5.91 (d, J = 3.5 Hz, 1H), 5.75 (dd, J = 10.4, 7.8 Hz, 1H), 5.71 (dd, J = 10.4, 8.0 Hz, 1H), 5.57 (dd, J = 10.4, 3.5 Hz, 1H), 5.54 (dd, J = 10.4, 3.6 Hz, 1H), 4.86 (d, J = 7.8 Hz, 1H), 4.67 (d, J = 7.9 Hz, 1H), 4.21 – 4.15 (m, 2H), 4.00 – 3.96 (m, 1H), 3.91 – 3.85 (m, 1H), 3.71 (dt, J = 10.2, 5.2 Hz, 1H), 3.66 (dd, J = 10.1, 5.7 Hz, 1H), 3.59 (dd, J = 10.1, 7.7 Hz, 1H), 3.44 – 3.38 (m, 1H), 2.10 – 2.01 (m, 2H), 1.86 (t, J = 2.6 Hz, 1H), 1.68 – 1.44 (m, 2H), -0.02 (s, 9H); ^{13}C NMR (151 MHz, $CDCl_3$) δ 165.5, 165.3, 165.2, 133.5, 133.3, 133.24, 133.20, 133.15, 130.1, 130.04, 129.98, 129.80, 129.76, 129.7, 129.4, 128.9, 128.6, 128.43, 128.37, 128.3, 101.6, 101.4, 83.5, 73.9, 73.3, 72.0, 71.7, 70.1, 69.83, 68.78, 68.7, 68.3, 68.1, 67.7, 59.9, 28.2, 14.7, -0.8; ESI-HRMS m/z calculated for $[M+Na]^+$ $C_{62}H_{60}O_{17}SiNa$ 1127.3497; found 1127.3496

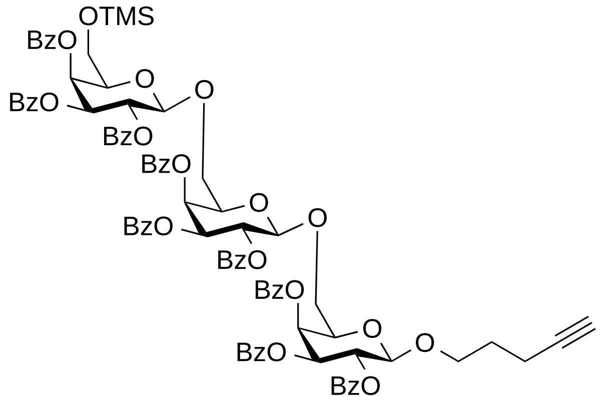

4-Pentynyl 6-O-trimethylsilyl-2,3,4-tri-O-benzoyl-β-D-galactopyranosyl-(1→6)-2,3,4-tri-O-benzoyl-β-D-galactopyranosyl-(1→6)-2,3,4-tri-O-benzoyl-β-D-galactopyranoside **13_3b**. ^{1}H NMR (600 MHz, $CDCl_3$) δ 8.10 – 8.06 (m, 5H), 8.05 – 8.02 (m, 2H), 7.99 – 7.95 (m, 4H), 7.95 – 7.92 (m, 2H), 7.82 – 7.77 (m, 7H), 7.62 – 7.59 (m, 1H), 7.60 – 7.55 (m, 2H), 7.54 – 7.50 (m, 2H), 7.50 – 7.47 (m, 1H), 7.47 – 7.42 (m, 9H), 7.42 – 7.37 (m, 5H), 7.28 – 7.24 (m, 5H), 5.95 (dd, *J* = 3.5, 1.2 Hz, 1H), 5.88 (dd, *J* = 3.4, 1.1 Hz, 1H), 5.87 (dd, *J* = 3.5, 1.2 Hz, 1H), 5.73 – 5.67 (m, 2H), 5.63 (dd, *J* = 10.4, 7.9 Hz, 1H), 5.56 – 5.49 (m, 3H), 4.79 (d, *J* = 7.9 Hz, 1H), 4.68 (d, *J* = 8.0 Hz, 1H), 4.63 (d, *J* = 7.9 Hz, 1H), 4.16 – 4.12 (m, 1H), 4.11 – 4.06 (m, 2H), 3.96 (dd, *J* = 9.9, 7.1 Hz, 1H), 3.88 – 3.84 (m, 1H), 3.81 (dd, *J* = 10.4, 7.1 Hz, 1H), 3.74 (dt, *J* = 10.4, 5.3 Hz, 1H), 3.65 (dd, *J* = 10.0, 5.6 Hz, 1H), 3.47 – 3.42 (m, 1H), 3.40 (dd, *J* = 10.0, 5.5 Hz, 1H), 3.33 (dd, *J* = 10.0, 8.4 Hz, 1H), 2.09 – 2.02 (m, 2H), 1.85 (t, *J* = 2.6 Hz, 1H), 1.57 – 1.45 (m, 2H), -0.07 (s, 9H); ^{13}C NMR (151 MHz, $CDCl_3$) δ 165.5, 165.3, 165.12, 165.07, 165.06, 133.4, 133.2, 133.11, 133.06, 130.08, 130.07, 130.0, 129.9, 129.84, 129.80, 129.7, 129.5, 129.4, 129.1, 128.6, 128.5, 128.40, 128.36, 128.22, 128.18, 101.6, 101.1, 101.0, 73.7, 73.0, 72.5, 72.0, 71.8, 71.7, 70.0, 70.0, 69.9, 68.6, 68.1, 67.9, 67.7, 66.4, 59.3, 28.2, 14.7, -0.9; ESI-HRMS m/z calculated for $[M+NH_4]^+$ $C_{89}H_{86}O_{25}SiN$ 1596.5258; found 1596.5264

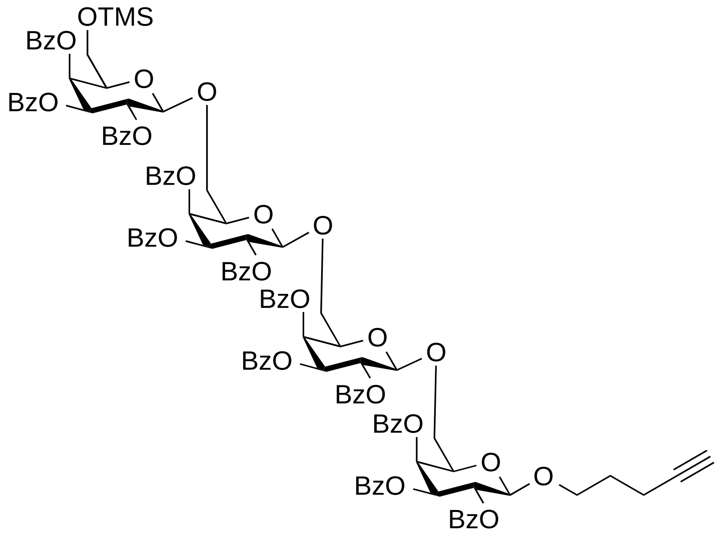

4-Pentynyl 6-O-trimethylsilyl-2,3,4-tri-O-benzoyl-β-D-galactopyranosyl-(1→6)-2,3,4-tri-O-benzoyl-β-D-galactopyranosyl-(1→6)-2,3,4-tri-O-benzoyl-β-D-galactopyranosyl-(1→6)-2,3,4-tri-O-benzoyl-β-D-galactopyranoside **13_4b**. ^{1}H NMR (600 MHz, $CDCl_3$) δ 8.09 – 8.05 (m, 5H), 8.04 – 7.99 (m, 7H), 7.98 – 7.93 (m, 7H), 7.93 – 7.90 (m, 2H), 7.81 – 7.77 (m, 9H), 7.58 – 7.48 (m, 8H), 7.47 – 7.42 (m, 5H), 7.41 – 7.37 (m, 12H), 7.28 – 7.16 (m, 6H), 5.92 (d, *J* = 3.4 Hz, 1H), 5.89 – 5.86 (m, 2H), 5.85 (d, *J* = 3.8 Hz, 1H), 5.73 – 5.64 (m, 2H), 5.64 – 5.56 (m, 2H), 5.57 – 5.50 (m, 2H), 5.50 – 5.45 (m, 2H), 4.82 (d, *J* = 7.9 Hz, 1H), 4.65 (d, *J* = 8.0 Hz, 1H), 4.57 (d, *J* = 7.9 Hz, 1H), 4.43 (d, *J* = 7.8 Hz, 1H), 4.30 (d, *J* = 7.2 Hz, 1H), 4.16 – 4.12 (m, 1H), 4.11 – 4.05 (m, 4H), 3.96 (t, *J* = 6.6 Hz, 1H), 3.91 – 3.86 (m, 1H), 3.85 – 3.77 (m, 2H), 3.74 – 3.66 (m, 2H), 3.59 (dd, *J* = 9.7, 5.7 Hz, 1H), 3.41 – 3.33 (m, 2H), 3.32 – 3.26 (m, 2H), 1.84 (t, *J* = 2.6 Hz, 1H), -0.07 (s, 9H); ESI-HRMS m/z calculated for $[M+Na]^+$ $C_{116}H_{104}O_{33}SiNa$ 2075.6127; found 2075.6118

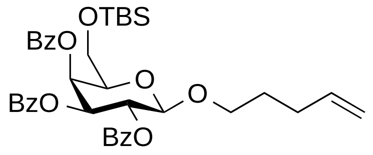

4-Pentenyl 6-O-tert-butyldimethylsilyl-2,3,4-tri-O-benzoyl-β-D-galactopyranoside **14_1c**. ^{1}H NMR (600 MHz, $CDCl_3$) δ 8.12 – 8.09 (m, 2H), 8.01 – 7.98 (m, 2H), 7.84 – 7.81 (m, 2H), 7.65 – 7.61 (m, 1H), 7.55 – 7.48 (m, 3H), 7.47 – 7.43 (m, 1H), 7.42 – 7.39 (m, 2H), 7.29 – 7.25 (m, 2H), 5.97 (dd, *J* = 3.5, 1.2 Hz, 1H), 5.77 (dd, *J* = 10.4, 8.0 Hz, 1H), 5.69 (ddt, *J* = 16.9, 10.3, 6.6 Hz, 1H), 5.62 (dd, *J* = 10.4, 3.5 Hz, 1H), 4.89 – 4.83 (m, 2H), 4.79 (d, *J* = 7.9 Hz, 1H), 4.07 – 3.98 (m, 2H), 3.88 (dd, *J* = 10.0, 6.0 Hz, 1H), 3.82 (dd, *J* = 10.0, 7.5 Hz, 1H), 3.64 – 3.56 (m, 1H), 2.10 – 1.95 (m, 2H), 1.77 – 1.61 (m, 2H), 0.87 (s, 9H), 0.02 (s, 3H), -0.02 (s, 3H); ^{13}C NMR (151 MHz, $CDCl_3$) δ 165.7, 165.5, 165.3, 137.8 (10), 133.3, 133.14, 133.13, 129.9, 129.8, 129.7, 129.6, 129.0, 128.6, 128.4, 128.2, 114.9, 101.7, 74.2, 72.0, 70.1, 69.5, 68.0, 61.0, 29.8, 28.6, 25.8, 18.2, -5.59, -5.62; ESI-HRMS m/z calculated for $[M+Na]^+$ $C_{38}H_{46}O_9SiNa$ 697.2808; found 697.2797

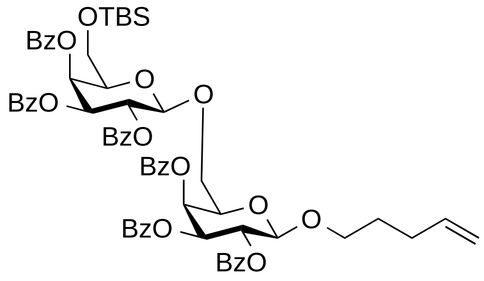

4-Pentenyl 6-O-tert-butyldimethylsilyl-2,3,4-tri-O-benzoyl-β-D-galactopyranosyl-(1→6)-2,3,4-tri-O-benzoyl-β-D-galactopyranoside **14_2c**. ^{1}H NMR (600 MHz, $CDCl_3$) δ 8.12 – 8.08 (m, 4H), 7.99 – 7.94 (m, 4H), 7.82 – 7.78 (m, 4H), 7.66 – 7.61 (m, 2H), 7.54 – 7.47 (m, 6H), 7.47 – 7.42 (m, 2H), 7.41 – 7.37 (m, 4H), 7.29 – 7.24 (m, 4H), 5.93 (dd, *J* = 3.5, 1.1 Hz, 1H), 5.90 (dd, *J* = 3.6, 1.1 Hz, 1H), 5.76 – 5.69 (m, 2H), 5.64 – 5.56 (m, 2H), 5.54 (dd, *J* = 10.4, 3.5 Hz, 1H), 4.86 (d, *J* = 7.9 Hz, 1H), 4.85 – 4.75 (m, 2H), 4.64 (d, *J* = 8.0 Hz, 1H), 4.19 – 4.15 (m, 2H), 3.97 (ddd, *J* = 7.2, 5.8, 1.2 Hz, 1H), 3.87 (dd, *J* = 11.6, 8.2 Hz, 1H), 3.71 (dd, *J* = 10.0, 5.9 Hz, 1H), 3.67 – 3.61 (m, 2H), 3.29 – 3.24 (m, 1H), 1.95 – 1.81 (m, 2H), 1.54 – 1.34 (m, 2H), 0.80 (s, 9H), -0.07 (s, 3H), -0.11 (s, 3H); ^{13}C NMR (151 MHz, $CDCl_3$) δ 165.6, 165.5, 165.44, 165.40, 165.3, 165.2, 137.8 (10), 133.5, 133.3, 133.2, 133.1, 130.1, 130.0, 129.8, 129.74, 129.68, 129.53, 129.47, 129.2, 129.0, 128.9, 128.6, 128.39, 128.35, 128.24, 128.22, 114.8, 101.41, 101.38, 74.0, 73.3, 71.9, 71.7, 70.2, 69.9, 69.1, 68.8, 68.3, 67.7, 60.5, 29.7, 28.4, 25.7, 18.1, -5.66, -5.71; ESI-HRMS m/z calculated for $[M+NH_4]^+$ $C_{65}H_{72}O_{17}SiN$ 1166.4570; found 1166.4559

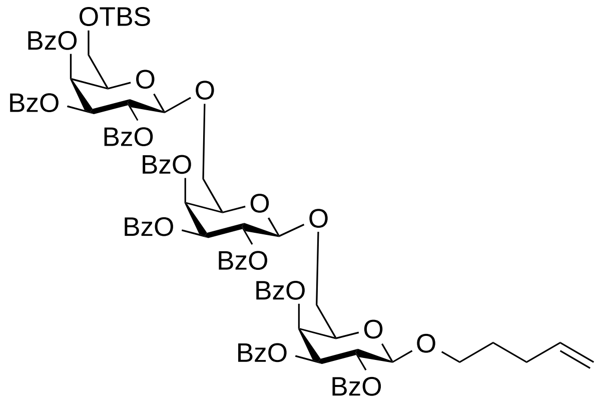

4-Pentenyl 6-O-tert-butyldimethylsilyl-2,3,4-tri-O-benzoyl-β-D-galactopyranosyl-(1→6)-2,3,4-tri-O-benzoyl-β-D-galactopyranosyl-(1→6)-2,3,4-tri-O-benzoyl-β-D-galactopyranoside **14_3c**. ^{1}H NMR (600 MHz, $CDCl_3$) δ 8.11 – 8.07 (m, 4H), 8.05 – 8.02 (m, 2H), 7.98 – 7.92 (m, 7H), 7.81 – 7.77 (m, 6H), 7.62 – 7.55 (m, 3H), 7.54 – 7.49 (m, 2H), 7.48 – 7.44 (m, 6H), 7.44 – 7.40 (m, 4H), 7.40 – 7.36 (m, 5H), 7.29 – 7.23 (m, 6H), 5.93 (dd, *J* = 3.4, 1.1 Hz, 1H), 5.89 – 5.86 (m, 2H), 5.73 – 5.67 (m, 2H), 5.64 – 5.56 (m, 2H), 5.55 – 5.51 (m, 3H), 4.84 – 4.75 (m, 2H), 4.77 (d, *J* = 7.9 Hz, 1H), 4.65 (d, *J* = 7.9 Hz, 1H), 4.60 (d, *J* = 7.8 Hz, 1H), 4.13 – 4.05 (m, 3H), 3.96 (dd, *J* = 10.0, 6.9 Hz, 1H), 3.85 (dd, *J* = 8.3, 5.6 Hz, 1H), 3.79 (dd, *J* = 10.2, 6.8 Hz, 1H), 3.70 (dt, *J* = 9.9, 5.8 Hz, 1H), 3.63 (dd, *J* = 9.9, 5.6 Hz, 1H), 3.47 (dd, *J* = 9.9, 5.5 Hz, 1H), 3.39 (dd, *J* = 9.9, 8.5 Hz, 1H), 3.35 – 3.30 (m, 1H), 1.95 – 1.83 (m, 2H), 1.57 – 1.39 (m, 2H), 0.78 (s, 9H), -0.12 (s, 3H), -0.17 (s, 3H); ^{13}C NMR (151 MHz, $CDCl_3$) δ 165.6, 165.5, 165.43, 165.36, 165.3,

165.13, 165.08, 137.8 (10), 133.34, 133.31, 133.18, 133.15, 133.12, 133.09, 133.0, 130.08, 130.07, 130.0, 129.8, 129.73, 129.69, 129.53, 129.49, 129.41, 129.35, 129.1, 129.0, 128.52, 128.51, 128.42, 128.38, 128.36, 128.23, 128.19, 114.8, 101.5, 101.00, 100.96, 73.7, 72.9, 72.6, 71.9, 71.8, 71.7, 70.1, 70.0, 69.9, 69.2, 68.6, 67.9, 67.5, 67.4, 66.5, 59.9, 29.7, 28.4, 25.7, 18.0, -5.71, -5.76; ESI-HRMS m/z calculated for $[M+Na]^+$ $C_{92}H_{90}O_{25}SiNa$ 1645.5438; found 1645.5448

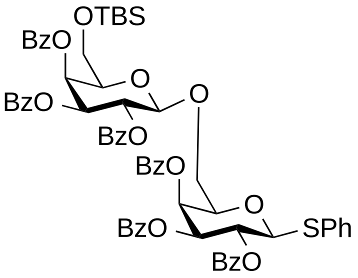

Phenyl 6-O-trimethylsilyl-2,3,4-tri-O-benzoyl-β-D-galactopyranosyl-(1→6)-2,3,4-tri-O-benzoyl-1-thio-β-D-galactopyranoside **15_2b**. ^{1}H NMR (600 MHz, $CDCl_3$) δ 8.12 – 8.09 (m, 2H), 8.00 – 7.97 (m, 5H), 7.88 – 7.85 (m, 2H), 7.84 – 7.81 (m, 2H), 7.80 – 7.76 (m, 2H), 7.65 – 7.61 (m, 2H), 7.60 – 7.57 (m, 2H), 7.57 – 7.54 (m, 1H), 7.51 – 7.47 (m, 3H), 7.47 – 7.43 (m, 5H), 7.43 – 7.37 (m, 5H), 7.28 – 7.24 (m, 4H), 5.95 (dd, *J* = 3.5, 1.1 Hz, 1H), 5.94 (dd, *J* = 3.1, 1.0 Hz, 1H), 5.76 (dd, *J* = 10.4, 7.9 Hz, 1H), 5.70 (t, *J* = 9.9 Hz, 1H), 5.56 (dd, *J* = 10.4, 3.4 Hz, 1H), 5.52 (dd, *J* = 9.9, 3.3 Hz, 1H), 4.90 (d, *J* = 4.7 Hz, 1H), 4.88 (d, *J* = 6.7 Hz, 1H), 4.23 (ddd, *J* = 6.0, 4.7, 1.1 Hz, 1H), 4.08 (dd, *J* = 10.9, 4.7 Hz, 1H), 4.00 – 3.94 (m, 2H), 3.62 (dd, *J* = 10.0, 5.8 Hz, 1H), 3.56 (dd, *J* = 10.0, 7.9 Hz, 1H), -0.02 (s, 9H); ^{13}C NMR (151 MHz, $CDCl_3$) δ 165.7, 165.5, 165.4, 165.3, 165.14, 165.11, 134.3, 133.5, 133.33, 133.26, 133.19, 133.15, 131.0, 130.01, 129.96, 129.9, 129.8, 129.47, 129.45, 129.4, 129.2, 129.0, 128.9, 128.59, 128.55, 128.5, 128.44, 128.39, 128.34, 128.25, 127.8, 101.4, 85.4, 77.11, 73.9, 73.1, 72.1, 69.9, 68.6, 67.9, 67.8, 67.7, 59.8, -0.8; ESI-HRMS m/z calculated for $[M+Na]^+$ $C_{63}H_{58}O_{16}SSiNa$ 1153.3113; found 1153.3125

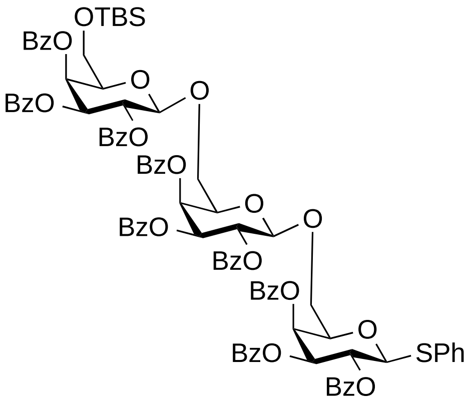

Phenyl 6-O-trimethylsilyl-2,3,4-tri-O-benzoyl-β-D-galactopyranosyl-(1→6)-2,3,4-tri-O-benzoyl-β-D-galactopyranosyl-(1→6)-2,3,4-tri-O-benzoyl-1-thio-β-D-galactopyranoside **15_3b**.

^{1}H NMR (600 MHz, $CDCl_3$) δ 8.11 – 8.07 (m, 5H), 8.00 – 7.97 (m, 2H), 7.96 – 7.93 (m, 4H), 7.86 – 7.79 (m, 7H), 7.78 – 7.75 (m, 2H), 7.61 – 7.53 (m, 5H), 7.52 – 7.46 (m, 2H), 7.46 – 7.40 (m, 10H), 7.39 – 7.34 (m, 6H), 7.28 – 7.23 (m, 7H), 5.95 (dd, *J* = 3.5, 1.1 Hz, 1H), 5.91 (dd, *J* = 3.3, 1.0 Hz, 1H), 5.89 – 5.87 (m, 1H), 5.72 – 5.67 (m, 2H), 5.67 – 5.62 (m, 1H), 5.54 – 5.49 (m, 3H), 4.90 (d, *J* = 9.9 Hz, 1H), 4.79 (d, *J* = 7.9 Hz, 1H), 4.59 (d, *J* = 7.9 Hz, 1H), 4.18 (t, *J* = 6.1 Hz, 1H), 4.06 (t, *J* = 6.3 Hz, 1H), 3.99 (dd, *J* = 10.7, 5.3 Hz, 1H), 3.92 (dd, *J* = 10.0, 7.1 Hz, 1H), 3.88 – 3.82 (m, 2H), 3.59 (dd, *J* = 10.0, 5.5 Hz, 1H), 3.40 (dd, *J* = 9.9, 5.5 Hz, 1H), 3.33 (dd, *J* = 9.9, 8.5 Hz, 1H), -0.08 (s, 9H); ^{13}C NMR (151 MHz, $CDCl_3$) δ 165.6, 165.47, 165.46, 165.4, 165.2, 165.14, 165.11, 165.09, 165.0, 134.1, 133.34, 133.32, 133.21, 133.16, 133.10, 131.06, 130.12, 130.05, 130.0, 129.9, 129.8, 129.7, 129.53, 129.45, 129.4, 129.3, 129.1, 129.03, 128.95, 128.9, 128.53, 128.46, 128.43, 128.37, 128.2, 101.09, 101.07, 85.5, 76.8, 73.7, 73.1, 72.5, 72.0, 71.8, 70.0, 69.8, 68.5, 67.9, 67.8, 67.42, 67.37, 66.6, 59.2, -0.9; ESI-HRMS m/z calculated for $[M+NH_4]^+$ $C_{90}H_{84}O_{24}SSiN$ 1622.4873; found 1622.4887

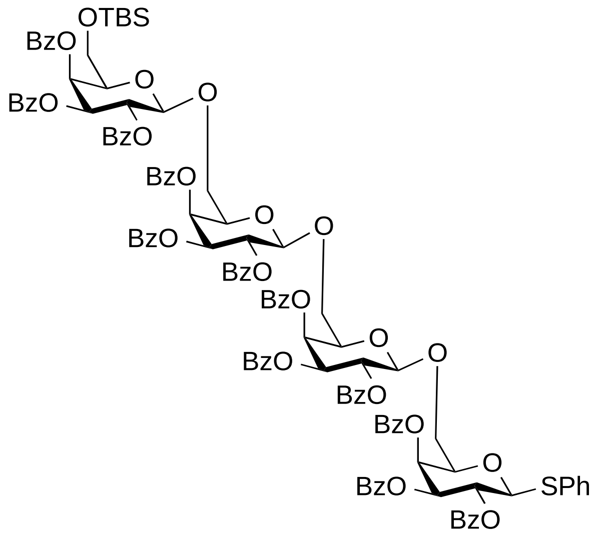

Phenyl 6-O-trimethylsilyl-2,3,4-tri-O-benzoyl-β-D-galactopyranosyl-(1→6)-2,3,4-tri-O-benzoyl-β-D-galactopyranosyl-(1→6)-2,3,4-tri-O-benzoyl-β-D-galactopyranosyl-(1→6)-2,3,4-tri-O-benzoyl-1-thio-β-D-galactopyranoside **15_4b**. ^{1}H NMR (600 MHz, $CDCl_3$) δ 8.09 – 8.05 (m, 3H), 8.05 – 8.02 (m, 2H), 7.99 – 7.95 (m, 5H), 7.95 – 7.91 (m, 5H), 7.83 – 7.80 (m, 3H), 7.80 – 7.77 (m, 3H), 7.76 – 7.73 (m, 2H), 7.56 – 7.51 (m, 6H), 7.51 – 7.43 (m, 8H), 7.43 – 7.34 (m, 16H), 7.28 – 7.22 (m, 12H), 5.93 – 5.85 (m, 4H), 5.71 – 5.57 (m, 4H), 5.53 – 5.45 (m, 4H), 4.87 (d, *J* = 9.8 Hz, 1H), 4.80 (d, *J* = 7.9 Hz, 1H), 4.52 (d, *J* = 7.8 Hz, 1H), 4.44 (d, *J* = 7.9 Hz, 1H), 4.17 (t, *J* = 6.2 Hz, 1H), 4.04 (t, *J* = 6.3 Hz, 1H), 3.99 (dd, *J* = 10.7, 5.4 Hz, 1H), 3.92 (t, *J* = 6.6 Hz, 1H), 3.88 – 3.85 (m, 1H), 3.83 (dd, *J* = 10.7, 3.5 Hz, 1H), 3.80 (d, *J* = 7.8 Hz, 1H), 3.72 (dd, *J* = 10.0, 7.6 Hz, 1H), 3.56 – 3.51 (m, 1H), 3.35 – 3.29 (m, 2H), 3.26 (dd, *J* = 10.0, 8.5 Hz, 1H), -0.08 (s, 9H); ^{13}C NMR (151 MHz, $CDCl_3$) δ 165.7, 165.6, 165.5, 165.4, 165.33, 165.26, 165.2, 165.13, 165.07, 165.04, 165.03, 165.0, 134.2, 133.4, 133.3, 133.2, 133.1, 130.2, 130.04, 130.01, 130.0, 129.9, 129.84, 129.83, 129.7, 129.4, 129.12, 129.10, 128.9, 128.6, 128.5, 128.4, 128.2, 101.1, 101.0, 100.8, 85.4, 73.7, 73.0, 72.21, 72.17, 72.1, 72.0, 71.8, 71.7, 70.1, 69.92, 69.87, 69.84, 69.82, 69.0, 68.49, 67.9, 67.74, 67.73, 67.64, 67.62, 67.4, 67.3, 66.34, 66.27, 59.2, -0.9; ESI-HRMS m/z calculated for $[M+NH_4]^+$ $C_{117}H_{106}O_{32}SSiN$ 2096.6188; found 2096.6213

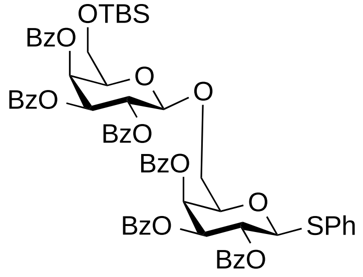

Phenyl 6-O-tert-butyldimethylsilyl-2,3,4-tri-O-benzoyl-β-D-galactopyranosyl-(1→6)-2,3,4-tri-O-benzoyl-1-thio-β-D-galactopyranoside **15_2c**. ^{1}H NMR (400 MHz, $CDCl_3$) δ 8.13 – 8.09 (m, 2H), 8.01 – 7.96 (m, 4H), 7.88 – 7.84 (m, 2H), 7.84 – 7.80 (m, 2H), 7.79 – 7.75 (m, 2H), 7.66 – 7.61 (m, 2H), 7.60 – 7.55 (m, 2H), 7.52 – 7.50 (m, 2H), 7.50 – 7.47 (m, 4H), 7.46 – 7.40 (m, 6H), 7.40 – 7.37 (m, 2H), 7.29 – 7.24 (m, 5H), 5.95 – 5.92 (m, 2H), 5.75 (dd, *J* = 10.4, 7.9 Hz, 1H), 5.69 (t, *J* = 9.9 Hz, 1H), 5.59 (dd, *J* = 10.4, 3.4 Hz, 1H), 5.51 (dd, *J* = 10.0, 3.2 Hz, 1H), 4.89 (d, *J* = 7.9 Hz, 1H), 4.86 (d, *J* = 9.8 Hz, 1H), 4.24 – 4.19 (m, 1H), 4.08 (dd, *J* = 10.9, 4.5 Hz, 1H), 4.00 – 3.90 (m, 2H), 3.69 (dd, *J* = 10.0, 5.7 Hz, 1H), 3.61 (dd, *J* = 9.9, 8.0 Hz, 1H), 0.81 (s, 9H), -0.07 (s, 3H), -0.11 (s, 3H); ^{13}C NMR (151 MHz, $CDCl_3$) δ 165.6, 165.4, 165.3, 165.12, 165.10, 134.3, 133.4, 133.31, 133.27, 133.2, 133.14, 133.10, 130.9, 129.98, 129.96, 129.83, 129.81, 129.79, 129.54, 129.49, 129.4, 129.2, 129.0, 128.9, 128.6, 128.5, 128.42, 128.38, 128.2, 101.5, 85.4, 76.9, 74.0, 73.1, 72.0, 70.0, 68.6, 68.00, 67.9, 67.7, 60.4, 25.7, 18.1, 14.2, -5.65, -5.70.

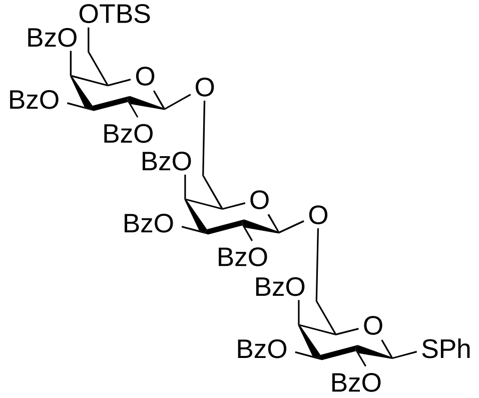

Phenyl 6-O-tert-butyldimethylsilyl-2,3,4-tri-O-benzoyl-β-D-galactopyranosyl-(1→6)-2,3,4-tri-O-benzoyl-β-D-galactopyranosyl-(1→6)-2,3,4-tri-O-benzoyl-1-thio-β-D-galactopyranoside **15_3c**. ^{1}H NMR (600 MHz, $CDCl_3$) δ 8.12 – 8.08 (m, 4H), 8.00 – 7.97 (m, 2H), 7.96 – 7.94 (m, 3H), 7.86 – 7.82 (m, 4H), 7.82 – 7.79 (m, 4H), 7.79 – 7.76 (m, 2H), 7.62 – 7.54 (m, 6H), 7.52 – 7.48 (m, 2H), 7.48 – 7.36 (m, 17H), 7.29 – 7.23 (m, 6H), 5.95 (dd, *J* = 3.4, 1.8 Hz, 1H), 5.93 – 5.91 (m, 1H), 5.88 (dd, *J* = 3.3, 1.8 Hz, 1H), 5.72 – 5.62 (m, 3H), 5.56 – 5.51 (m, 3H), 4.91 (d, *J* = 8.6 Hz, 1H), 4.79 (d, *J* = 7.8 Hz, 1H), 4.58 (d, *J* = 7.8 Hz, 1H), 4.20 – 4.13 (m, 1H), 4.09 – 4.05 (m, 1H), 4.02 – 3.97 (m, 1H), 3.98 – 3.92 (m, 1H), 3.88 – 3.81 (m, 2H), 3.64 – 3.58 (m, 1H), 3.53 – 3.46 (m, 1H), 3.44 – 3.38 (m, 1H), 0.79 (s, 9H), -0.12 (s, 3H), -0.16 (s, 3H); ^{13}C

NMR (151 MHz, $CDCl_3$) δ 165.6, 165.5, 165.4, 165.2, 165.13, 165.10, 165.0, 134.1, 133.4, 133.33, 133.31, 133.18, 133.15, 133.10, 133.06, 131.1, 130.1, 130.03, 129.99, 129.98, 129.9, 129.84, 129.82, 129.7, 129.53, 129.50, 129.43, 129.40, 129.3, 129.1, 129.0, 128.9, 128.54, 128.45, 128.42, 128.37, 128.22, 128.20, 101.2, 101.1, 85.5, 77.26, 73.75,, 73.1, 72.6, 71.9, 71.8, 70.1, 69.8, 68.5, 67.9, 67.5, 67.3, 66.8, 60.4, 59.9, 25.7, 21.1, 18.0, -5.71, -5.75; ESI-HRMS m/z calculated for $[M+Na]^+$ $C_{93}H_{86}O_{24}SSiNa$ 1669.4897; found 1669.4906

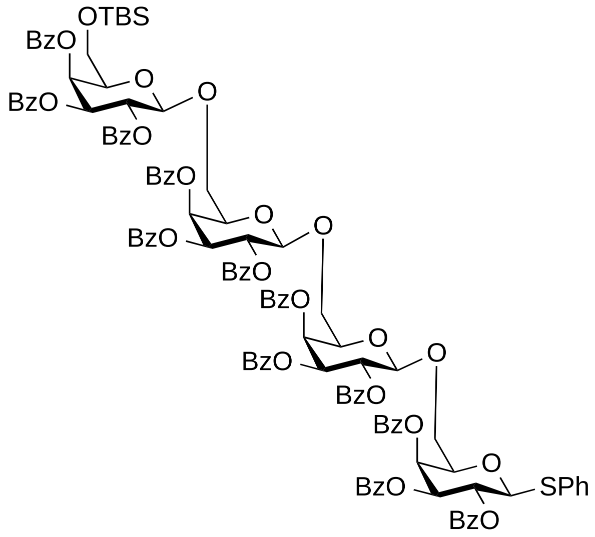

Phenyl 6-O-tert-butyldimethylsilyl-2,3,4-tri-O-benzoyl-β-D-galactopyranosyl-(1→6)-2,3,4-tri-O-benzoyl-β-D-galactopyranosyl-(1→6)-2,3,4-tri-O-benzoyl-β-D-galactopyranosyl-(1→6)-2,3,4-tri-O-benzoyl-1-thio-β-D-galactopyranoside **15_4c**. ^{1}H NMR (600 MHz, $CDCl_3$) δ 8.10 – 8.08 (m, 4H), 8.06 – 8.01 (m, 4H), 7.99 – 7.92 (m, 8H), 7.85 – 7.78 (m, 12H), 7.77 – 7.74 (m, 2H), 7.57 – 7.53 (m, 5H), 7.52 – 7.43 (m, 7H), 7.42 – 7.35 (m, 14H), 7.33 – 7.30 (m, 1H), 7.29 – 7.20 (m, 8H), 5.93 (dd, J = 3.4, 1.1 Hz, 1H), 5.91 – 5.88 (m, 2H), 5.86 (dd, J = 3.4, 1.1 Hz, 1H), 5.72 – 5.68 (m, 1H), 5.68 – 5.63 (m, 1H), 5.63 – 5.58 (m, 2H), 5.54 – 5.46 (m, 4H), 4.89 (d, J = 9.9 Hz, 1H), 4.79 (d, J = 7.9 Hz, 1H), 4.52 (d, J = 7.9 Hz, 1H), 4.44 (d, J = 7.9 Hz, 1H), 4.19 – 4.17 (m, 1H), 4.10 – 4.05 (m, 1H), 4.05 – 4.01 (m, 1H), 4.01 – 3.98 (m, 1H), 3.94 – 3.90 (m, 1H), 3.90 – 3.83 (m, 2H), 3.83 – 3.78 (m, 1H), 3.75 (dd, J = 9.8, 7.4 Hz, 1H), 3.53 (dd, J = 9.8, 5.6 Hz, 1H), 3.40 (dd, J = 9.9, 5.4 Hz, 1H), 3.36 – 3.29 (m, 2H), 0.79 (s, 9H), -0.13 (s, 3H), -0.18 (s, 3H); ^{13}C NMR (151 MHz, $CDCl_3$) δ 165.7, 165.6, 165.5, 165.43, 165.38, 165.35, 165.3, 165.2, 165.13, 165.06, 165.05, 165.02, 164.99, 134.2, 133.5, 133.42, 133.39, 133.34, 133.30, 133.22, 133.17, 133.14, 133.10, 133.05, 131.0, 130.21, 130.16, 130.15, 130.1, 130.0, 129.87, 129.85, 129.83, 129.79, 129.76, 129.73, 129.65, 129.6, 129.52, 129.45, 129.44, 129.40, 129.23, 129.20, 129.15, 129.13, 129.06, 129.0, 128.91, 128.87, 128.8, 128.7, 128.62, 128.59, 128.53, 128.50, 128.42, 128.38, 128.36, 128.3, 128.22, 128.20, 128.17, 101.1, 101.0, 100.8, 85.5, 76.9, 73.7, 73.0, 72.3, 72.2, 71.9, 71.8, 71.7, 70.1, 70.0, 69.8, 69.0, 68.5, 67.9, 67.8, 67.7, 67.4, 67.3, 66.4, 66.2, 59.8, 29.7, 25.8, 25.7, 25.3, 18.0, 14.2, -5.72, -5.76; ESI-HRMS m/z calculated for $[M+Na]^+$ $C_{120}H_{108}O_{32}SSiNa$ 2143.6211; found 2143.6212

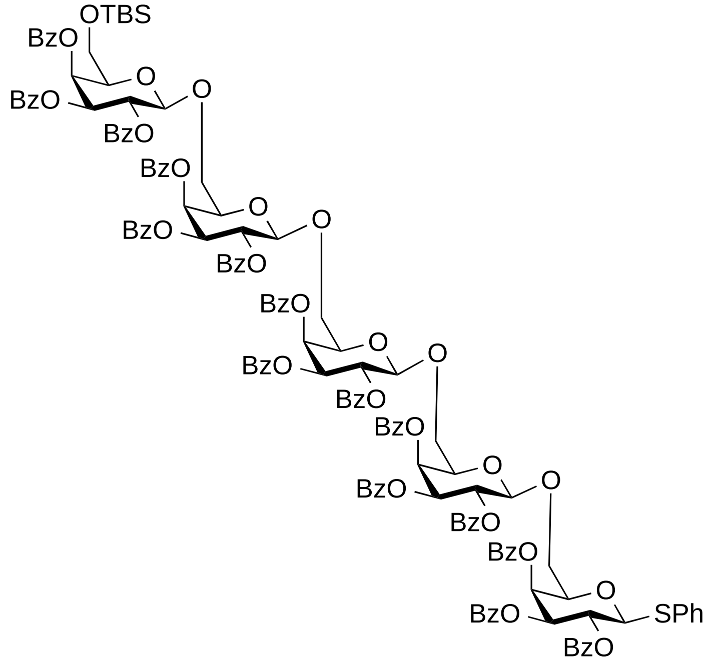

Phenyl 6-O-tert-butyldimethylsilyl-2,3,4-tri-O-benzoyl-β-D-galactopyranosyl-(1→6)-2,3,4-tri-O-benzoyl-β-D-galactopyranosyl-(1→6)-2,3,4-tri-O-benzoyl-β-D-galactopyranosyl-(1→6)-2,3,4-tri-O-benzoyl-β-D-galactopyranosyl-(1→6)-2,3,4-tri-O-benzoyl-1-thio-β-D-galactopyranoside **15_5c**. ^{1}H NMR (600 MHz, $CDCl_3$) δ 8.09 – 8.06 (m, 4H), 8.05 – 8.02 (m, 2H), 8.00 (dd, J = 8.3, 1.4 Hz, 2H), 7.99 – 7.94 (m, 6H), 7.94 – 7.91 (m, 5H), 7.89 – 7.86 (m, 2H), 7.83 – 7.78 (m, 13H), 7.76 – 7.74 (m, 2H), 7.71 – 7.68 (m, 2H), 7.57 – 7.50 (m, 5H), 7.50 – 7.40 (m, 12H), 7.38 – 7.32 (m, 13H), 7.33 – 7.30 (m, 2H), 7.28 – 7.21 (m, 10H), 6.01 (d, J = 3.3 Hz, 1H), 5.90 (dd, J = 3.4, 1.2 Hz, 1H), 5.89 – 5.86 (m, 3H), 5.85, 5.69 – 5.63 (m, 2H), 5.61 – 5.54 (m, 3H), 5.52 – 5.48 (m, 3H), 5.48 – 5.43 (m, 2H), 4.87 (d, J = 9.9 Hz, 1H), 4.77 (d, J = 7.9 Hz, 1H), 4.51 (d, J = 7.9 Hz, 1H), 4.41 (d, J = 7.8 Hz, 1H), 4.39 (d, J = 7.7 Hz, 1H), 4.30 – 4.23 (m, 2H), 4.07 (dd, J = 11.4, 3.7 Hz, 1H), 4.02 (t, J = 6.7 Hz, 1H), 3.97 (dd, J = 10.7, 5.4 Hz, 1H), 3.90 – 3.86 (m, 2H), 3.86 – 3.81 (m, 2H), 3.78 (dd, J = 9.3, 5.4 Hz, 1H), 3.68 – 3.62 (m, 2H), 3.55 – 3.49 (m, 1H), 3.36 (dd, J = 9.9, 5.4 Hz, 1H), 3.31 – 3.24 (m, 2H), 3.21 (dd, J = 9.7, 5.0 Hz, 1H), 0.78 (s, 9H), -0.14 (s, 3H), -0.19 (s, 3H); ^{13}C NMR (151 MHz, $CDCl_3$) δ 165.60, 165.56, 165.47, 165.45, 165.41, 165.38, 165.34, 165.33, 165.25, 165.2, 165.1, 165.03, 165.00, 164.9, 134.1, 133.5, 133.33, 133.30, 133.2, 133.1, 131.0, 130.20, 130.15, 130.10, 130.05, 129.94, 129.86, 129.82, 129.79, 129.74, 129.71, 129.51, 129.50, 129.46, 129.42, 129.24, 129.19, 129.14, 129.06, 129.0, 128.9, 128.8, 128.5, 128.41, 128.37, 128.20, 128.16, 101.2, 101.0, 100.9, 100.7, 85.5, 74.8, 73.6, 73.0, 72.3, 72.10, 72.06, 71.93, 71.91, 71.8, 71.7, 70.1, 70.0, 69.9, 69.8, 68.48, 68.46, 68.3, 67.9, 67.8, 67.5, 67.34, 66.29, 66.1, 66.0, 65.9, 60.4, 59.6, 25.7, 18.0, -5.74, -5.77; ESI-HRMS m/z calculated for $[M+Na]^+$ $C_{147}H_{130}O_{40}SSiNa$ 2617.7526; found 2617.7558

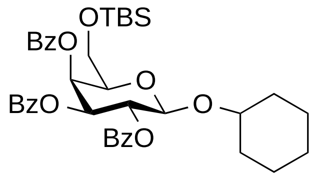

Cyclohexyl 6-O-tert-butyldimethylsilyl-2,3,4-tri-O-benzoyl-β-D-galactopyranoside **16_1c**. ^{1}H NMR (600 MHz, $CDCl_3$) δ 8.13 – 8.09 (m, 2H), 8.00 – 7.97 (m, 2H), 7.83 – 7.80 (m, 2H), 7.65 – 7.61 (m, 1H), 7.55 – 7.48 (m, 3H), 7.46 – 7.42 (m, 1H), 7.42 – 7.38 (m, 2H), 7.28 – 7.24 (m, 2H), 5.95 (dd, *J* = 3.6, 1.2 Hz, 1H), 5.74 (dd, *J* = 10.4, 7.9 Hz, 1H), 5.60 (dd, *J* = 10.4, 3.5 Hz, 1H), 4.88 (d, *J* = 7.9 Hz, 1H), 4.02 (ddd, *J* = 7.4, 6.2, 1.3 Hz, 1H), 3.89 – 3.79 (m, 2H), 3.77 – 3.69 (m, 1H), 1.98 (d, *J* = 13.1 Hz, 1H), 1.83 – 1.71 (m, 2H), 1.65 – 1.59 (m, 1H), 1.54 – 1.45 (m, 2H), 1.33 – 1.23 (m, 2H), 1.22 – 1.11 (m, 2H), 0.87 (s, 9H), 0.02 (s, 3H), -0.02 (s, 3H); ^{13}C NMR (151 MHz, $CDCl_3$) δ 165.7, 165.6, 165.2, 133.3, 133.1, 133.02, 129.99, 129.82, 129.75, 129.64, 129.56, 129.1, 128.5, 128.3, 128.2, 100.2, 78.3, 74.2, 72.2, 70.3, 68.1, 61.2, 33.4, 31.7, 25.8, 25.5, 23.9, 23.7, -5.59, -5.61; ESI-HRMS m/z calculated for $[M+Na]^+$ $C_{39}H_{48}O_9SiNa$ 711.2965; found 711.2968

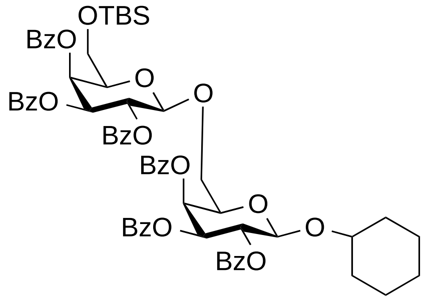

Cyclohexyl 6-O-tert-butyldimethylsilyl-2,3,4-tri-O-benzoyl-β-D-galactopyranosyl-(1→6)-2,3,4-tri-O-benzoyl-β-D-galactopyranoside **16_2c**. ^{1}H NMR (600 MHz, $CDCl_3$) δ 8.12 – 8.09 (m, 4H), 7.99 – 7.95 (m, 4H), 7.82 – 7.78 (m, 4H), 7.65 – 7.61 (m, 2H), 7.55 – 7.51 (m, 2H), 7.51 – 7.47 (m, 4H), 7.47 – 7.42 (m, 2H), 7.41 – 7.38 (m, 4H), 7.29 – 7.24 (m, 4H), 5.92 (d, *J* = 1.2 Hz, 1H), 5.92 (d, *J* = 1.1 Hz, 1H), 5.76 – 5.71 (m, 1H), 5.74 – 5.69 (m, 1H), 5.56 (dd, *J* = 10.4, 3.5 Hz, 1H), 5.53 (dd, *J* = 10.4, 3.5 Hz, 1H), 4.89 (d, *J* = 7.9 Hz, 1H), 4.79 (d, *J* = 8.0 Hz, 1H), 4.19 – 4.14 (m, 1H), 4.13 (dd, *J* = 10.5, 4.5 Hz, 1H), 3.99 – 3.90 (m, 2H), 3.69 (dd, *J* = 10.0, 5.7 Hz, 1H), 3.64 – 3.57 (m, 2H), 1.79 – 1.73 (m, 1H), 1.66 – 1.59 (m, 2H), 1.56 – 1.49 (m, 2H), 1.44 – 1.34 (m, 2H), 1.29 – 1.21 (m, 2H), 1.14 – 1.07 (m, 2H), 0.80 (s, 9H), -0.07 (s, 3H), -0.11 (s, 3H); ^{13}C NMR (151 MHz, $CDCl_3$) δ 165.6, 165.52, 165.50, 165.4, 165.2, 133.4, 133.3, 133.2, 133.13, 133.08, 133.06, 130.1, 130.0, 129.8, 129.73, 129.65, 129.62, 129.56, 129.50, 129.3, 129.0, 128.6, 128.5, 128.4, 128.3, 128.2, 101.1, 99.9, 73.9, 73.4, 72.0, 71.9, 70.2, 70.0, 68.8, 67.8, 67.7, 60.4, 33.1, 31.3, 25.7, 25.4, 23.5, 23.3, 18.1, -5.66, -5.71; ESI-HRMS m/z calculated for $[M+Na]^+$ $C_{66}H_{70}O_{17}SiNa$ 1185.4280; found 1185.4280

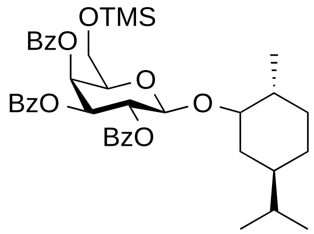

Menthyl 6-O-trimethylsilyl-2,3,4-tri-O-benzoyl-β-D-galactopyranoside **17_1b**. ^{1}H NMR (600 MHz, $CDCl_3$) δ 8.12 – 8.09 (m, 2H), 7.99 – 7.96 (m, 2H), 7.81 – 7.78 (m, 2H), 7.65 – 7.60 (m, 1H), 7.54 – 7.47 (m, 3H), 7.46 – 7.42 (m, 1H), 7.41 – 7.37 (m, 2H), 7.27 – 7.23 (m, 2H), 5.94 (dd, *J* = 3.5, 1.2 Hz, 1H), 5.80 (dd, *J* = 10.4, 7.9 Hz, 1H), 5.58 (dd, *J* = 10.4, 3.5 Hz, 1H), 4.82 (d, *J* = 7.9 Hz, 1H), 4.07 – 4.01 (m, 1H), 3.84 – 3.74 (m, 2H), 3.38 (td, *J* = 10.6, 4.4 Hz, 1H), 2.29 (dd, *J* = 12.7, 5.3 Hz, 1H), 2.01 – 1.89 (m, 1H), 1.66 – 1.61 (m, 1H), 1.58 – 1.52 (m, 2H), 1.44 – 1.35 (m, 2H), 1.25 – 1.14 (m, 1H), 0.95 (d, *J* = 6.5 Hz, 3H), 0.93 – 0.78 (m, 3H), 0.50 (d, *J* = 7.0 Hz, 3H), 0.39 (d, *J* = 6.9 Hz, 1H), 0.06 (s, 9H); ^{13}C NMR (151 MHz, $CDCl_3$) δ 165.70, 165.68, 165.3, 133.3, 133.1, 133.04, 130.00, 129.8, 129.7, 128.5, 128.23, 128.20, 103.2, 83.3, 77.24, 77.19, 77.0, 76.9, 76.8, 74.1, 72.2, 70.1, 68.2, 60.7, 48.0, 43.2, 34.2, 31.7, 29.7, 24.7, 22.7, 22.3, 20.7, 15.4, -0.7; ESI-HRMS m/z calculated for $[M\text{-}Na]^+$ $C_{40}H_{50}O_9SiNa$ 725.3121; found 725.3124

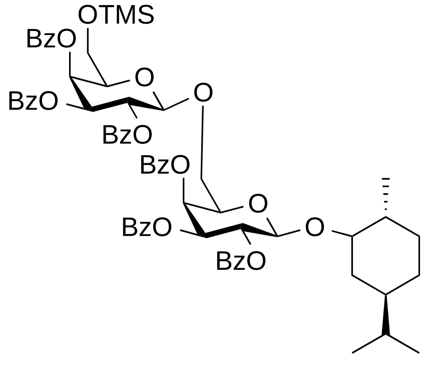

Menthyl 6-O-trimethylsilyl-2,3,4-tri-O-benzoyl-β-D-galactopyranosyl-(1→6)-2,3,4-tri-O-benzoyl-β-D-galactopyranoside **17_2b**. ^{1}H NMR (600 MHz, $CDCl_3$) δ 8.10 – 8.08 (m, 4H), 8.00 – 7.94 (m, 6H), 7.83 – 7.77 (m, 4H), 7.63 – 7.59 (m, 1H), 7.54 – 7.50 (m, 2H), 7.49 – 7.44 (m, 5H), 7.42 – 7.37 (m, 3H), 7.28 – 7.23 (m, 5H), 6.00 – 5.96 (m, 1H), 5.93 – 5.88 (m, 1H), 5.76 (dd, *J* = 10.5, 7.9 Hz, 1H), 5.70 (dd, *J* = 10.4, 7.8 Hz, 1H), 5.57 (dd, *J* = 10.5, 3.5 Hz, 1H), 5.53 (dd, *J* = 10.4, 3.3 Hz, 1H), 4.88 – 4.84 (m, 1H), 4.79 – 4.75 (m, 1H), 4.16 (d, *J* = 6.2 Hz, 1H), 4.10 (t, *J* = 8.3 Hz, 1H), 3.98 (dd, *J* = 10.1, 5.7 Hz, 1H), 3.93 (t, *J* = 7.3 Hz, 1H), 3.49 (dd, *J* = 9.9, 5.4 Hz, 1H), 3.42 (t, *J* = 9.2 Hz, 1H), 3.32 (s, 1H), 2.26 (d, *J* = 12.7 Hz, 1H), 1.91 (s, 2H), 1.52 (s, 1H), 1.21 (dd, *J* = 23.0, 11.2 Hz, 2H), 0.93 (d, *J* = 6.3 Hz, 4H), 0.91 – 0.74 (m, 4H), 0.46 (dd, *J* = 7.3, 2.3 Hz, 2H), 0.42 – 0.36 (m, 2H), -0.06 (s, 9H); ^{13}C NMR (151 MHz, $CDCl_3$) δ 165.6, 165.5, 165.3, 165.2, 133.5, 133.43, 133.38, 133.31, 133.25, 133.16, 130.07, 130.03, 129.84, 129.79, 129.74, 129.70, 128.5, 128.4, 128.2, 103.1, 101.1, 83.2, 73.8, 73.3, 72.9, 72.1, 71.8, 70.2, 70.0, 68.3, 67.5, 67.0, 59.5, 48.1, 43.2, 34.1, 31.7, 29.7, 24.6, 22.7, 22.4, 20.6, 15.5, -0.9; ESI-HRMS m/z calculated for $[M\text{-}NH_4]^+$ $C_{67}H_{76}O_{17}SiN$ 1194.4883; found 1194.4846

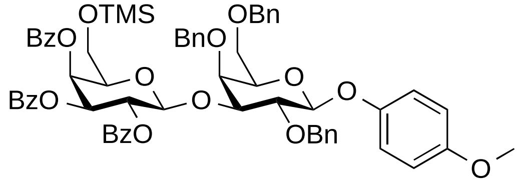

***4-Methoxyphenyl 6-O-trimethylsilyl-2,3,4-tri-O-benzoyl-β-D-galactopyranosyl-(1→3)-2,4,6-tri-O-benzyl-β-D-galactopyranoside* $\mathbf{18_1b}$**. ^{1}H NMR (600 MHz, $CDCl_3$) δ 8.18 – 8.15 (m, 2H), 8.13 – 8.10 (m, 2H), 8.10 – 8.07 (m, 2H), 8.02 – 7.99 (m, 1H), 7.99 – 7.95 (m, 1H), 7.86 – 7.83 (m, 2H), 7.68 – 7.63 (m, 2H), 7.55 – 7.51 (m, 5H), 7.41 – 7.38 (m, 5H), 7.37 – 7.33 (m, 4H), 7.34 – 7.29 (m, 4H), 7.03 – 7.01 (m, 2H), 6.81 – 6.78 (m, 2H), 5.86 – 5.83 (m, 1H), 5.76 – 5.73 (m, 2H), 5.29 – 5.27 (m, 1H), 5.03 (d, *J* = 11.4 Hz, 2H), 4.86 (d, *J* = 7.7 Hz, 1H), 4.84 – 4.78 (m, 2H), 4.67 (d, *J* = 7.4 Hz, 1H), 4.64 (d, *J* = 11.7 Hz, 1H), 4.48 (d, *J* = 11.8 Hz, 1H), 4.43 (d, *J* = 11.7 Hz, 1H), 4.15 (q, *J* = 7.1 Hz, 1H), 3.94 (dd, *J* = 9.3, 7.5 Hz, 2H), 3.81 (dd, *J* = 9.4, 3.1 Hz, 1H), 3.79 – 3.77 (m, 4H), 3.73 (dd, *J* = 3.1, 1.1 Hz, 1H), 3.71 – 3.68 (m, 1H), 3.68 – 3.64 (m, 1H), 3.62 (dd, *J* = 9.4, 6.4 Hz, 1H), 0.18 (s, 9H); ^{13}C NMR (151 MHz, $CDCl_3$) δ 165.0, 155.0, 151.7, 138.8, 138.7, 138.0, 133.7, 133.6, 133.5, 130.1, 130.00, 129.96, 129.7, 128.9, 128.7, 128.6, 128.5, 128.4, 128.3, 128.2, 127.9, 127.8, 127.6, 127.5, 118.4, 114.4, 103.1, 99.3, 79.5, 77.3, 77.1, 76.8, 76.8, 75.4, 75.3, 75.1, 73.7, 73.6, 72.3, 71.7, 69.1, 68.0, 65.9, 64.9, 55.6, 0.4; ESI-HRMS m/z calculated for $[M+Na]^+$ $C_{64}H_{66}O_{15}SiNa$ 1125.4068; found 1125.4059

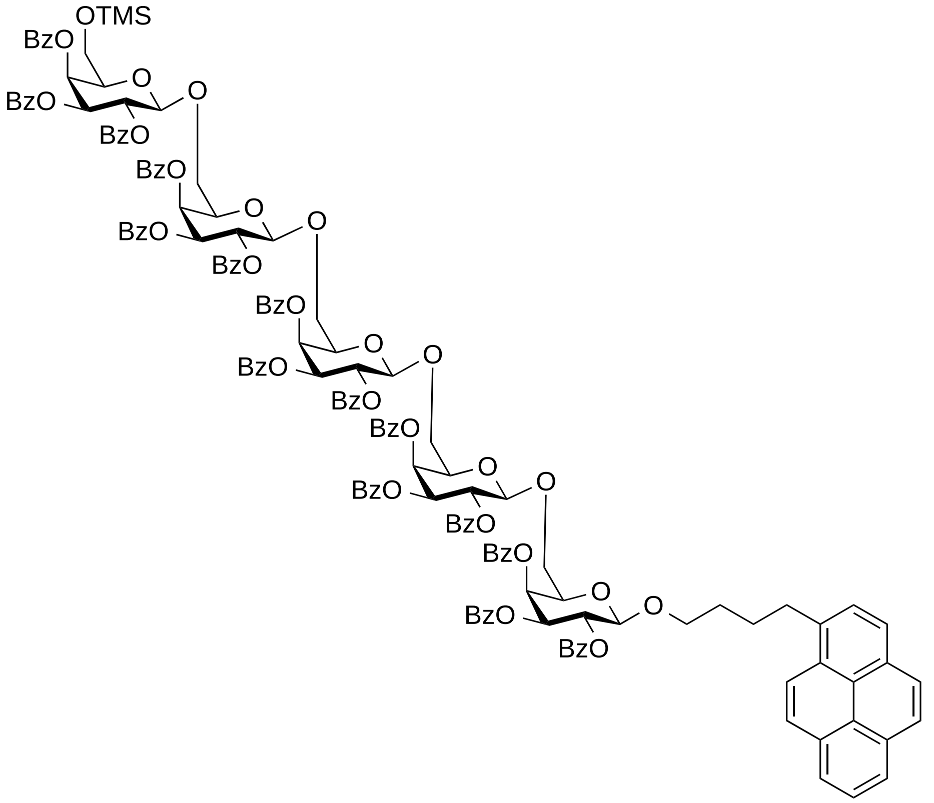

4-(Pyren-1-yl)butyl 6-O-trimethylsilyl-2,3,4-tri-O-benzoyl-β-D-galactopyranosyl-(1→6)-2,3,4-tri-O-benzoyl-β-D-galactopyranosyl-(1→6)-2,3,4-tri-O-benzoyl-β-D-galactopyranosyl-(1→6)-2,3,4-tri-O-benzoyl-β-D-galactopyranosyl-(1→6)-2,3,4-tri-O-benzoyl-β-D-galactopyranoside **4_5b**. ^{1}H NMR (600 MHz, $CDCl_3$) δ 8.08 – 7.99 (m, 13H), 7.99 – 7.90 (m, 12H), 7.81 – 7.74 (m, 9H), 7.55 – 7.49 (m, 3H), 7.49 – 7.40 (m, 13H), 7.41 – 7.31 (m, 17H), 7.27 – 7.22 (m, 14H), 7.07 – 7.03 (m, 3H), 5.86 (m, 6H), 5.80 (t, *J* = 3.3 Hz, 1H), 5.71 – 5.62 (m, 4H), 5.61 – 5.53 (m, 4H), 5.52 – 5.42 (m, 5H), 4.71 (d, *J* = 7.9 Hz, 1H), 4.61 (d, *J* = 8.0 Hz, 1H), 4.53 – 4.51 (m, 1H), 4.42 (d, *J* = 7.8 Hz, 1H), 4.37 (d, *J* = 7.8 Hz, 1H), 4.11 – 3.96 (m, 4H), 3.95 – 3.82 (m, 2H), 3.82 – 3.68 (m, 4H), 3.67 – 3.58 (m, 2H), 3.32 – 3.19 (m, 6H), 3.18 – 3.09 (m, 3H), -0.09 (s, 9H); ESI-HRMS m/z calculated for $[M+NH_4]^+$ $C_{161}H_{146}O_{41}SiN$ 2776.9140; found 2776.9135

References

(1) Pangborn, A. B.; Giardello, M. A.; Grubbs, R. H.; Rosen, R. K.; Timmers, F. J. *Organometallics* **1996**, *15*, 1518–1520.
(2) Fulmer, G. R.; Miller, A. J. M.; Sherden, N. H.; Gottlieb, H. E.; Nudelman, A.; Stoltz, B. M.; Bercaw, J. E.; Goldberg, K. I. *Organometallics* **2010**, *29*, 2176–2179.
(3) Kovac, P.; Glaudemans, C. P.; Guo, W.; Wong, T. C. *Carbohyd Res* **1985**, *140*, 299–311.
(4) Excoffier, G.; Gagnaire, D.; Utille, J. P. *Carbohyd Res* **1975**, *39*, 368–373.
(5) Posner, G. H.; Haines, S. R. *Tetrahedron Lett* **1985**, *26*, 5–8.
(6) Kovac, P.; Glaudemans, C. P. *Carbohyd Res* **1985**, *140*, 313–318.
(7) Gung, B. W.; Fox, R. M. *Tetrahedron* **2004**, *60*, 9405–9415.
(8) Pertici, F.; Pieters, R. J. *Chem Commun* **2012**, *48*, 4008–4010.
(9) Pattenden, G.; Ashweek, N. J.; Baker-Glenn, C. A. G.; Walker, G. M.; Yee, J. G. K. *Angew. Chem. Int. Ed.* **2007**, *46*, 4359–4363.
(10) Nashed, E. M.; Glaudemans, C. P. *J Org Chem* **1987**, *52*, 5255–5260.
(11) Keliris, A.; Ziegler, T.; Mishra, R.; Pohmann, R.; Sauer, M. G.; Ugurbil, K.; Engelmann, J. *Bioorg Med Chem* **2011**, *19*, 2529–2540.
(12) Hada, N.; Oka, J.; Nishiyama, A.; Takeda, T. *Tetrahedron Lett* **2006**, *47*, 6647–6650.
(13) Bundy, G. L.; Peterson, D. C. *Tetrahedron Lett* **1978**, *19*, 41–44.
(14) Sweeley, C. C.; Bentley, R.; Makita, M.; Wells, W. W. *J Am Chem Soc* **1963**, *85*, 2497–2507.
(15) Mai, K.; Patil, G. *J Org Chem* **1986**, *51*, 3545–3548.
(16) Corey, E. J.; Venkateswarlu, A. *J Am Chem Soc* **1972**, *94*, 6190–6191.
