# Supplementary material for: Galactan synthesis in a single step via oligomerization of monosaccharides
Source: Beilstein J Org Chem. 2014 Nov 13;10:2658–63. doi: 10.3762/bjoc.10.279 (PMC4273286; doi:10.3762/bjoc.10.279)

Supporting Information File 2

for

Galactan synthesis in a single step via oligomerization of monosaccharides

Marius Dräger and Amit Basu*

Address: Department of Chemistry, Box H, Brown University, Providence, RI 02912, USA

Email: Amit Basu - abasu@brown.edu

*Corresponding author

NMR spectra for all new compounds

Table of Contents

NMR spectra.. 5
Compound **1**.. 5
Proton.. 5
13C.. 6
HSQC.. 7
Compound **2**.. 8
Proton.. 8
Carbon.. 9
COSY.. 10
HSQC.. 11
19F.. 12
Compound **7**.. 13
Proton.. 13
Carbon.. 14
COSY.. 15
HSQC.. 16
Compound **8**.. 17
Proton.. 17
Carbon.. 18
COSY.. 19
Compound **12**.. 20
Proton.. 20
Carbon.. 21
Compound **21**.. 22
Proton.. 22
Carbon.. 23
Compound **23**.. 24
Proton.. 24
Compound $\mathbf{4_1a}$.. 25
Proton.. 25
Carbon.. 26
COSY.. 27
HSQC.. 28
Compound $\mathbf{4_2a}$.. 29
Proton.. 29
Carbon.. 30
COSY.. 31
HSQC.. 32
Compound $\mathbf{4_3a}$.. 33
Proton.. 33
COSY.. 34
Compound $\mathbf{4_1b}$.. 35
Proton.. 35
Carbon.. 36
COSY.. 37
HSQC.. 38
Compound $\mathbf{4_2b}$.. 39
Proton.. 39
Carbon.. 40
COSY.. 41
HSQC.. 42
Compound $\mathbf{4_3b}$.. 43
Proton.. 43
Carbon.. 44
COSY.. 45
HSQC.. 46

Compound **4_4b** ... 47
Proton ... 47
Carbon ... 48
COSY ... 49
HSQC ... 50
Compound **4_1c** ... 51
Proton ... 51
Carbon ... 52
Compound **4_2c** ... 53
Proton ... 53
Carbon ... 54
COSY ... 55
HSQC ... 56
Compound **4_3c** ... 57
Proton ... 57
Carbon ... 58
COSY ... 59
HSQC ... 60
Compound **4_4c** ... 61
Proton ... 61
Carbon ... 62
COSY ... 63
HSQC ... 64
Compound **4_5c** ... 65
Proton ... 65
Compound **13_1b** ... 66
Proton ... 66
Carbon ... 67
COSY ... 68
HSQC ... 69
Compound **13_2b** ... 70
Proton ... 70
Carbon ... 71
COSY ... 72
HSQC ... 73
Compound **13_3b** ... 74
Proton ... 74
Carbon ... 75
COSY ... 76
HSQC ... 77
Compound **13_4b** ... 78
Proton ... 78
Compound **14_1c** ... 79
Proton ... 79
Carbon ... 80
COSY ... 81
HSQC ... 82
Compound **14_2c** ... 83
Proton ... 83
Carbon ... 84
COSY ... 85
HSQC ... 86
Compound **14_3c** ... 87
Proton ... 87
Carbon ... 88
COSY ... 89
HSQC ... 90
Compound **15_2b** ... 91

Proton 91
Carbon 92
COSY 93
HSQC 94
Compound **15_3b** 95
Proton 95
Carbon 96
COSY 97
HSQC 98
Compound **15_4b** 99
Proton 99
Carbon 100
COSY 101
HSQC 102
Compound **15_2c** 103
Proton 103
Carbon 104
COSY 105
HSQC 106
Compound **15_3c** 107
Proton 107
Carbon 108
COSY 109
HSQC 110
Compound **15_4c** 111
Proton 111
Carbon 112
COSY 113
HSQC 114
Compound **15_5c** 115
Proton 115
Carbon 116
Compound **16_1c** 117
Proton 117
Carbon 118
COSY 119
HSQC 120
Compound **16_2c** 121
Proton 121
Carbon 122
COSY 123
HSQC 124
Compound **17_1b** 125
Proton 125
Carbon 126
COSY 127
HSQC 128
Compound **17_2b** 129
Proton 129
Carbon 130
COSY 131
Compound **18_1b** 132
Proton 132
Carbon 133
Compound **4_5b** 134
Proton 134

NMR spectra

<u>Compound **1**</u>

Proton

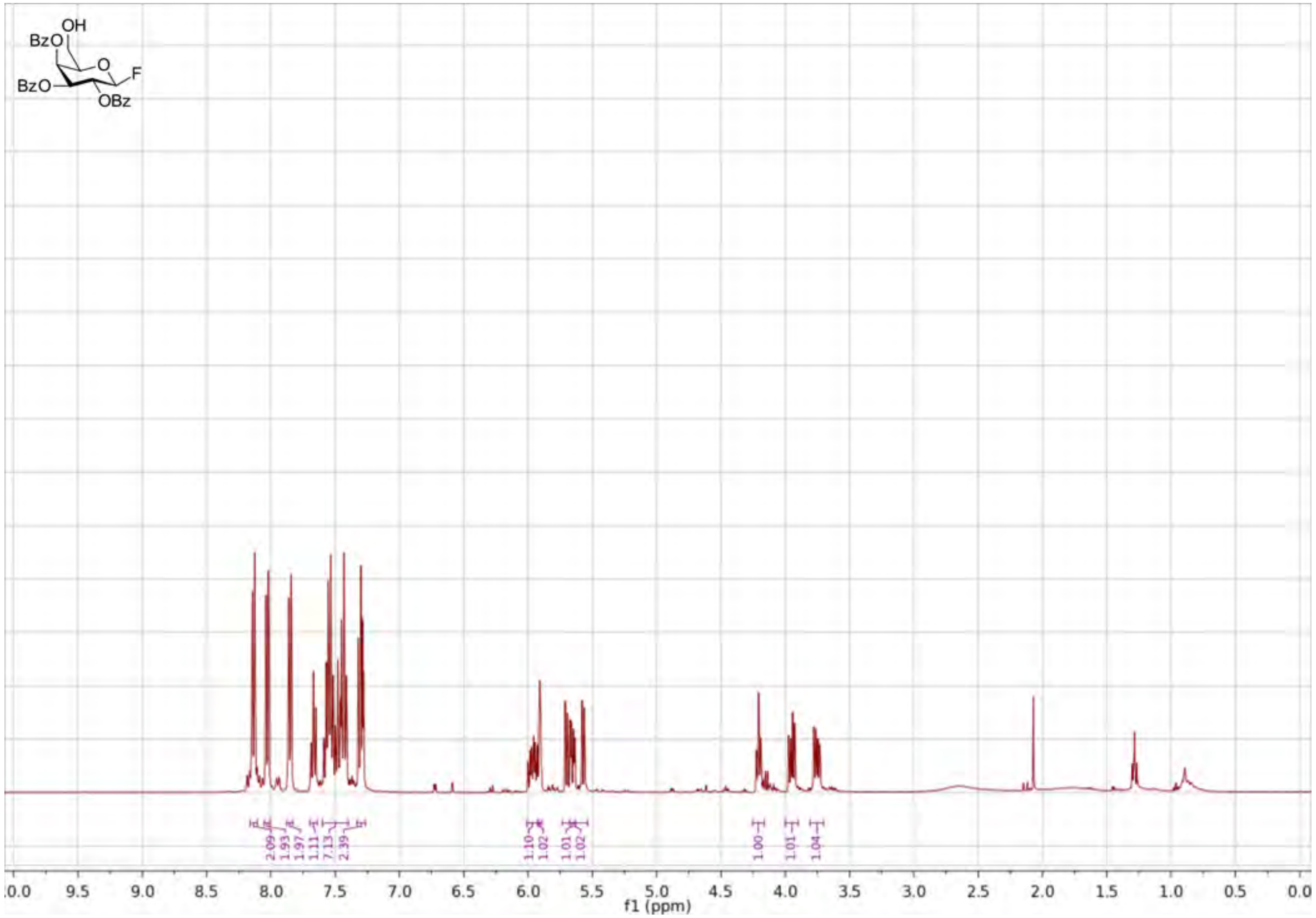

13C

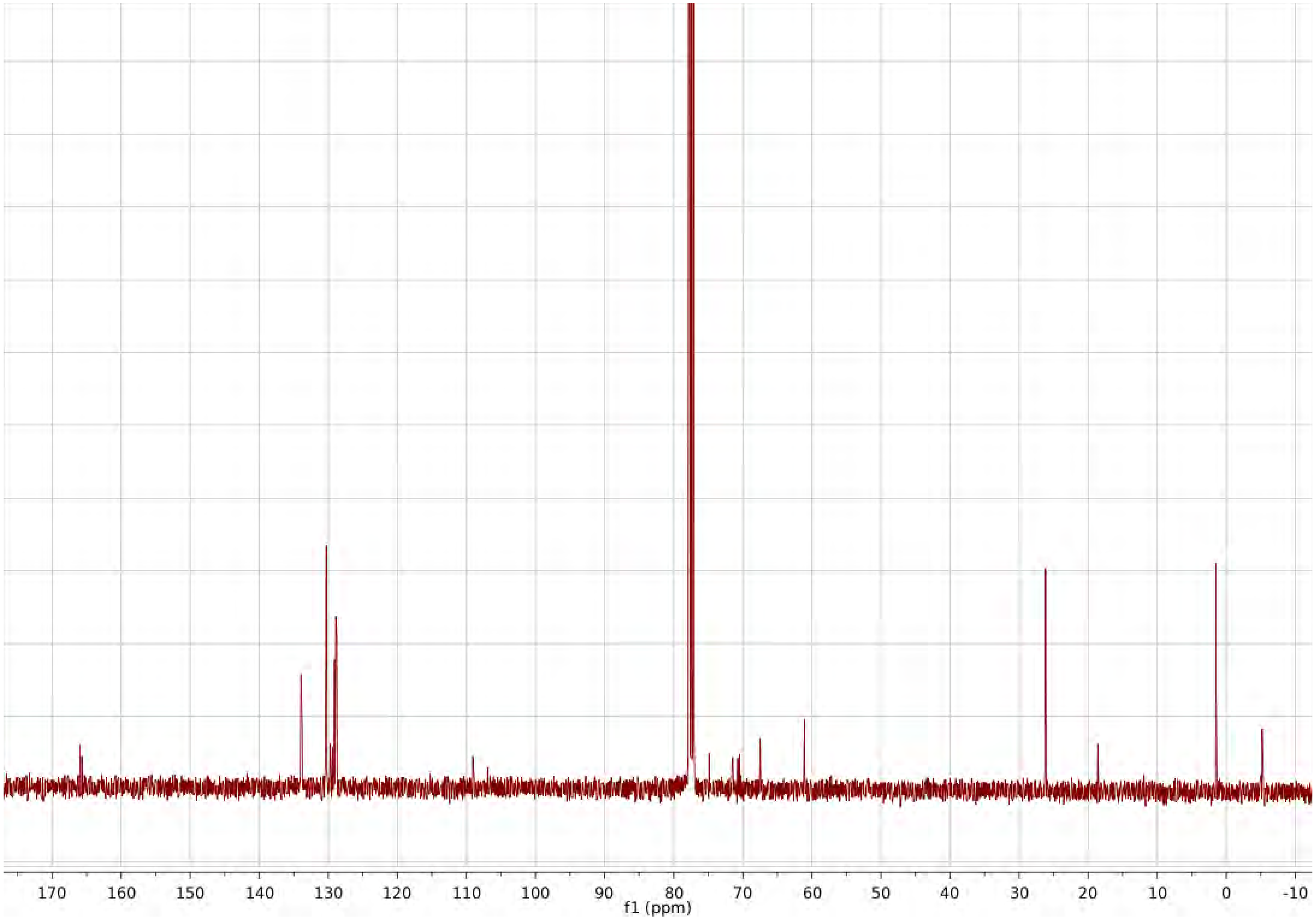

170
160
150
140
130
120
110
100
90
80
70
60
50
40
30
20
10
0
-10
f1 (ppm)

HSQC

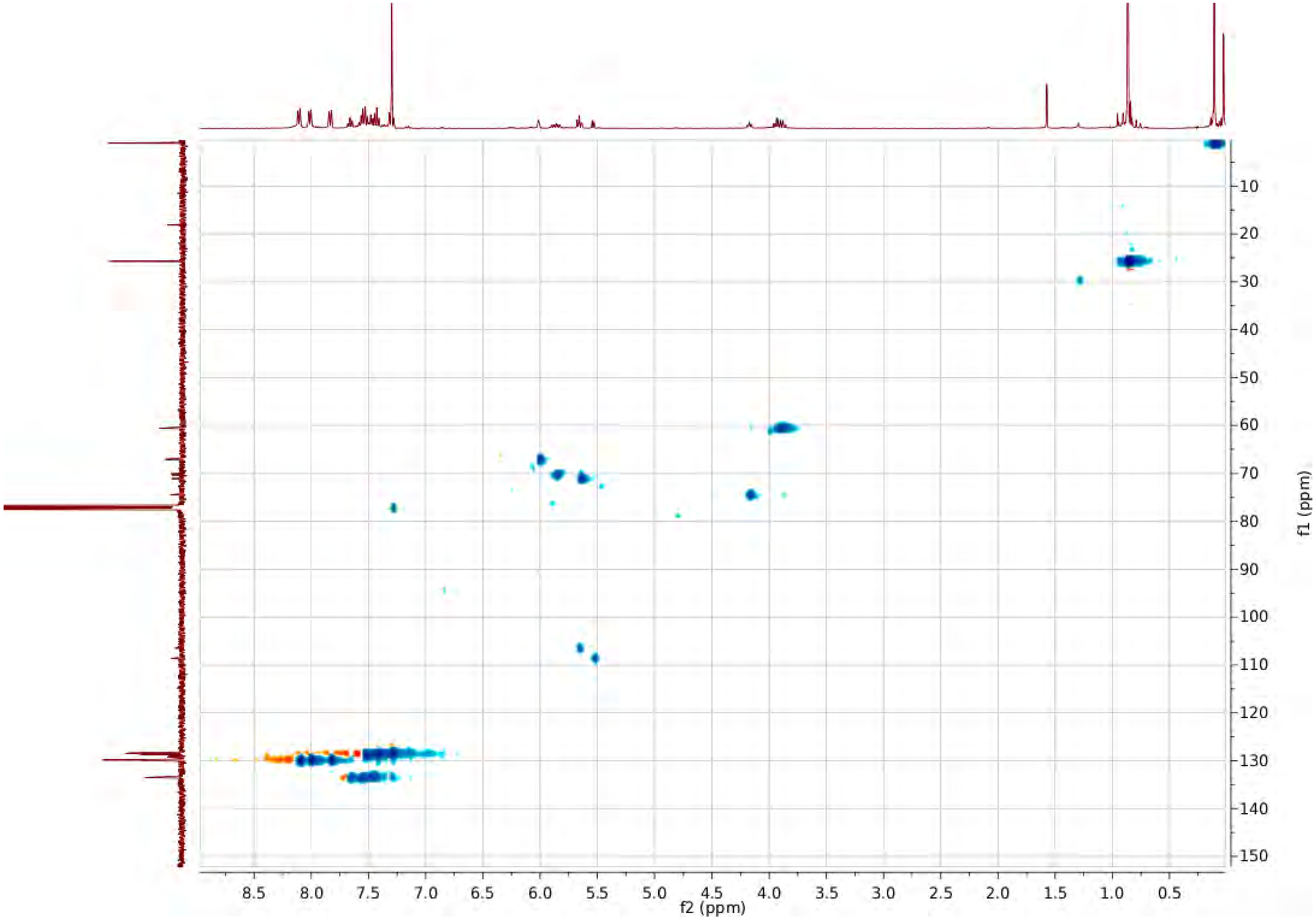

8.5
8.0
7.5
7.0
6.5
6.0
5.5
5.0
4.5
4.0
3.5
3.0
2.5
2.0
1.5
1.0
0.5
f2 (ppm)
10
20
30
40
50
60
70
80
90
100
110
120
130
140
150
f1 (ppm)

Compound **2**

Proton

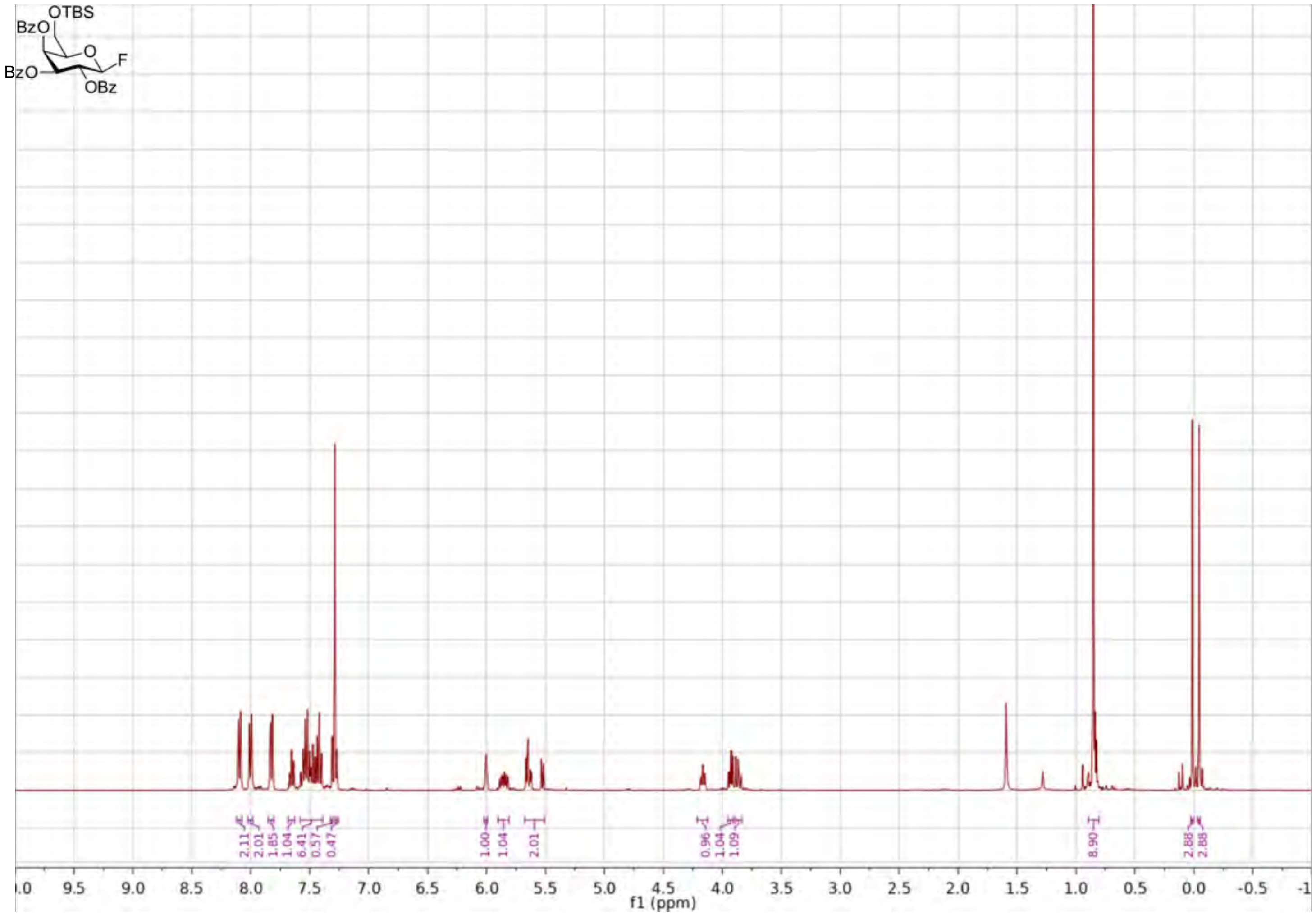

Carbon

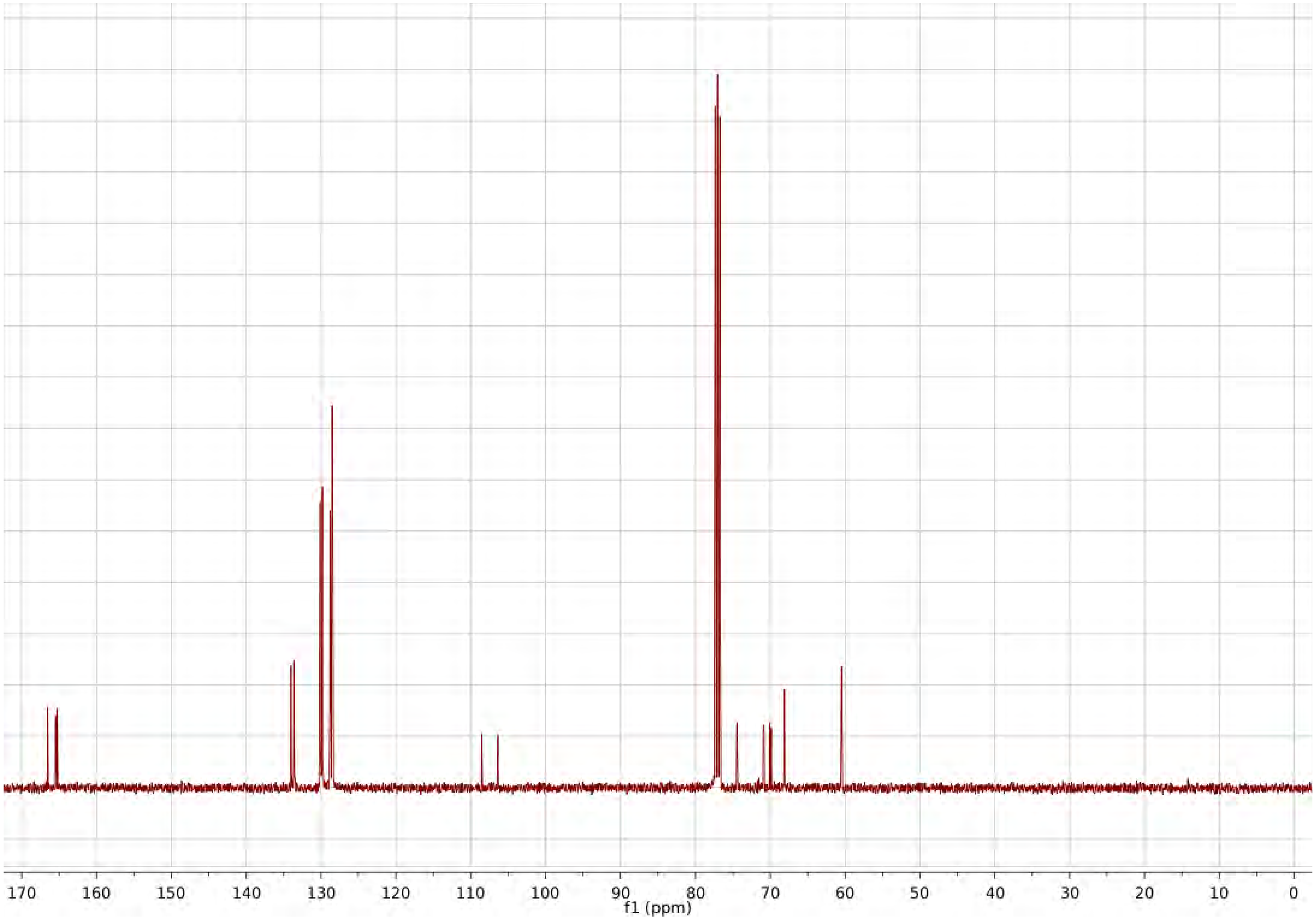

COSY

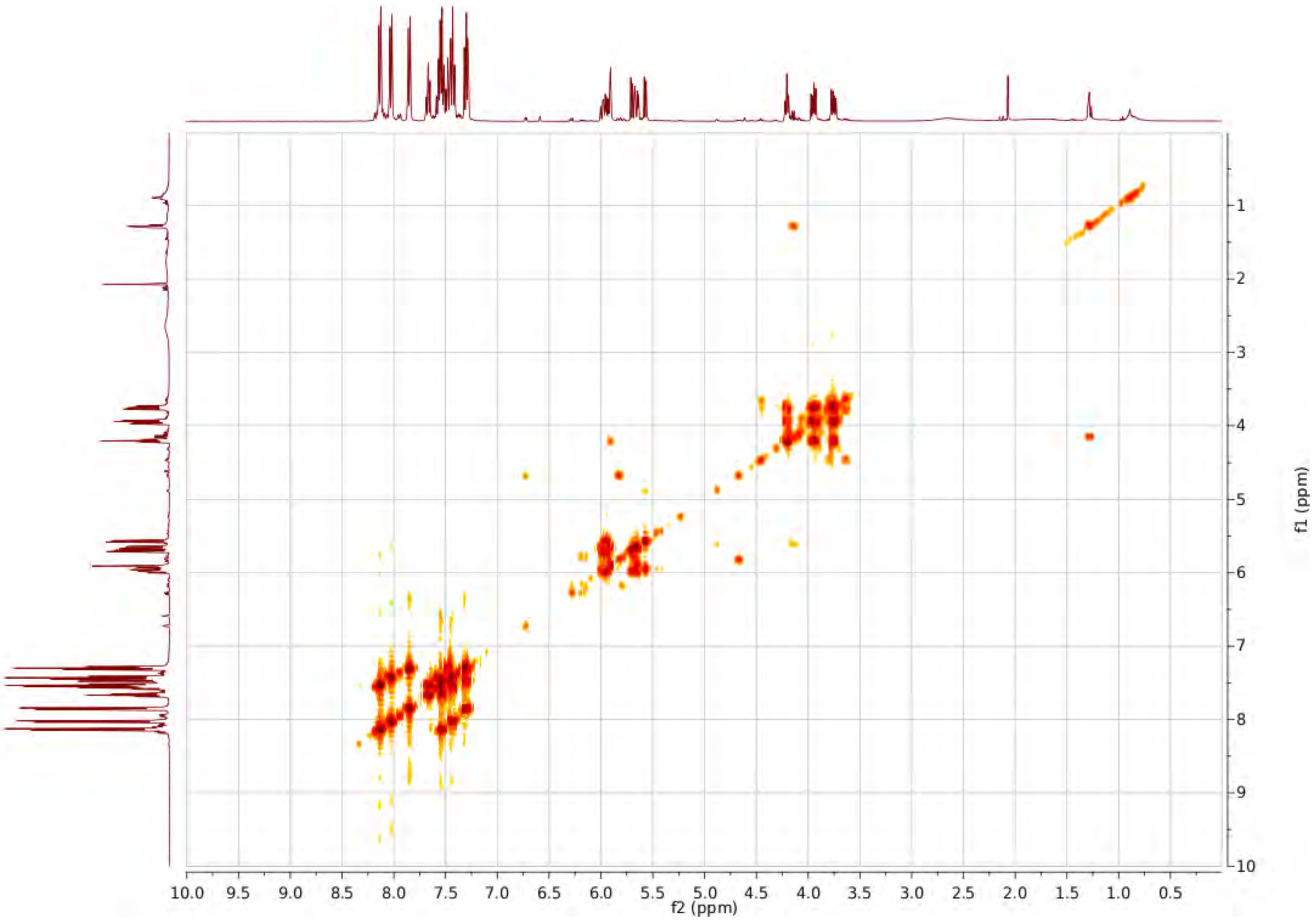

10.0
9.5
9.0
8.5
8.0
7.5
7.0
6.5
6.0
5.5
5.0
4.5
4.0
3.5
3.0
2.5
2.0
1.5
1.0
0.5
f2 (ppm)
1
2
3
4
5
6
7
8
9
10
f1 (ppm)

HSQC

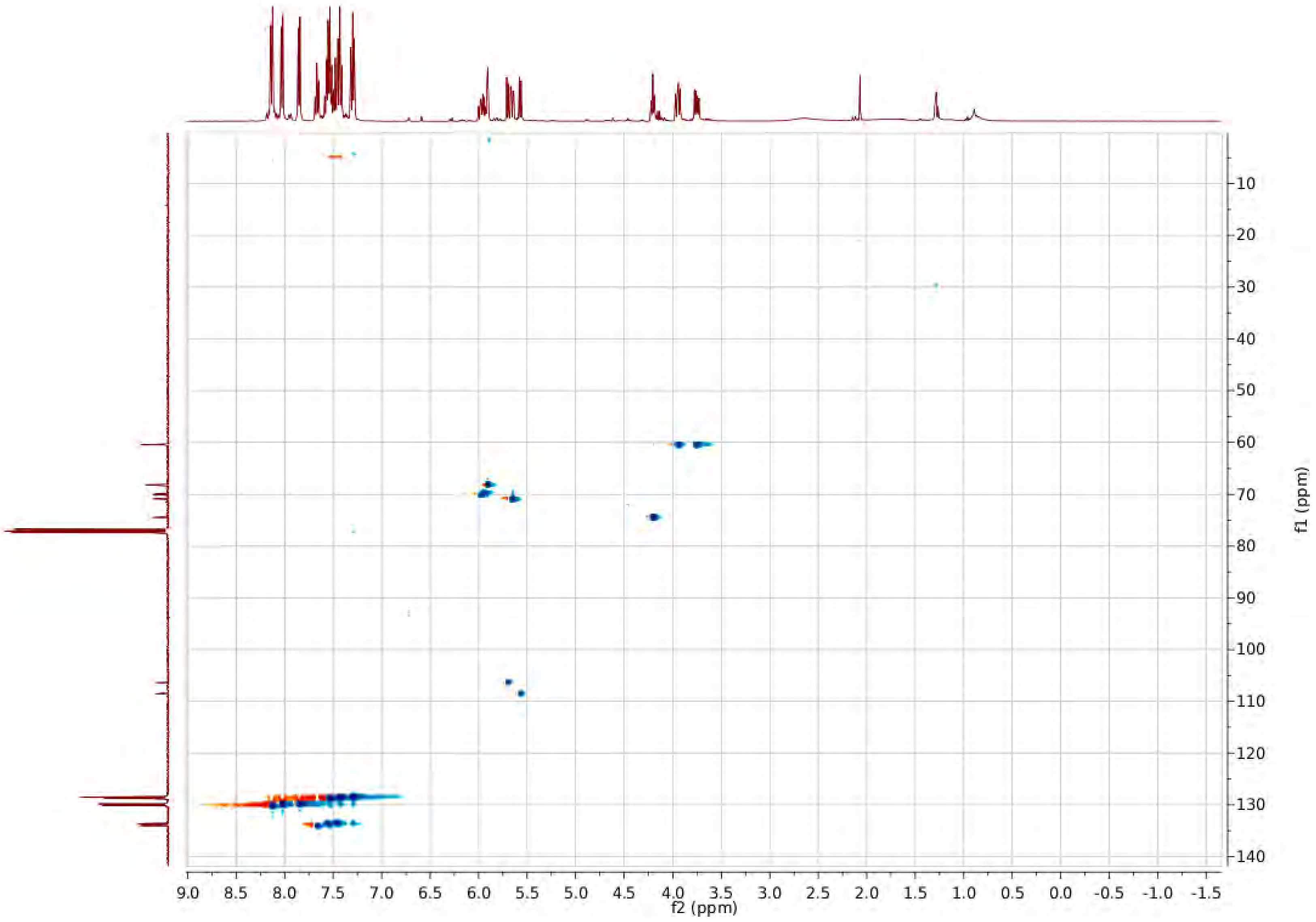

9.0 8.5 8.0 7.5 7.0 6.5 6.0 5.5 5.0 4.5 4.0 3.5 3.0 2.5 2.0 1.5 1.0 0.5 0.0 -0.5 -1.0 -1.5
f2 (ppm)
10 20 30 40 50 60 70 80 90 100 110 120 130 140
f1 (ppm)

19F

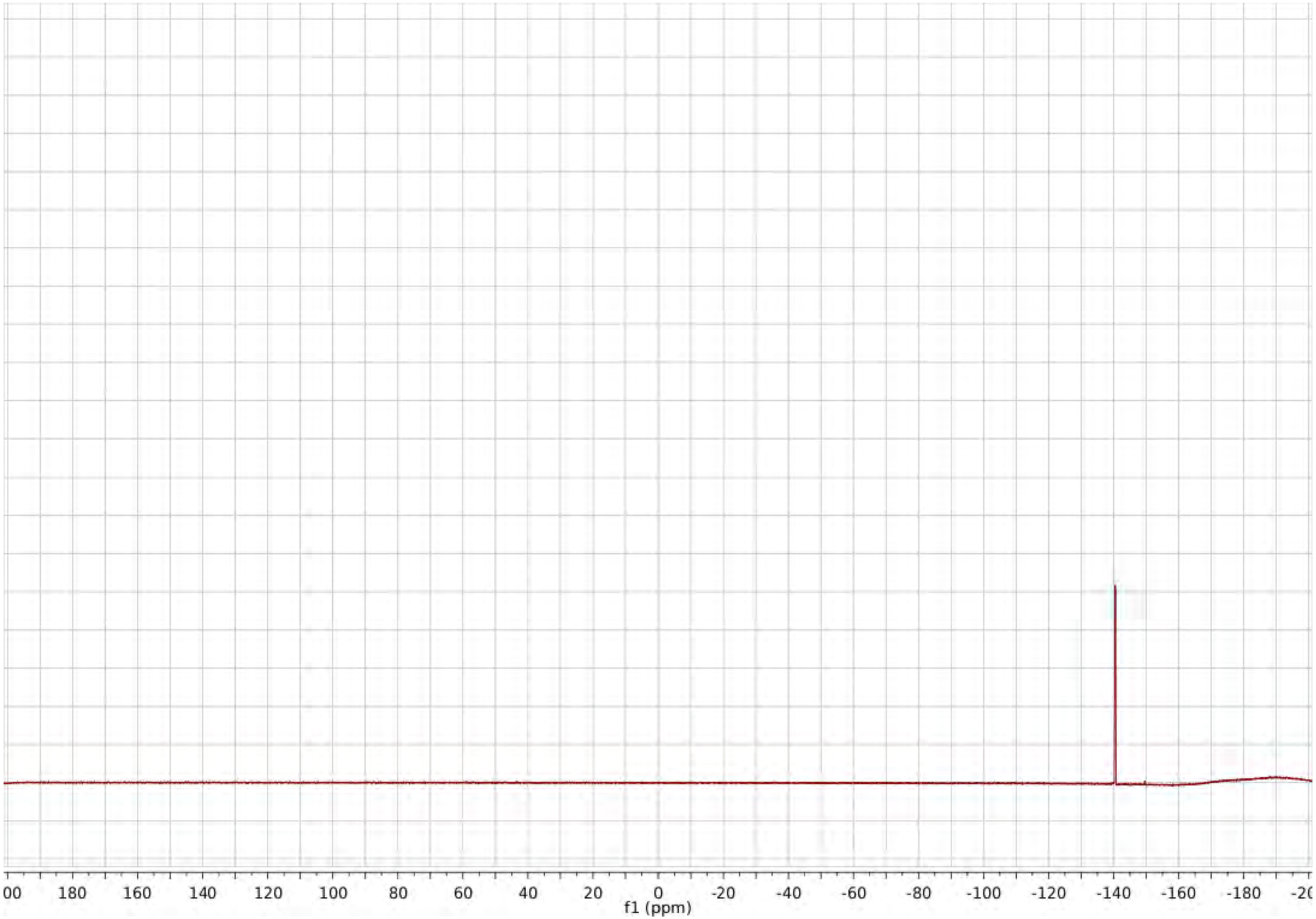

00
180
160
140
120
100
80
60
40
20
0
-20
-40
-60
-80
-100
-120
-140
-160
-180
-2(
f1 (ppm)

Compound **7**

Proton

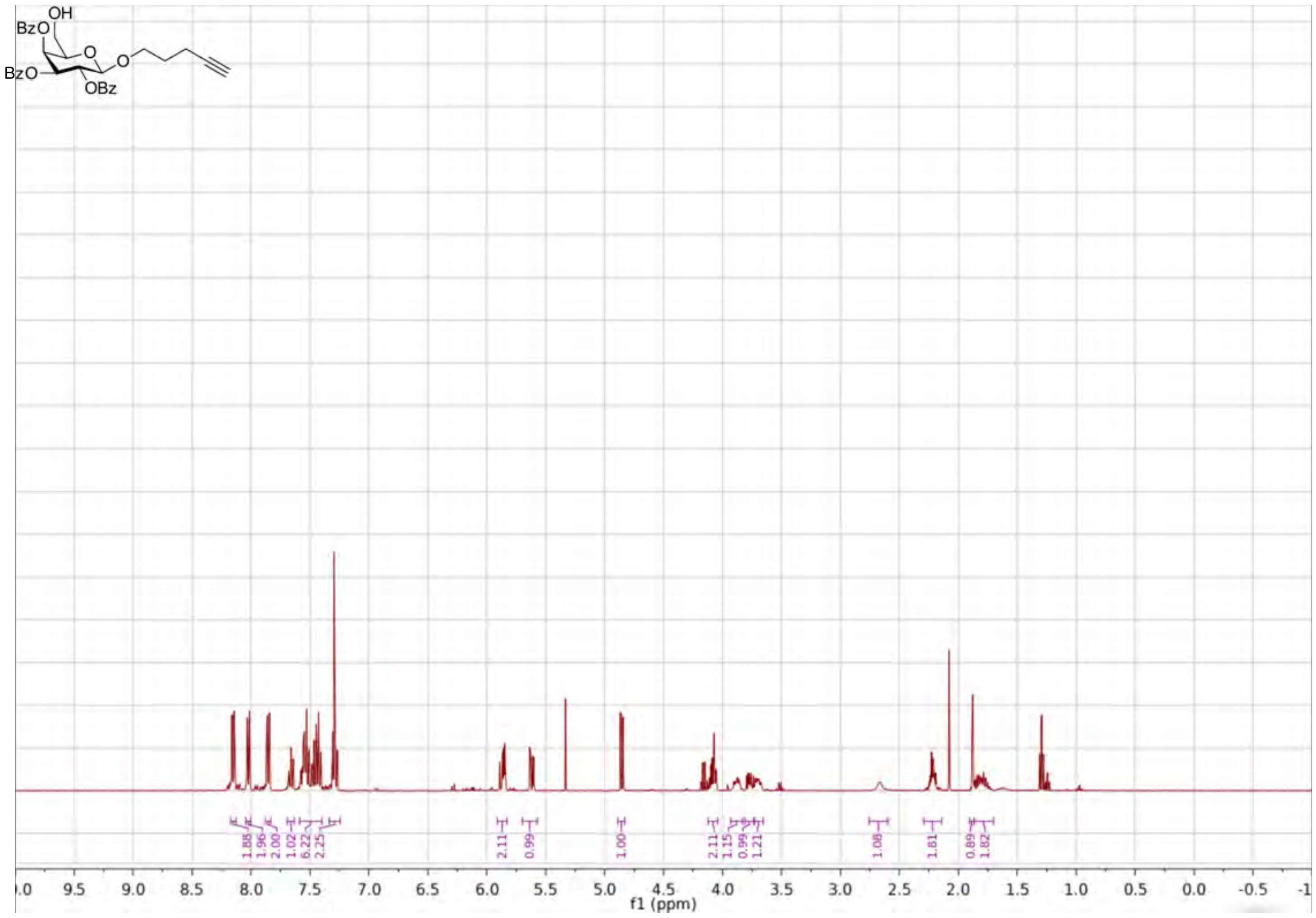

Carbon

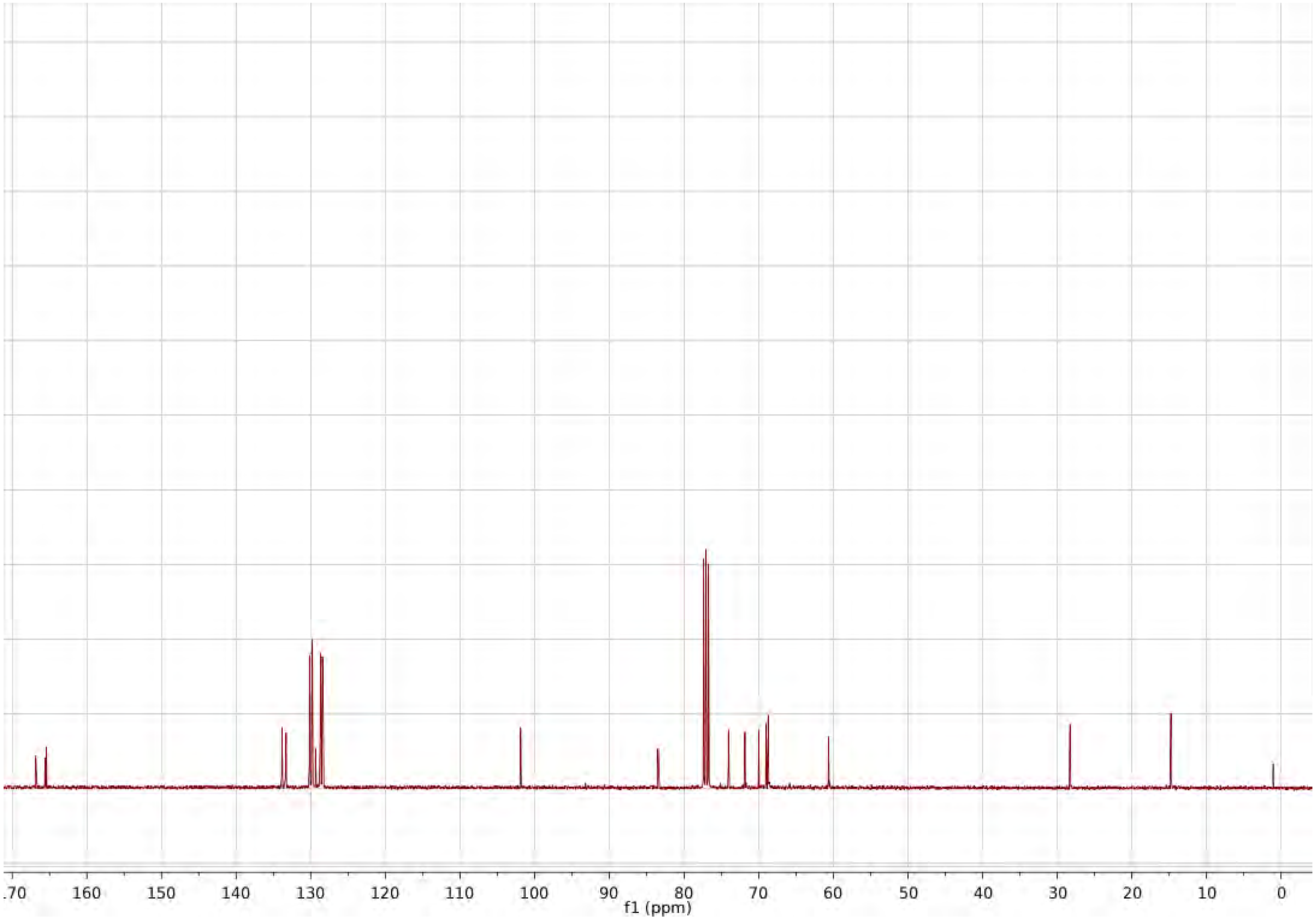

COSY

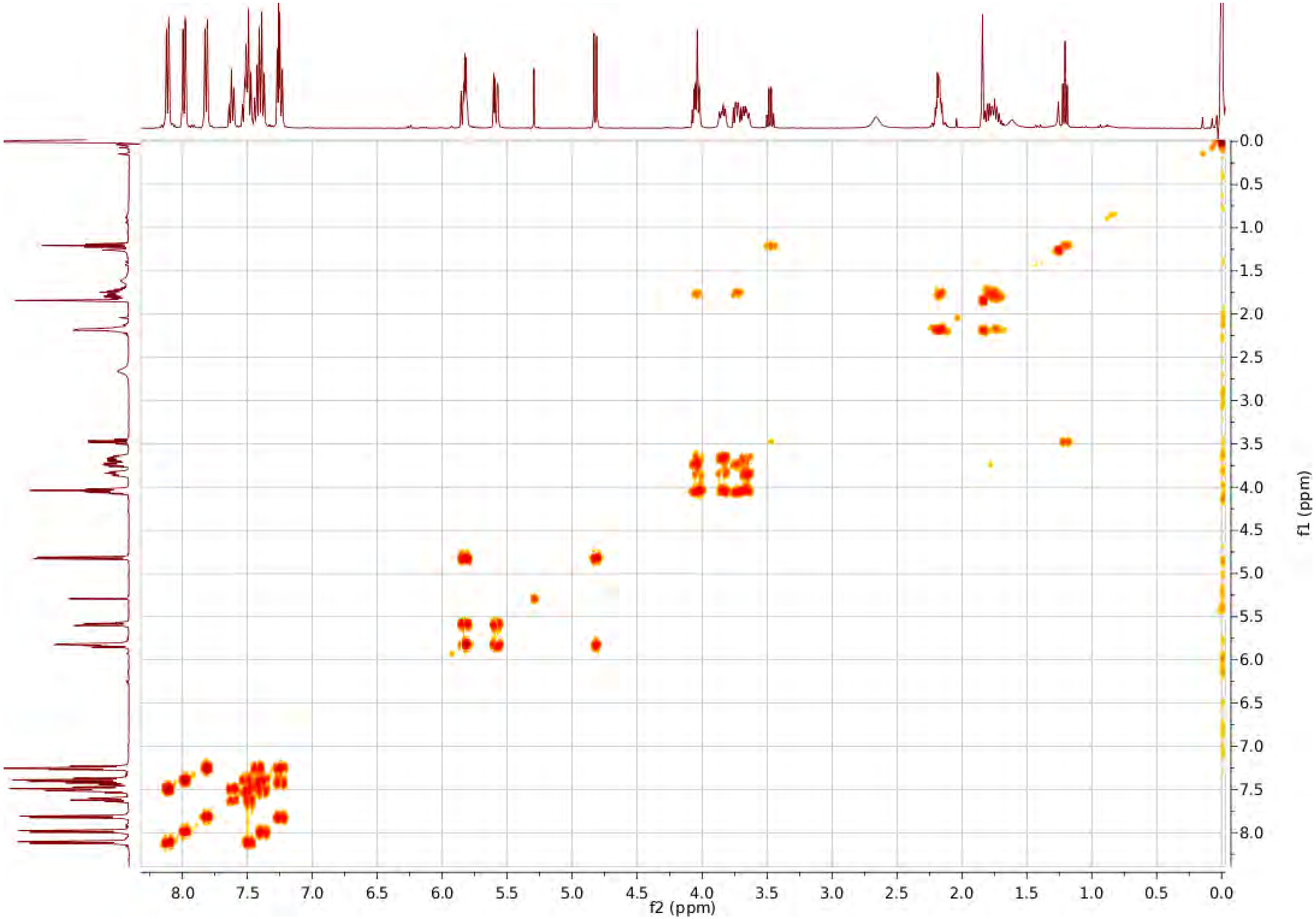

HSQC

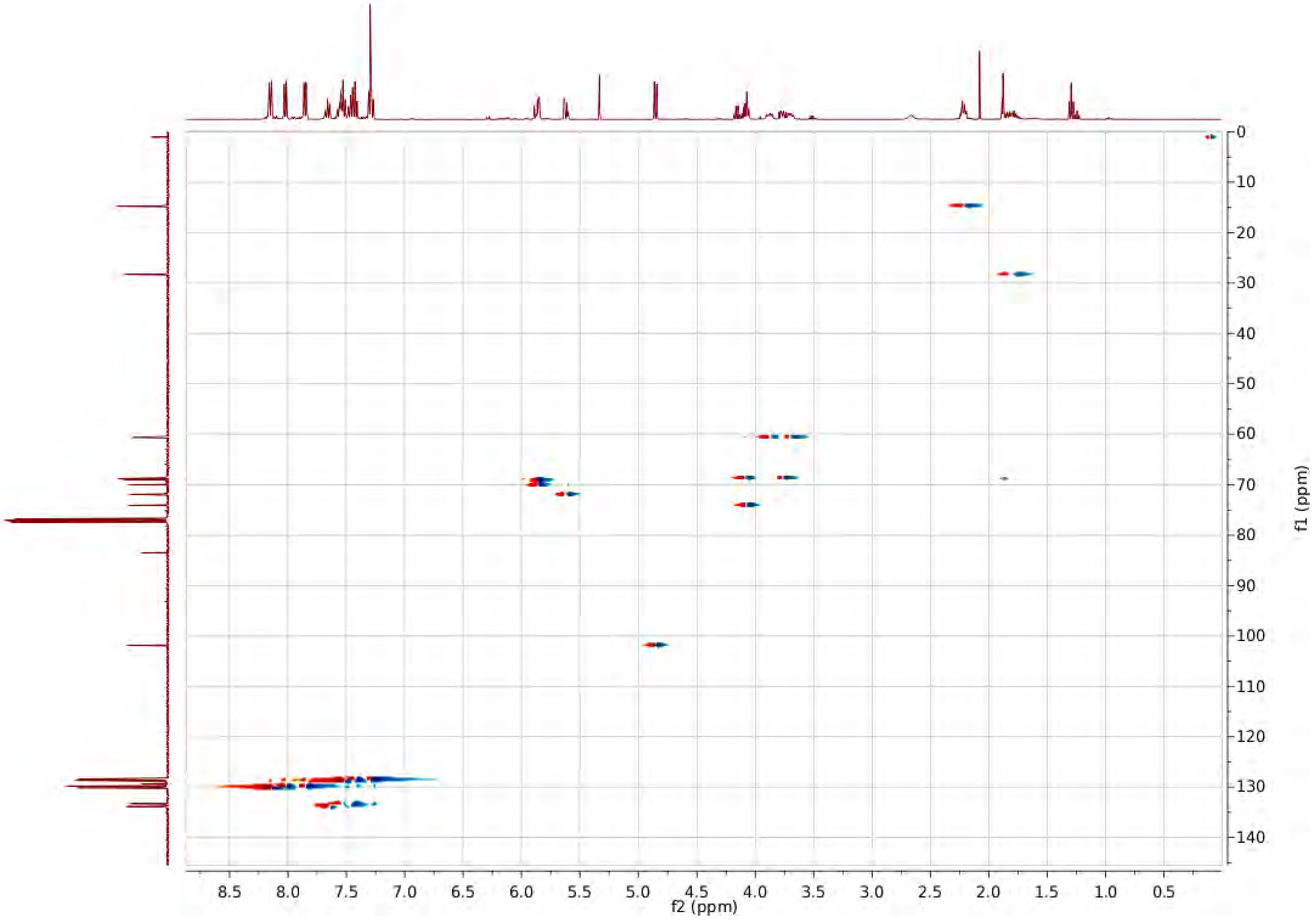

8.5
8.0
7.5
7.0
6.5
6.0
5.5
5.0
4.5
4.0
3.5
3.0
2.5
2.0
1.5
1.0
0.5
f2 (ppm)
0
10
20
30
40
50
60
70
80
90
100
110
120
130
140
f1 (ppm)

Compound **8**

Proton

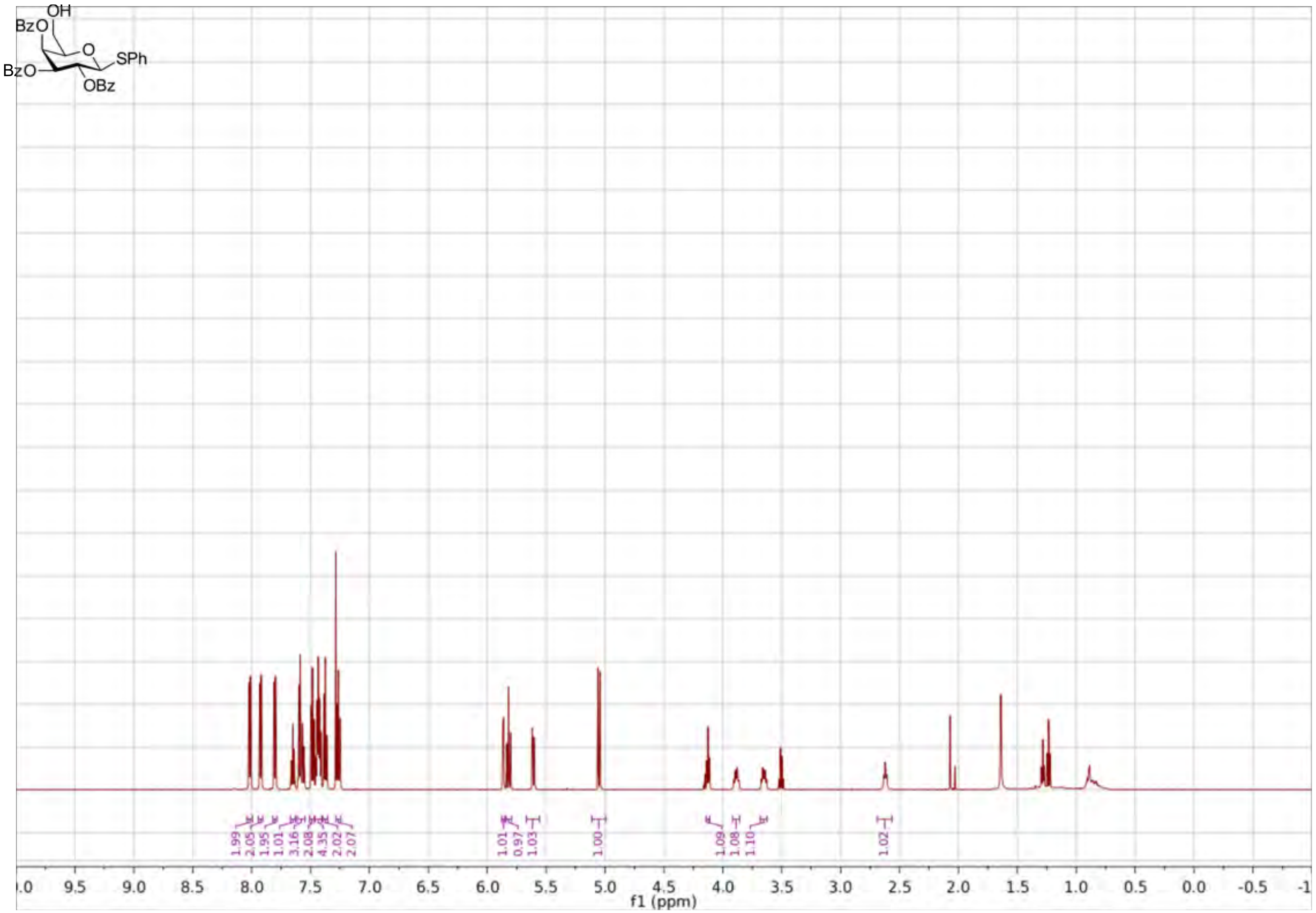

Carbon

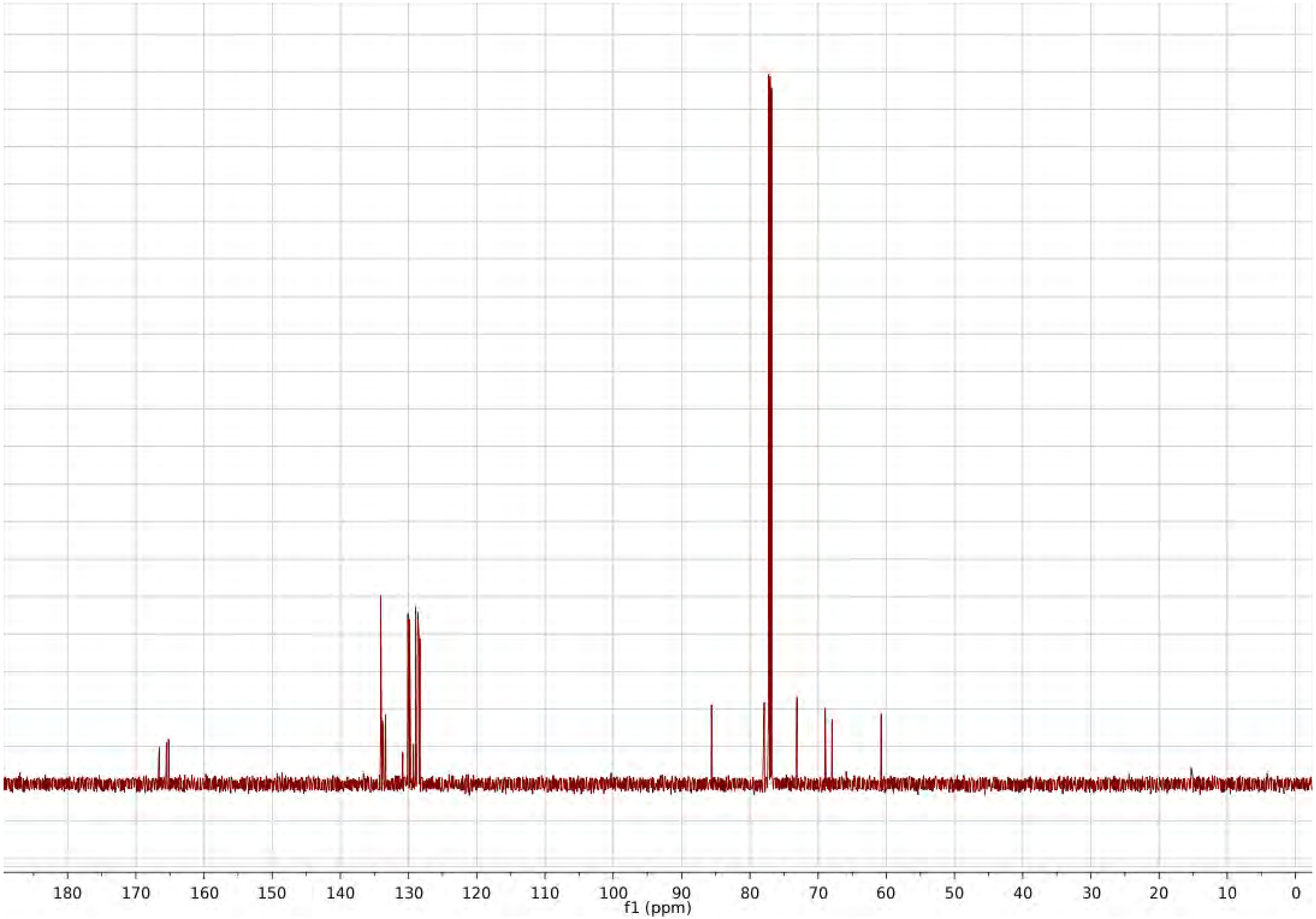

180
170
160
150
140
130
120
110
100
90
80
70
60
50
40
30
20
10
0
f1 (ppm)

COSY

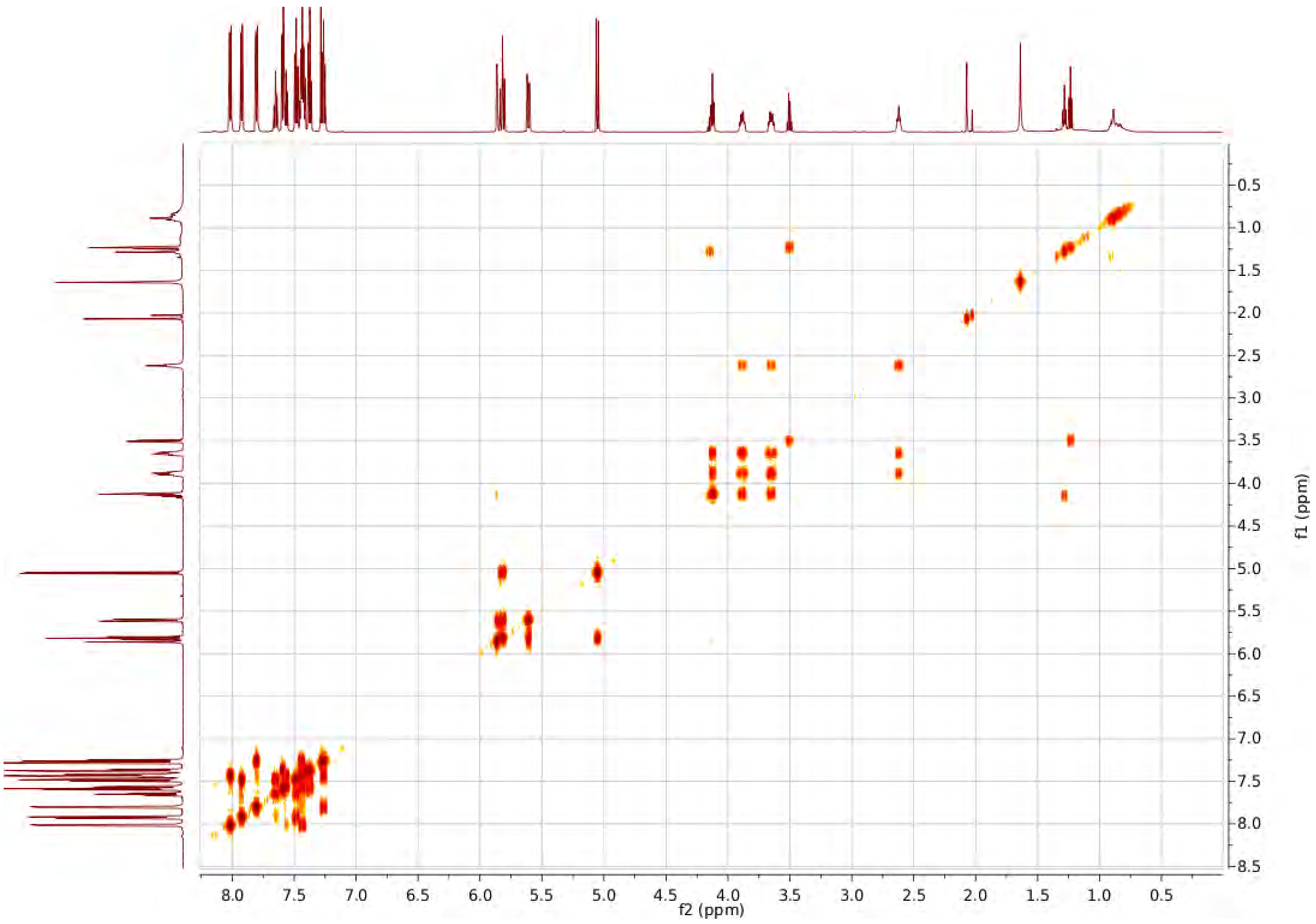

Compound **12**

Proton

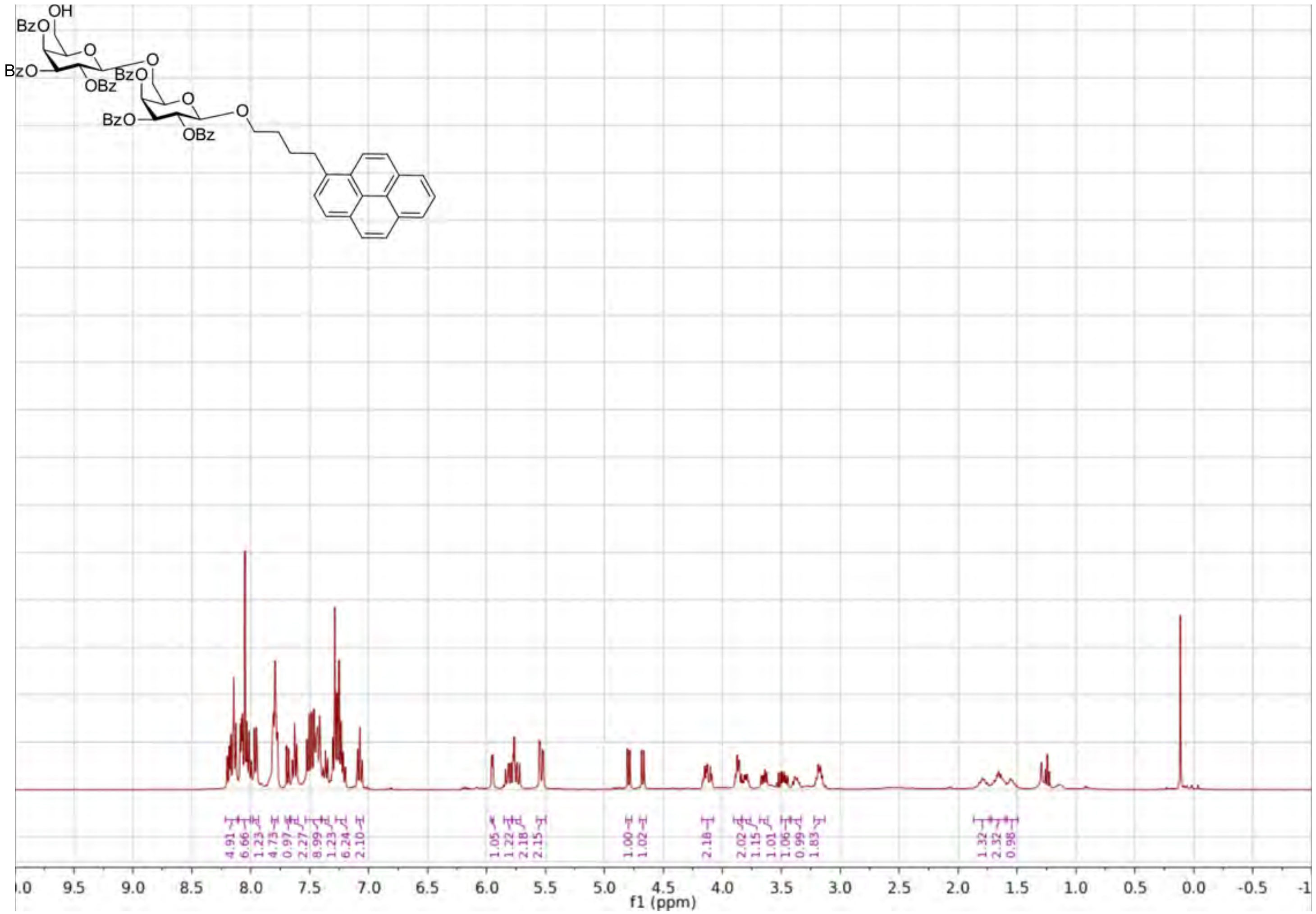

Carbon

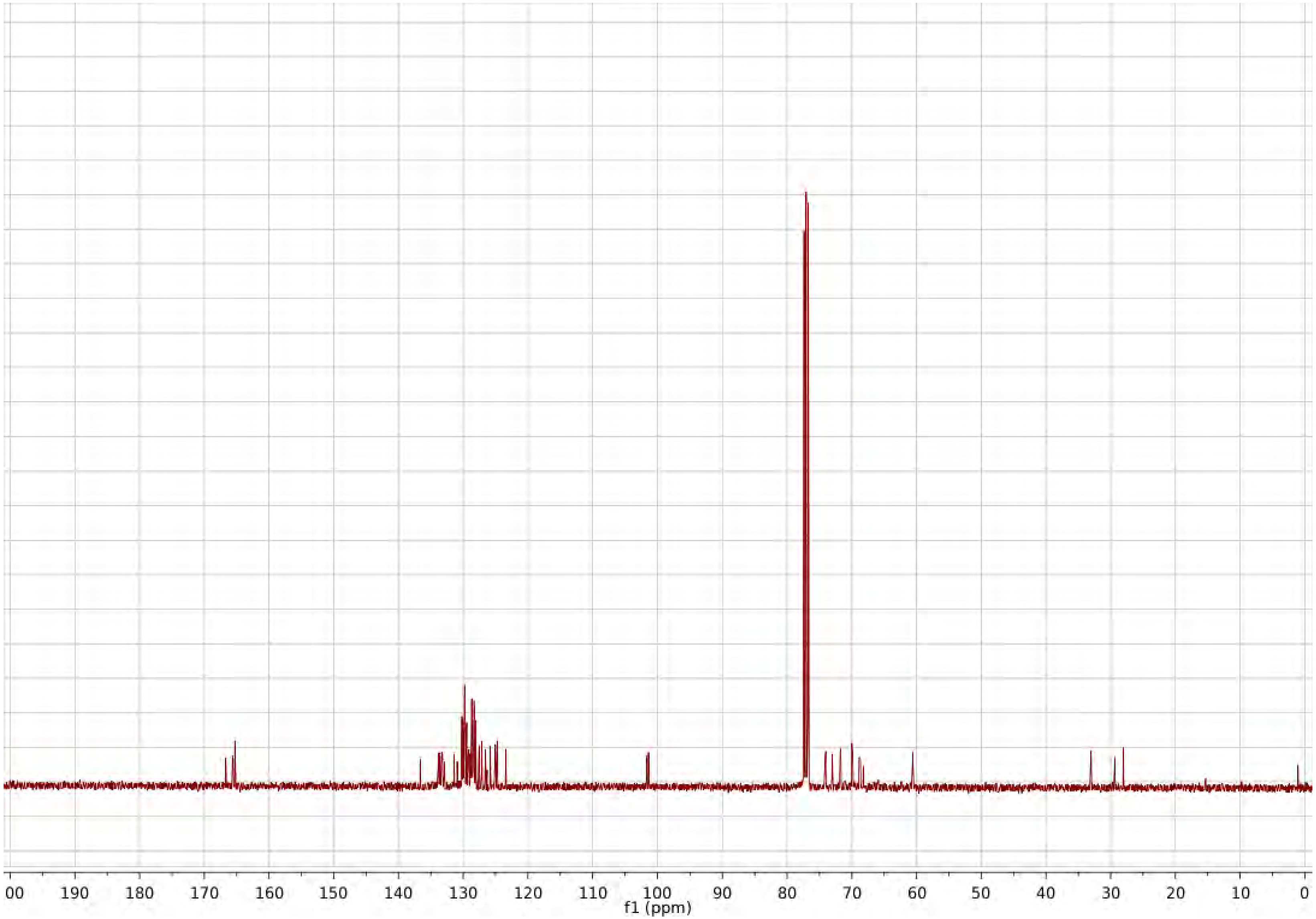
00
190
180
170
160
150
140
130
120
110
100
90
80
70
60
50
40
30
20
10
0
f1 (ppm)

Compound **21**

Proton

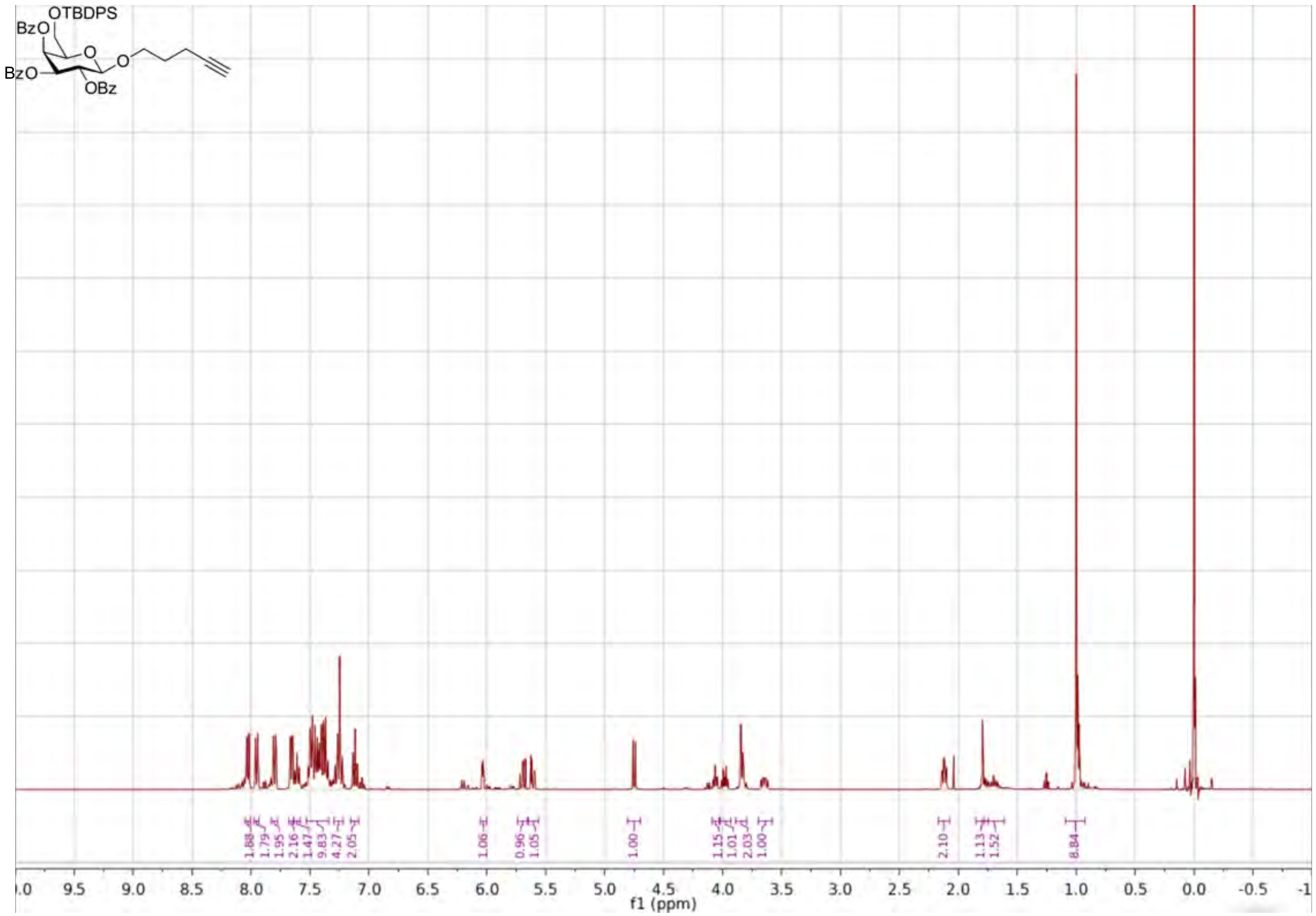

Carbon

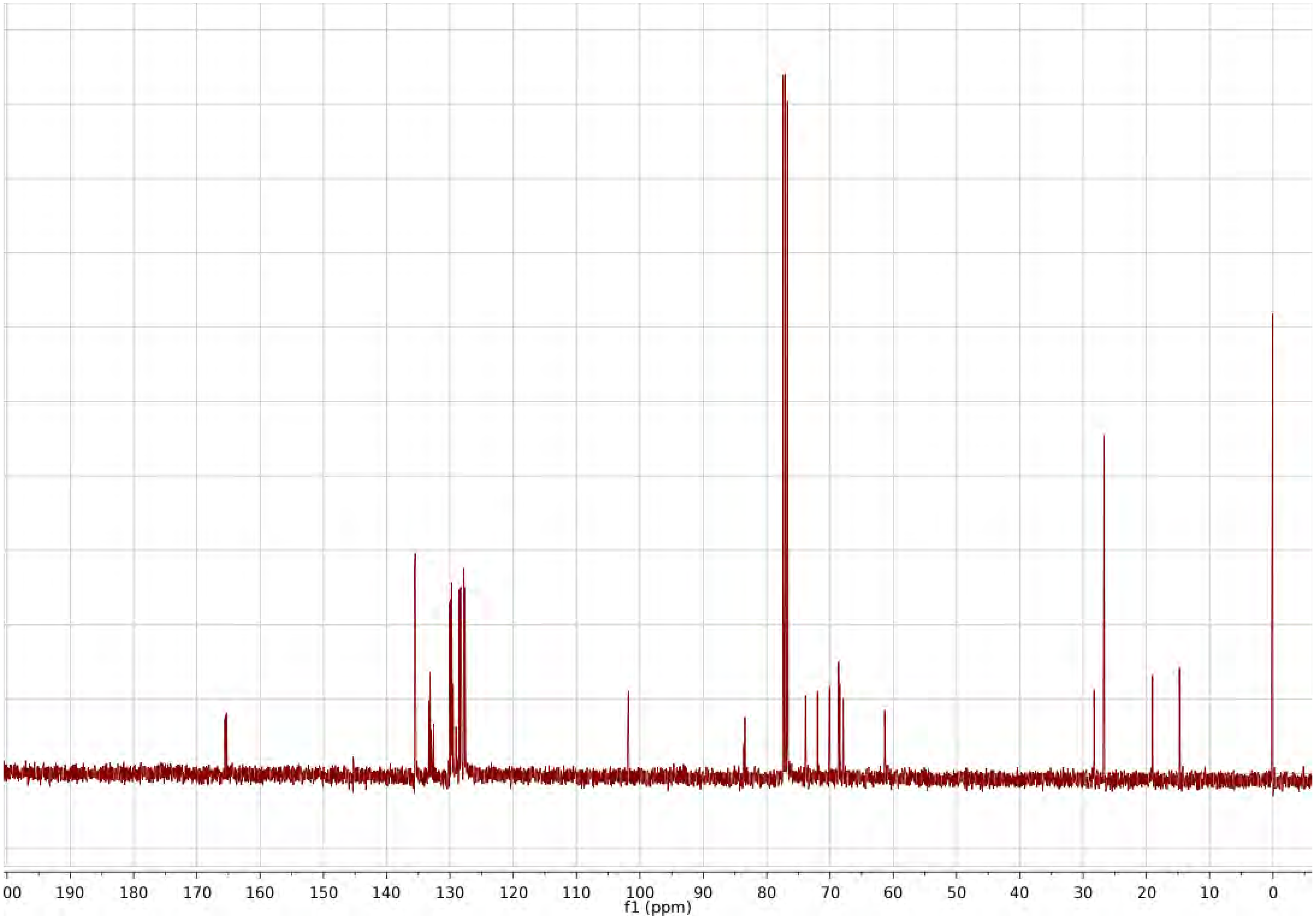
00
190
180
170
160
150
140
130
120
110
100
90
80
70
60
50
40
30
20
10
0
f1 (ppm)

Compound **23**

Proton

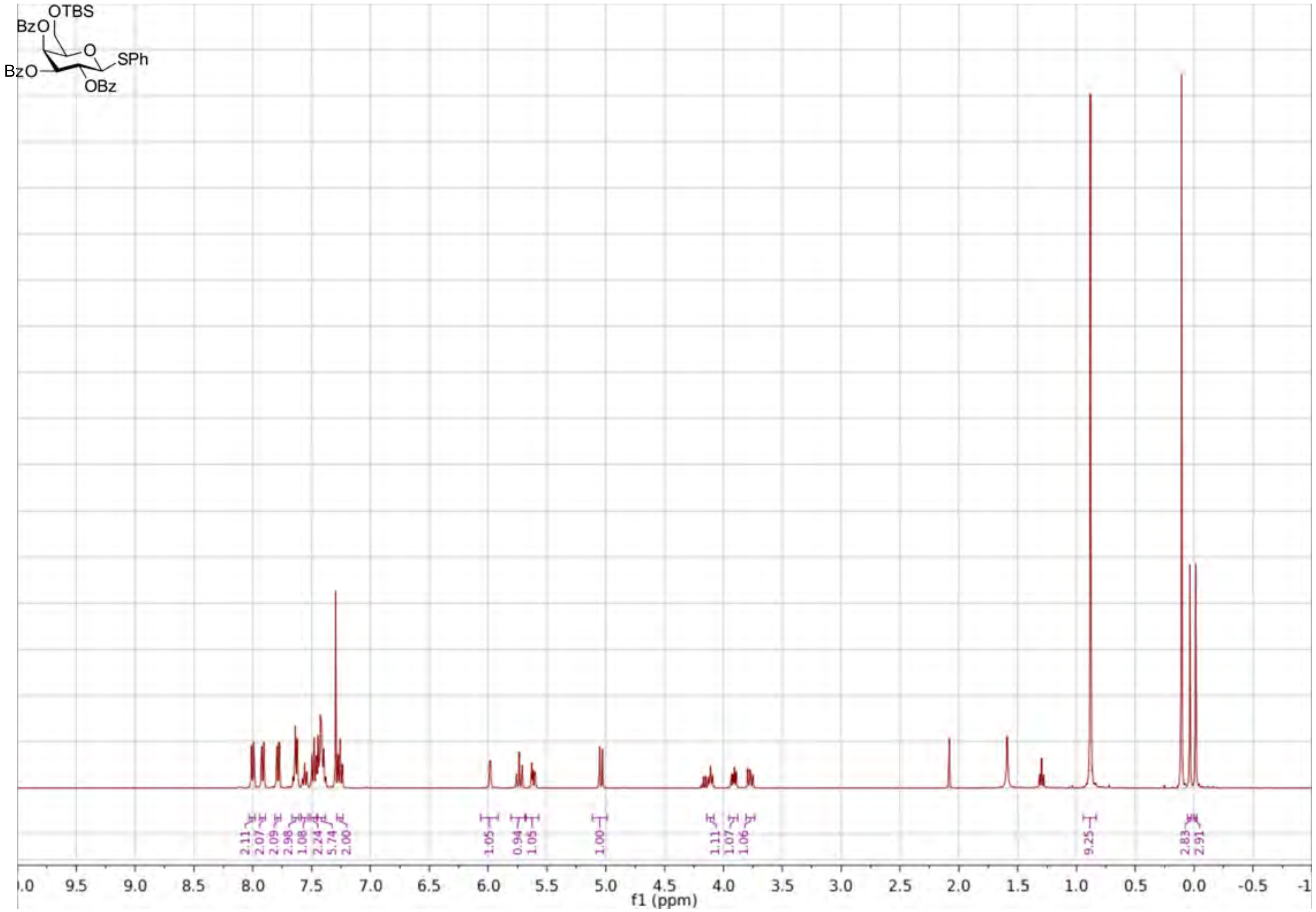

Compound $\mathbf{4_1a}$

Proton

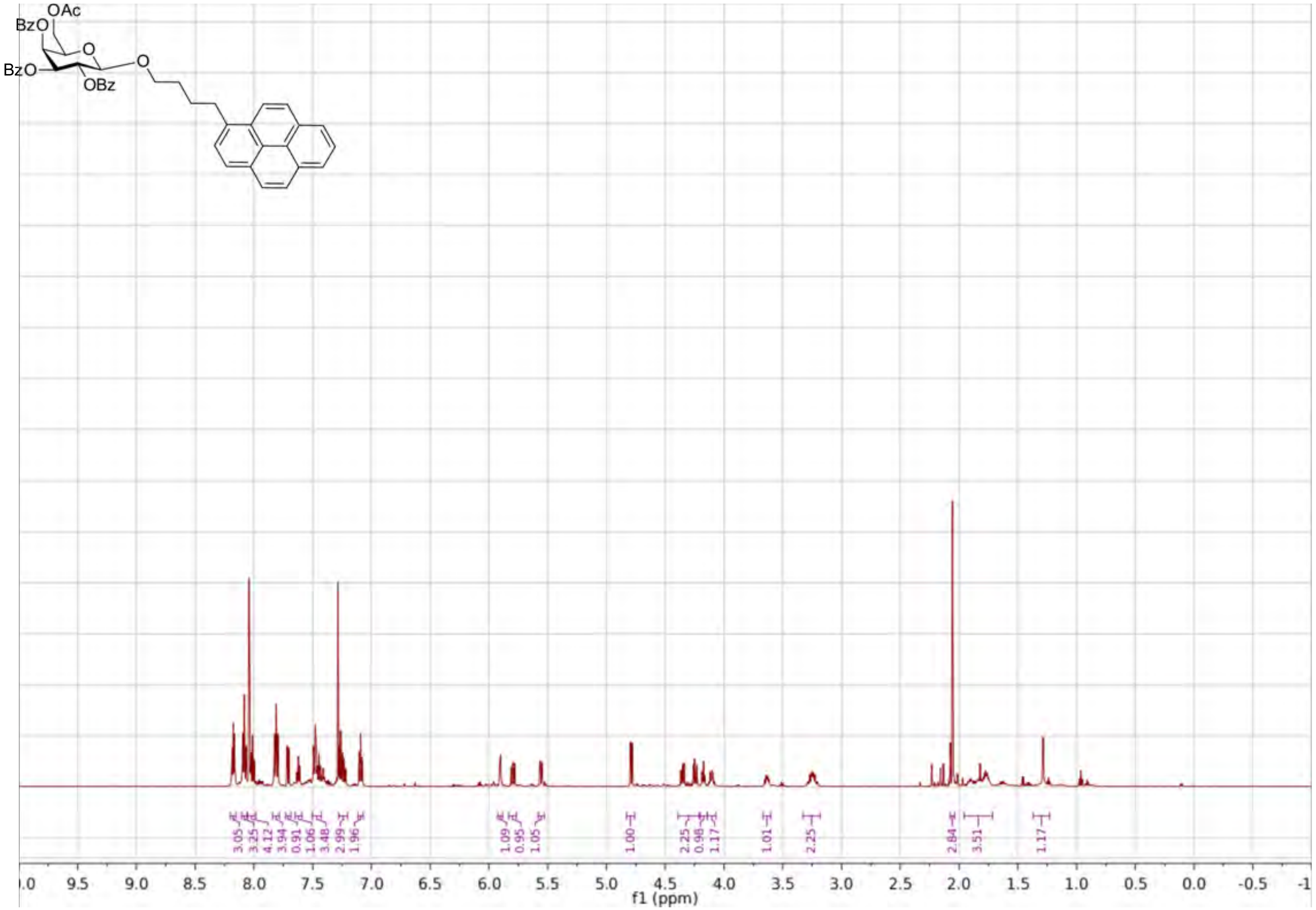

Carbon

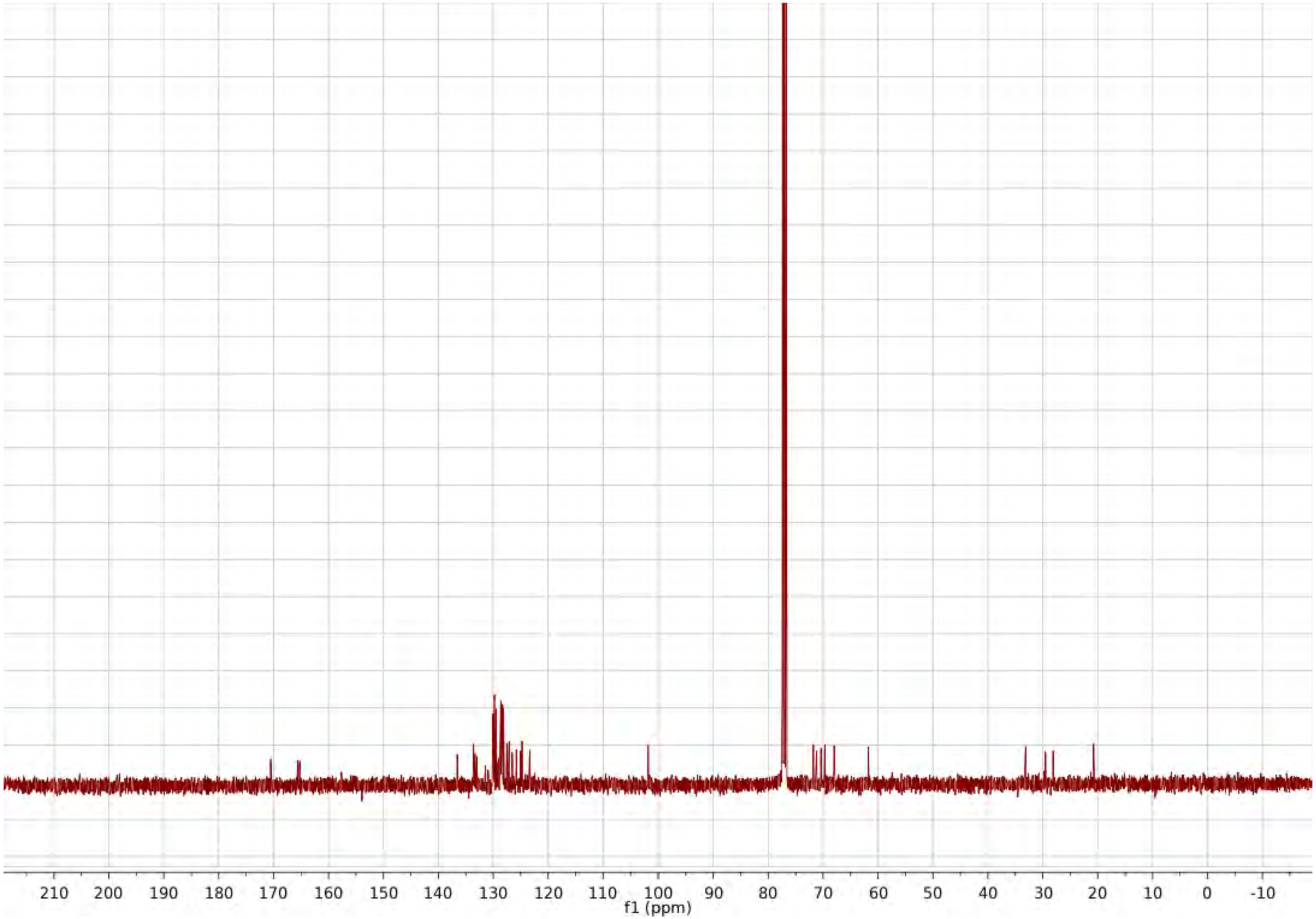

210 200 190 180 170 160 150 140 130 120 110 100 90 80 70 60 50 40 30 20 10 0 -10
f1 (ppm)

COSY

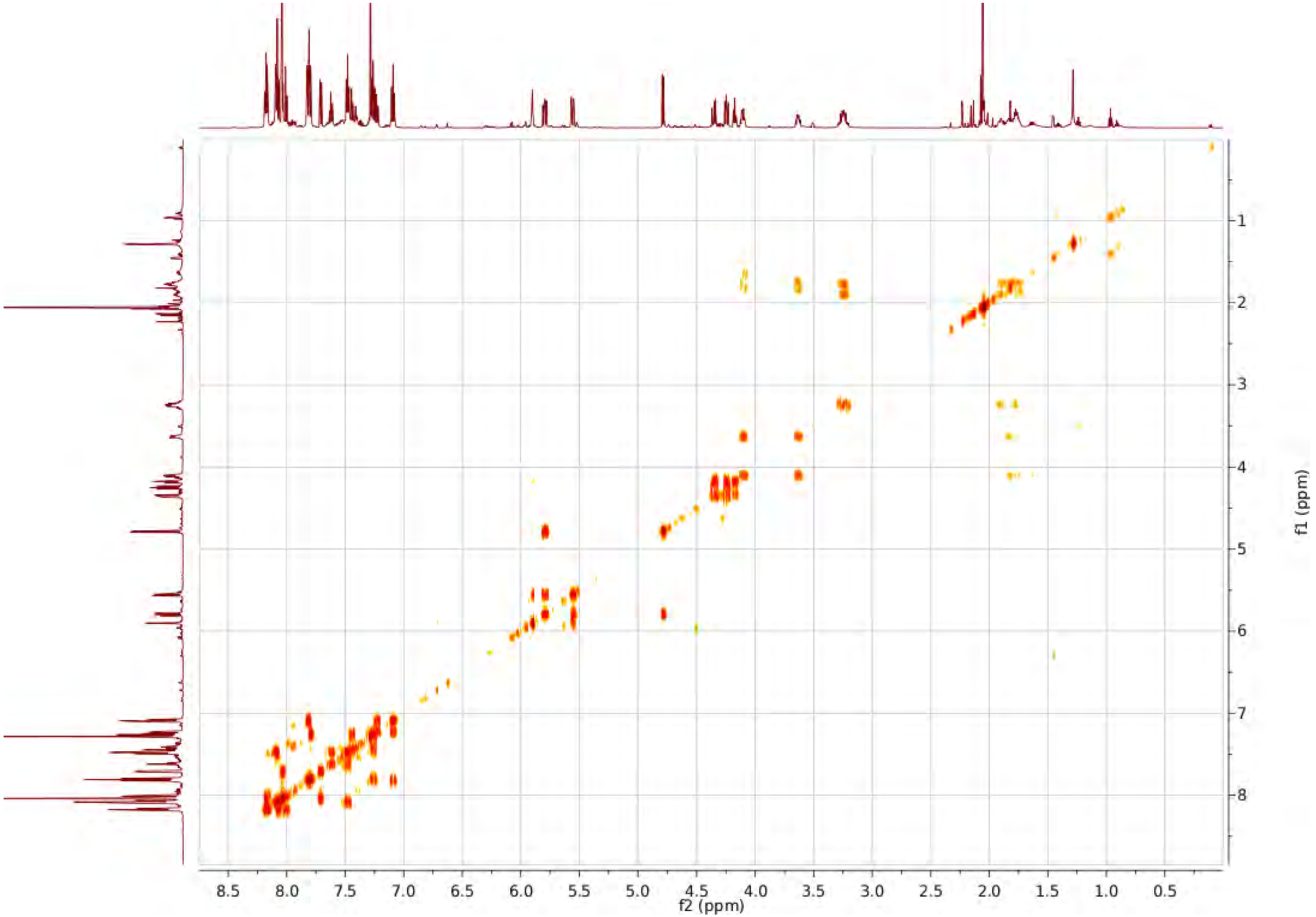
8.5
8.0
7.5
7.0
6.5
6.0
5.5
5.0
4.5
4.0
3.5
3.0
2.5
2.0
1.5
1.0
0.5
f2 (ppm)
1
2
3
4
5
6
7
8
f1 (ppm)

HSQC

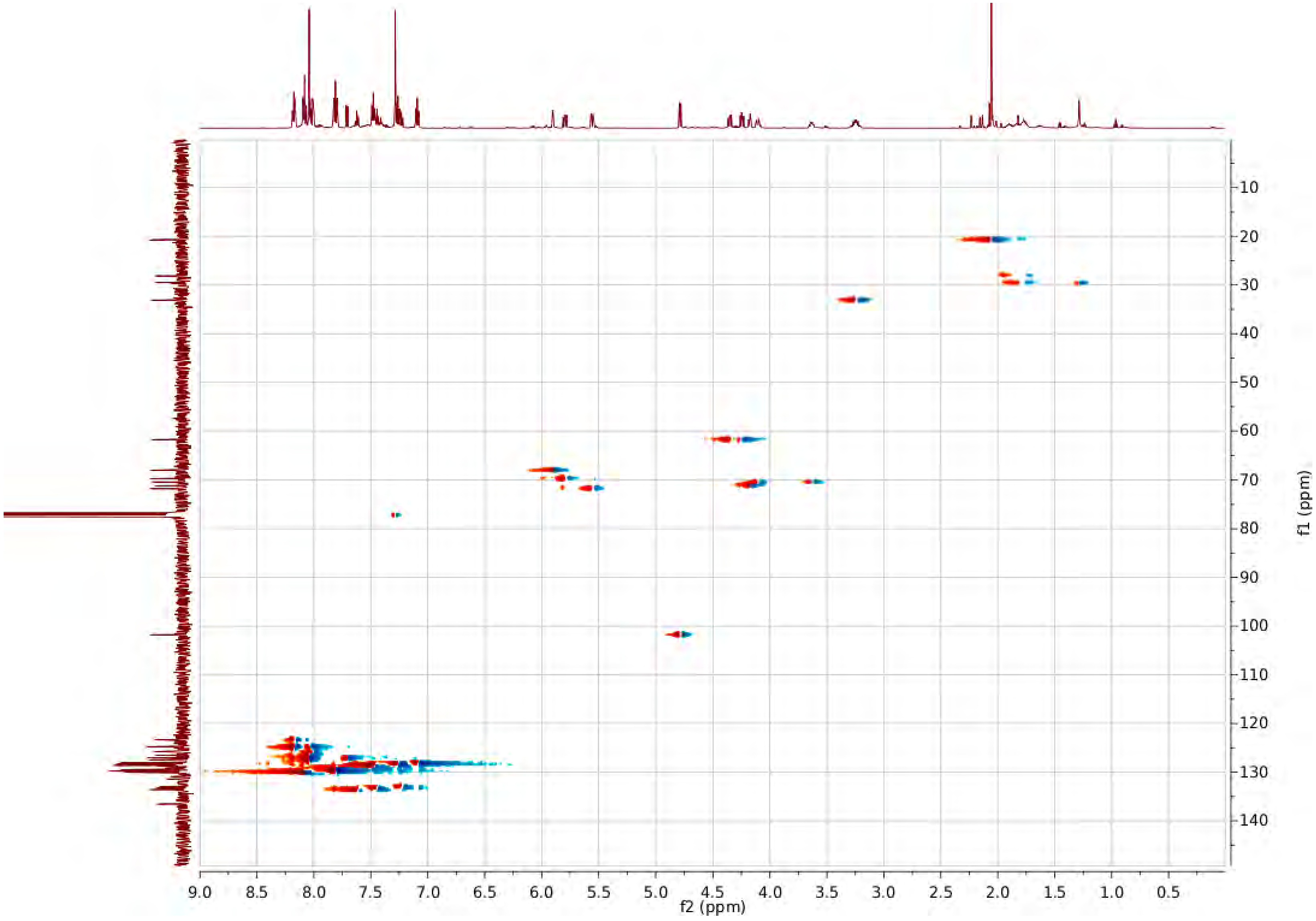

9.0
8.5
8.0
7.5
7.0
6.5
6.0
5.5
5.0
4.5
4.0
3.5
3.0
2.5
2.0
1.5
1.0
0.5
f2 (ppm)
10
20
30
40
50
60
70
80
90
100
110
120
130
140
f1 (ppm)

Compound 4_2a

Proton

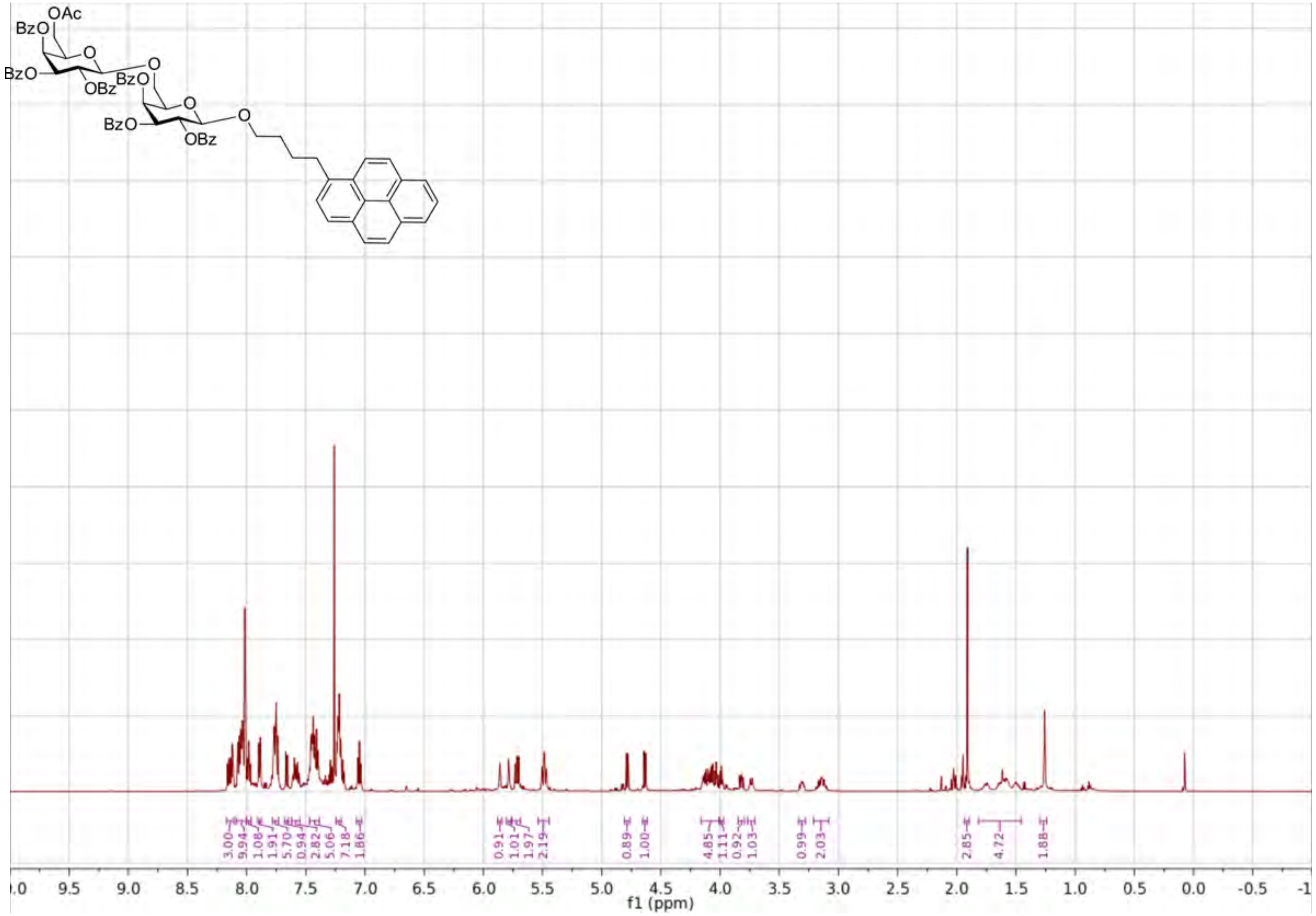

Carbon

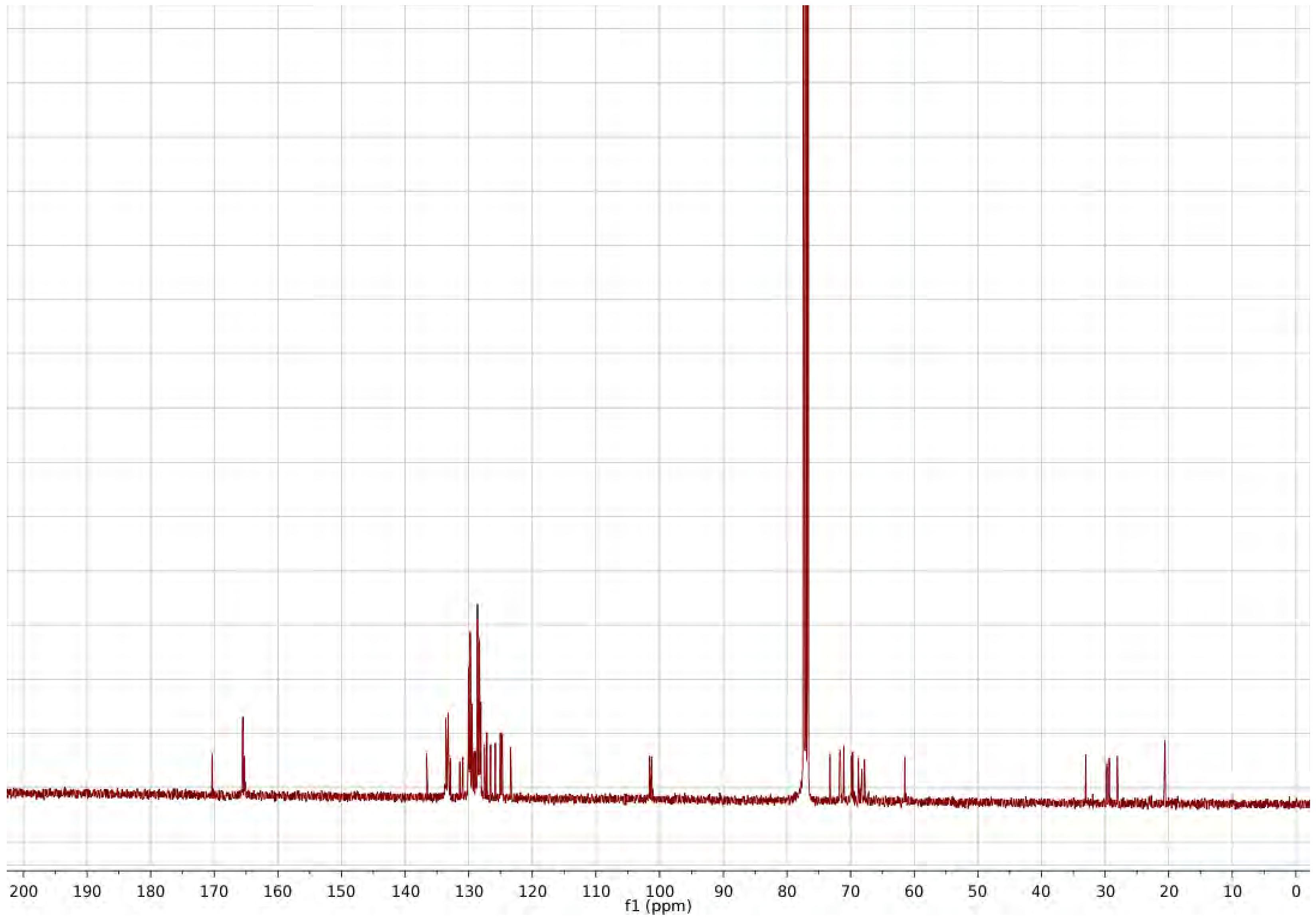
200
190
180
170
160
150
140
130
120
110
100
90
80
70
60
50
40
30
20
10
0
f1 (ppm)

COSY

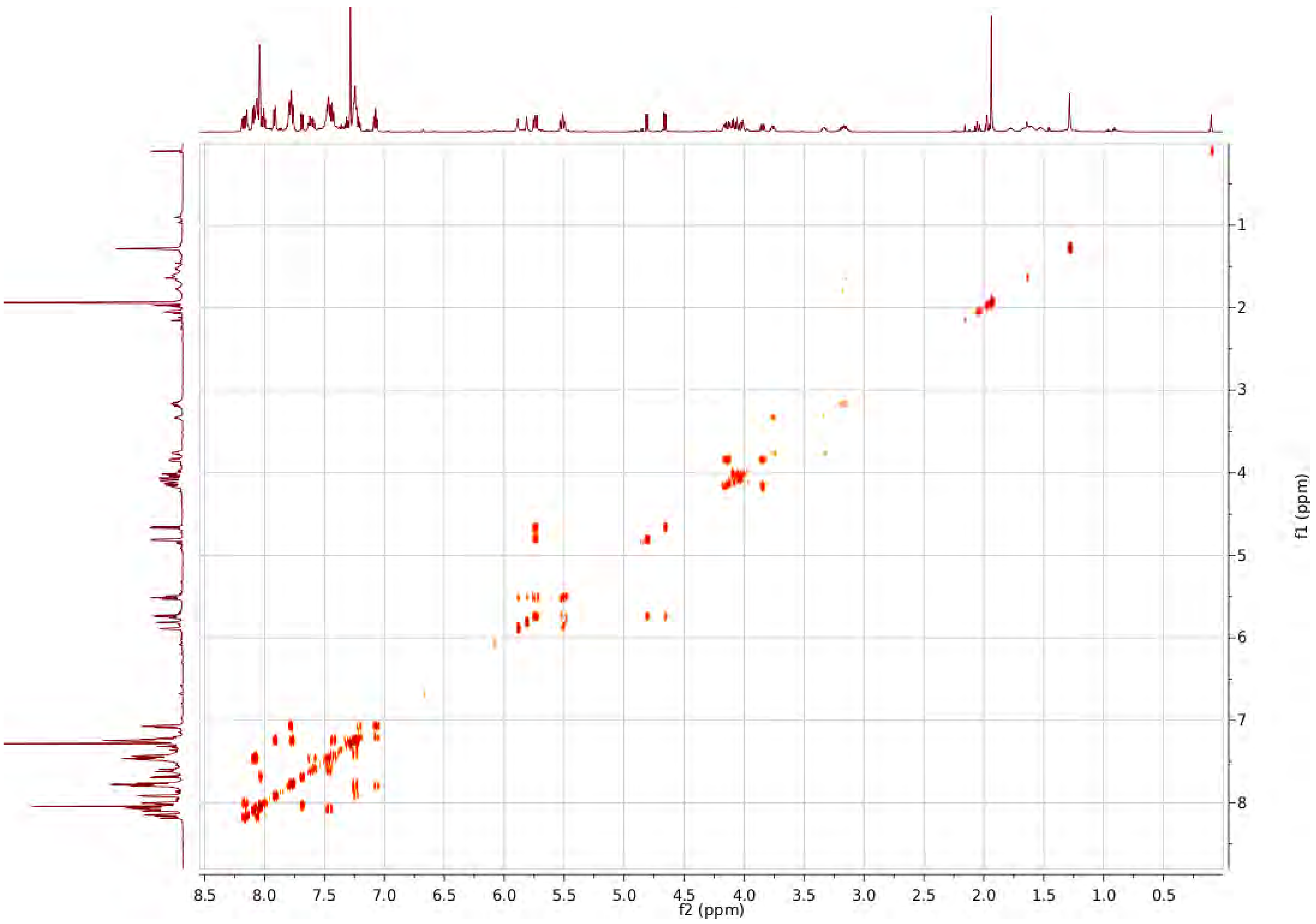

8.5
8.0
7.5
7.0
6.5
6.0
5.5
5.0
4.5
4.0
3.5
3.0
2.5
2.0
1.5
1.0
0.5
f2 (ppm)
1
2
3
4
5
6
7
8
f1 (ppm)

HSQC

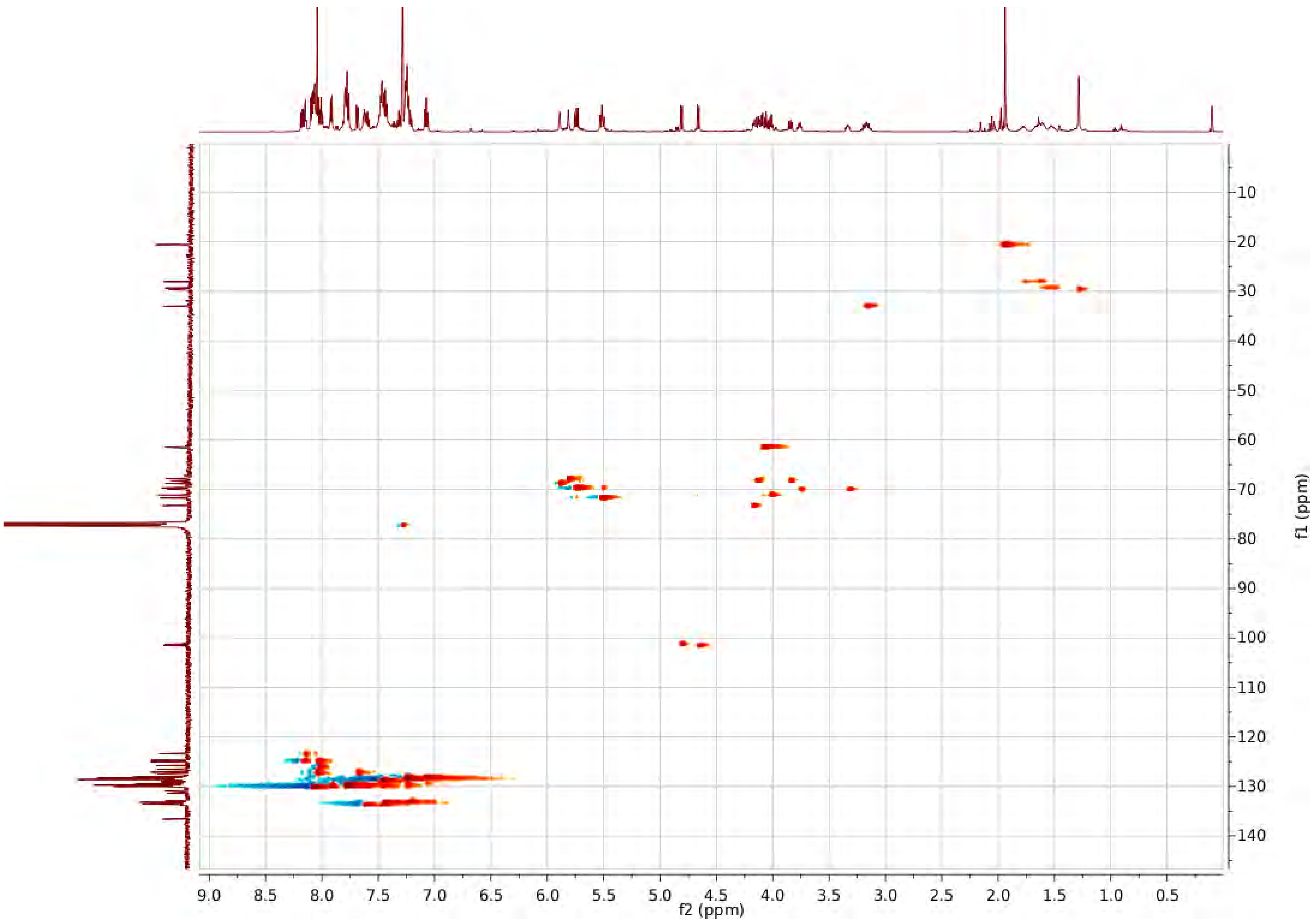

10
20
30
40
50
60
70
80
90
100
110
120
130
140
f1 (ppm)
9.0
8.5
8.0
7.5
7.0
6.5
6.0
5.5
5.0
4.5
4.0
3.5
3.0
2.5
2.0
1.5
1.0
0.5
f2 (ppm)

Compound **4₃a**

Proton

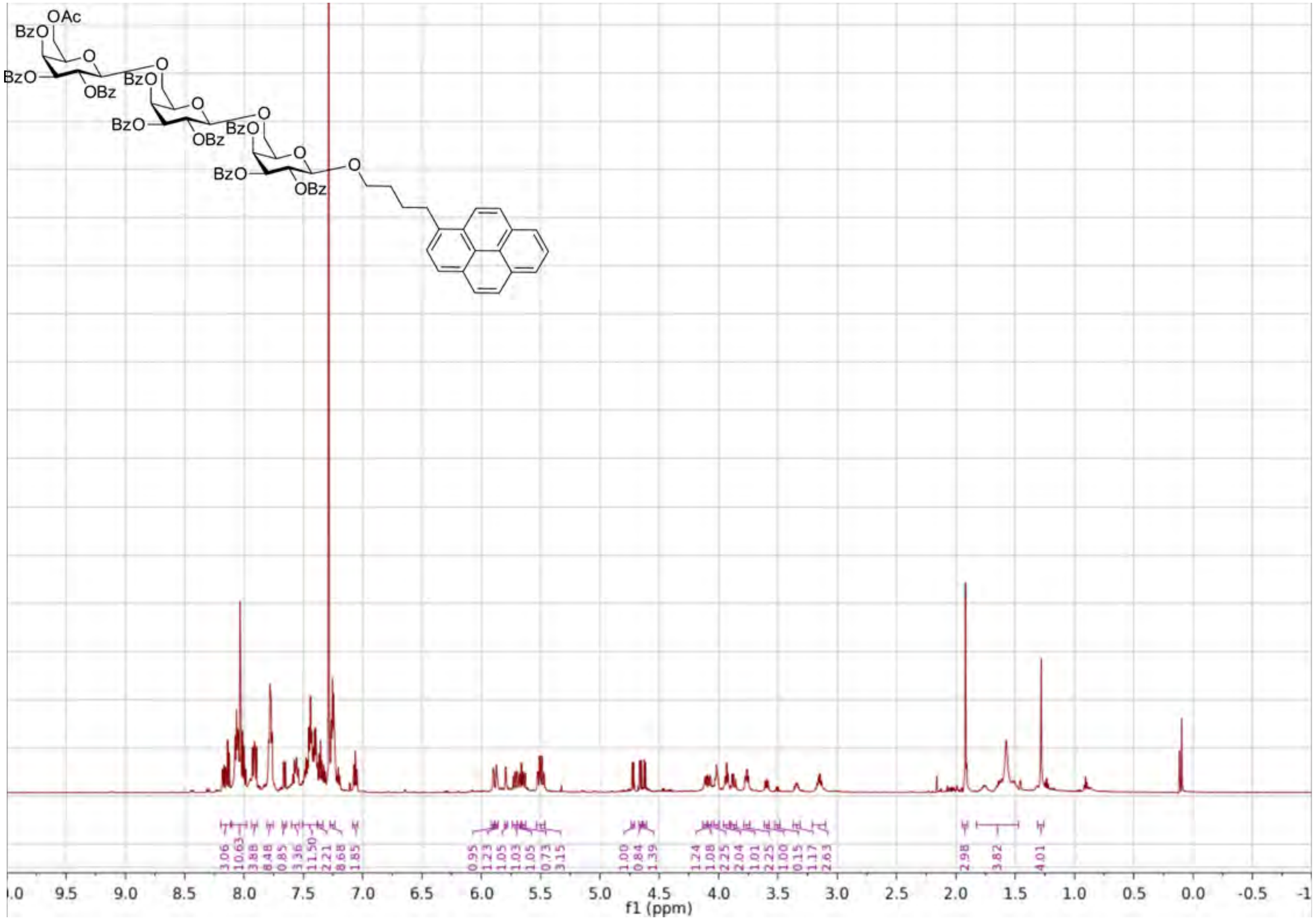

COSY

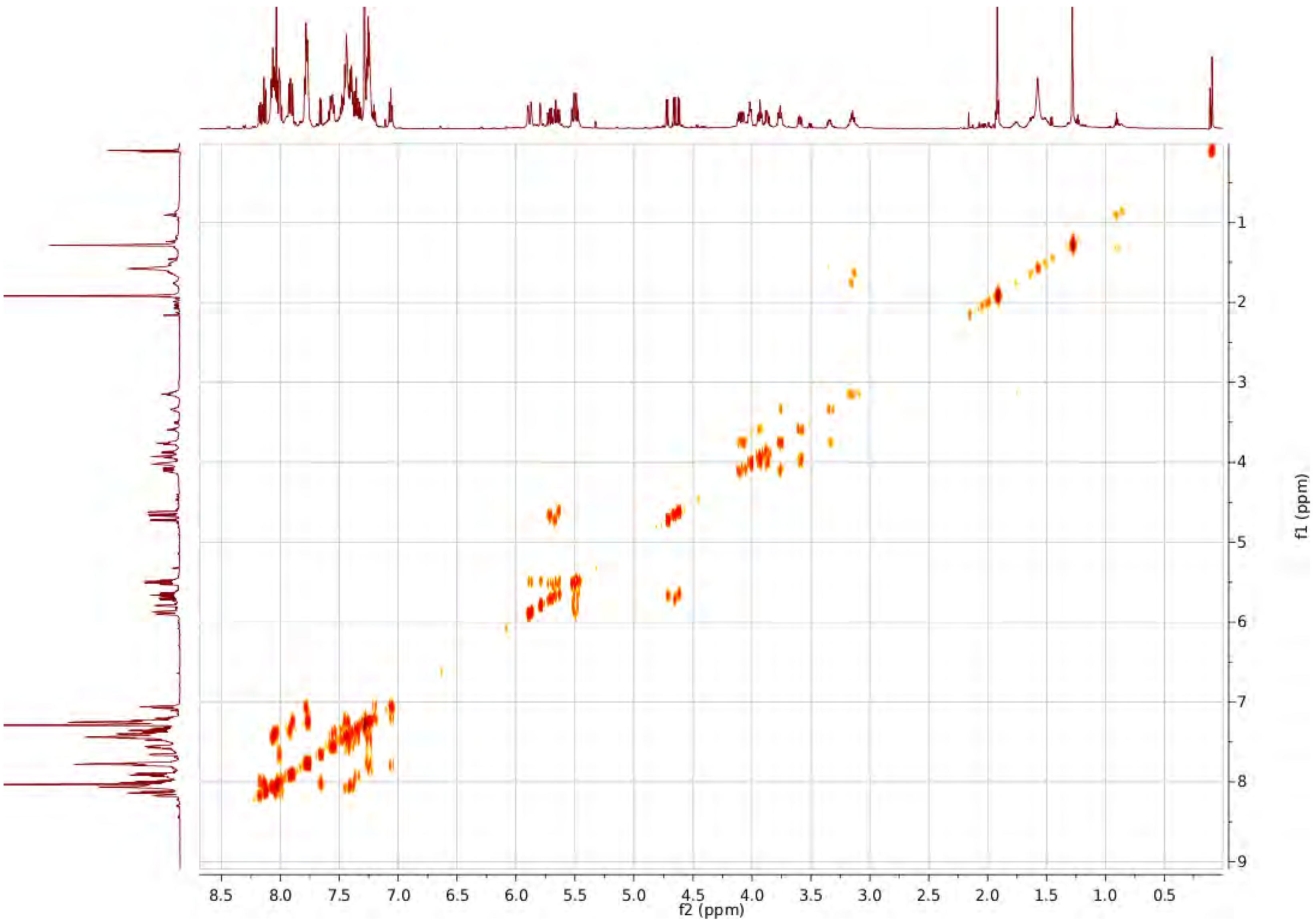

Compound 4_1**b**

Proton

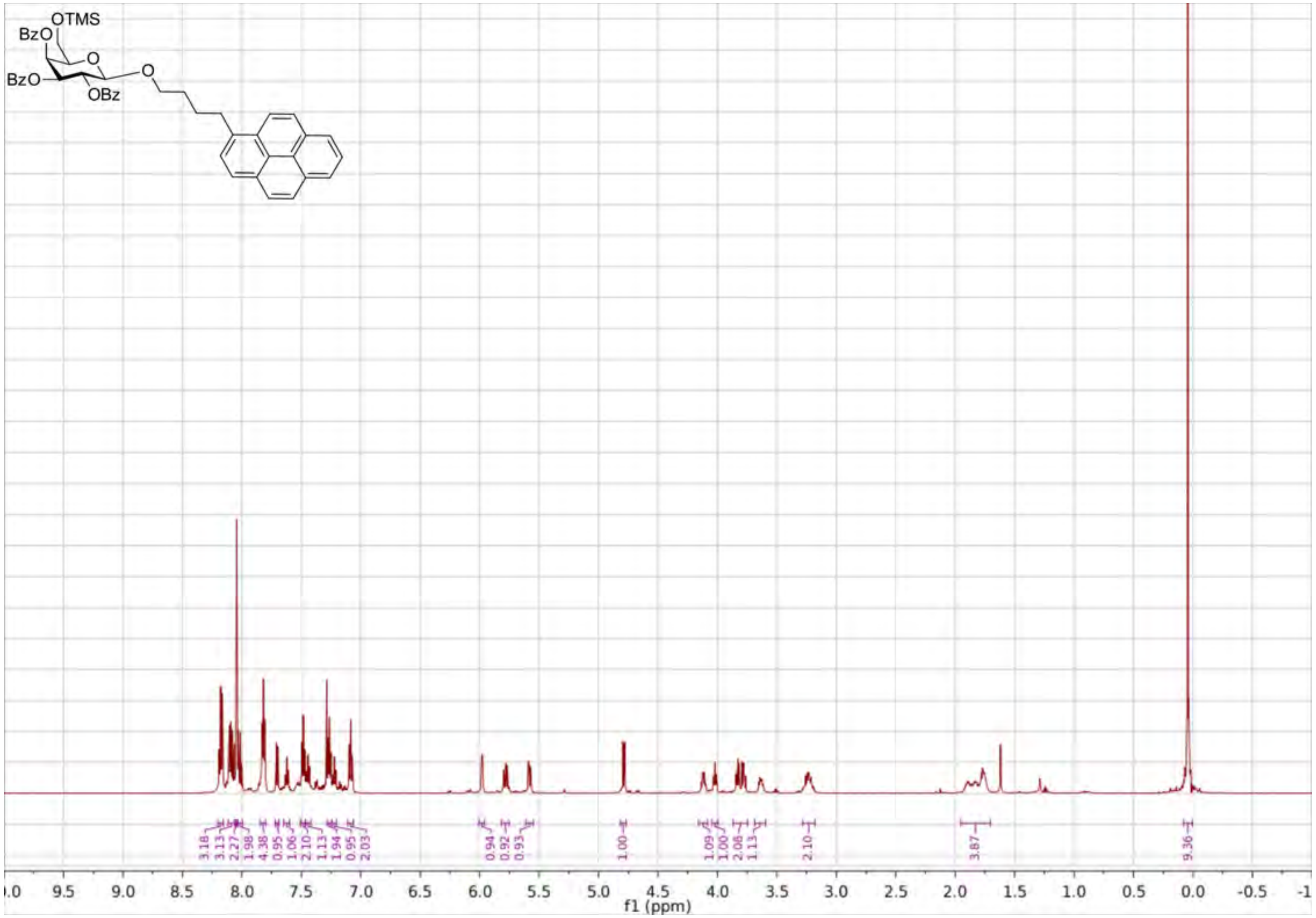

Carbon

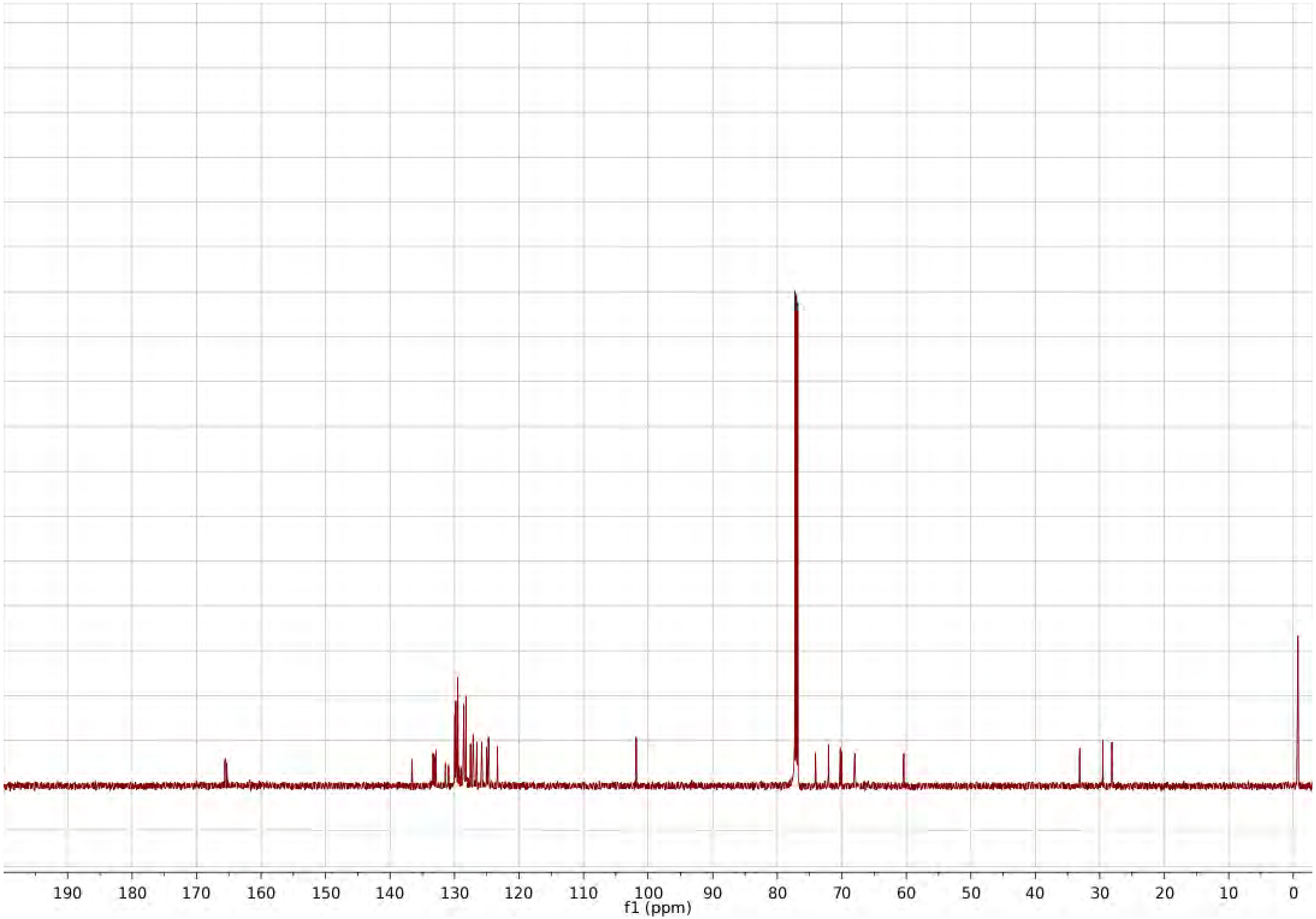

190
180
170
160
150
140
130
120
110
100
90
80
70
60
50
40
30
20
10
0
f1 (ppm)

COSY

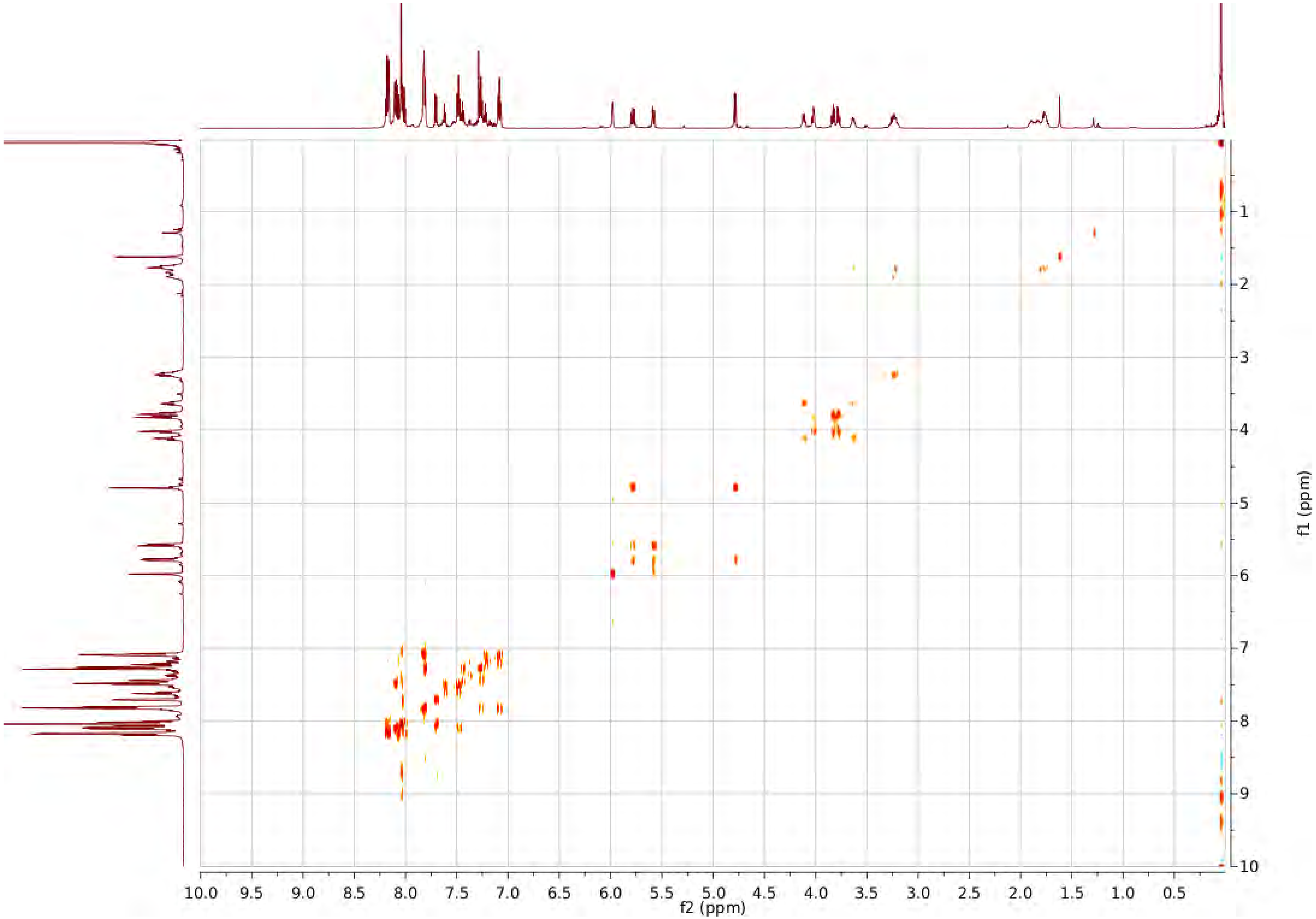

HSQC

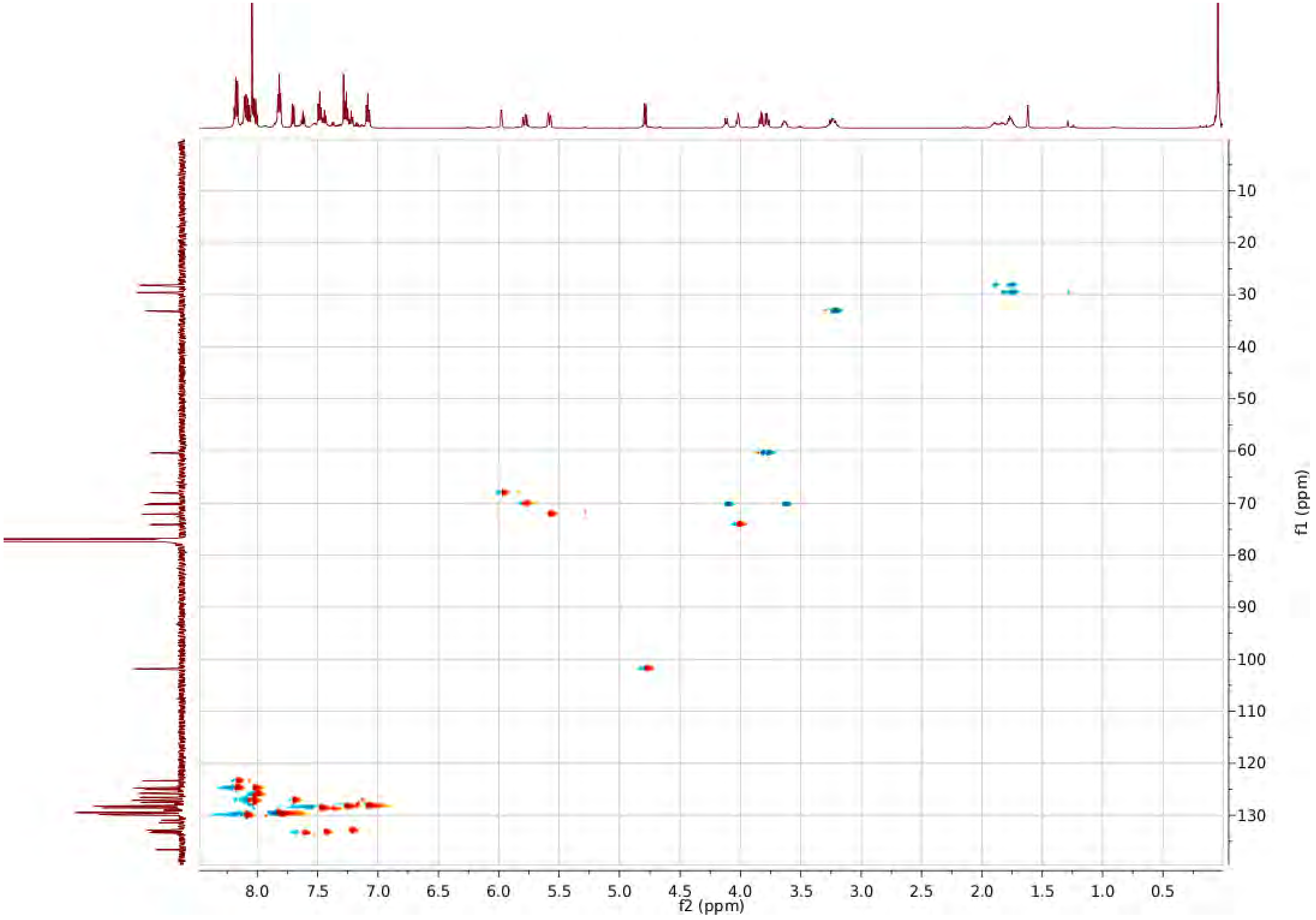

f2 (ppm)
f1 (ppm)
8.0
7.5
7.0
6.5
6.0
5.5
5.0
4.5
4.0
3.5
3.0
2.5
2.0
1.5
1.0
0.5
10
20
30
40
50
60
70
80
90
100
110
120
130

Compound 4_2b

Proton

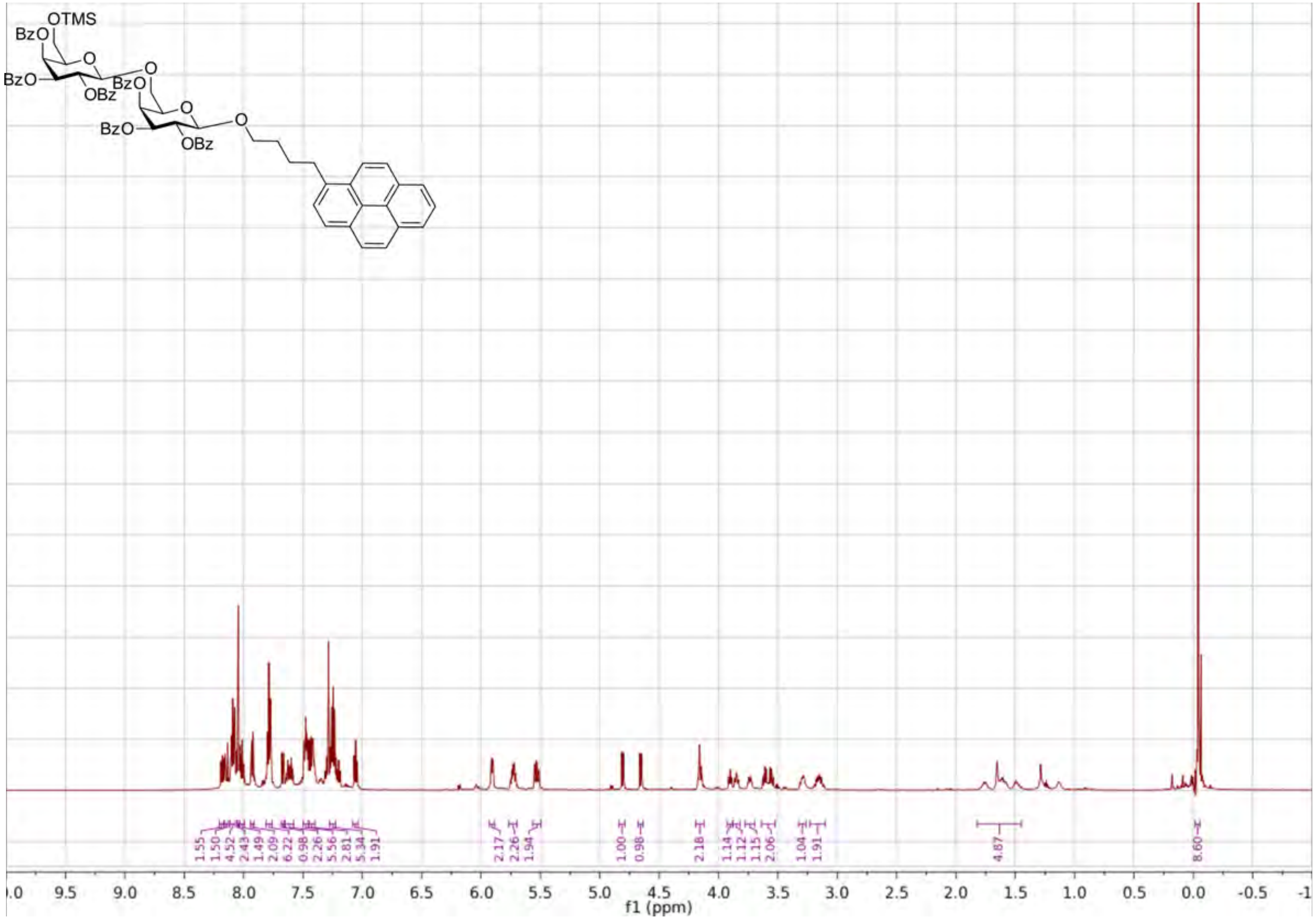

Carbon

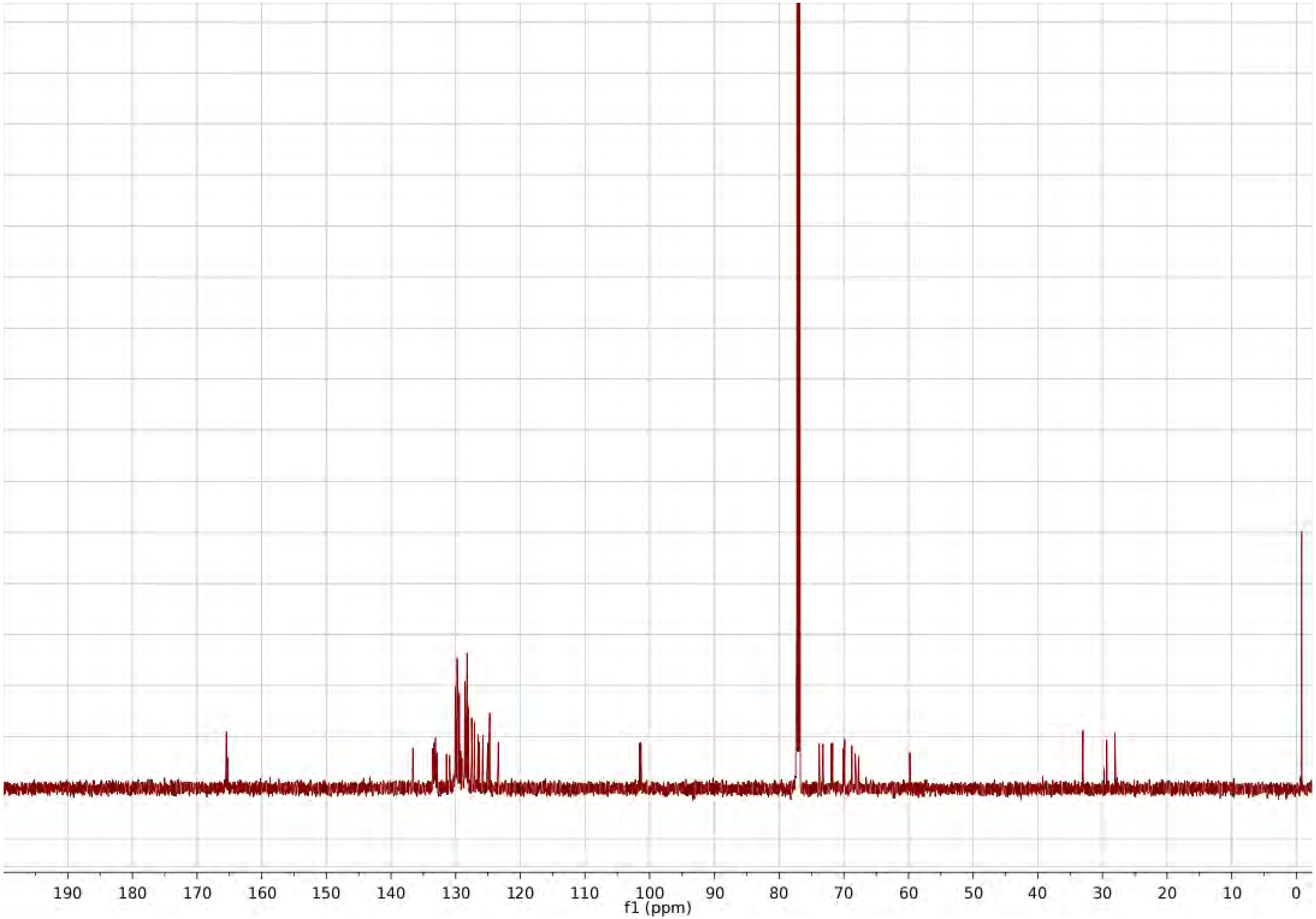

190
180
170
160
150
140
130
120
110
100
90
80
70
60
50
40
30
20
10
0
f1 (ppm)

COSY

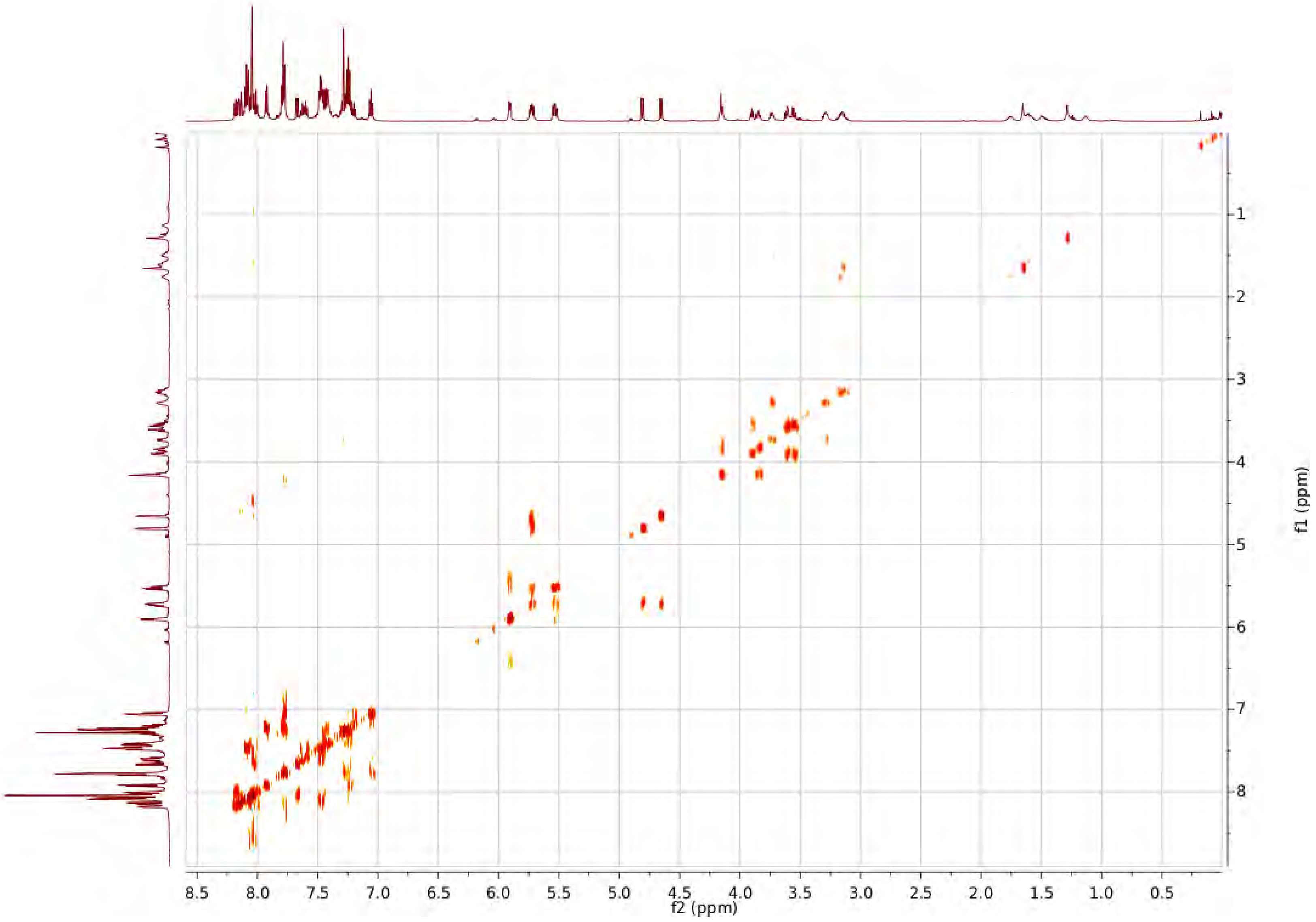
8.5
8.0
7.5
7.0
6.5
6.0
5.5
5.0
4.5
4.0
3.5
3.0
2.5
2.0
1.5
1.0
0.5
f2 (ppm)
1
2
3
4
5
6
7
8
f1 (ppm)

HSQC

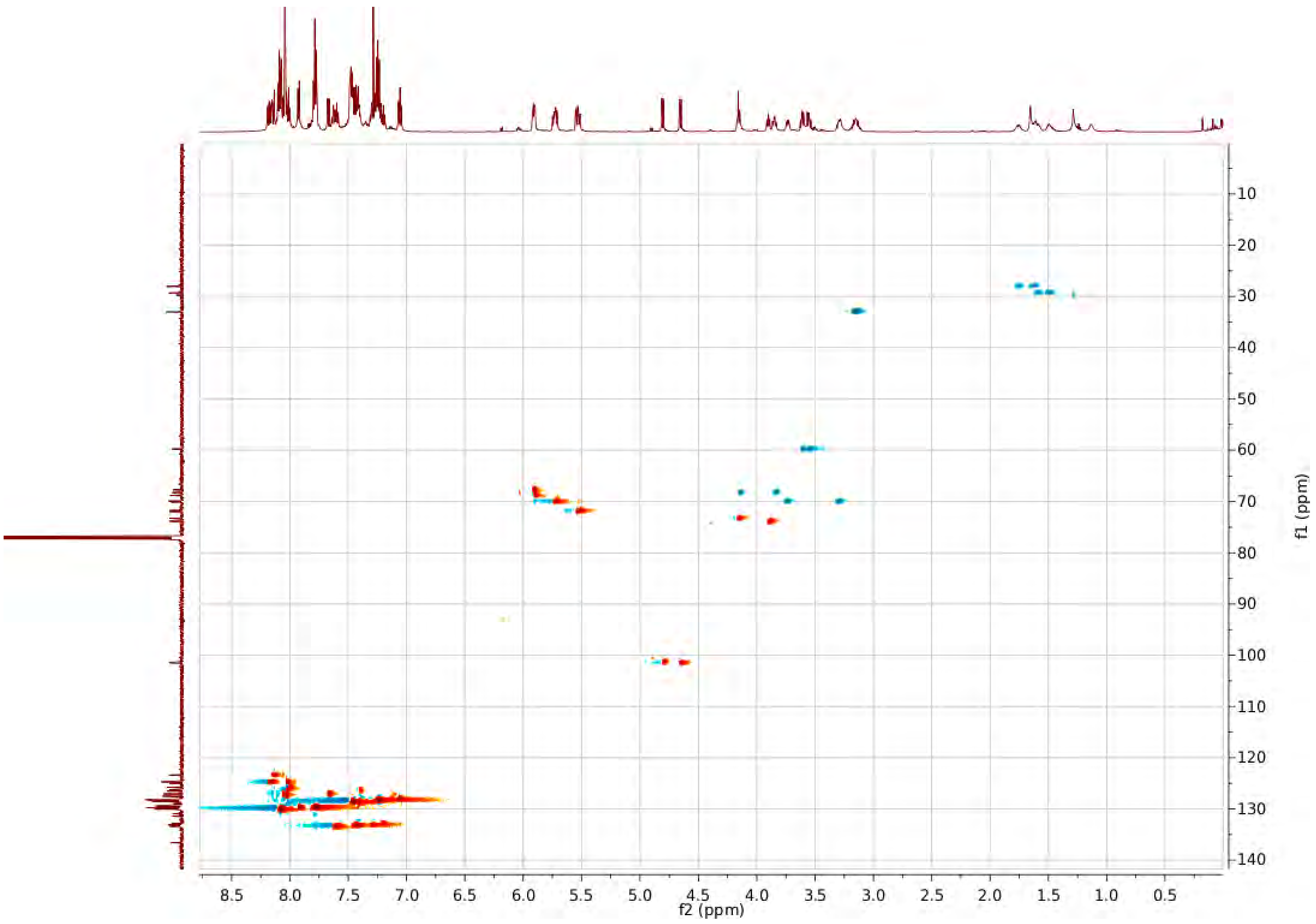

8.5 8.0 7.5 7.0 6.5 6.0 5.5 5.0 4.5 4.0 3.5 3.0 2.5 2.0 1.5 1.0 0.5
f2 (ppm)
10 20 30 40 50 60 70 80 90 100 110 120 130 140
f1 (ppm)

Compound **4₃b**

Proton

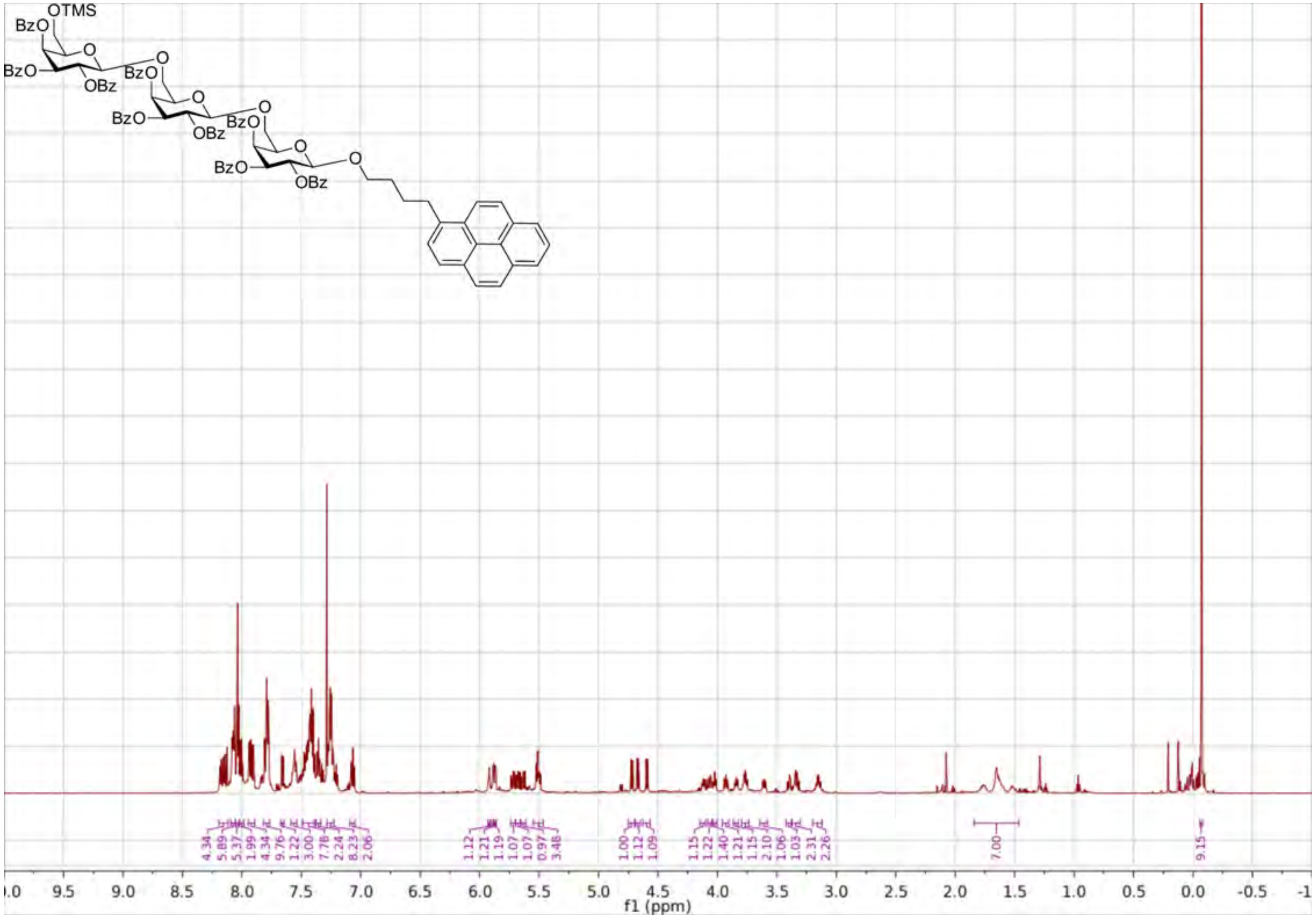

Carbon

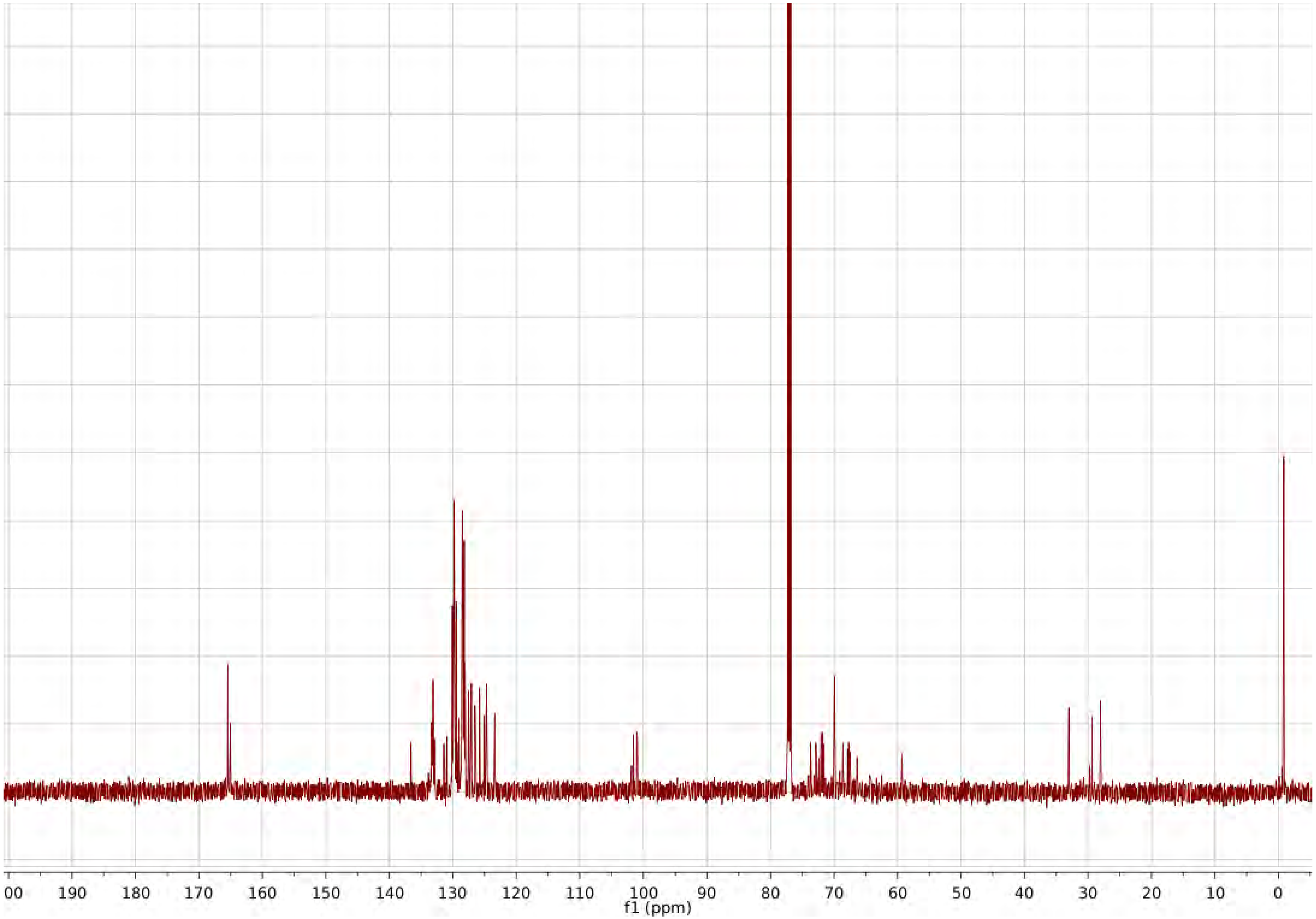

00
190
180
170
160
150
140
130
120
110
100
90
80
70
60
50
40
30
20
10
0
f1 (ppm)

COSY

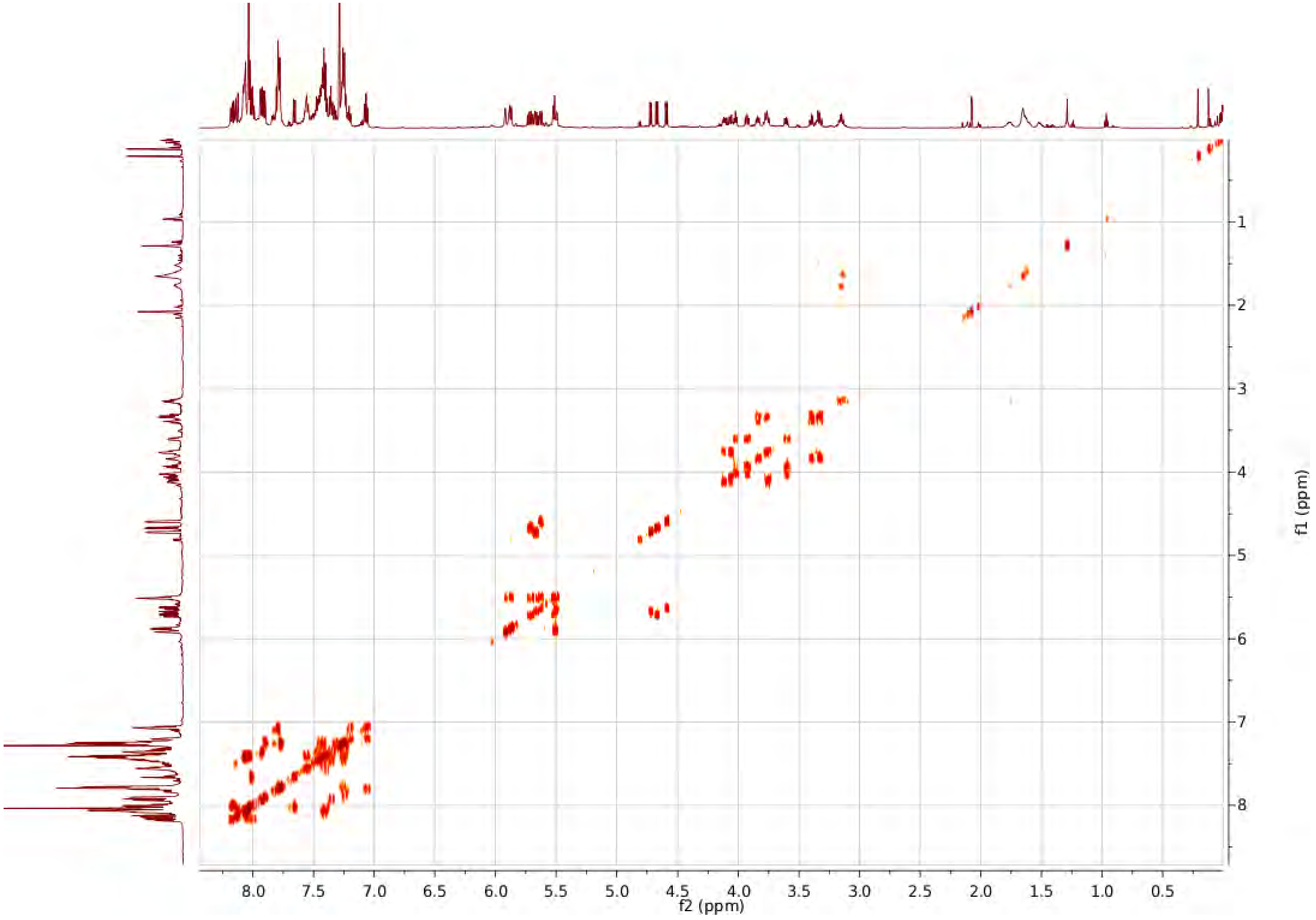
f1 (ppm)
f2 (ppm)
8.0
7.5
7.0
6.5
6.0
5.5
5.0
4.5
4.0
3.5
3.0
2.5
2.0
1.5
1.0
0.5
1
2
3
4
5
6
7
8

HSQC

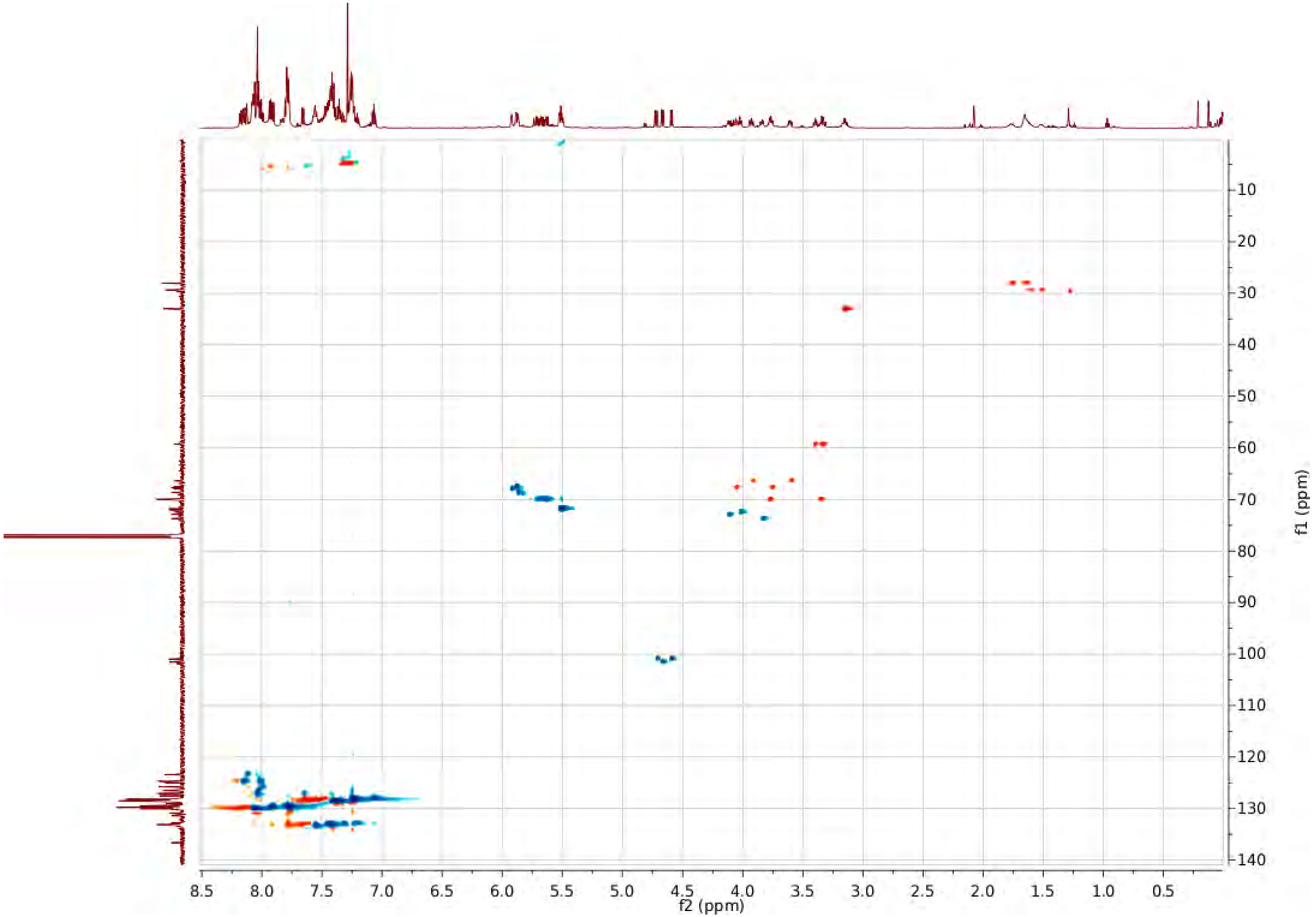

8.5
8.0
7.5
7.0
6.5
6.0
5.5
5.0
4.5
4.0
3.5
3.0
2.5
2.0
1.5
1.0
0.5
f2 (ppm)
10
20
30
40
50
60
70
80
90
100
110
120
130
140
f1 (ppm)

Compound 4_4b

Proton

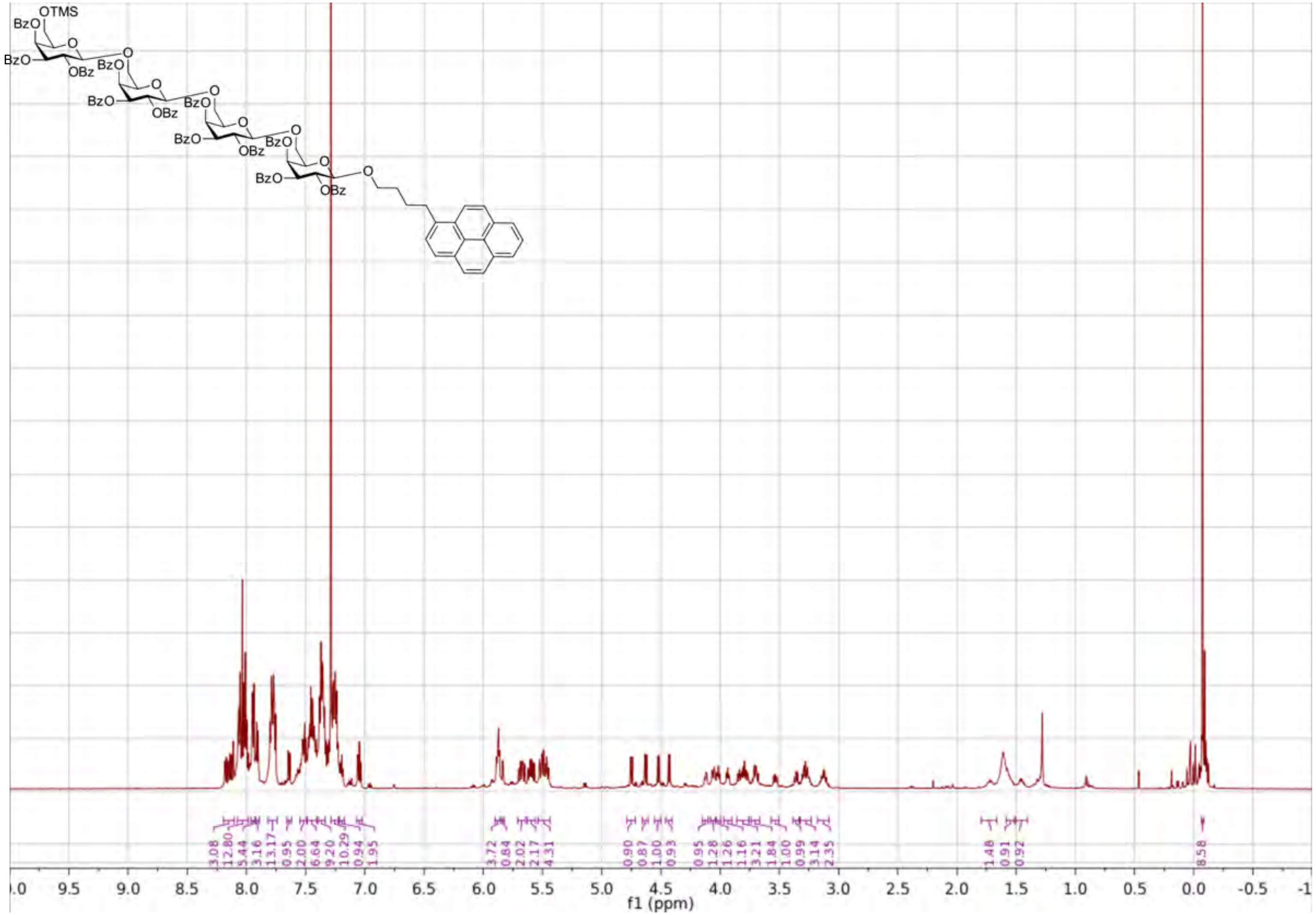

Carbon

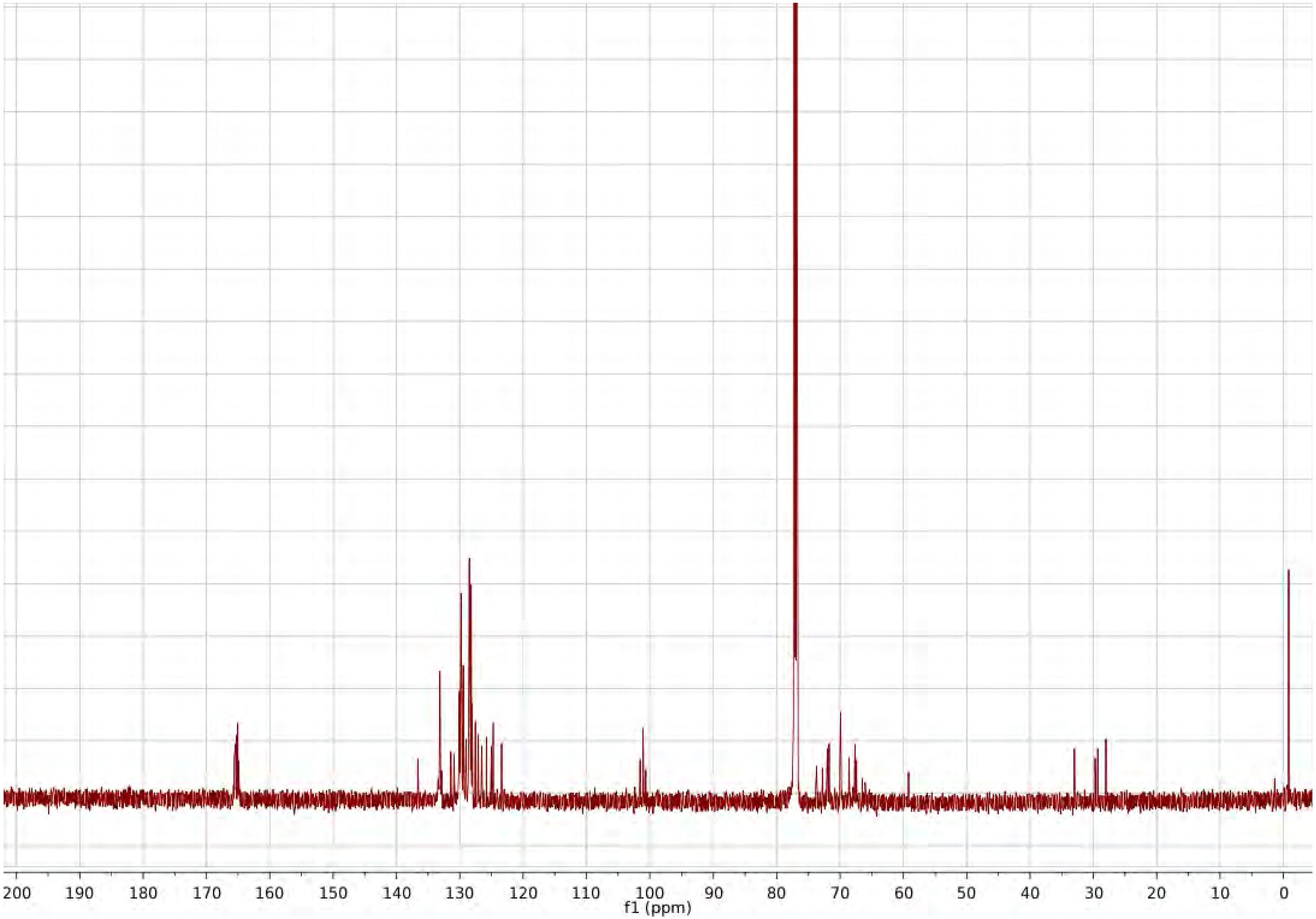
200
190
180
170
160
150
140
130
120
110
100
90
80
70
60
50
40
30
20
10
0
f1 (ppm)

COSY

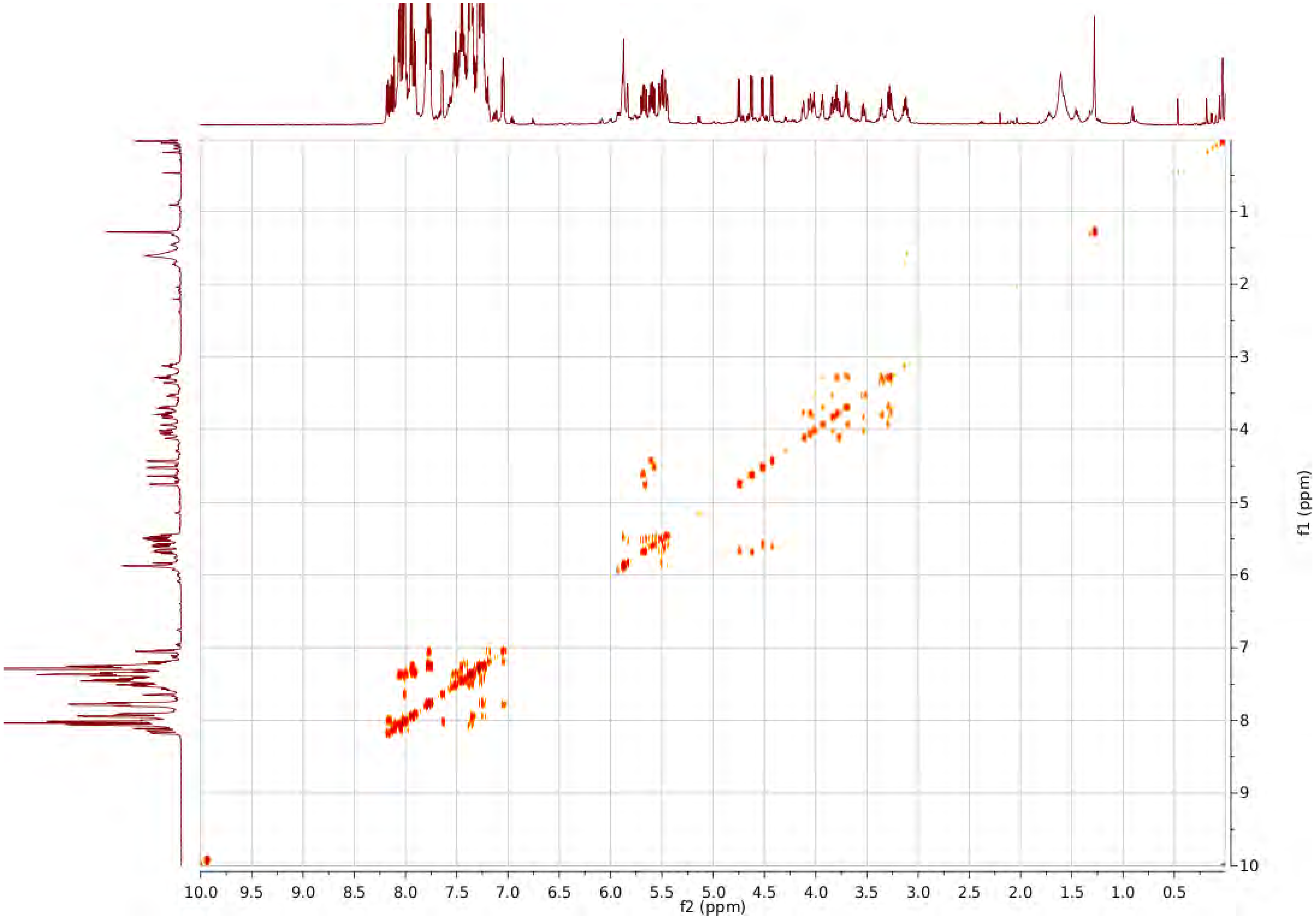

10.0
9.5
9.0
8.5
8.0
7.5
7.0
6.5
6.0
5.5
5.0
4.5
4.0
3.5
3.0
2.5
2.0
1.5
1.0
0.5
f2 (ppm)
1
2
3
4
5
6
7
8
9
10
f1 (ppm)

HSQC

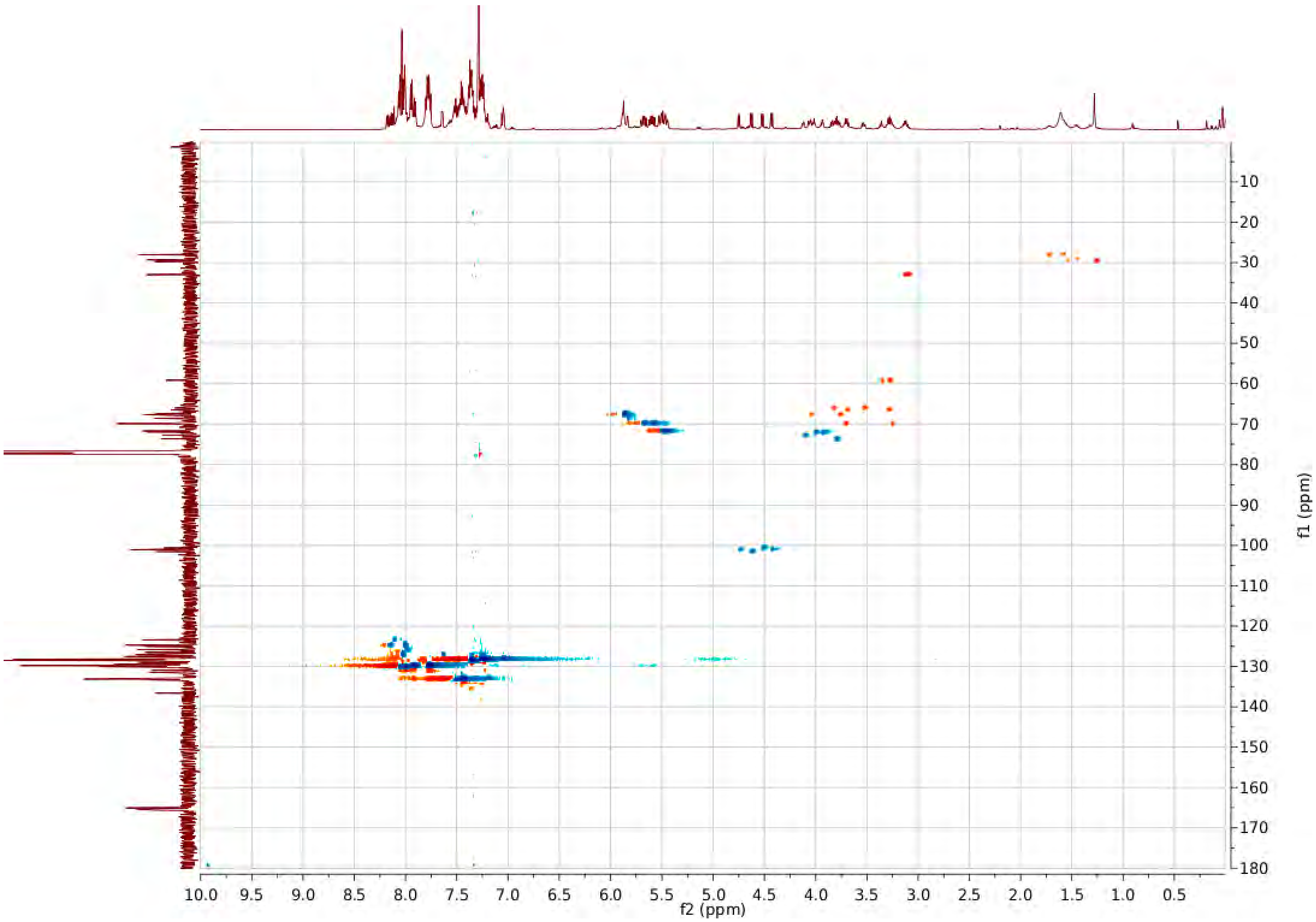

Compound $\mathbf{4_1c}$

Proton

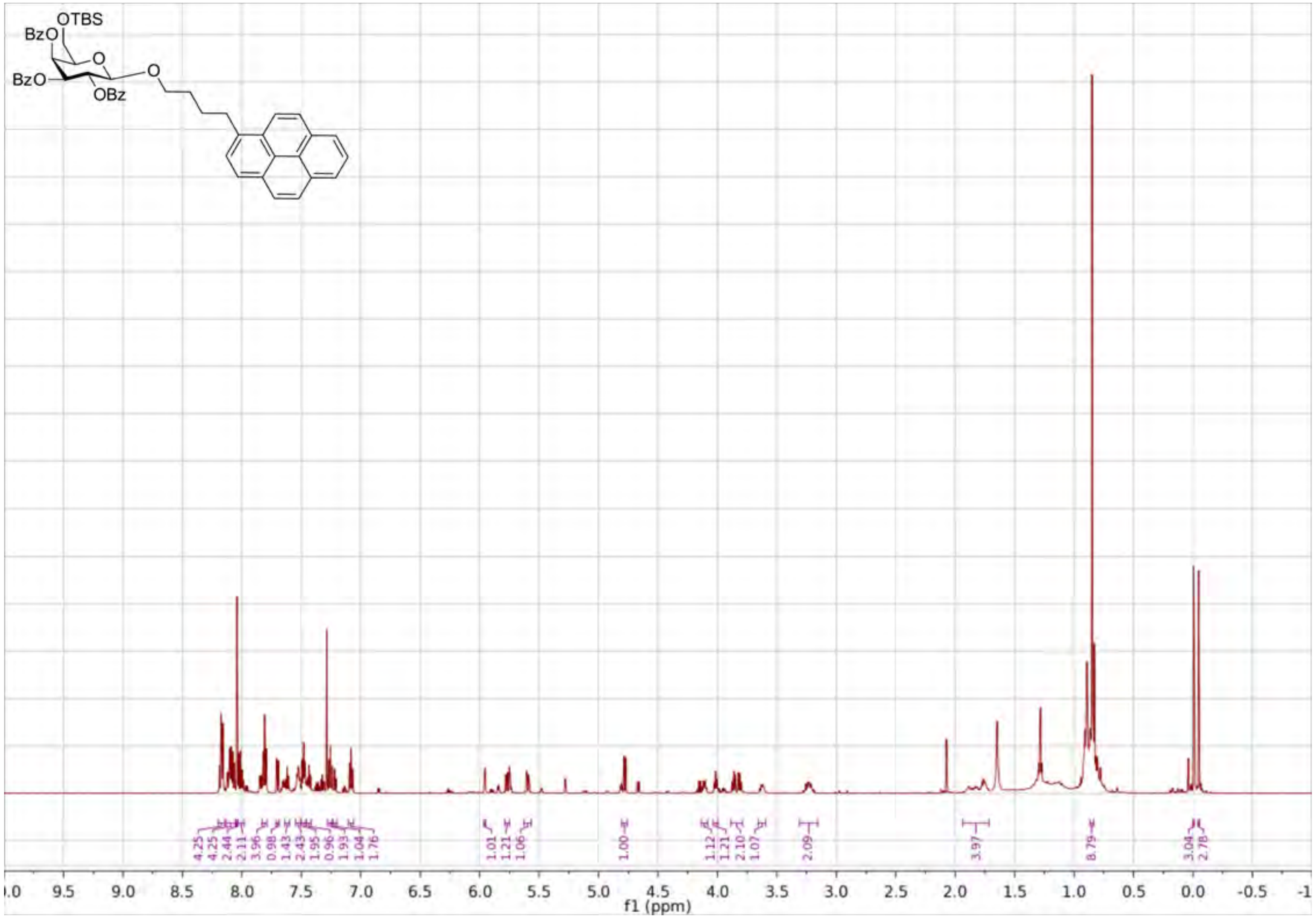

Carbon

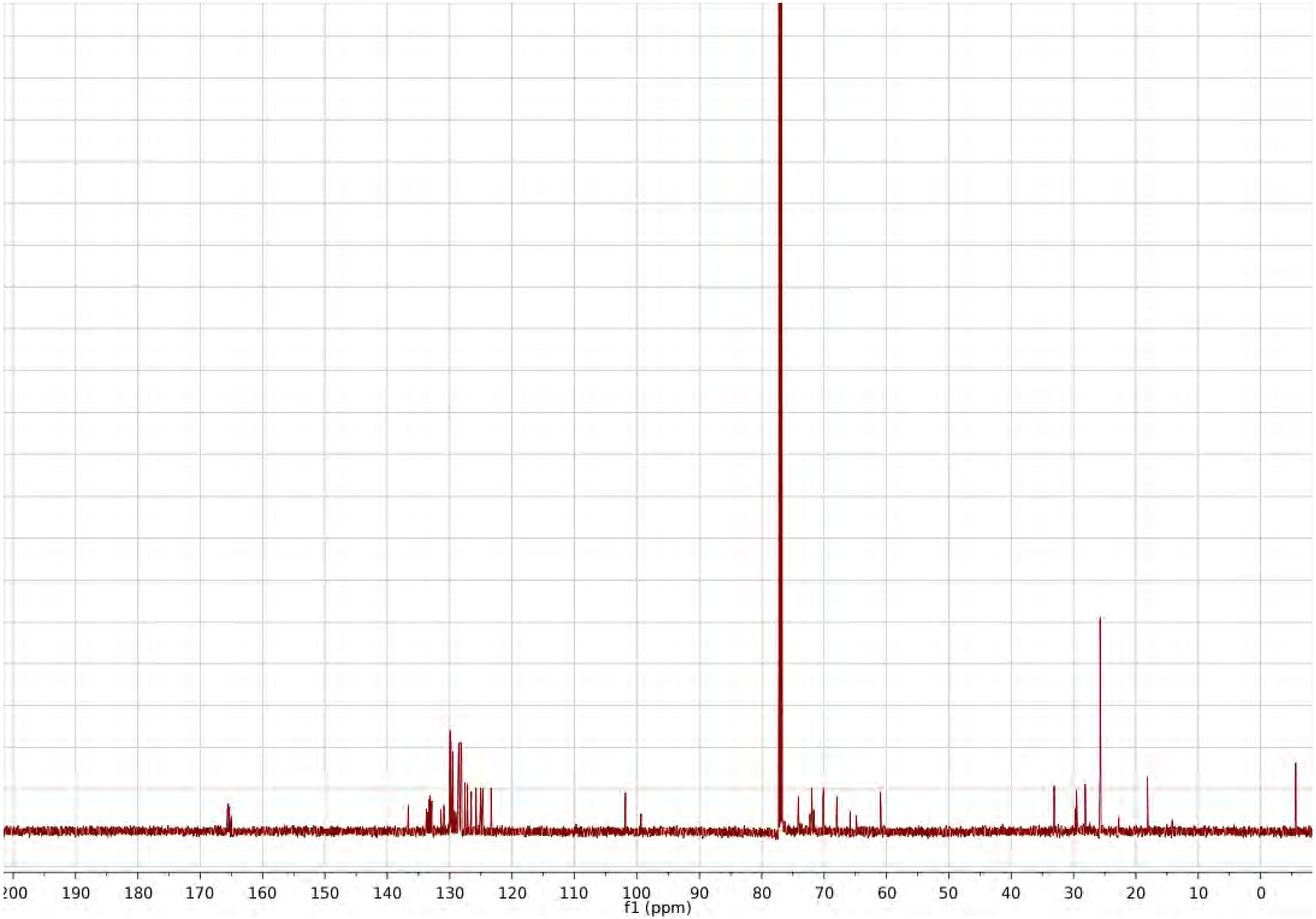

200
190
180
170
160
150
140
130
120
110
100
90
80
70
60
50
40
30
20
10
0
f1 (ppm)

Compound $\mathbf{4_2c}$

Proton

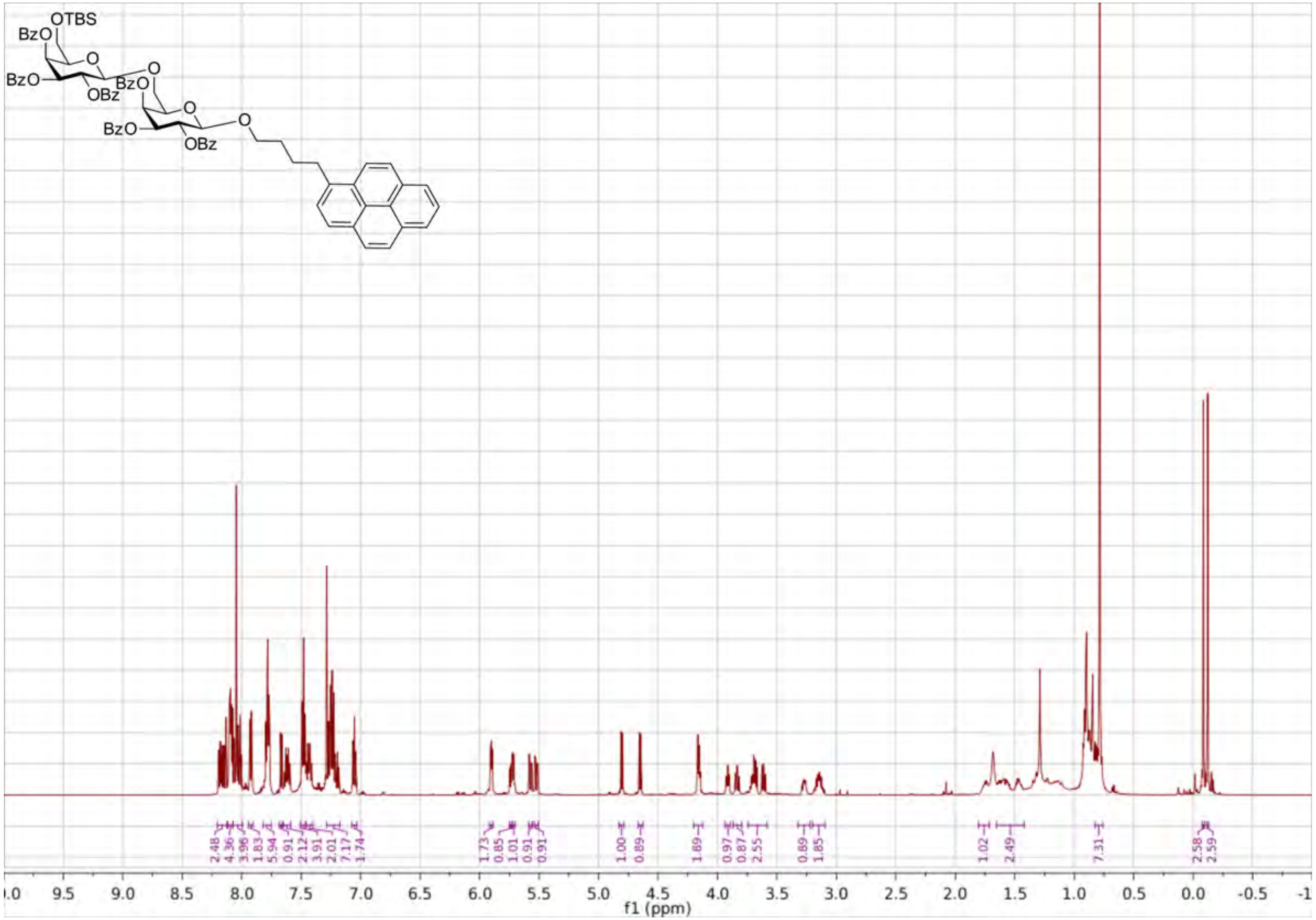

Carbon

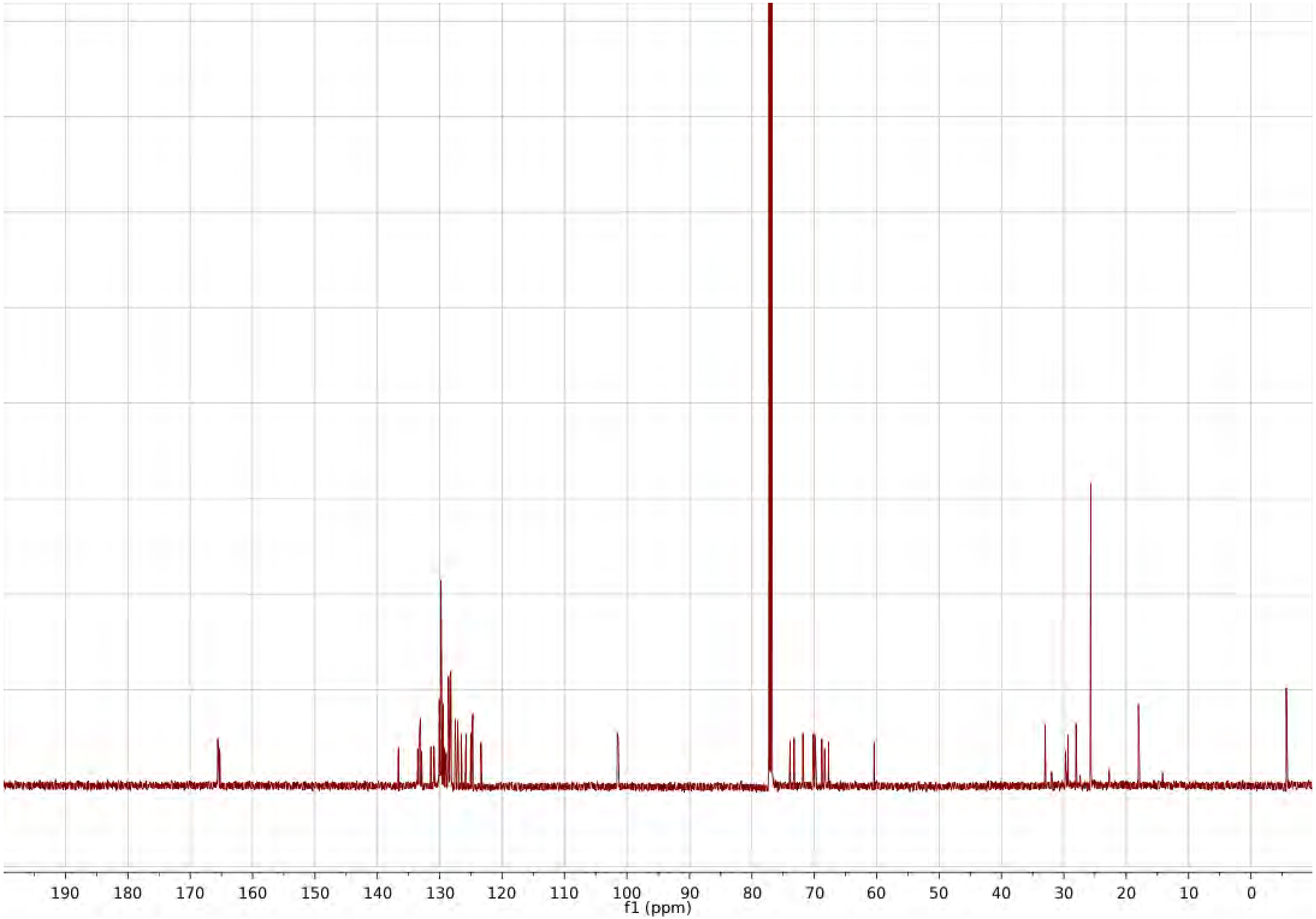

190
180
170
160
150
140
130
120
110
100
90
80
70
60
50
40
30
20
10
0
f1 (ppm)

COSY

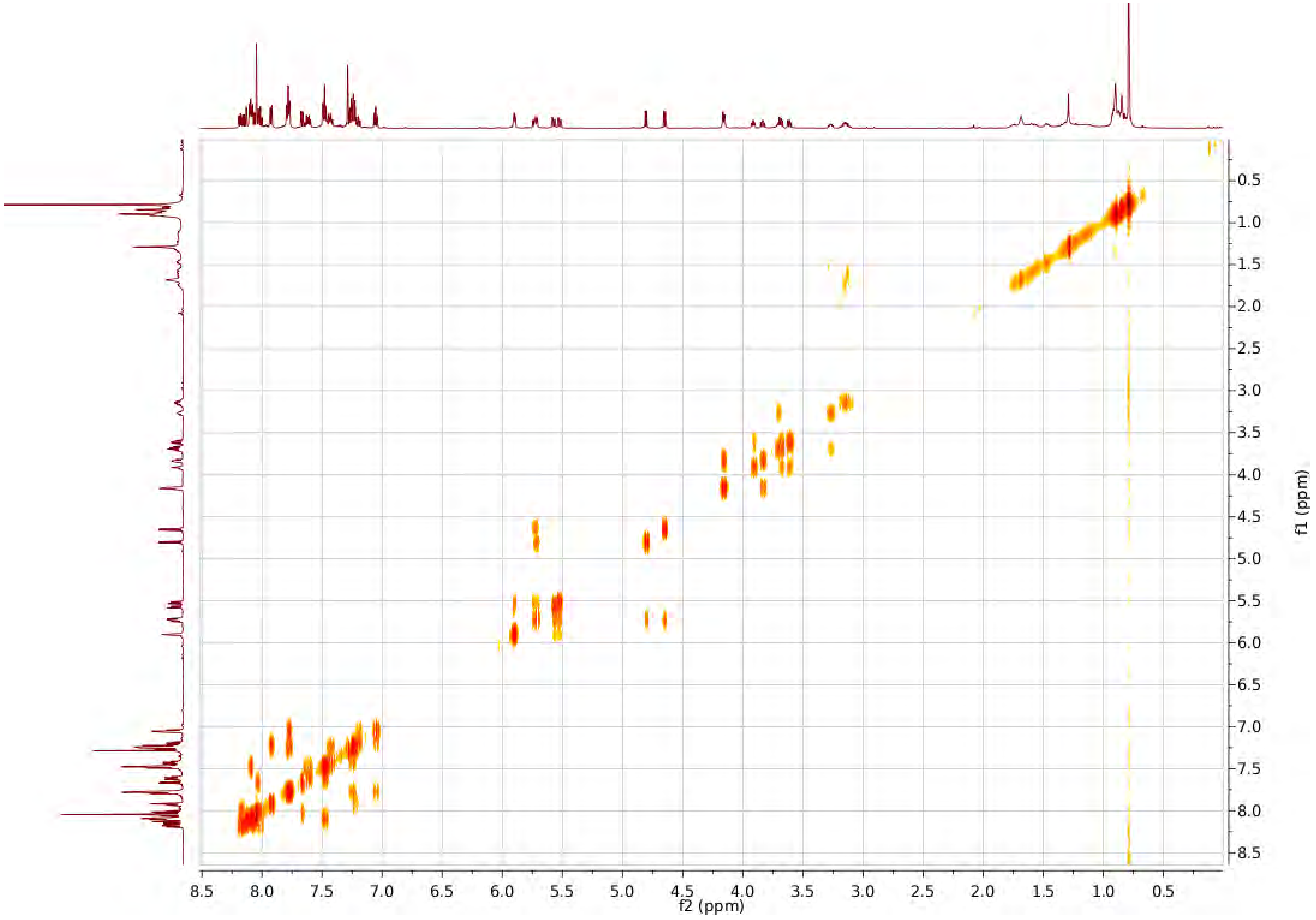

HSQC

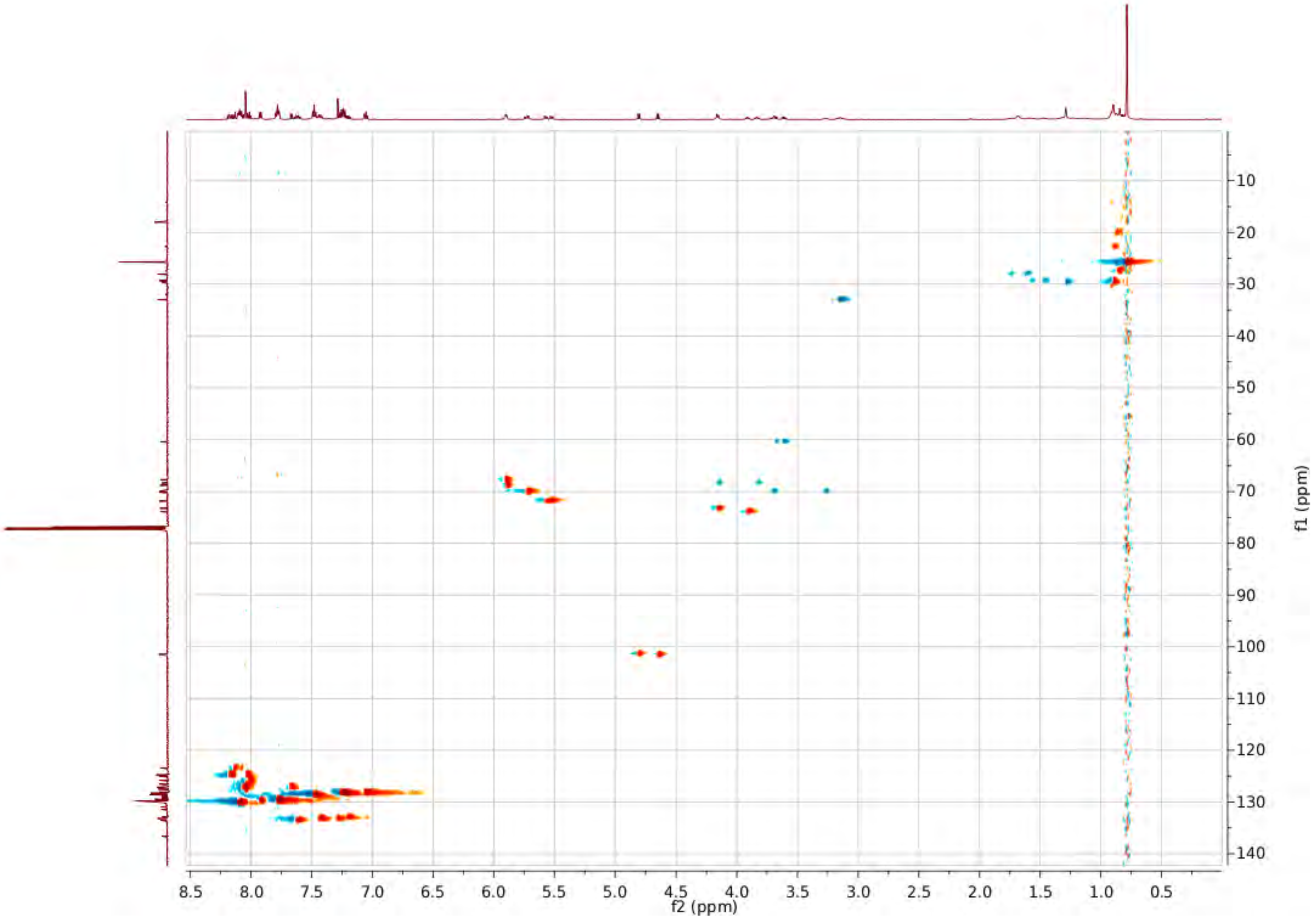

f2 (ppm)
f1 (ppm)
8.5
8.0
7.5
7.0
6.5
6.0
5.5
5.0
4.5
4.0
3.5
3.0
2.5
2.0
1.5
1.0
0.5
10
20
30
40
50
60
70
80
90
100
110
120
130
140

Compound **4₃c**

Proton

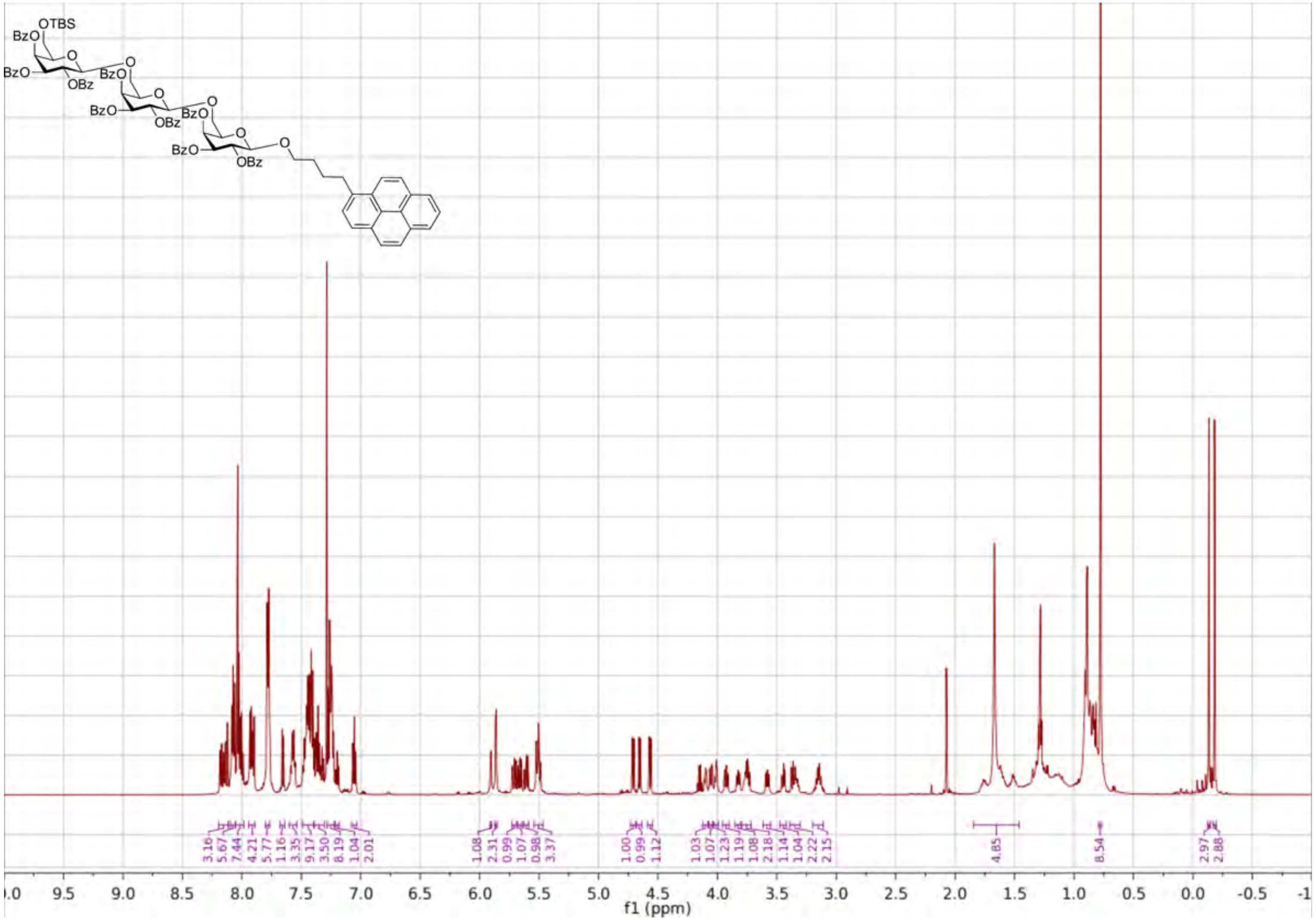

Carbon

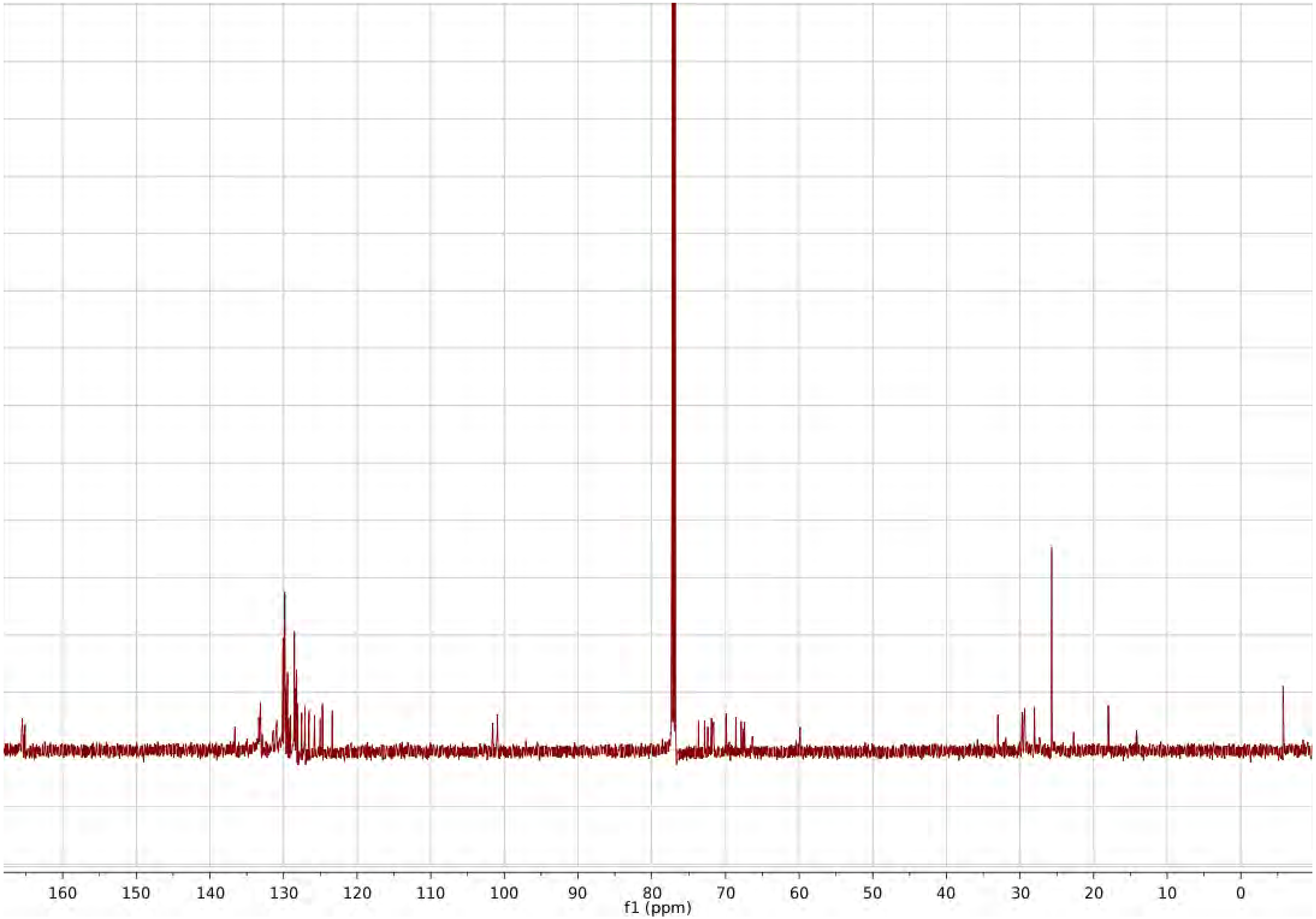

COSY

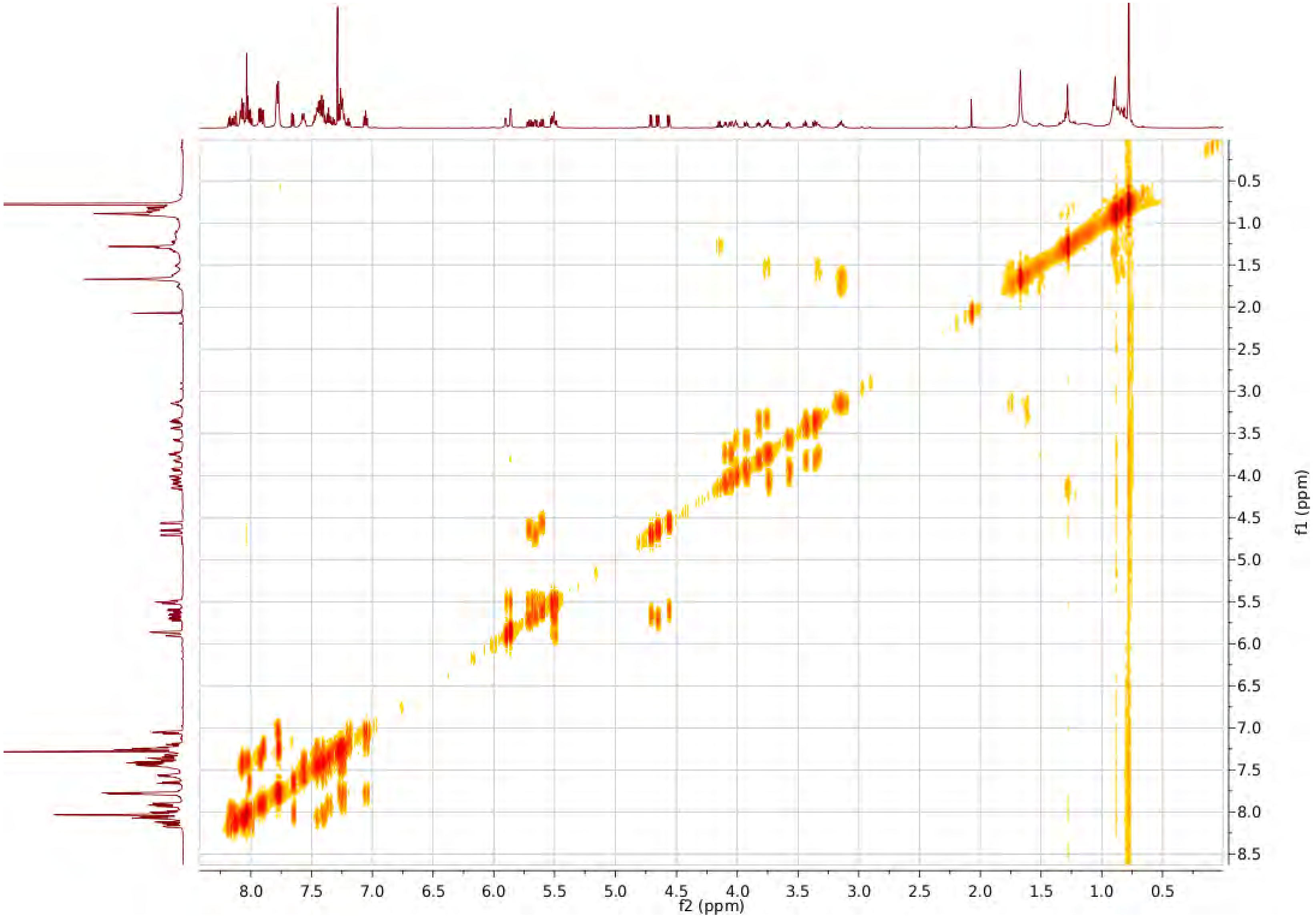

HSQC

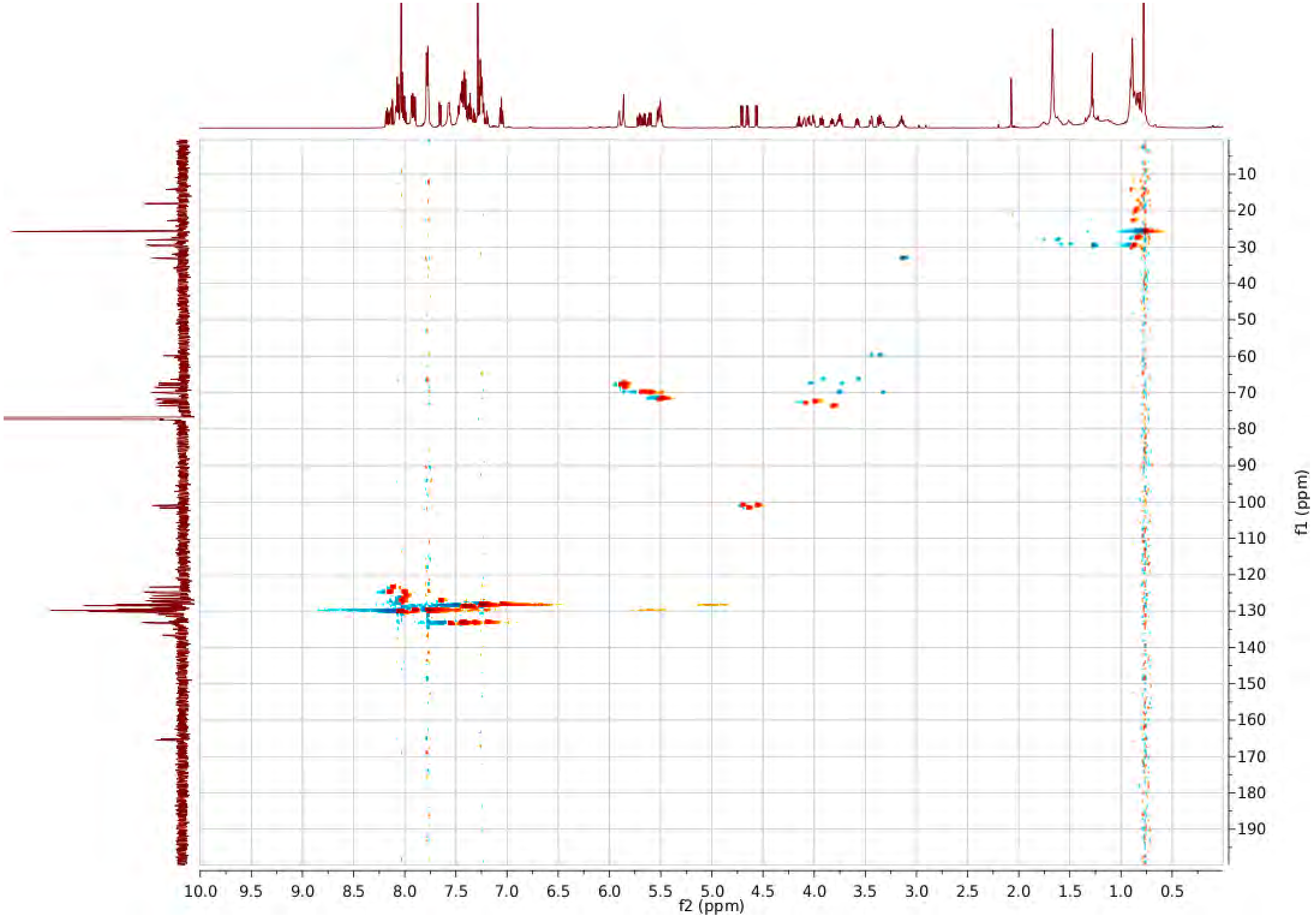

10.0 9.5 9.0 8.5 8.0 7.5 7.0 6.5 6.0 5.5 5.0 4.5 4.0 3.5 3.0 2.5 2.0 1.5 1.0 0.5
f2 (ppm)
10 20 30 40 50 60 70 80 90 100 110 120 130 140 150 160 170 180 190
f1 (ppm)

Compound **4₄c**

Proton

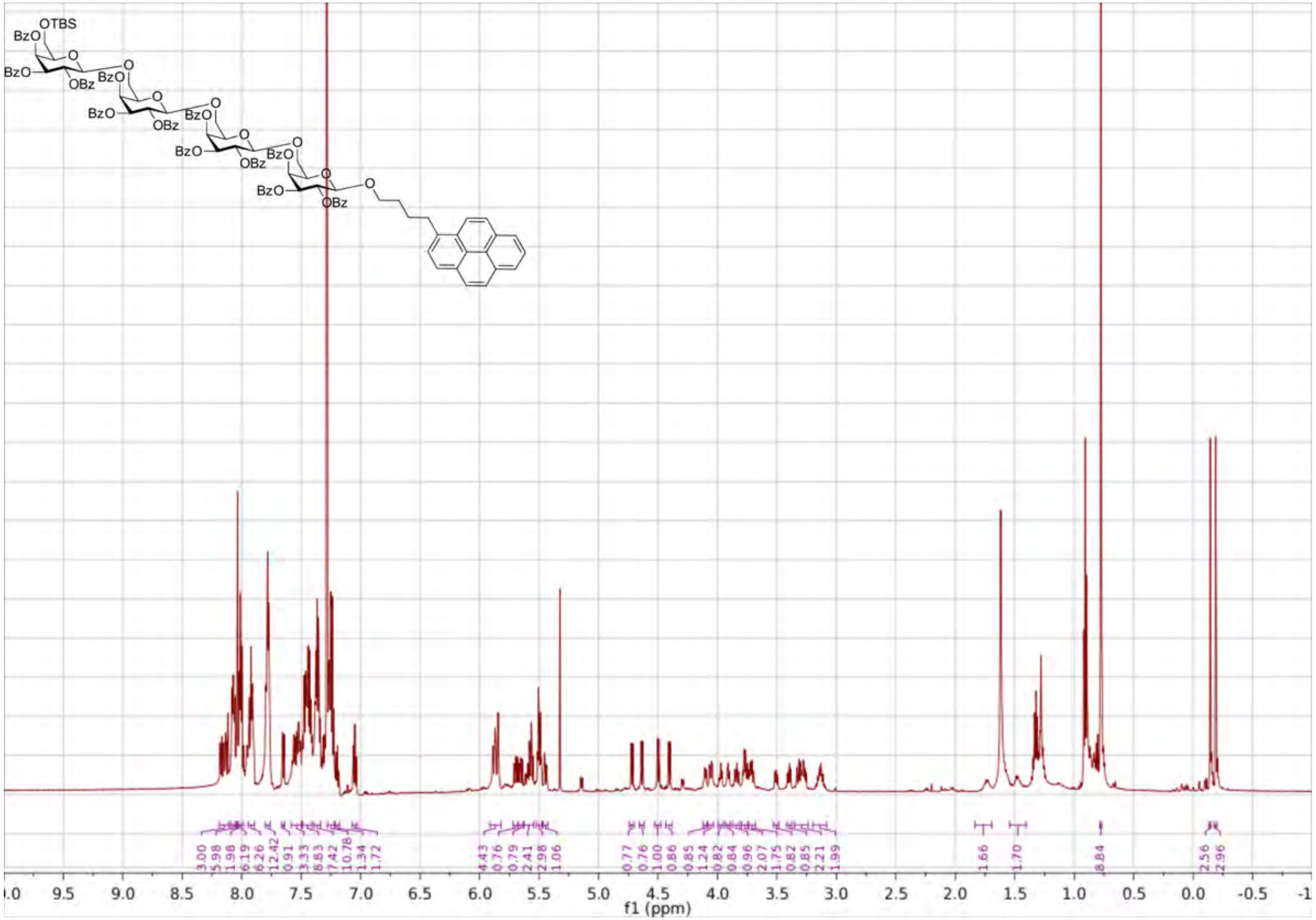

Carbon

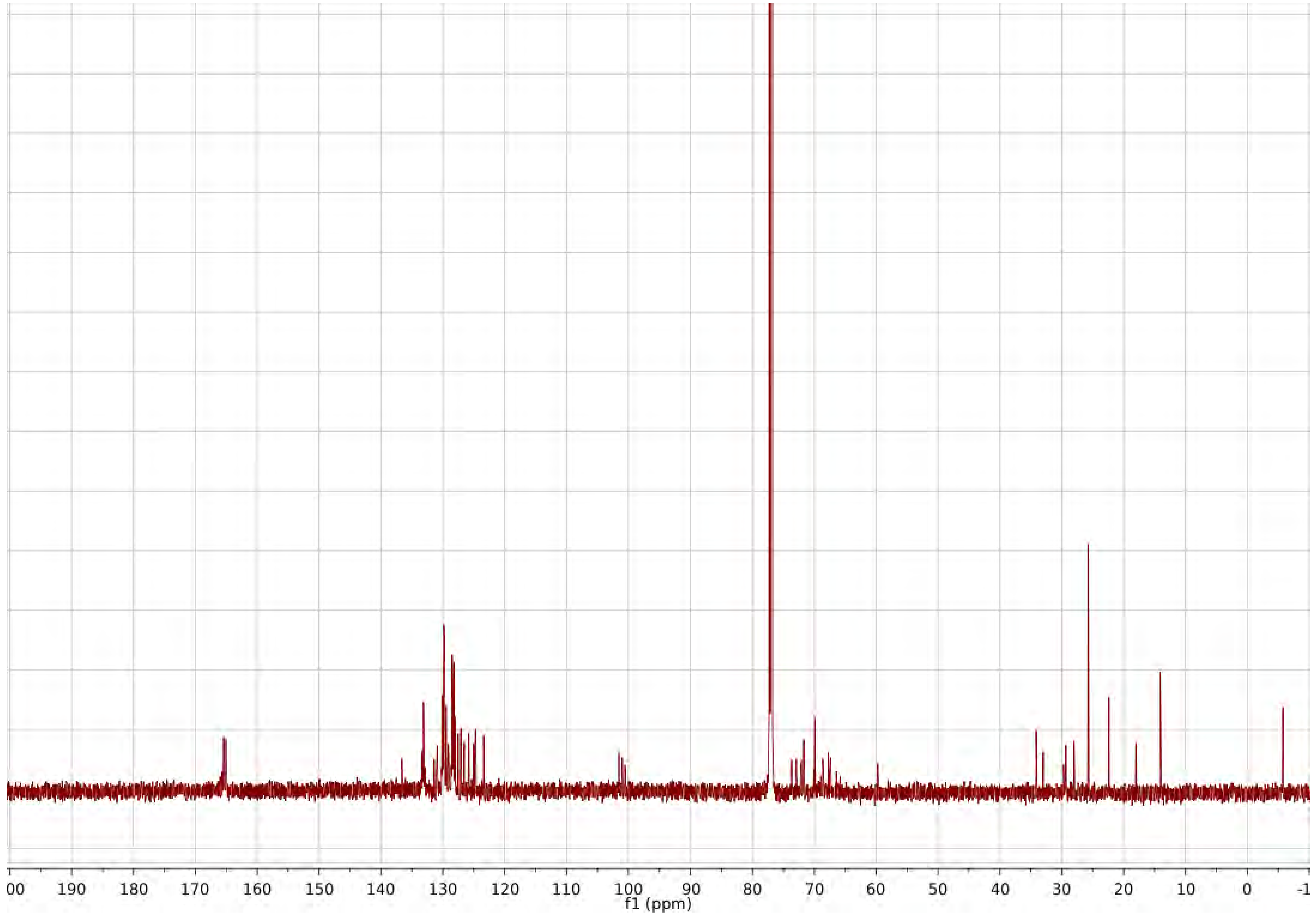

00
190
180
170
160
150
140
130
120
110
100
90
80
70
60
50
40
30
20
10
0
-1
f1 (ppm)

COSY

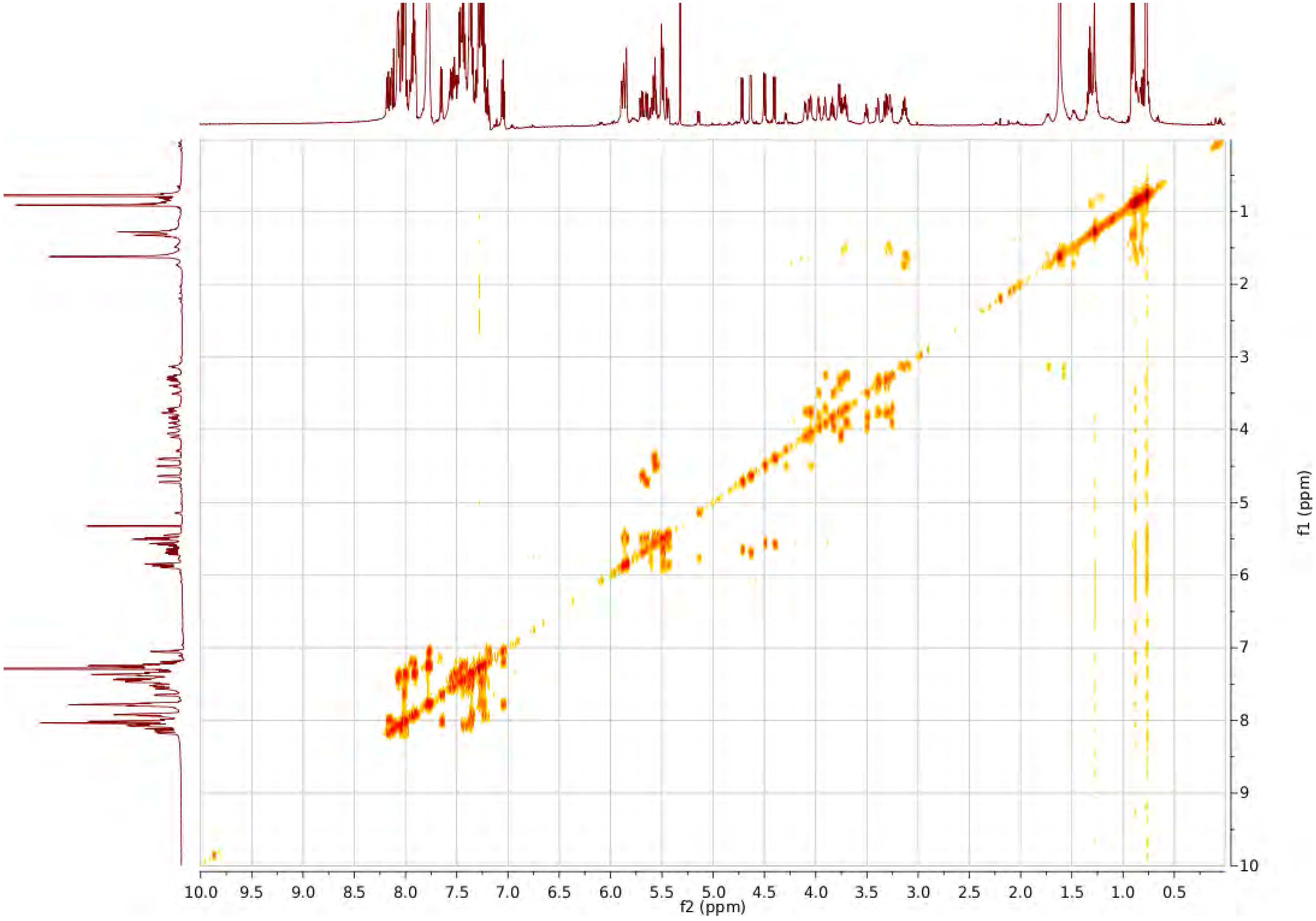

10.0
9.5
9.0
8.5
8.0
7.5
7.0
6.5
6.0
5.5
5.0
4.5
4.0
3.5
3.0
2.5
2.0
1.5
1.0
0.5
f2 (ppm)
1
2
3
4
5
6
7
8
9
10
f1 (ppm)

HSQC

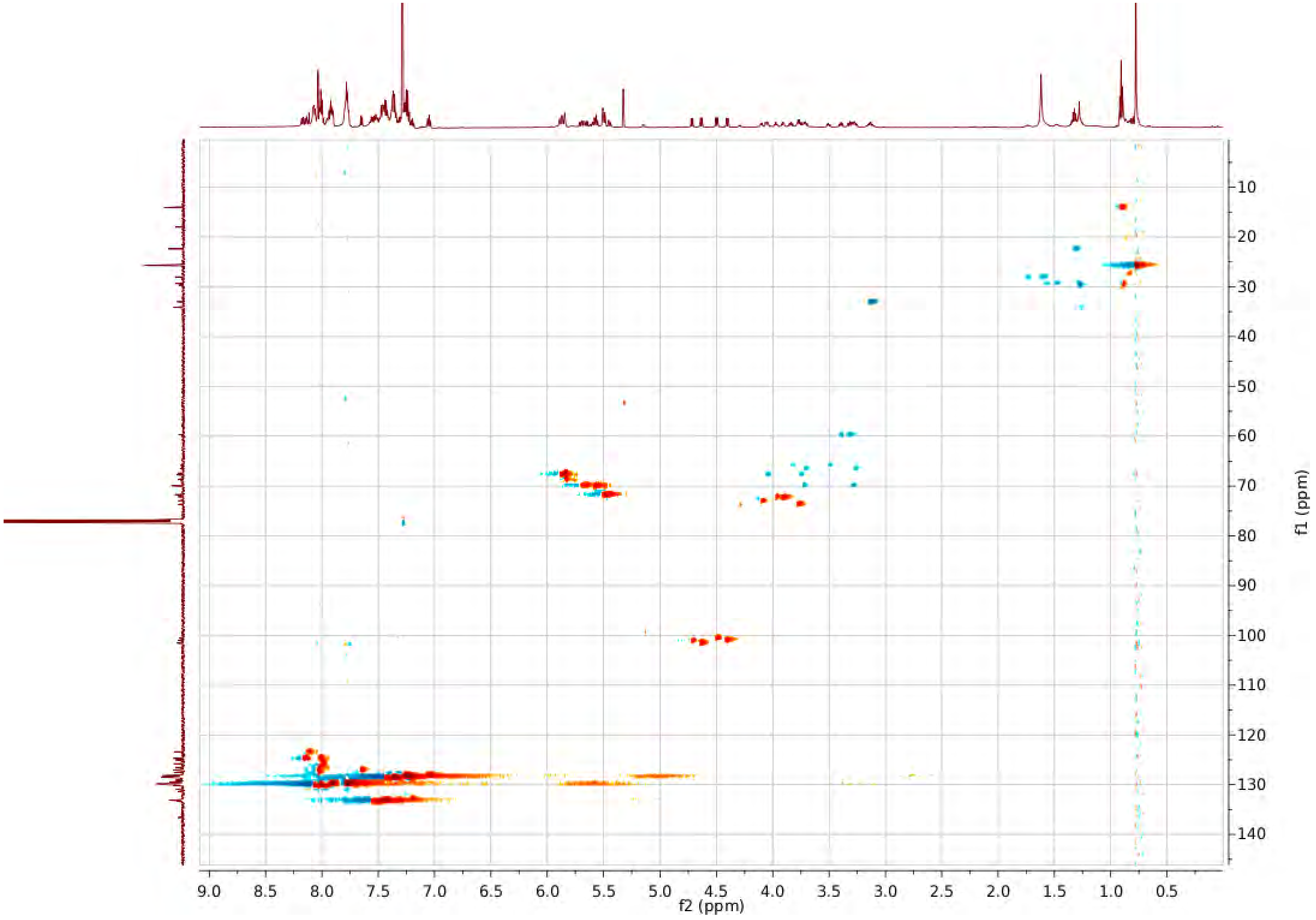

f2 (ppm)
f1 (ppm)
9.0 8.5 8.0 7.5 7.0 6.5 6.0 5.5 5.0 4.5 4.0 3.5 3.0 2.5 2.0 1.5 1.0 0.5
10 20 30 40 50 60 70 80 90 100 110 120 130 140

Compound $\mathbf{4_5c}$

Proton

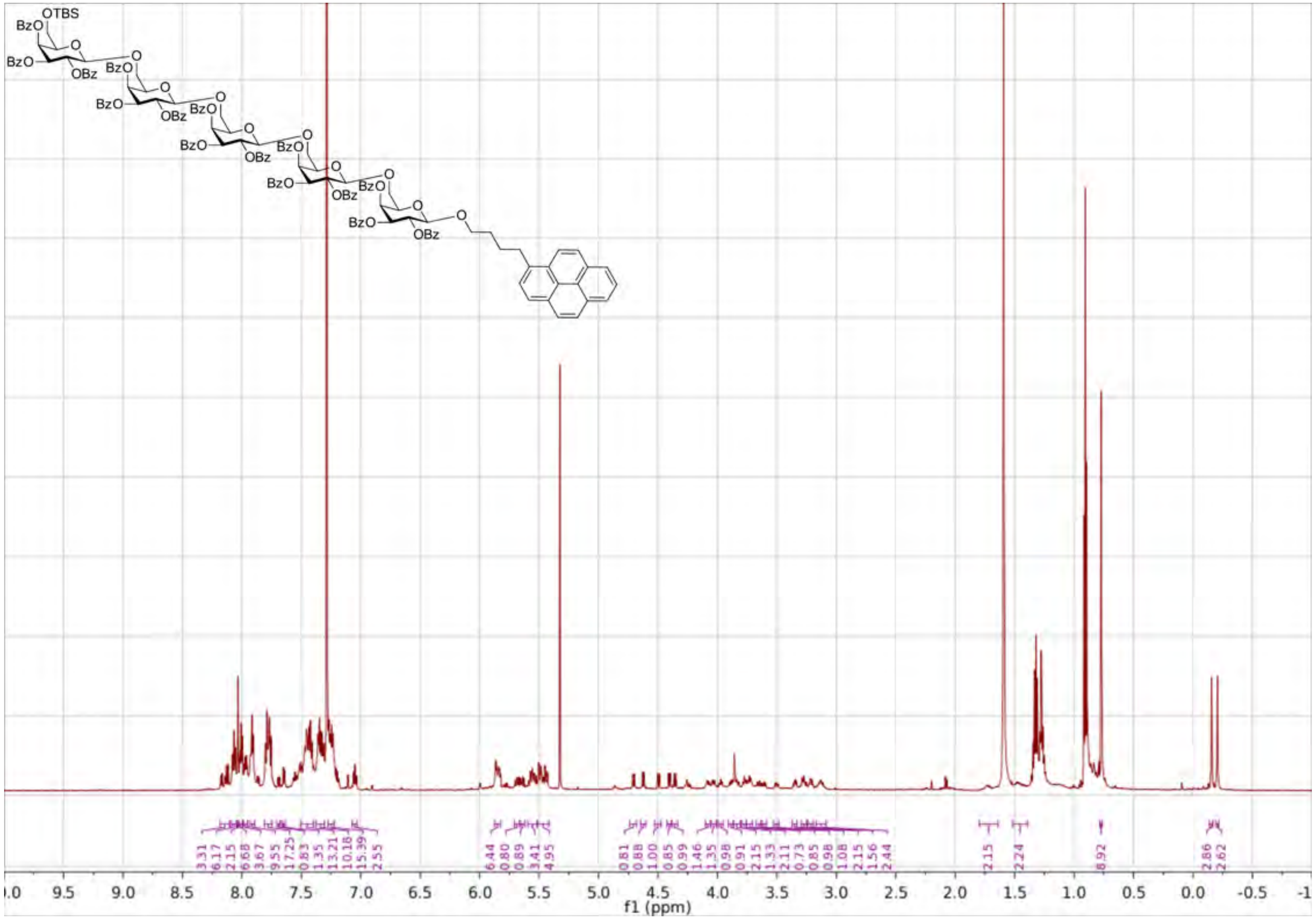

Compound **13_1b**

Proton

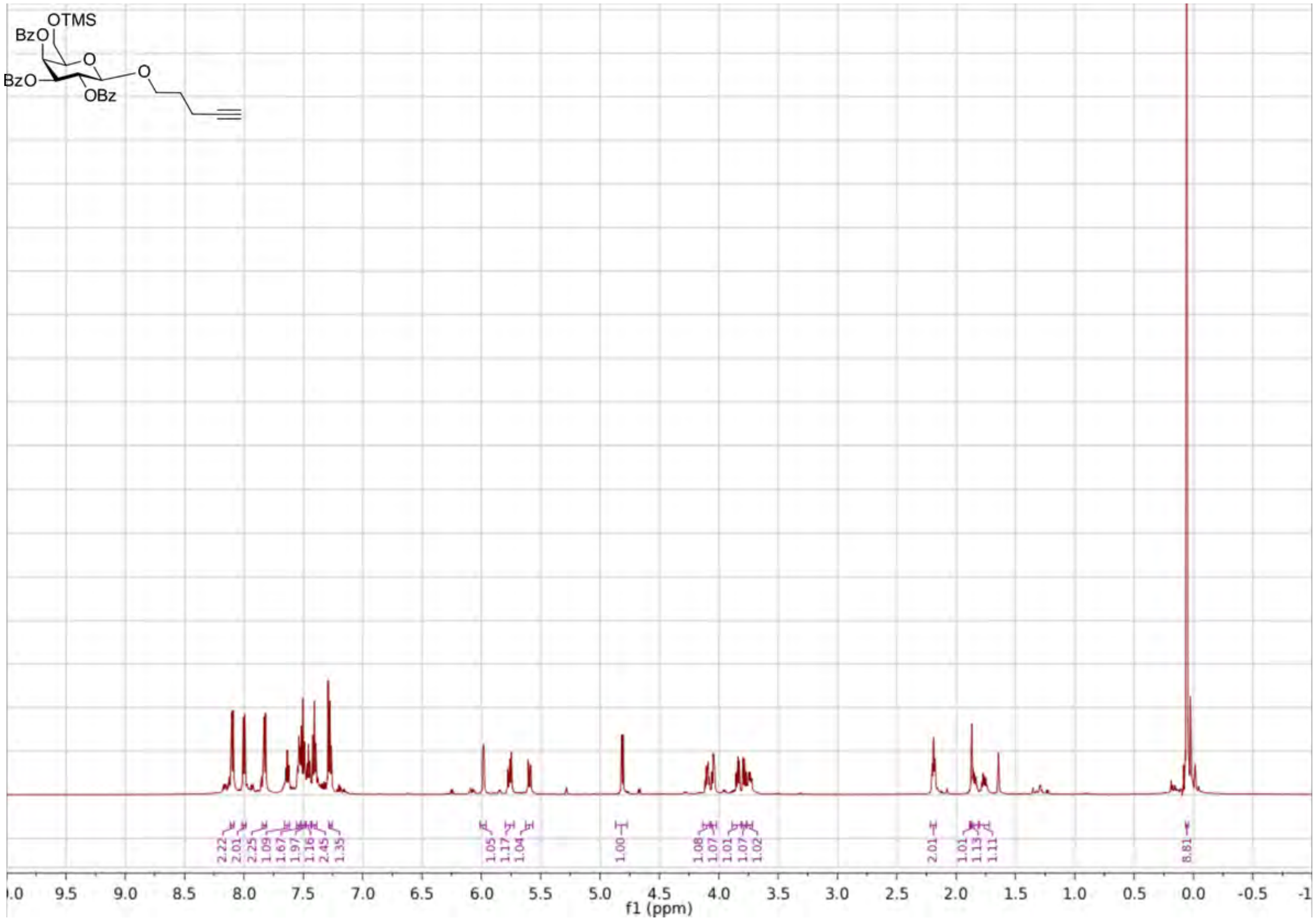

Carbon

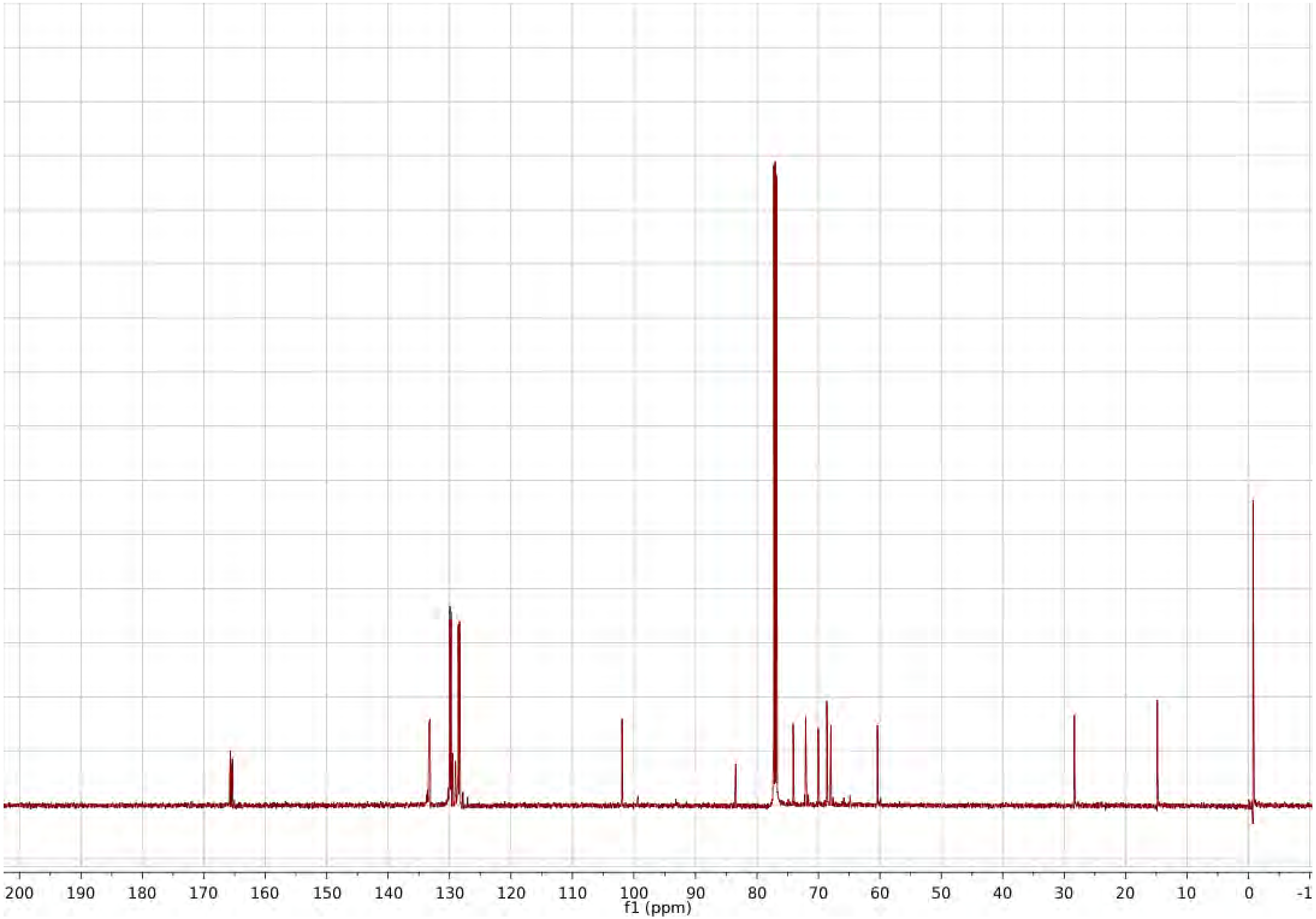
200
190
180
170
160
150
140
130
120
110
100
90
80
70
60
50
40
30
20
10
0
-1
f1 (ppm)

COSY

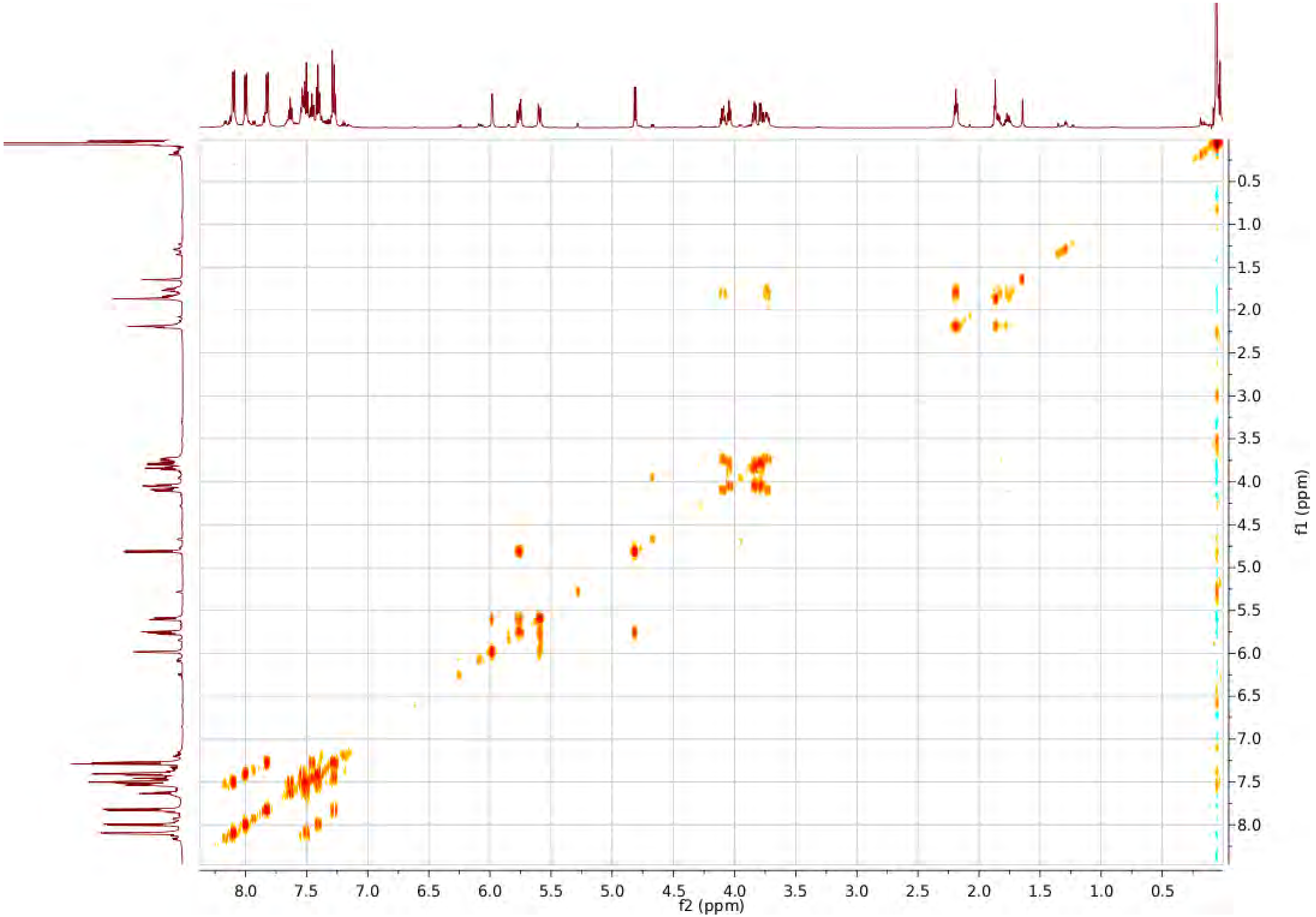

8.0
7.5
7.0
6.5
6.0
5.5
5.0
4.5
4.0
3.5
3.0
2.5
2.0
1.5
1.0
0.5
f2 (ppm)
0.5
1.0
1.5
2.0
2.5
3.0
3.5
4.0
4.5
5.0
5.5
6.0
6.5
7.0
7.5
8.0
f1 (ppm)

HSQC

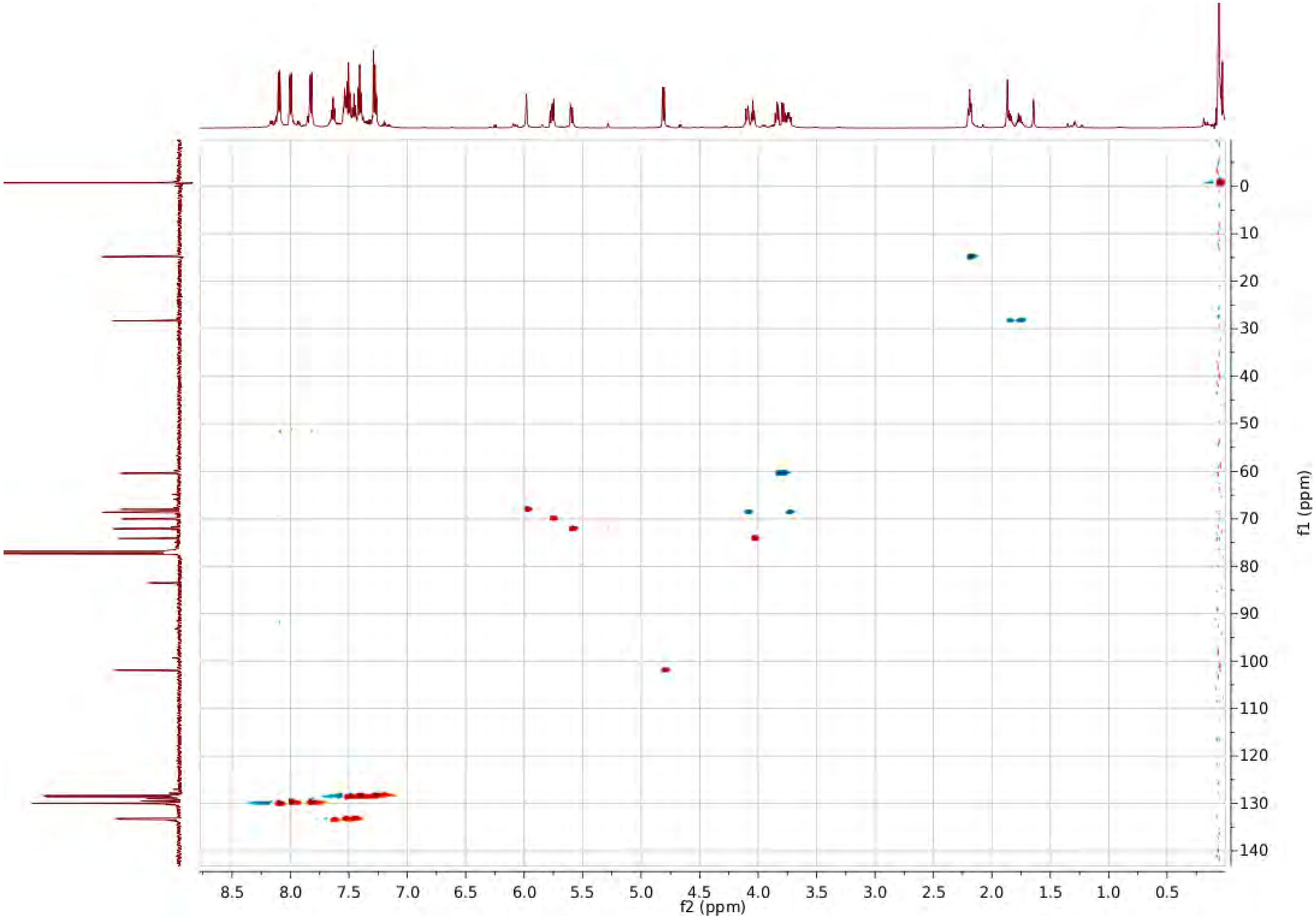

Compound **13_2b**

Proton

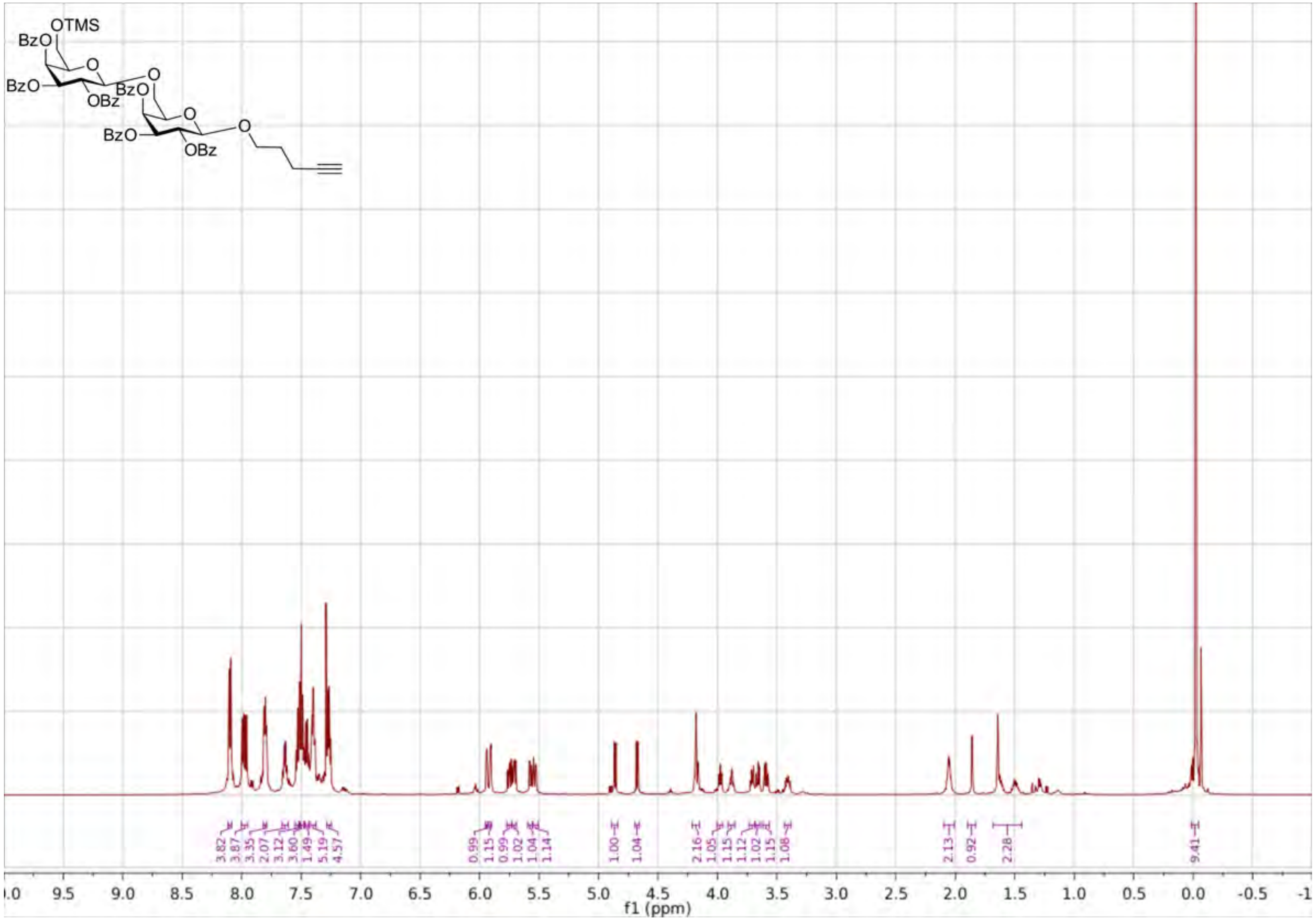

Carbon

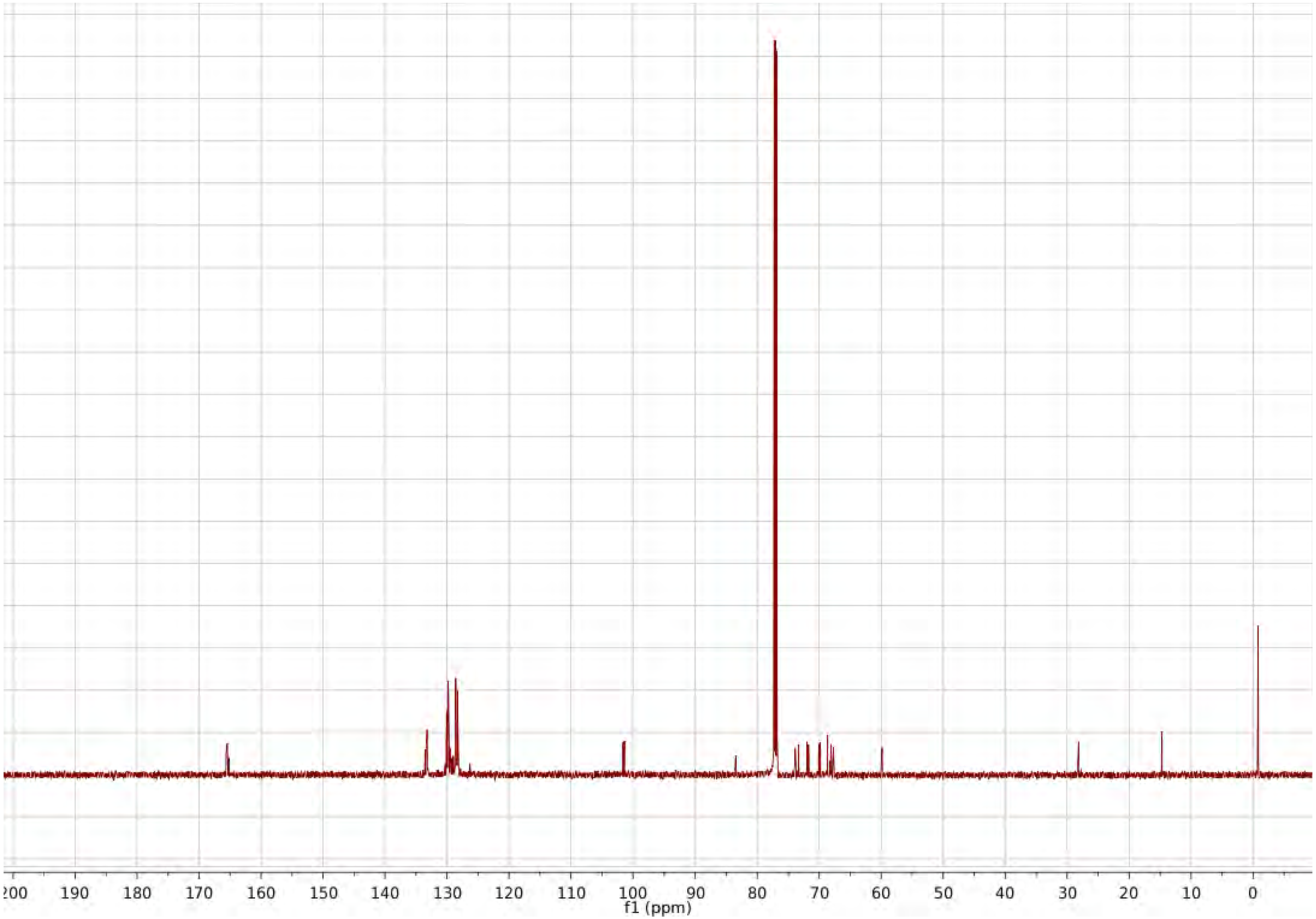

200
190
180
170
160
150
140
130
120
110
100
90
80
70
60
50
40
30
20
10
0
f1 (ppm)

COSY

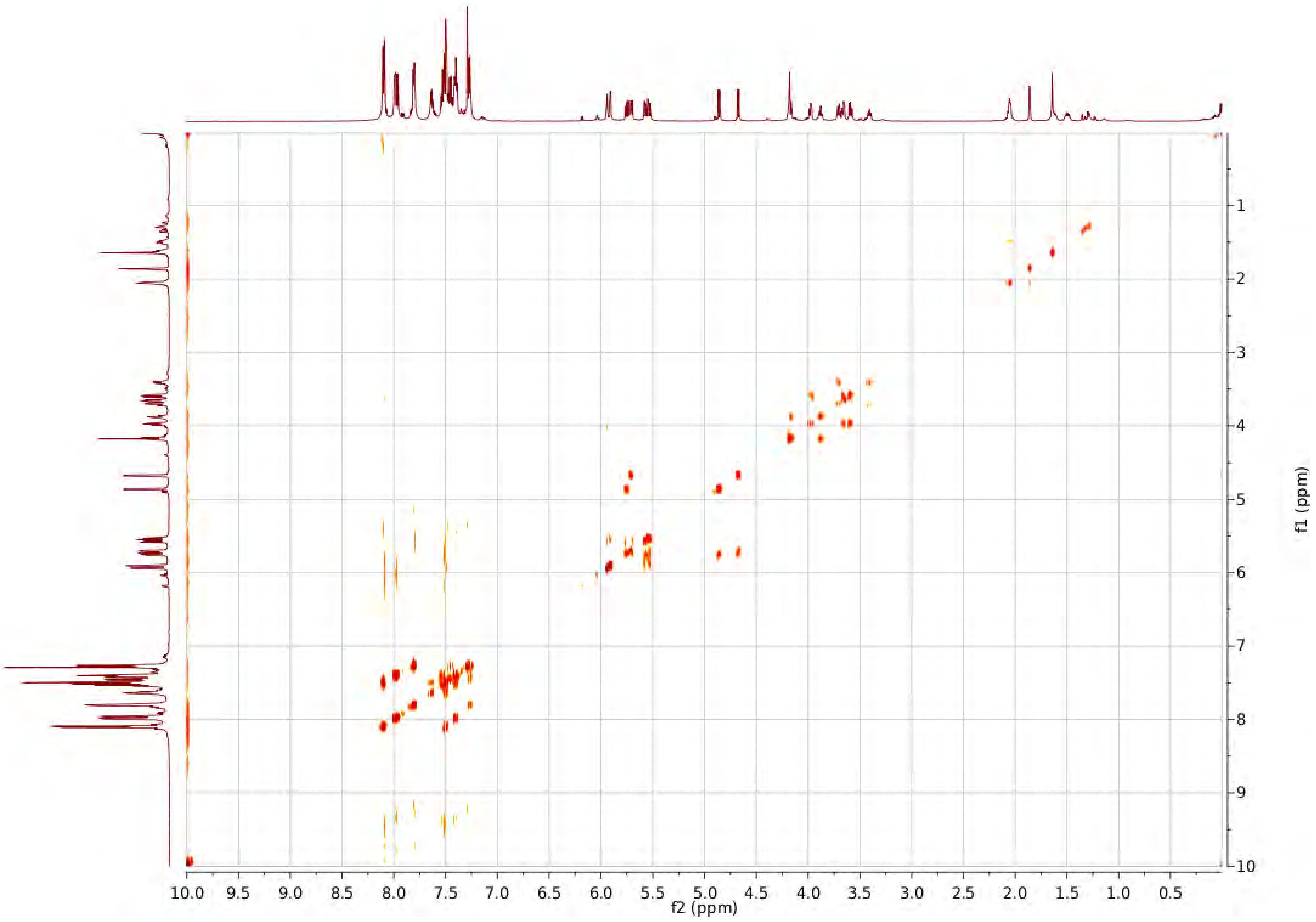

f2 (ppm)
f1 (ppm)
10.0
9.5
9.0
8.5
8.0
7.5
7.0
6.5
6.0
5.5
5.0
4.5
4.0
3.5
3.0
2.5
2.0
1.5
1.0
0.5
1
2
3
4
5
6
7
8
9
10

HSQC

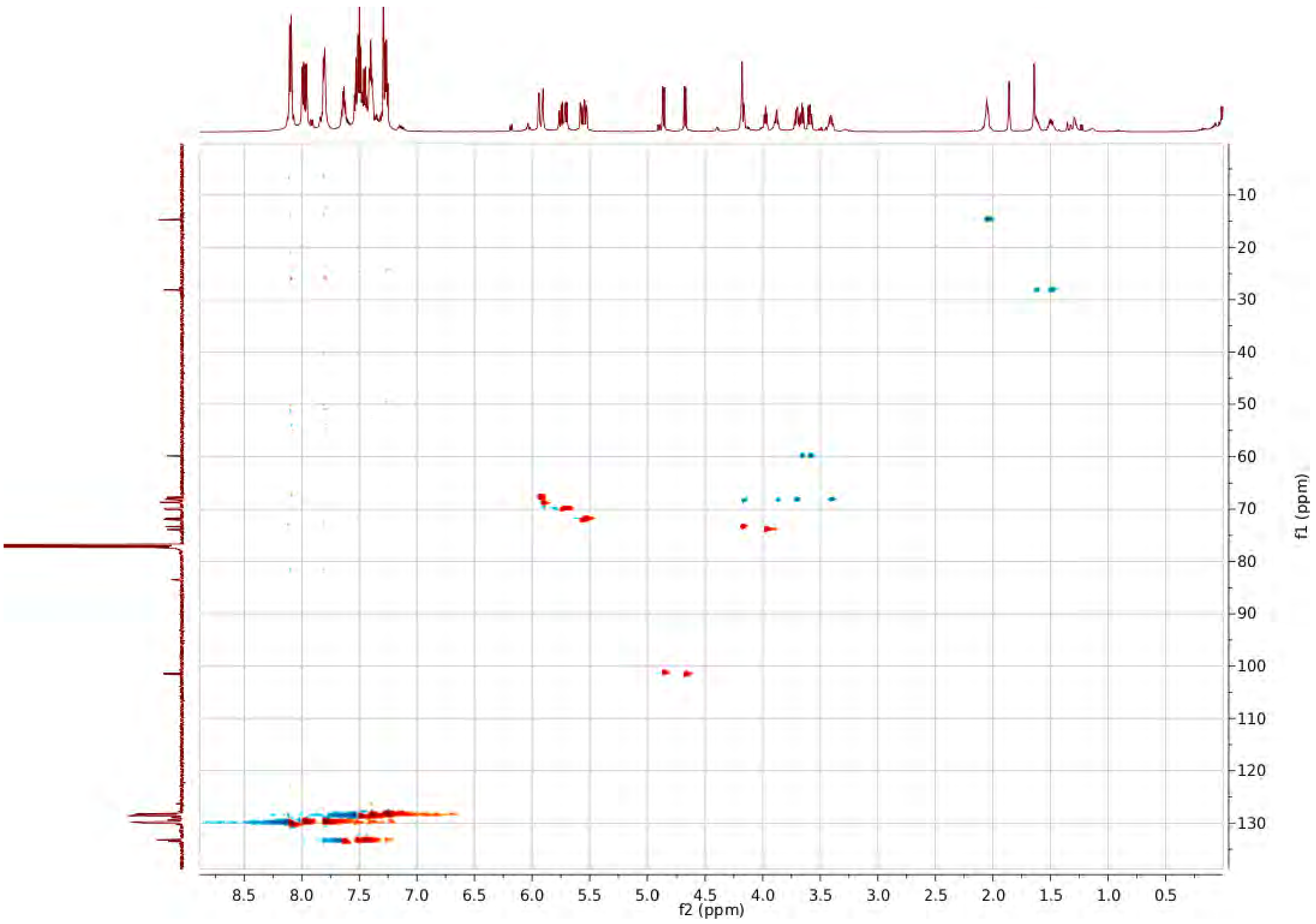

8.5
8.0
7.5
7.0
6.5
6.0
5.5
5.0
4.5
4.0
3.5
3.0
2.5
2.0
1.5
1.0
0.5
f2 (ppm)
10
20
30
40
50
60
70
80
90
100
110
120
130
f1 (ppm)

Compound **13$_3$b**

Proton

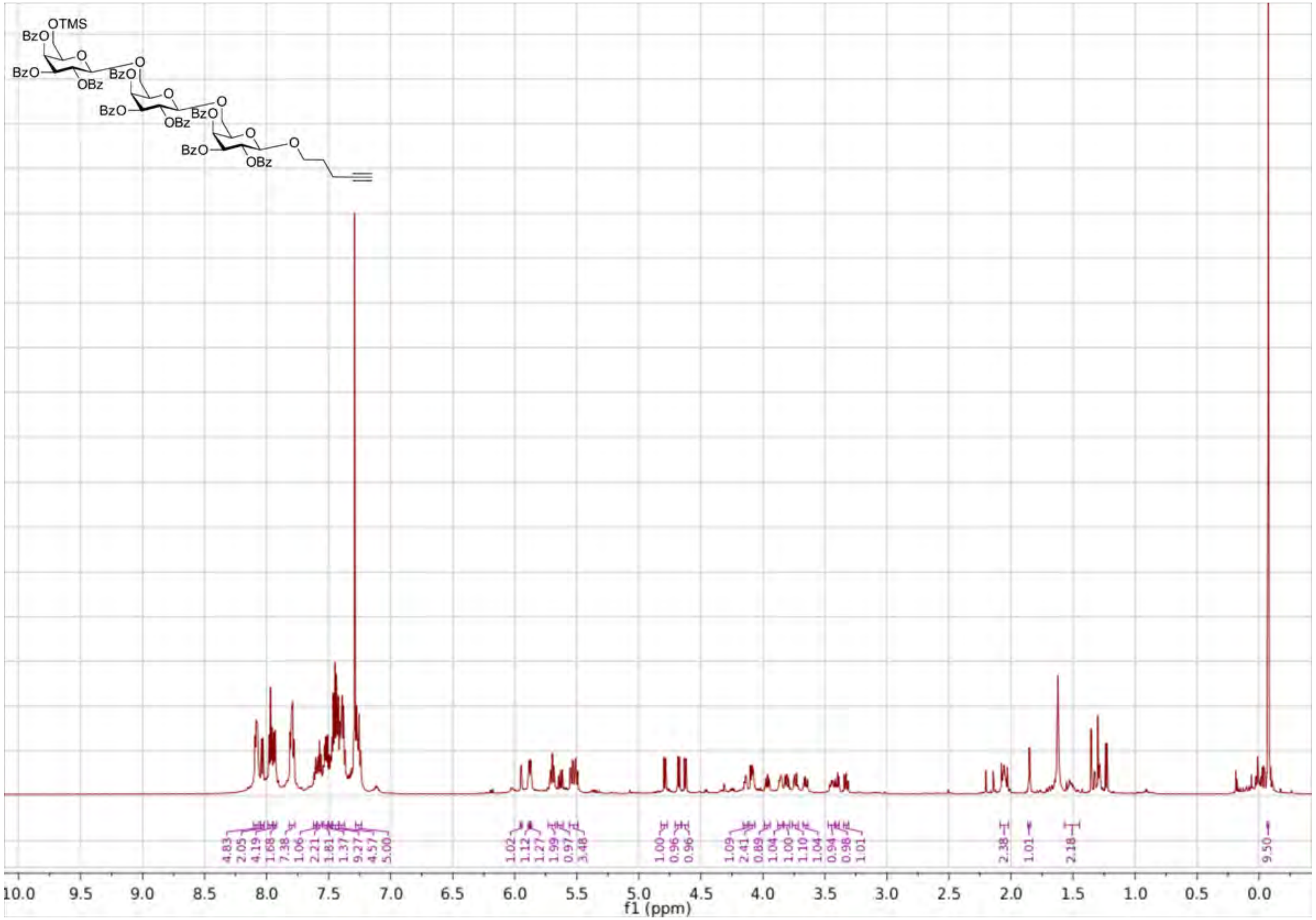

Carbon

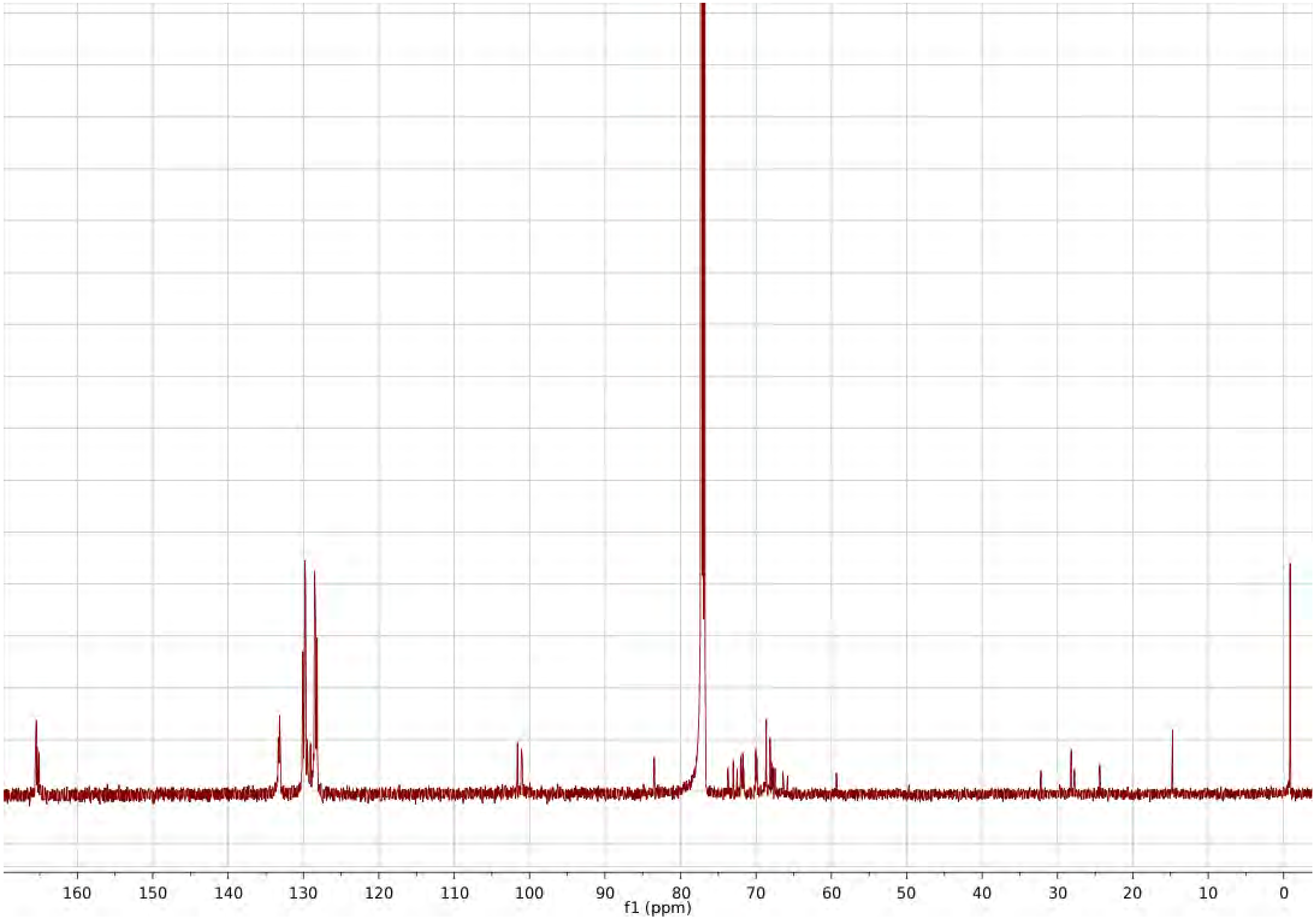

COSY

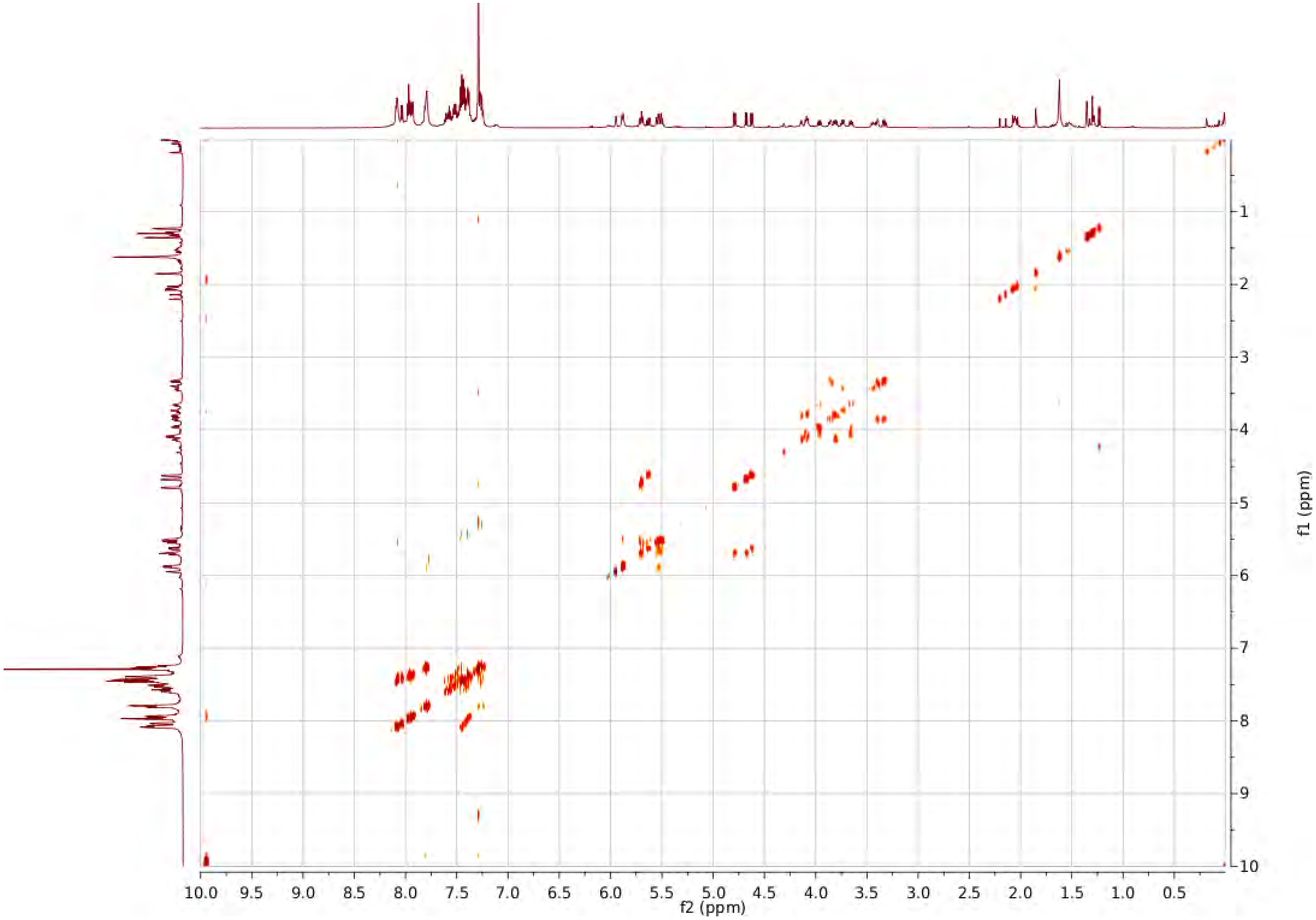
f2 (ppm)
f1 (ppm)
10.0
9.5
9.0
8.5
8.0
7.5
7.0
6.5
6.0
5.5
5.0
4.5
4.0
3.5
3.0
2.5
2.0
1.5
1.0
0.5
1
2
3
4
5
6
7
8
9
10

HSQC

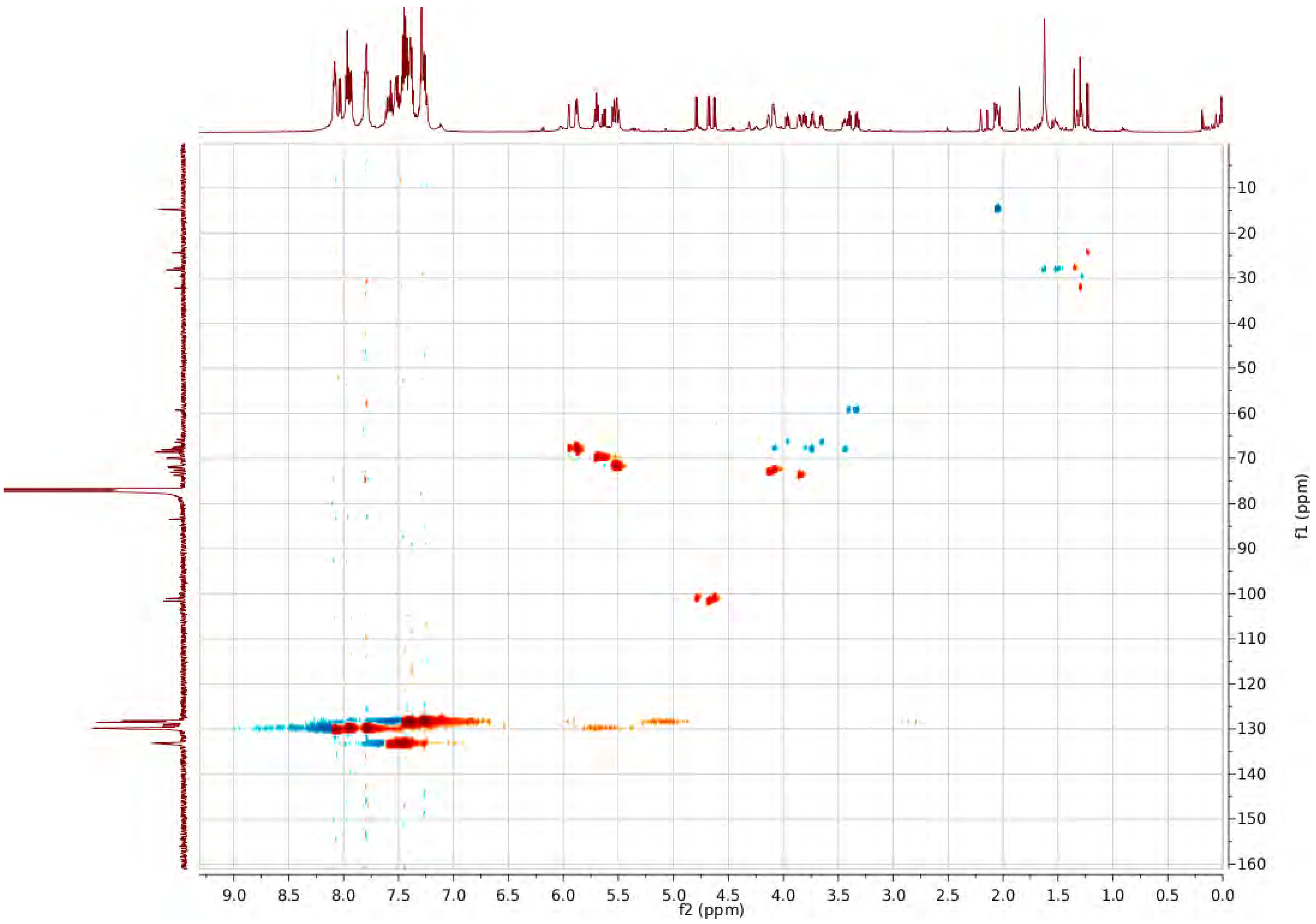

9.0 8.5 8.0 7.5 7.0 6.5 6.0 5.5 5.0 4.5 4.0 3.5 3.0 2.5 2.0 1.5 1.0 0.5 0.0
f2 (ppm)
10 20 30 40 50 60 70 80 90 100 110 120 130 140 150 160
f1 (ppm)

Compound **13$_4$b**

Proton

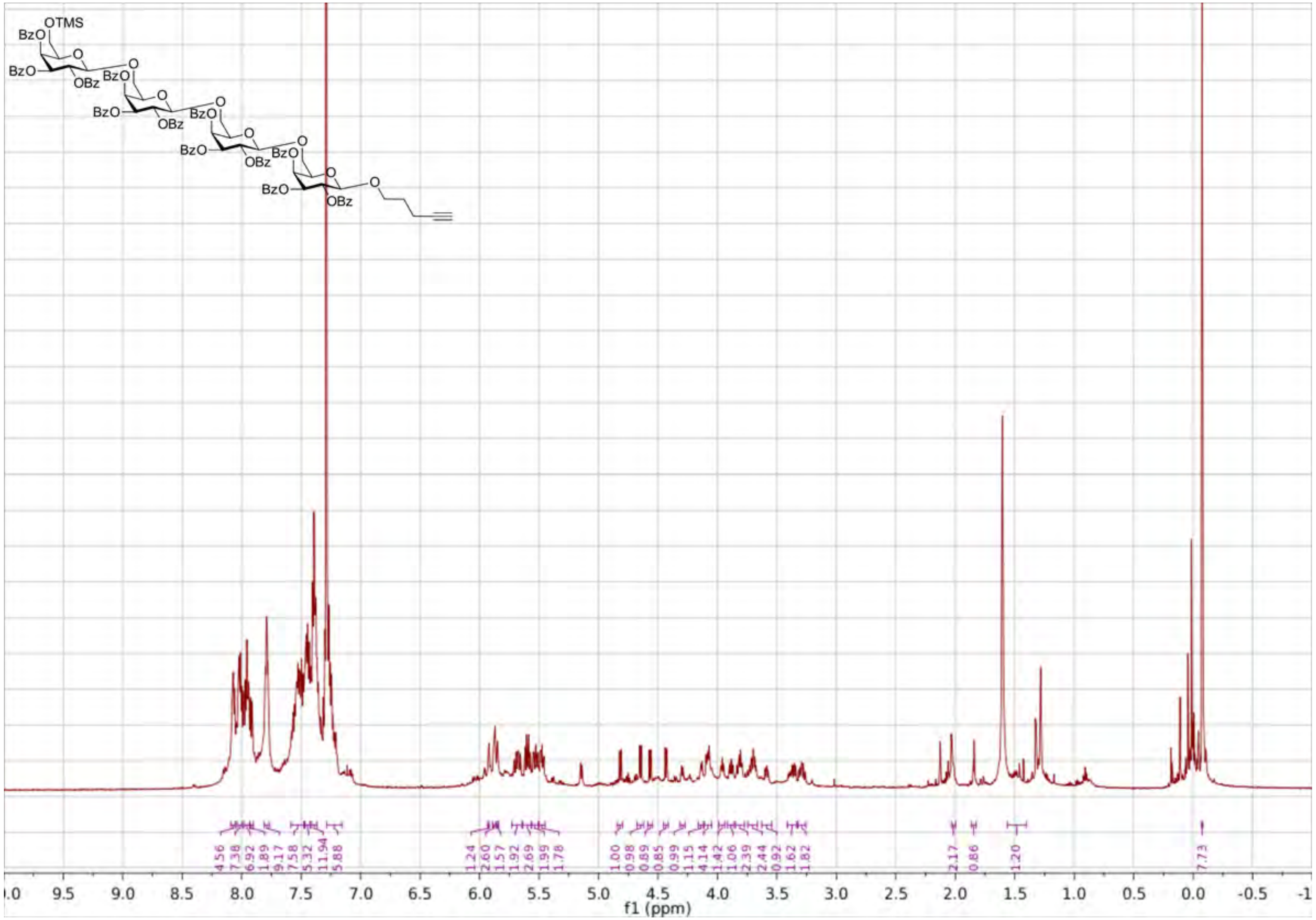

Compound $\mathbf{14_1c}$

Proton

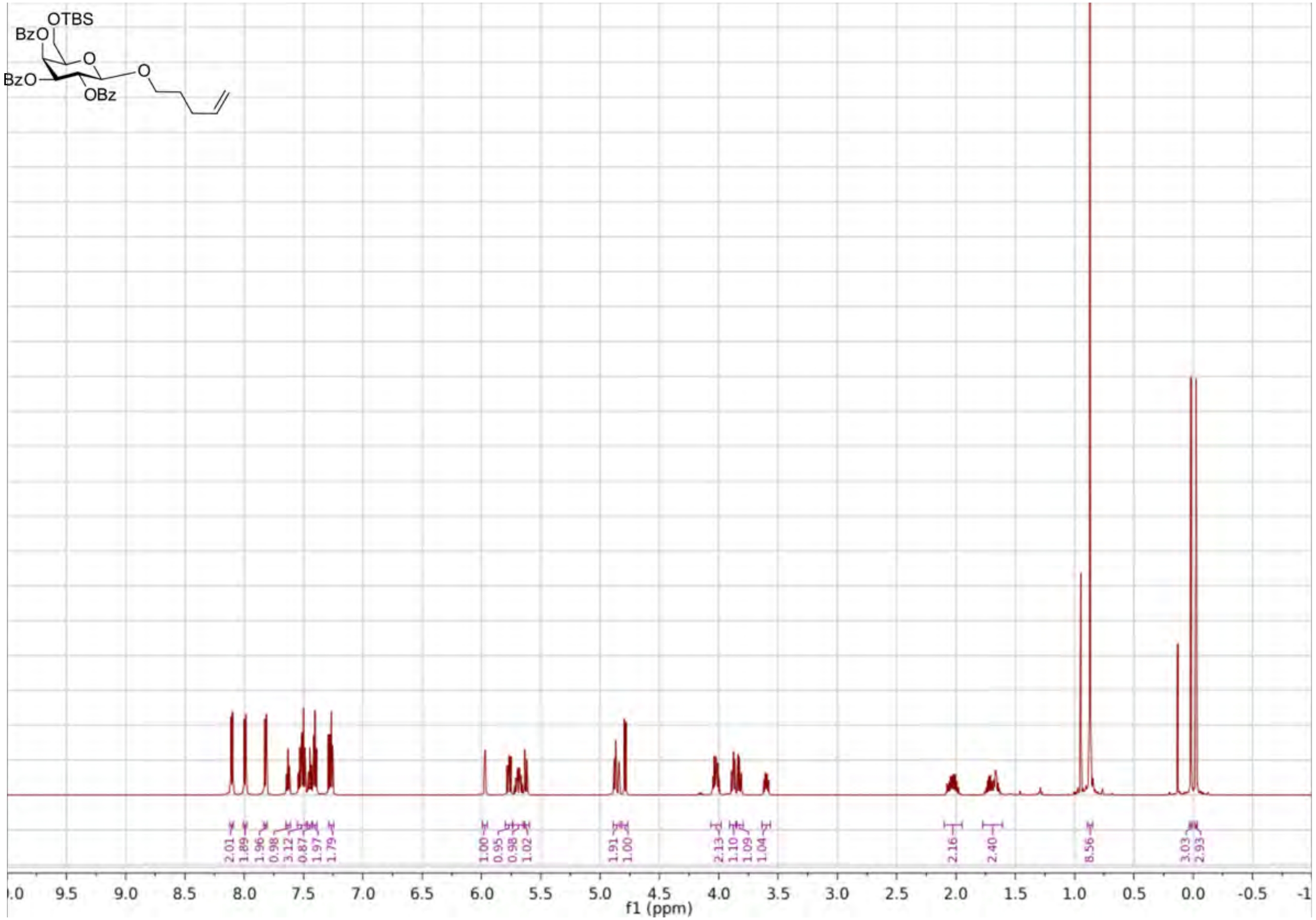

Carbon

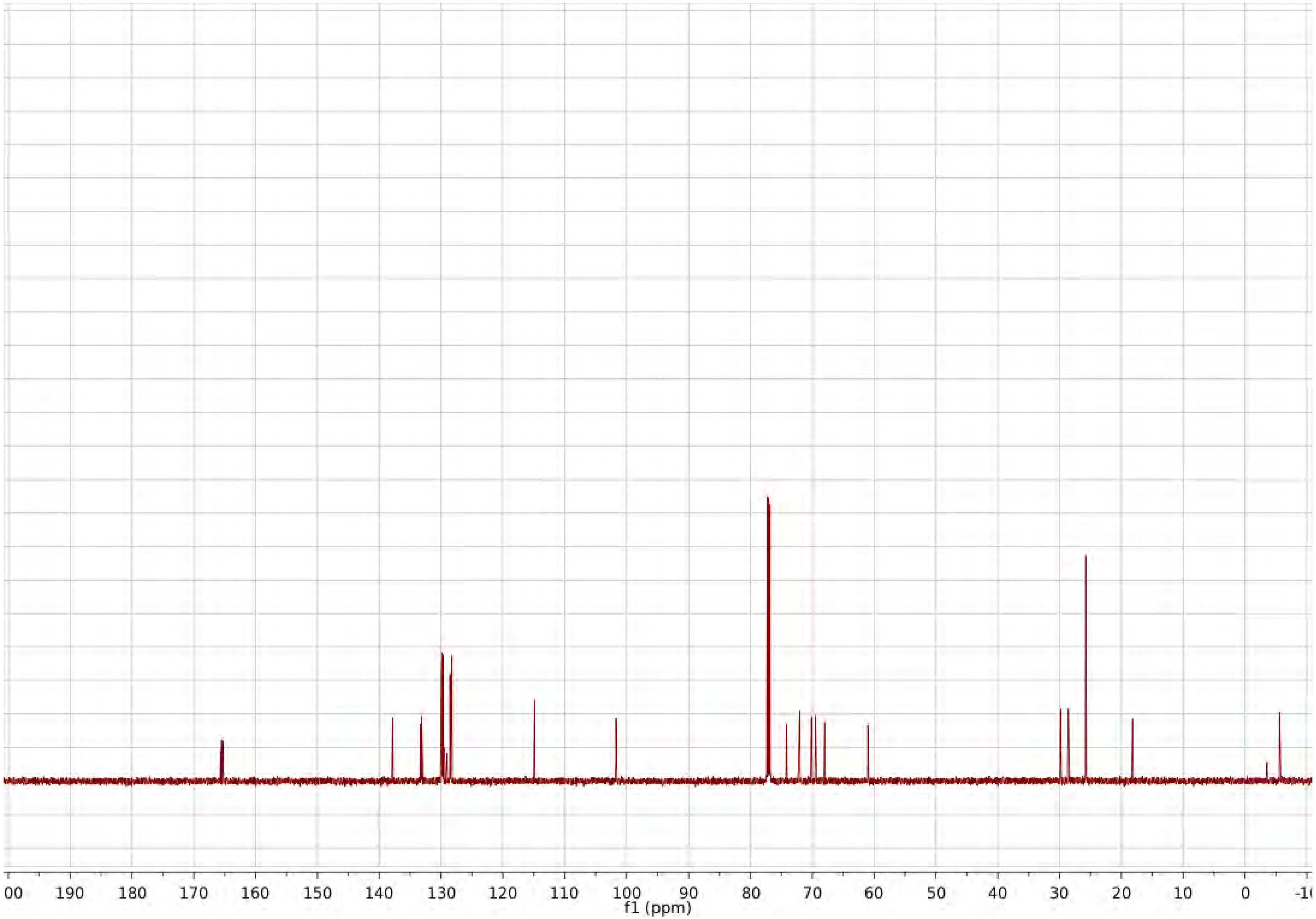
00 190 180 170 160 150 140 130 120 110 100 90 80 70 60 50 40 30 20 10 0 -1
f1 (ppm)

COSY

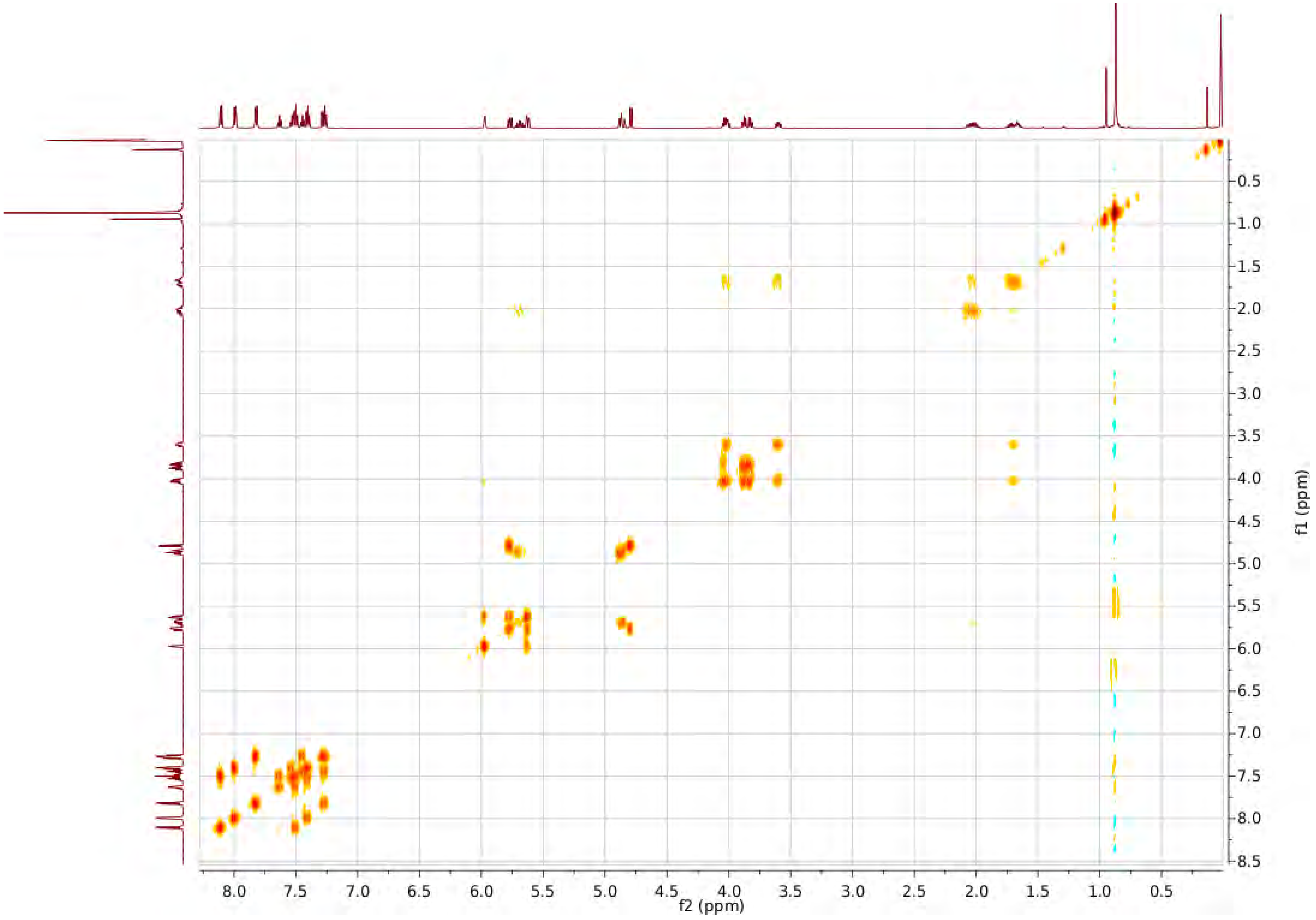

HSQC

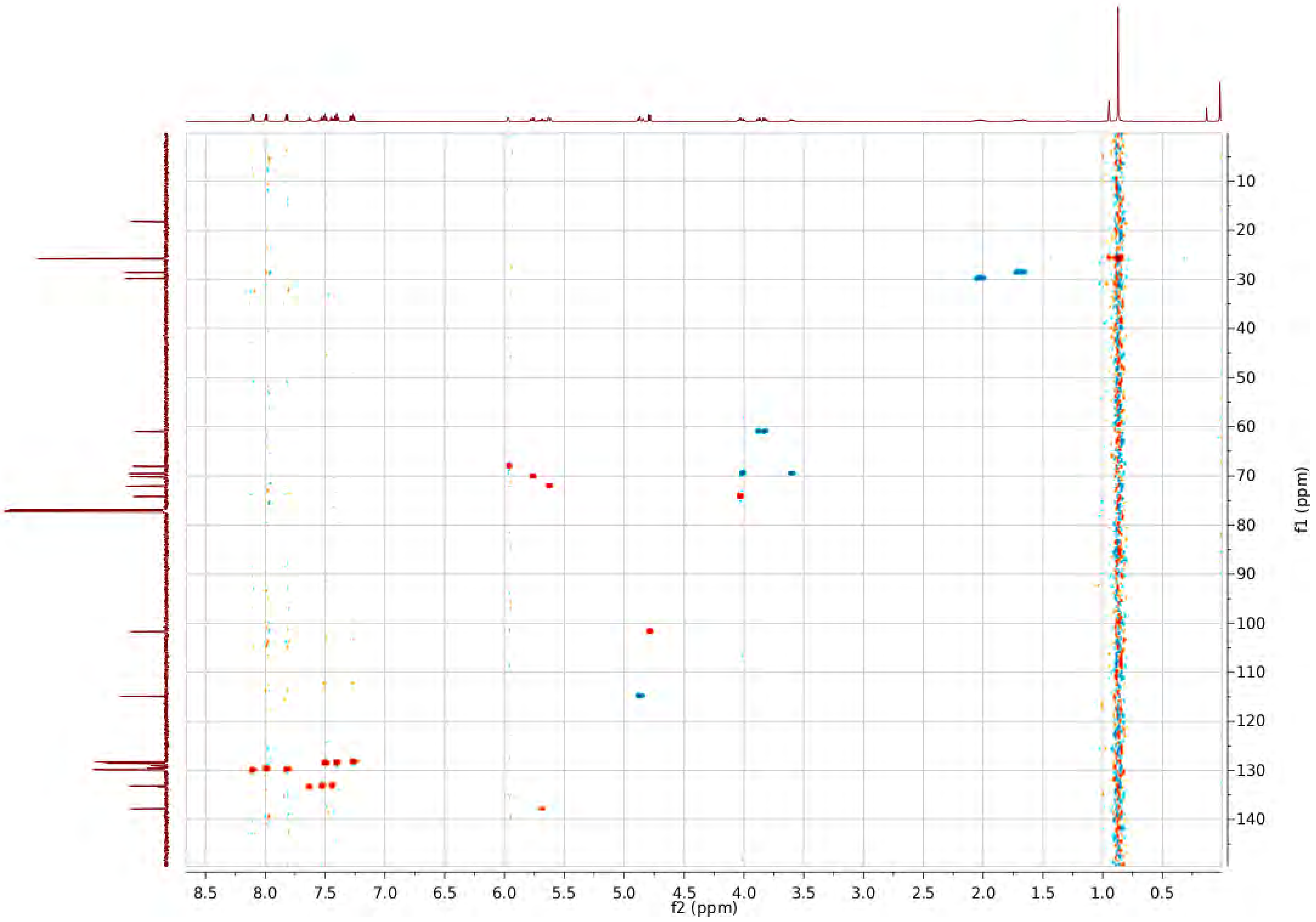

8.5
8.0
7.5
7.0
6.5
6.0
5.5
5.0
4.5
4.0
3.5
3.0
2.5
2.0
1.5
1.0
0.5
f2 (ppm)
10
20
30
40
50
60
70
80
90
100
110
120
130
140
f1 (ppm)

Compound **14₂c**

Proton

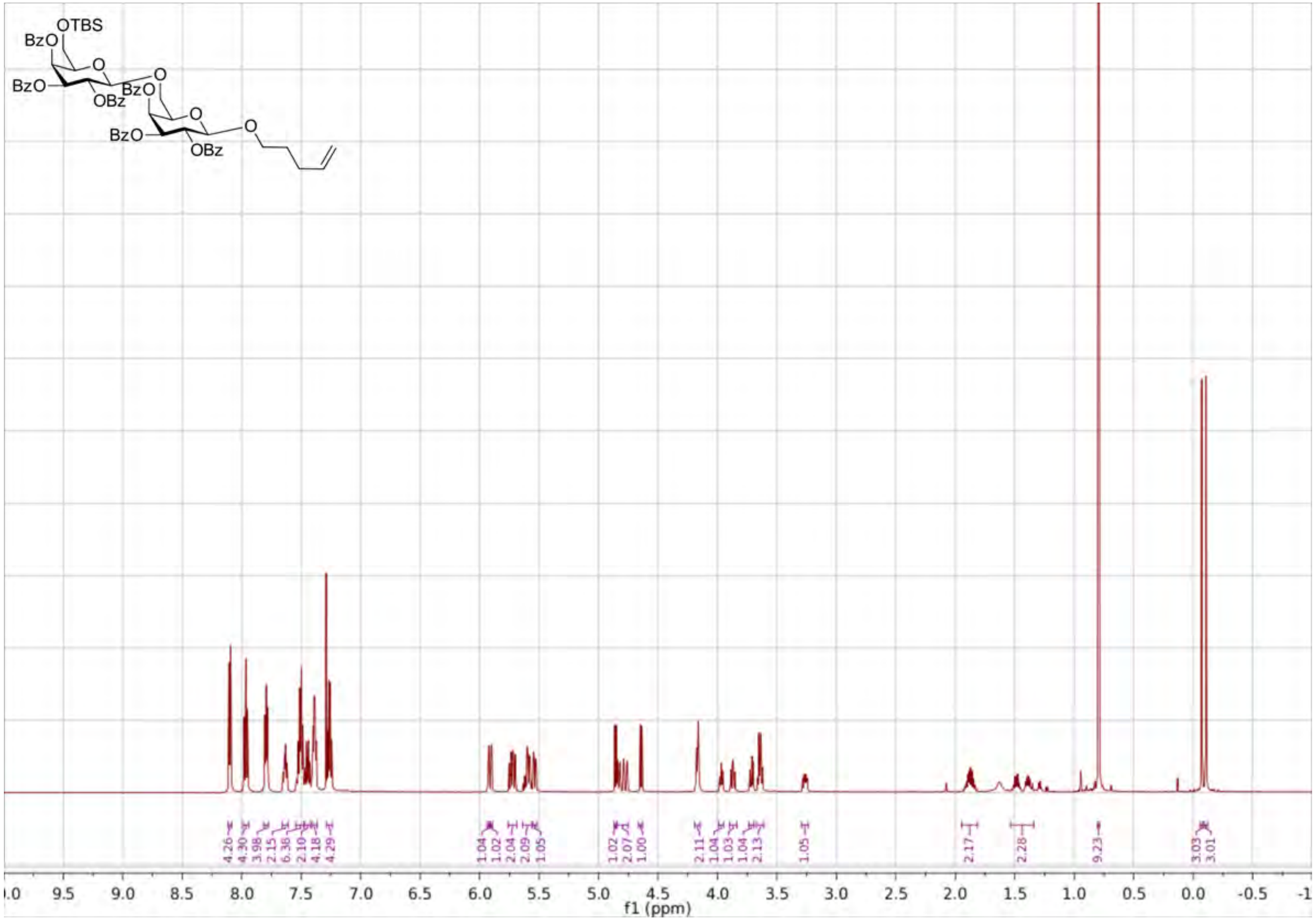

Carbon

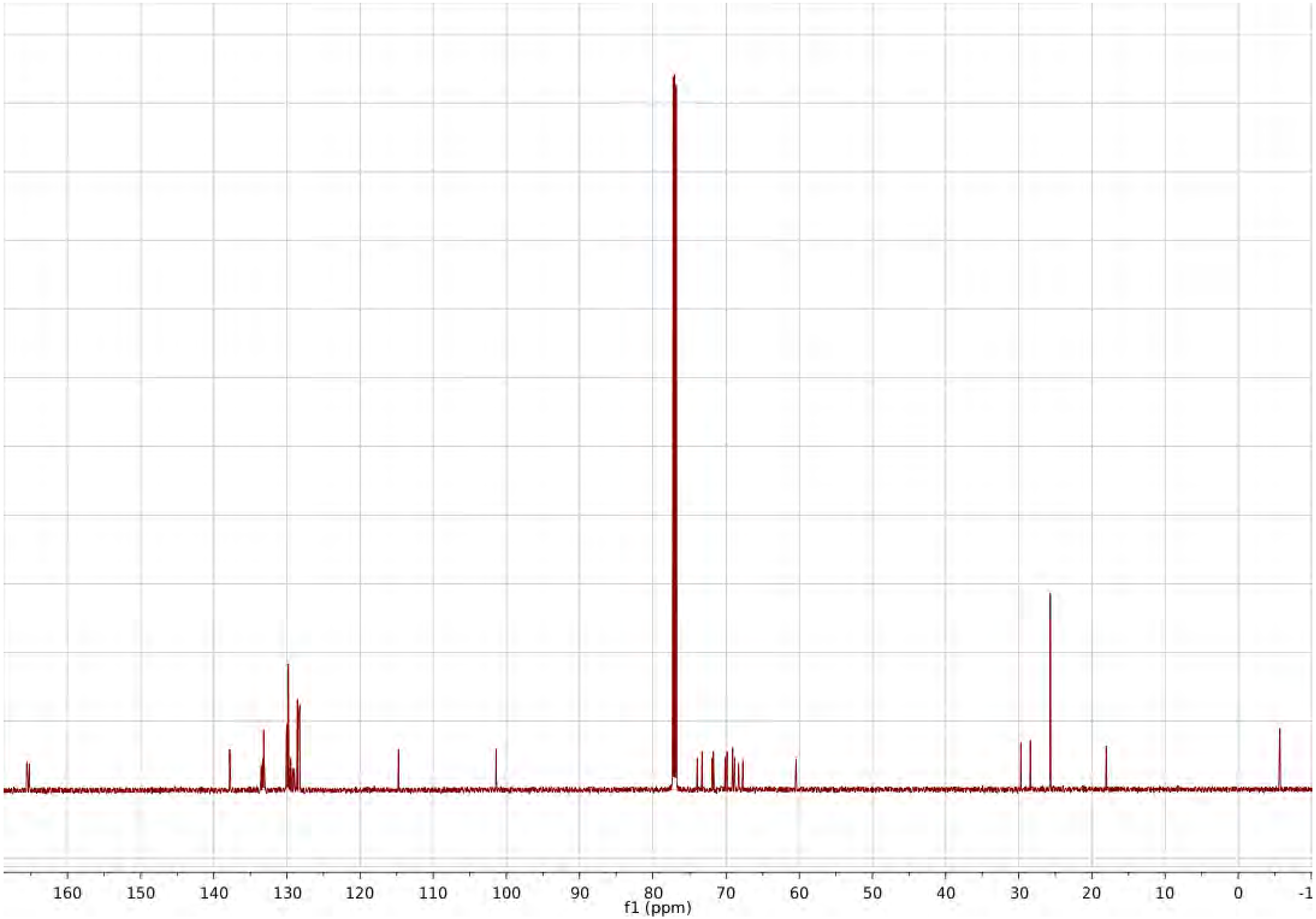

COSY

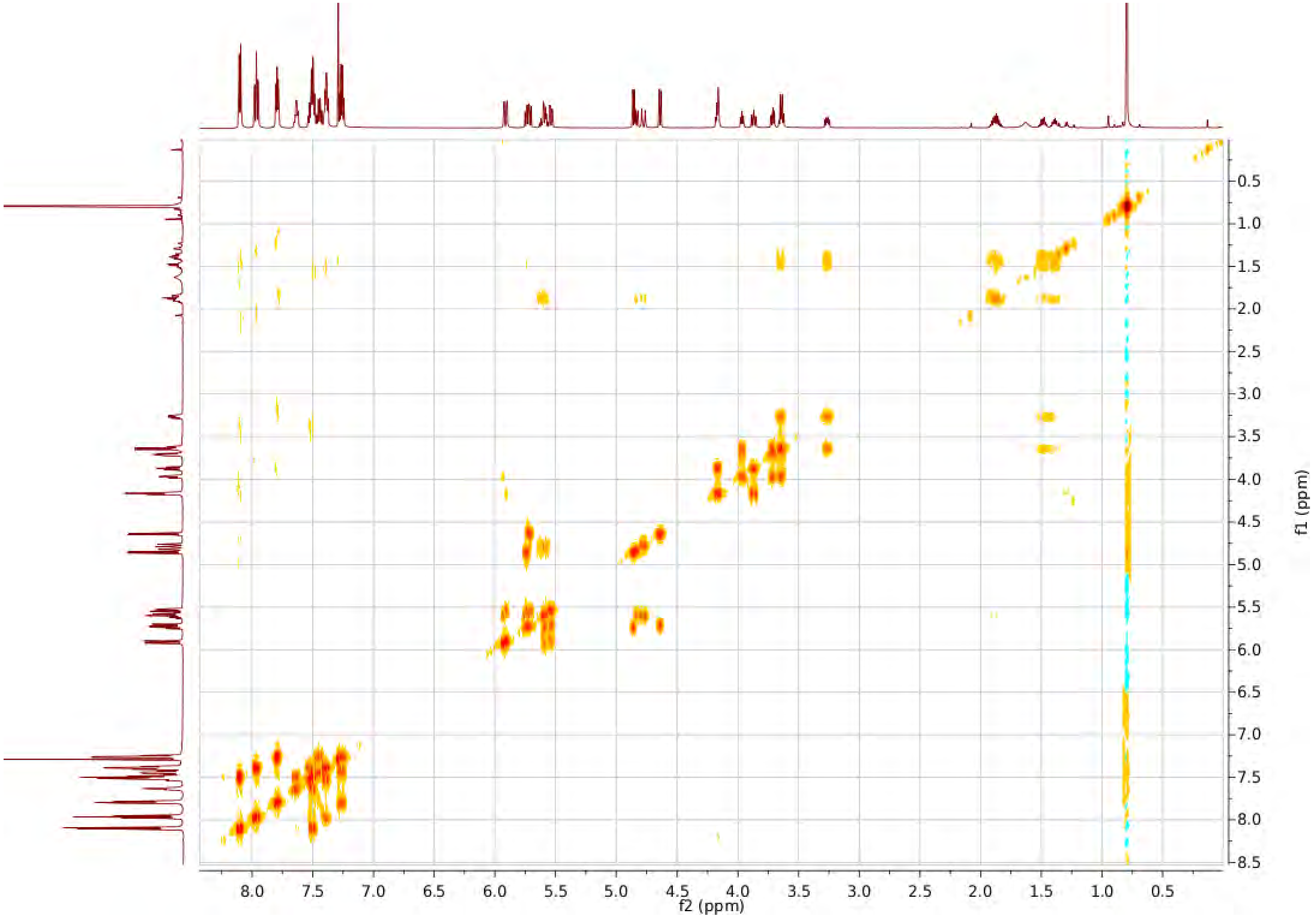

HSQC

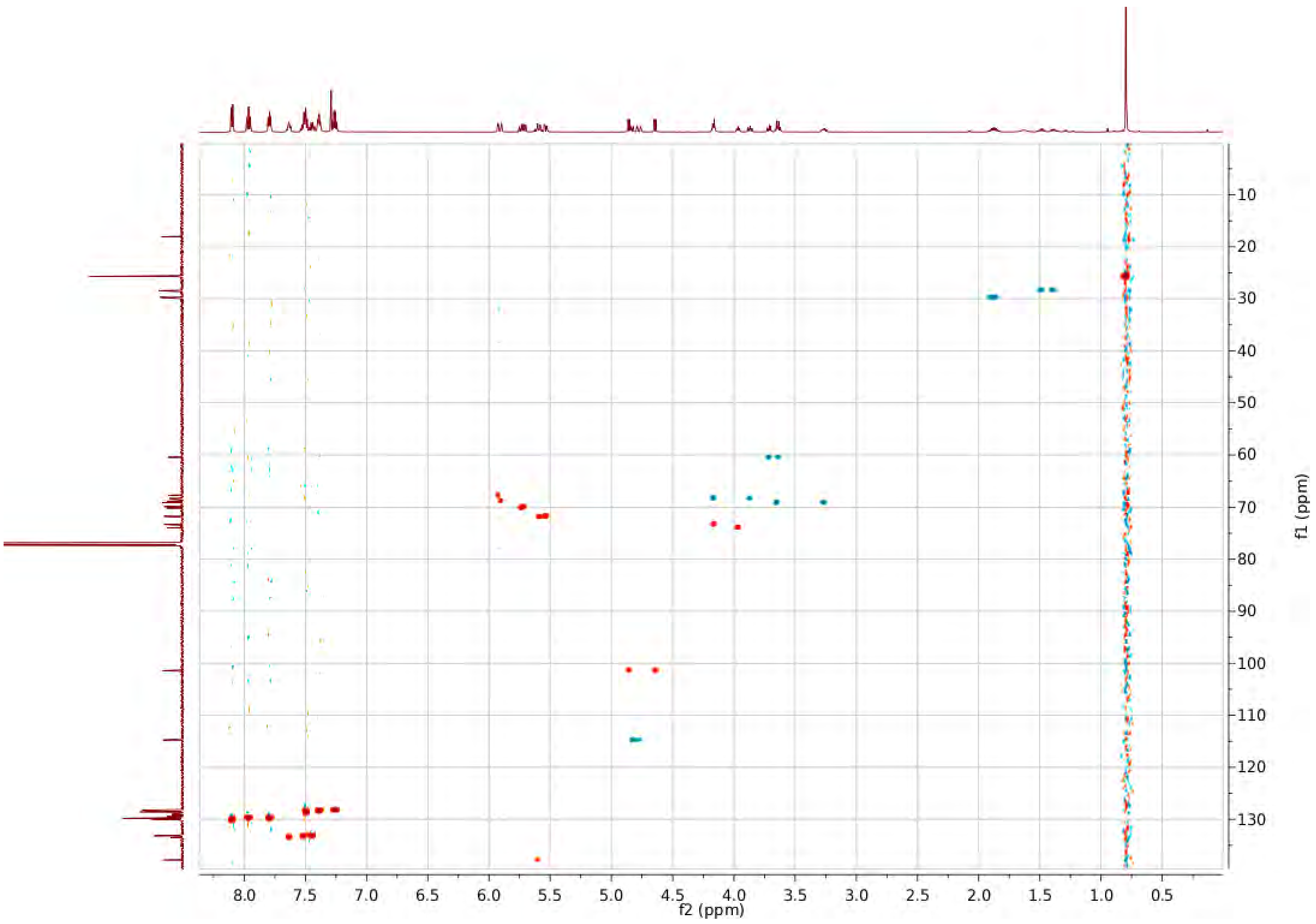

f2 (ppm)
f1 (ppm)
8.0
7.5
7.0
6.5
6.0
5.5
5.0
4.5
4.0
3.5
3.0
2.5
2.0
1.5
1.0
0.5
10
20
30
40
50
60
70
80
90
100
110
120
130

Compound **14₃c**

Proton

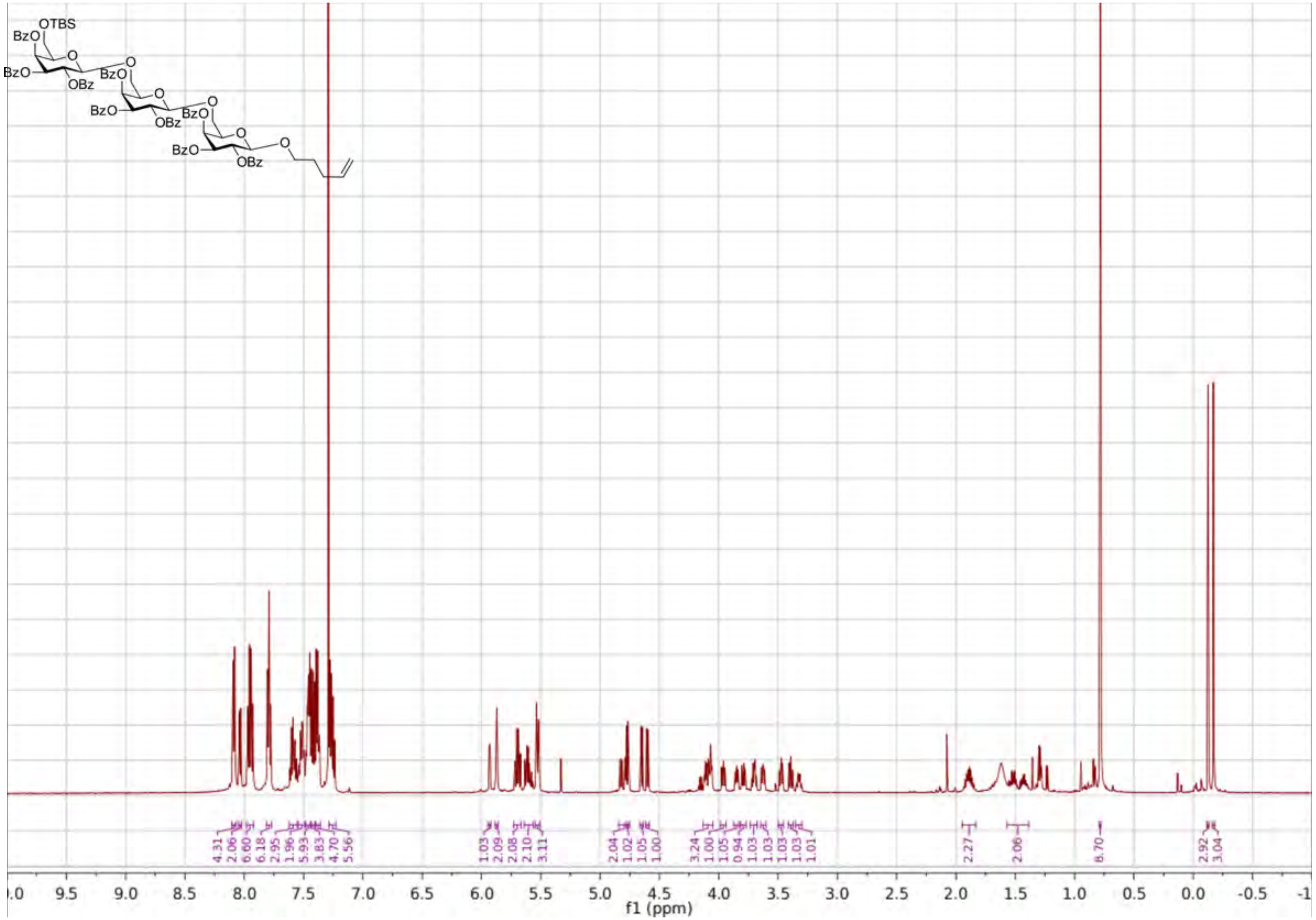

Carbon

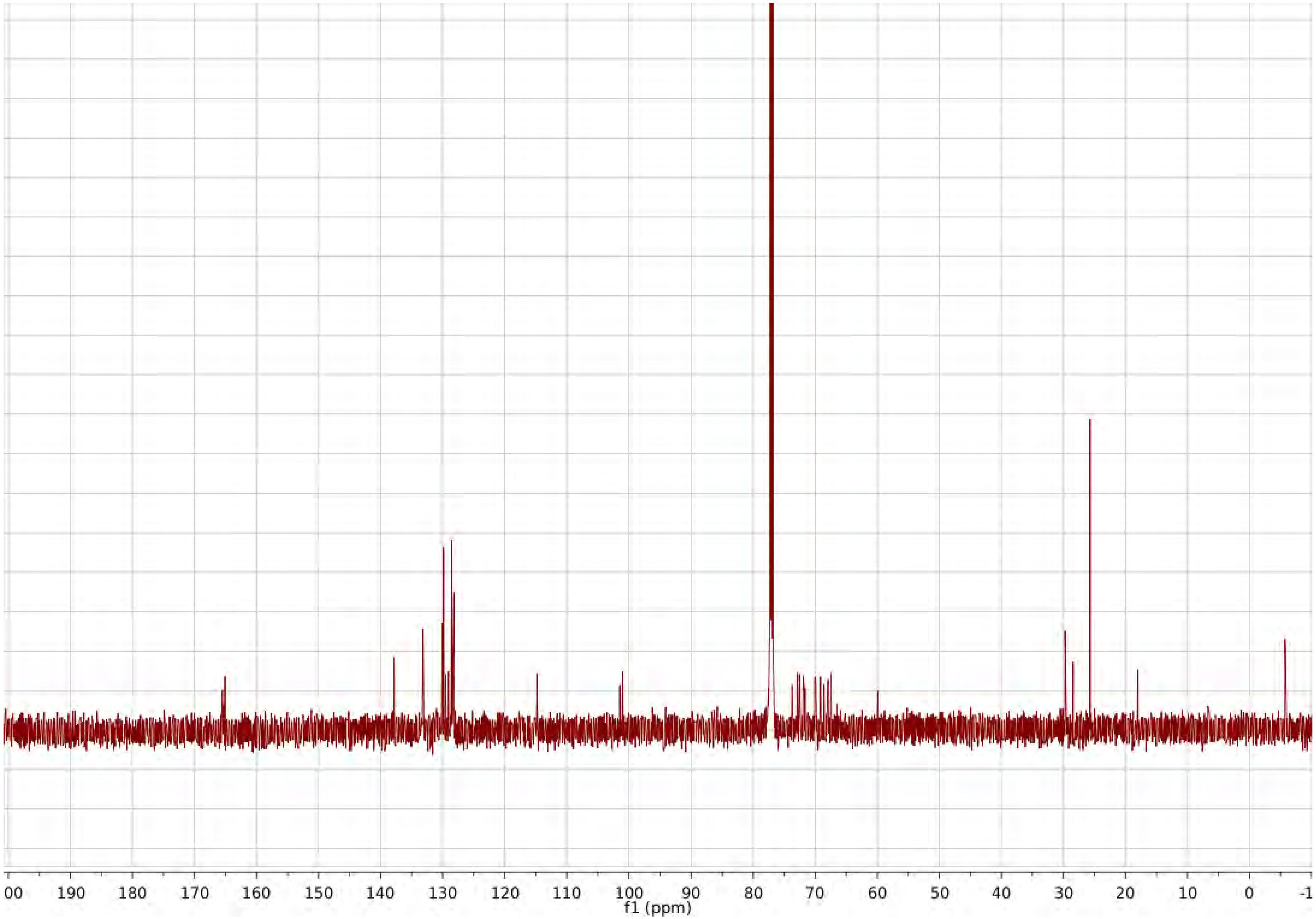

00
190
180
170
160
150
140
130
120
110
100
90
80
70
60
50
40
30
20
10
0
-1
f1 (ppm)

COSY

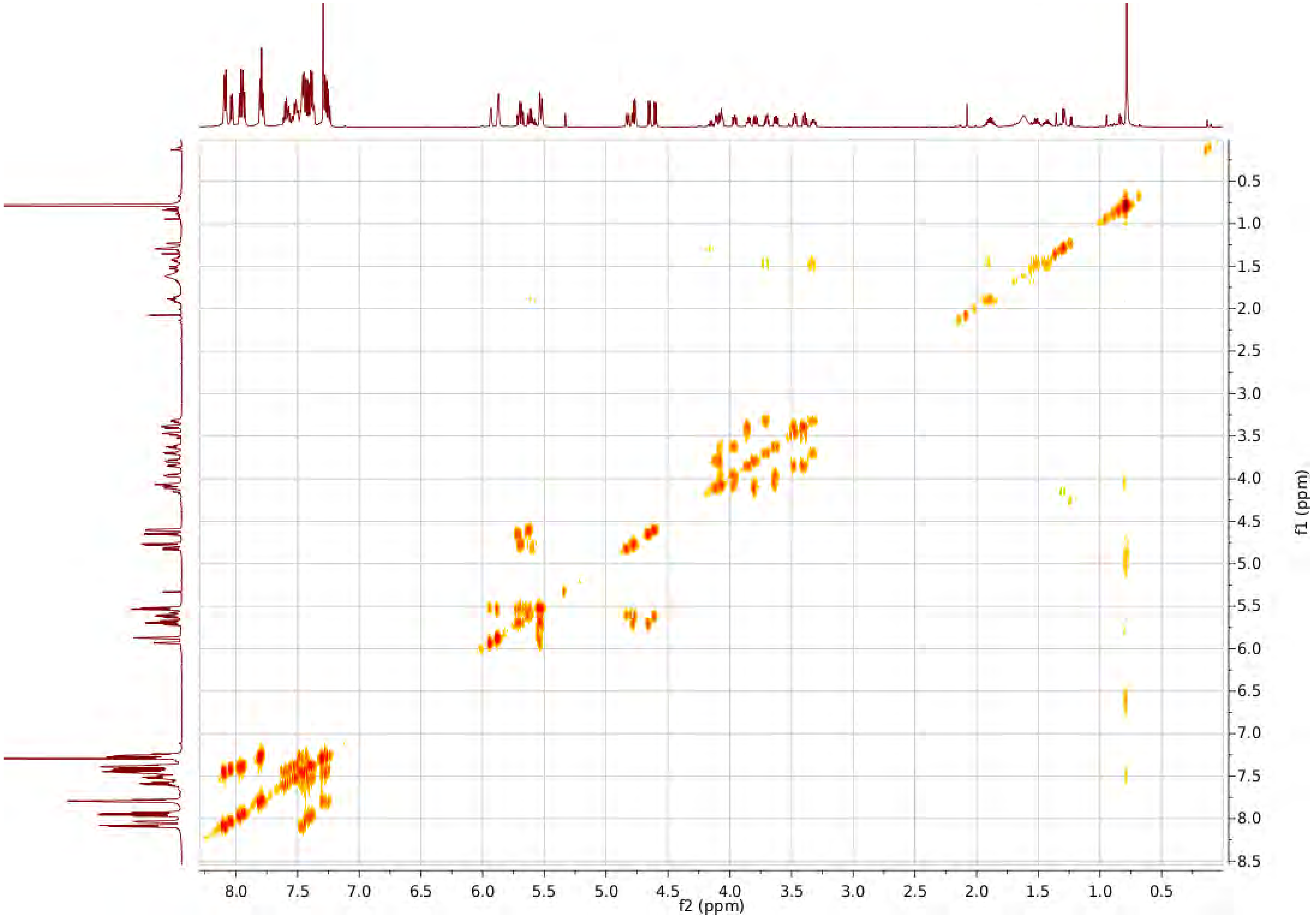

HSQC

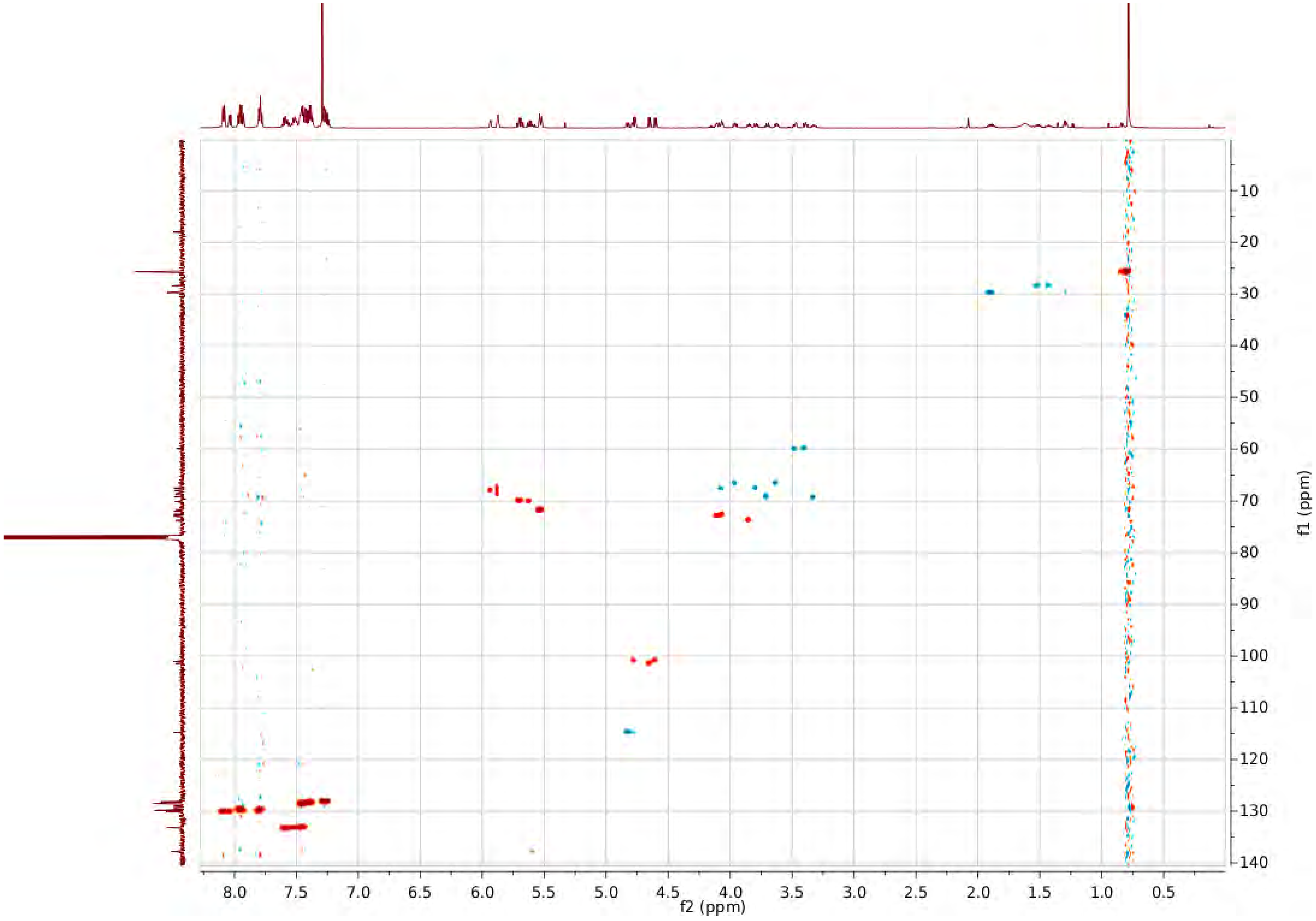

Compound **15₂b**

Proton

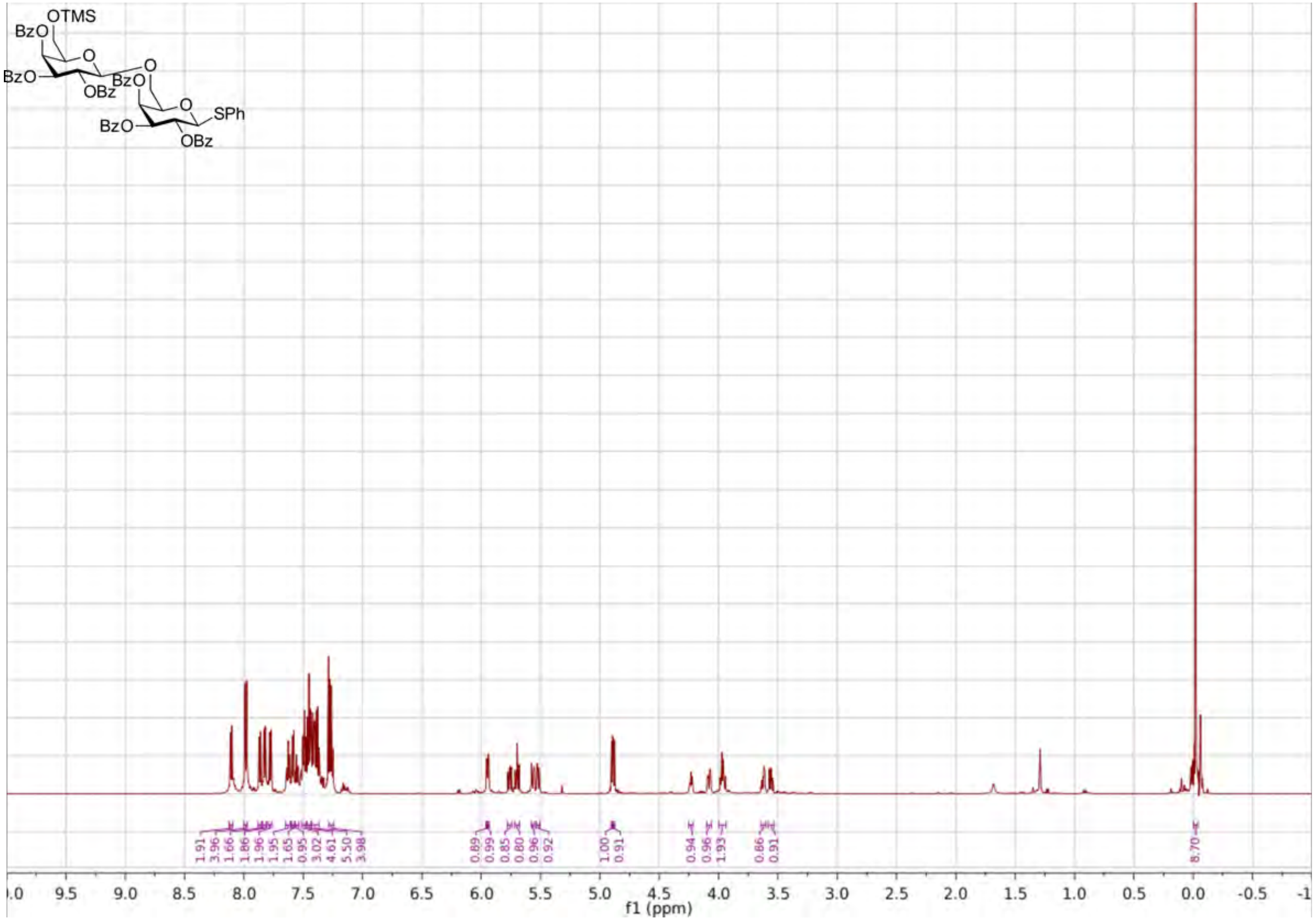

Carbon

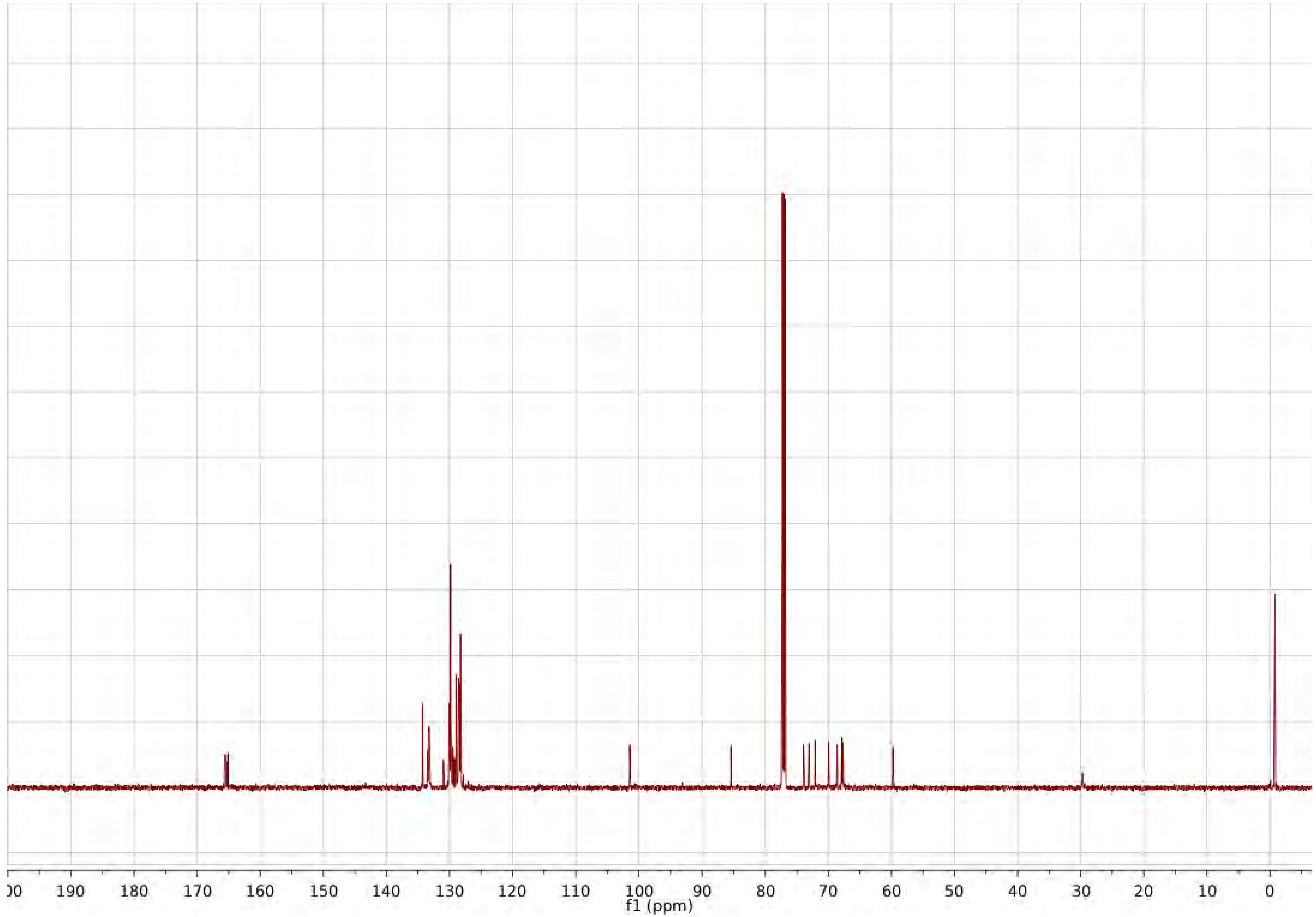

00
190
180
170
160
150
140
130
120
110
100
90
80
70
60
50
40
30
20
10
0
f1 (ppm)

COSY

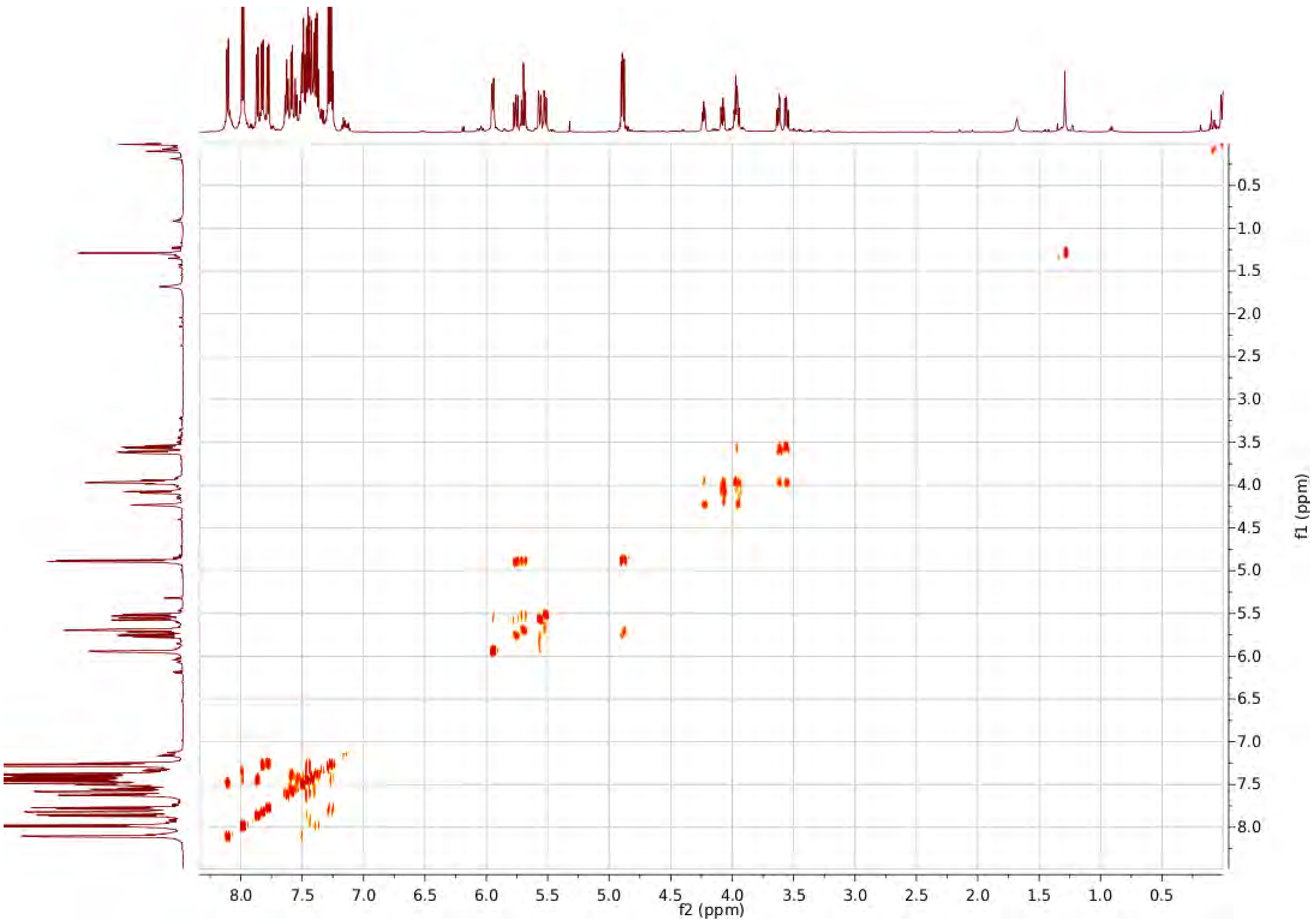

HSQC

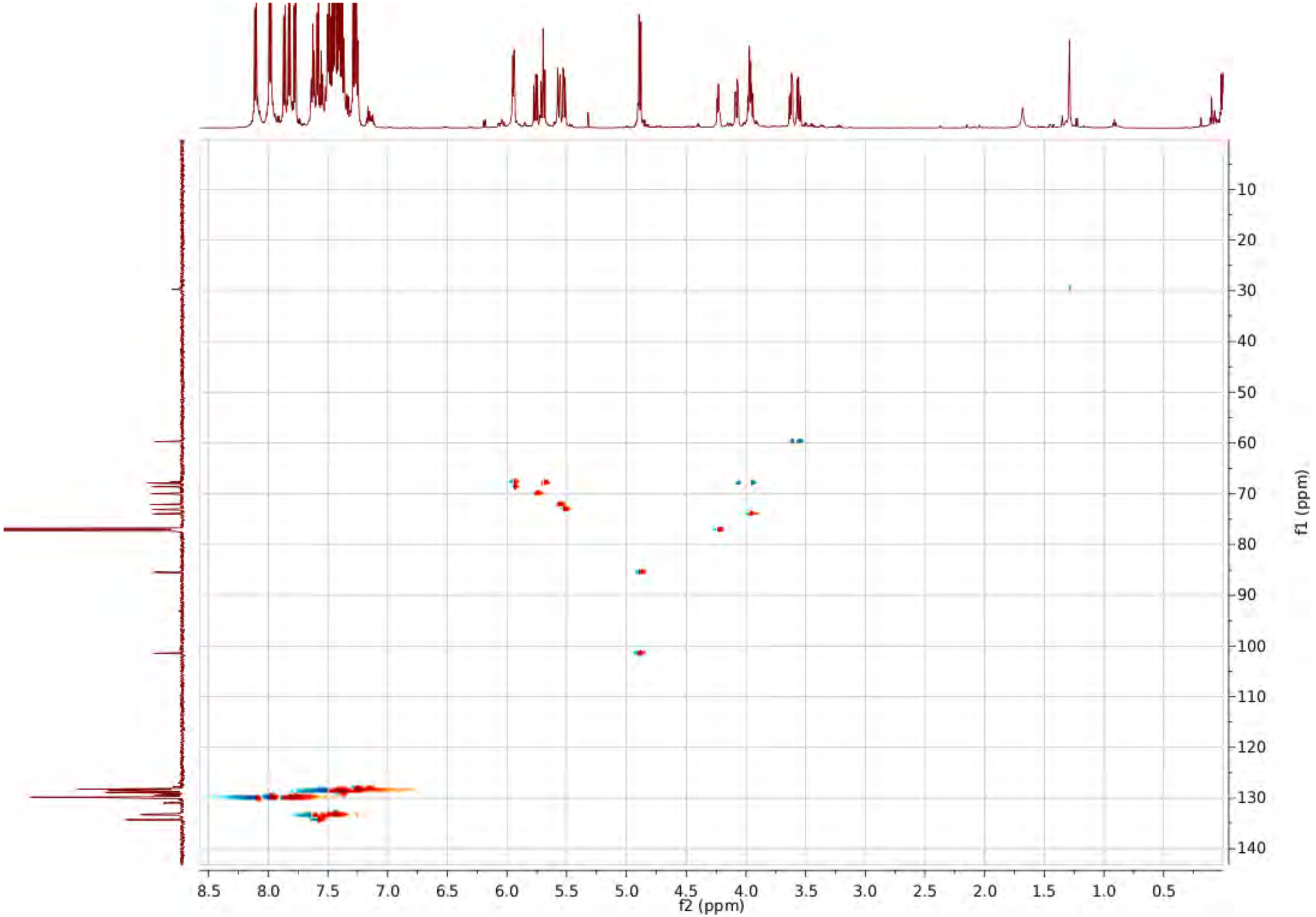
8.5
8.0
7.5
7.0
6.5
6.0
5.5
5.0
4.5
4.0
3.5
3.0
2.5
2.0
1.5
1.0
0.5
f2 (ppm)
10
20
30
40
50
60
70
80
90
100
110
120
130
140
f1 (ppm)

Compound **15$_3$b**

Proton

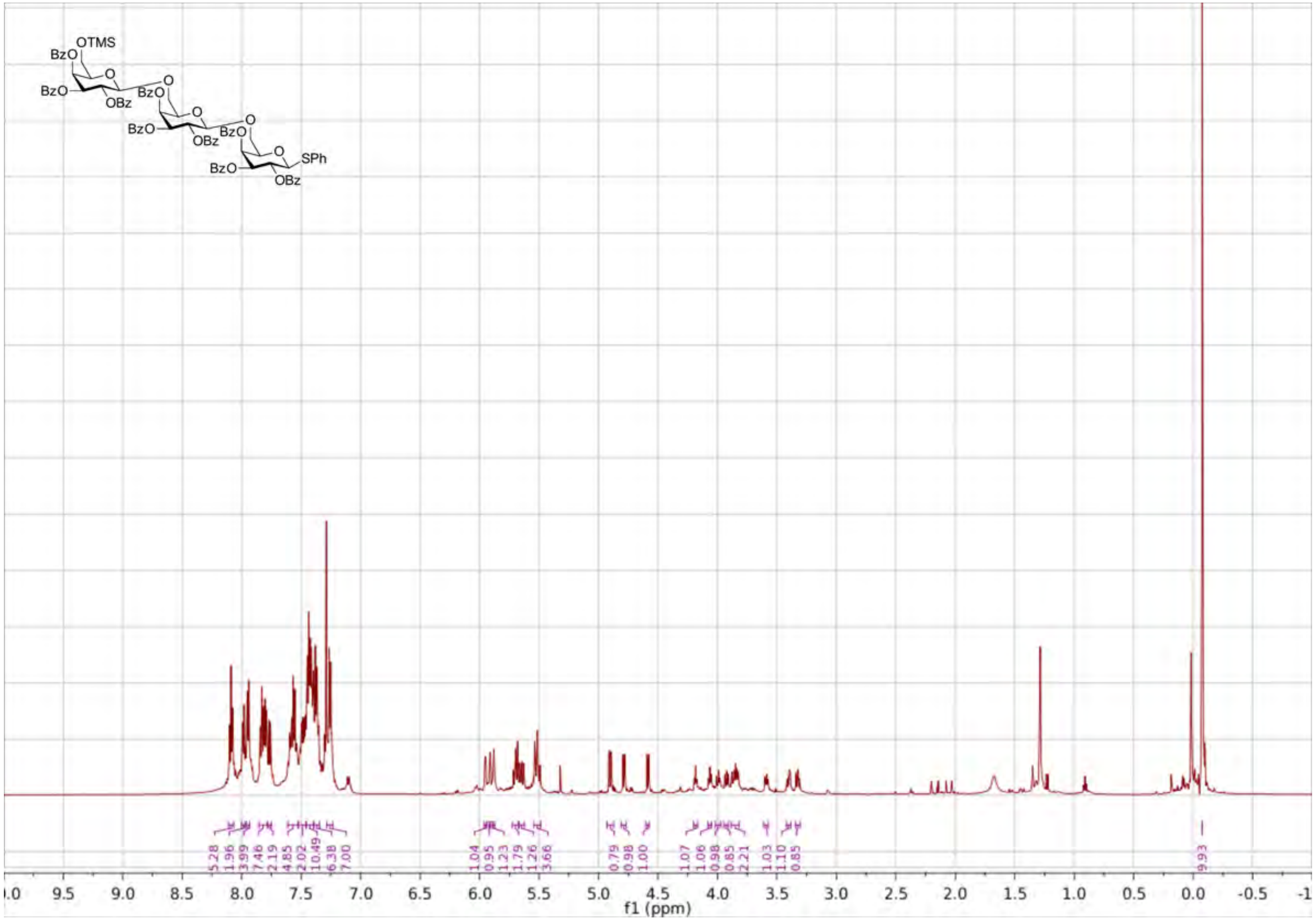

Carbon

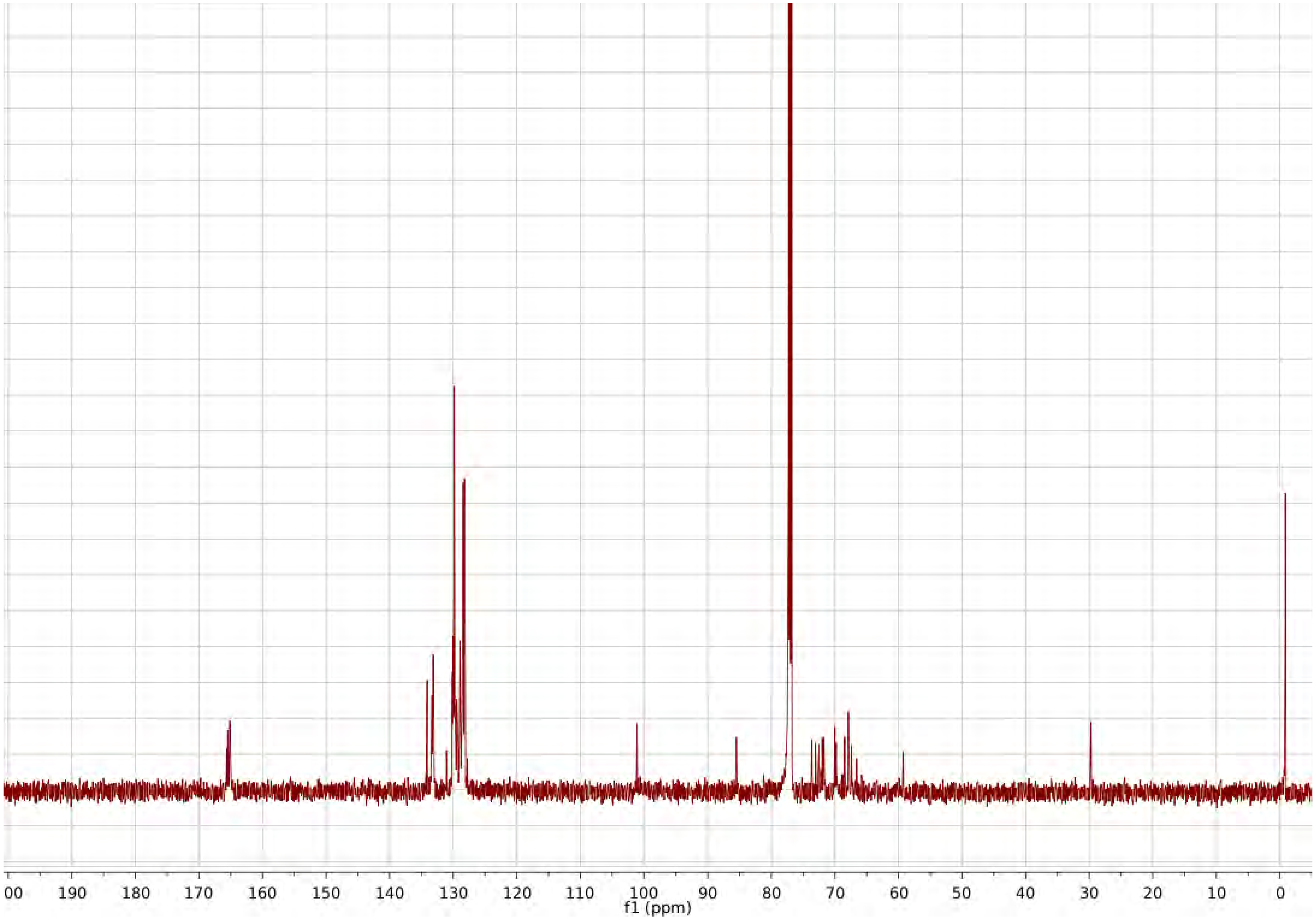
00
190
180
170
160
150
140
130
120
110
100
90
80
70
60
50
40
30
20
10
0
f1 (ppm)

COSY

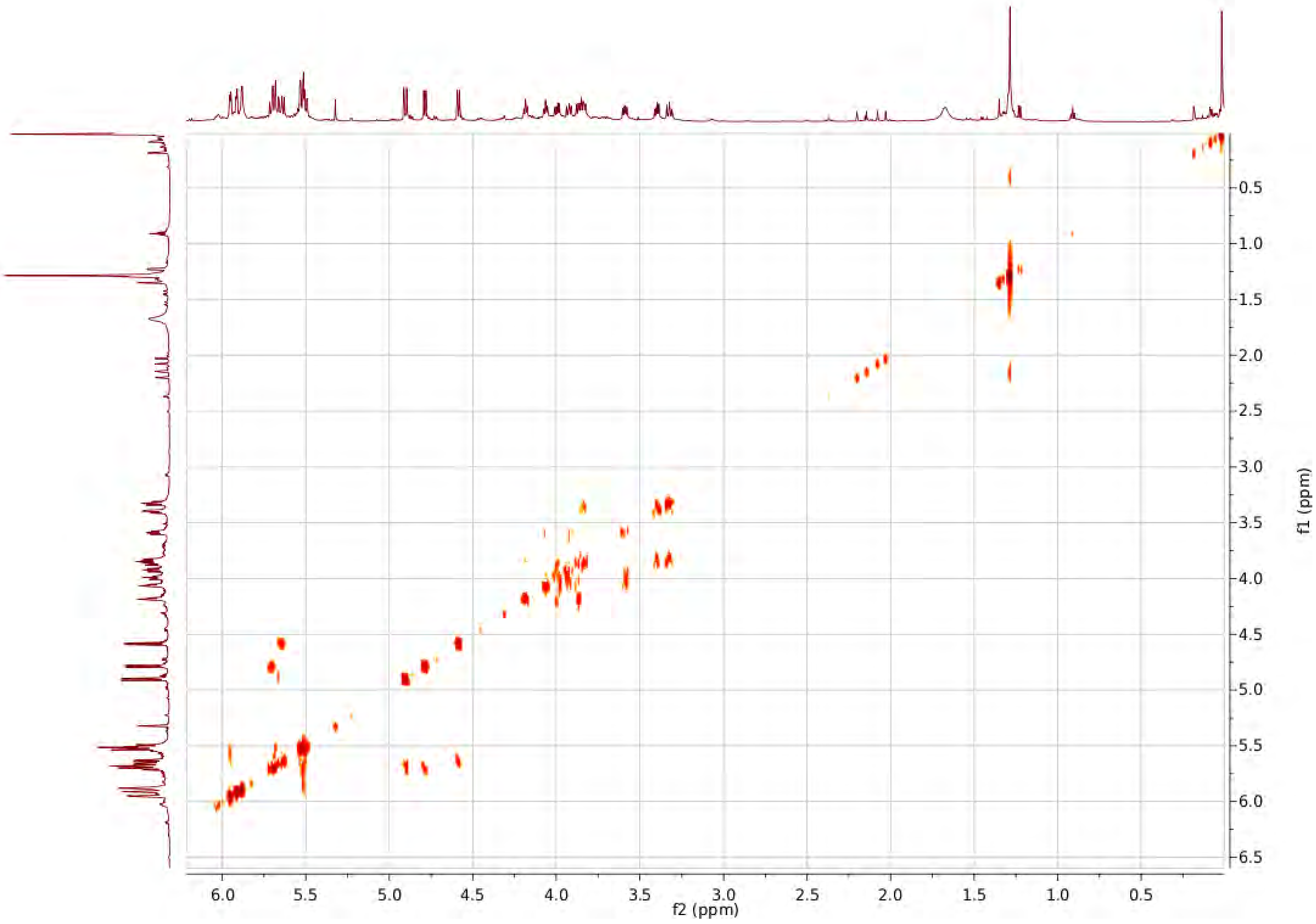

HSQC

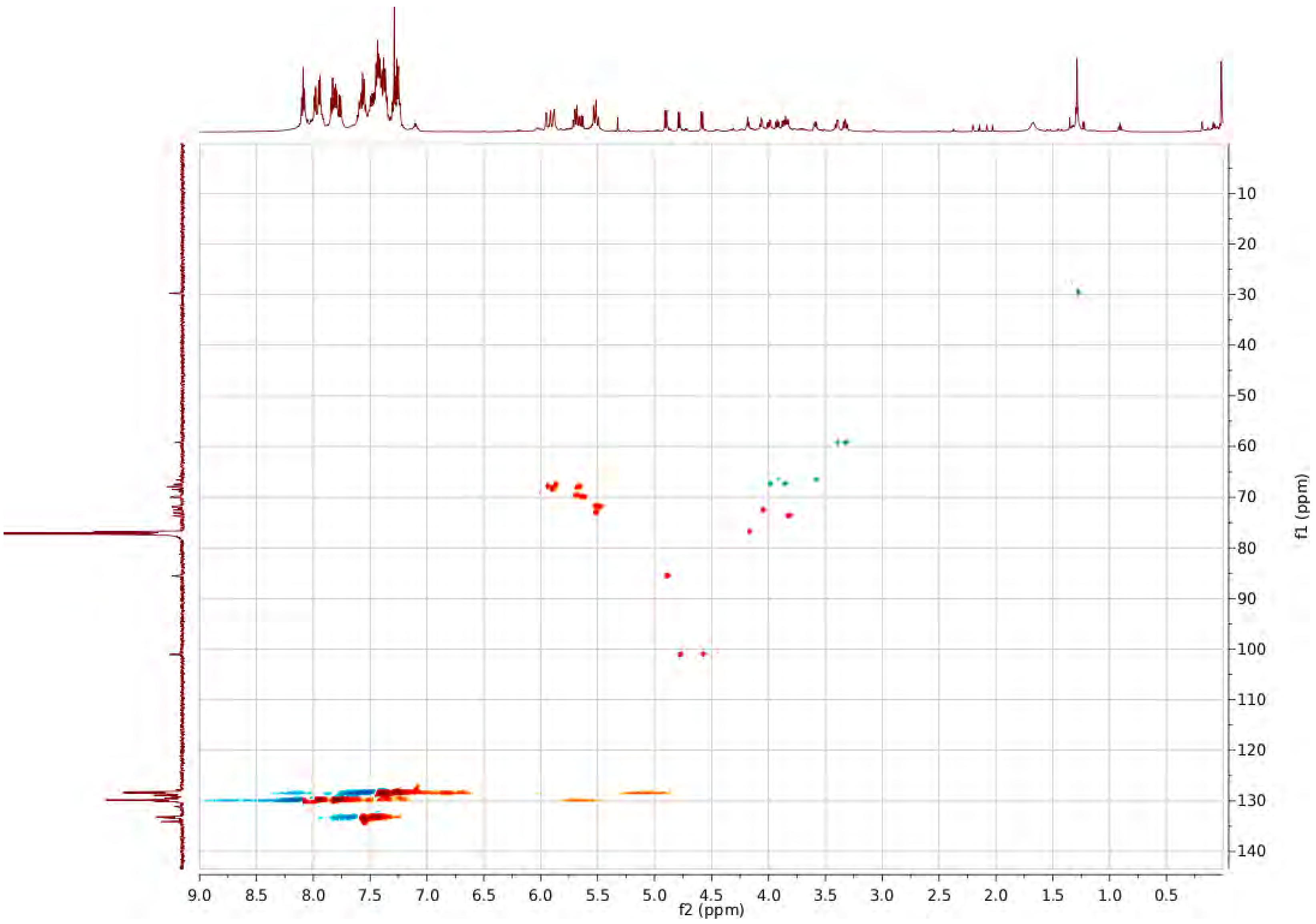
f2 (ppm)
f1 (ppm)
9.0
8.5
8.0
7.5
7.0
6.5
6.0
5.5
5.0
4.5
4.0
3.5
3.0
2.5
2.0
1.5
1.0
0.5
10
20
30
40
50
60
70
80
90
100
110
120
130
140

Compound **15_4b**

Proton

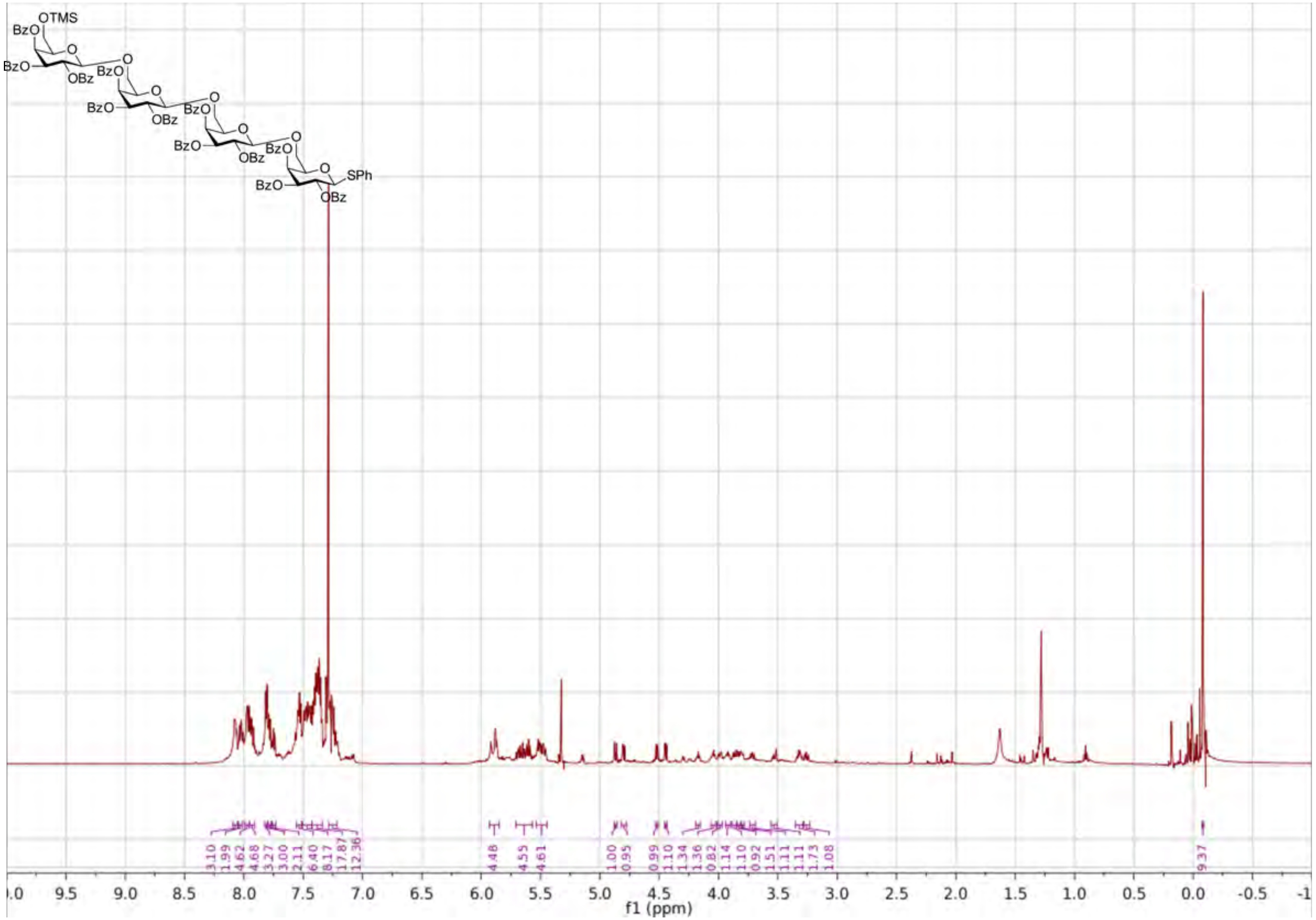

Carbon

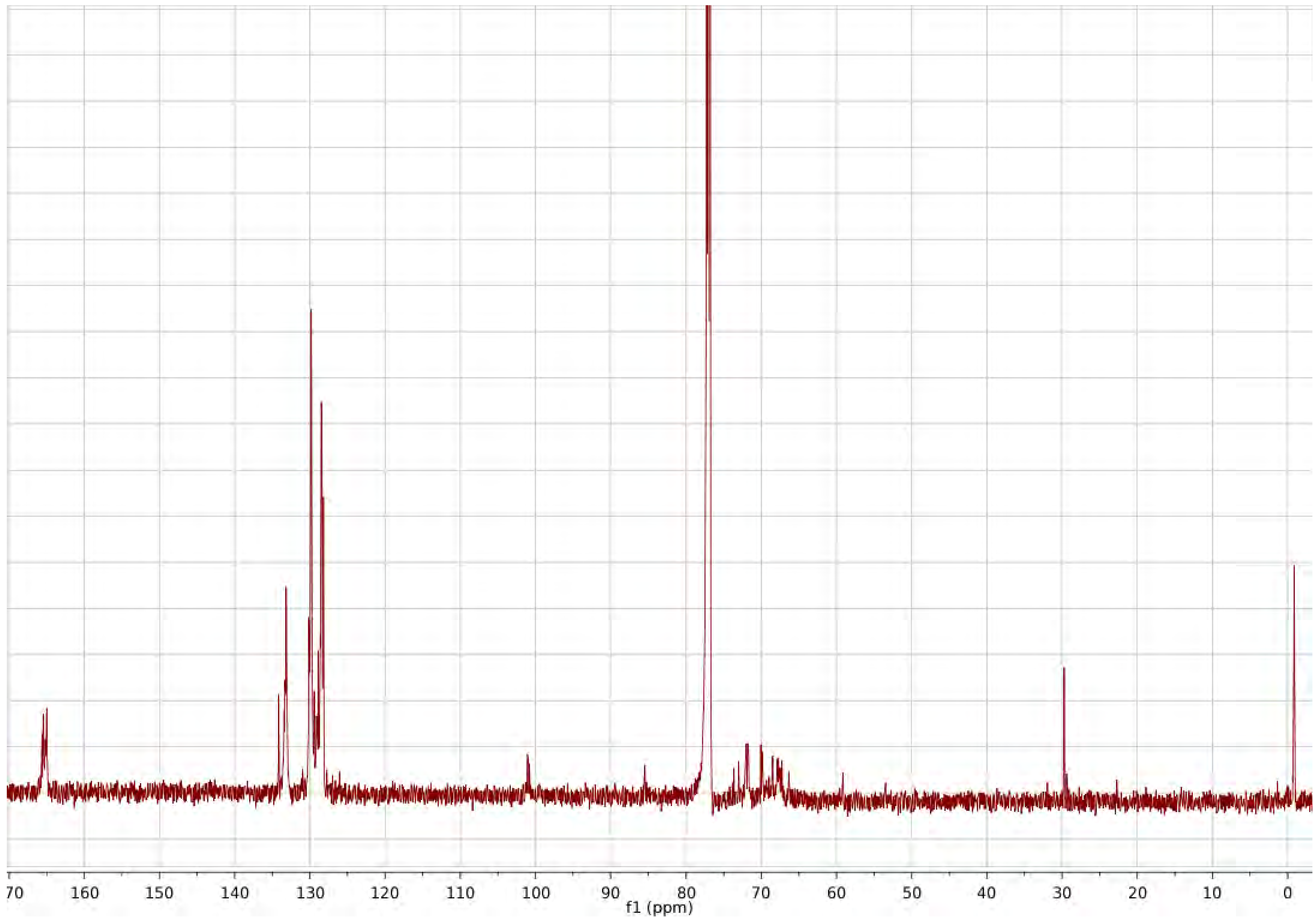

COSY

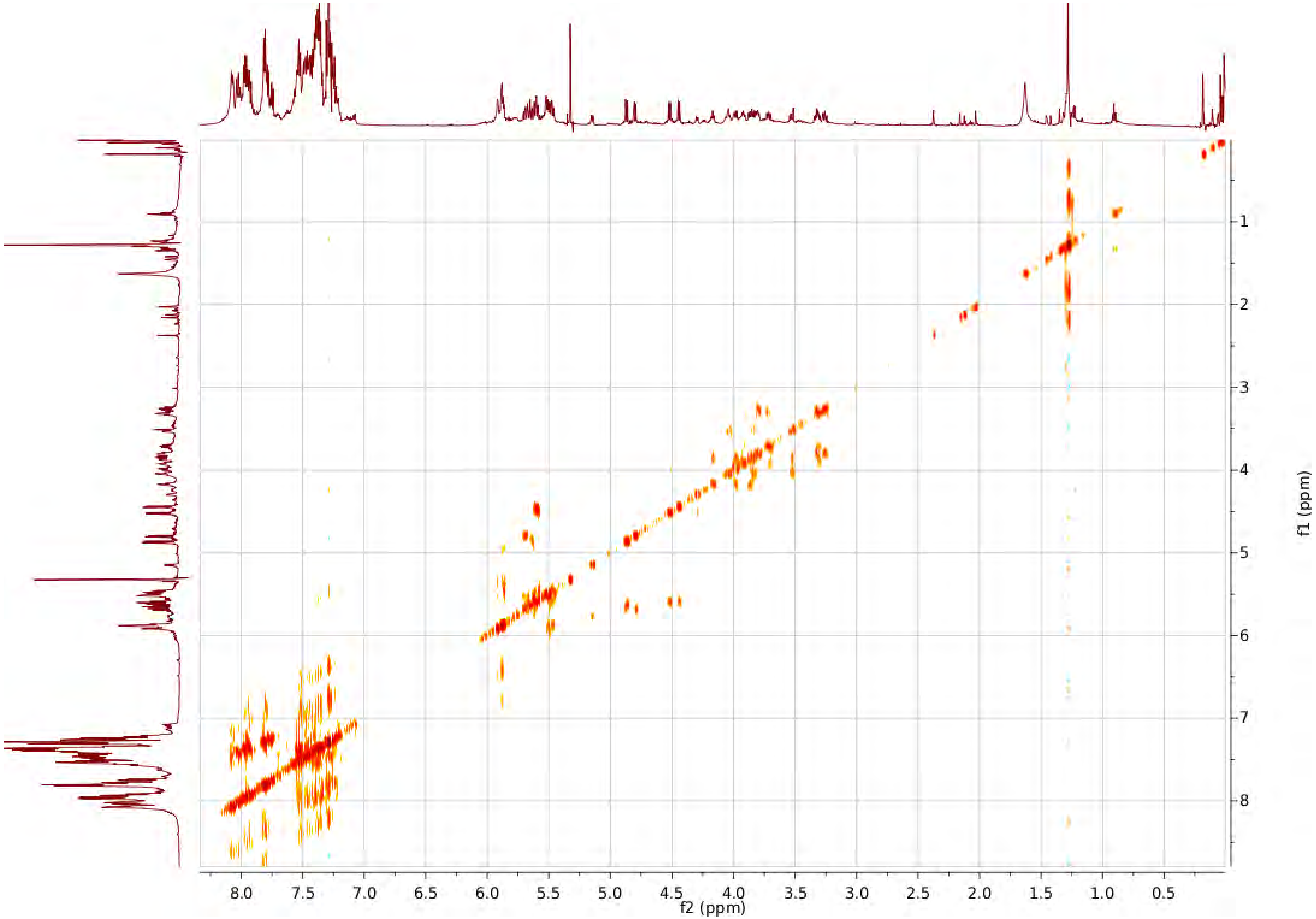

8.0
7.5
7.0
6.5
6.0
5.5
5.0
4.5
4.0
3.5
3.0
2.5
2.0
1.5
1.0
0.5
f2 (ppm)
1
2
3
4
5
6
7
8
f1 (ppm)

HSQC

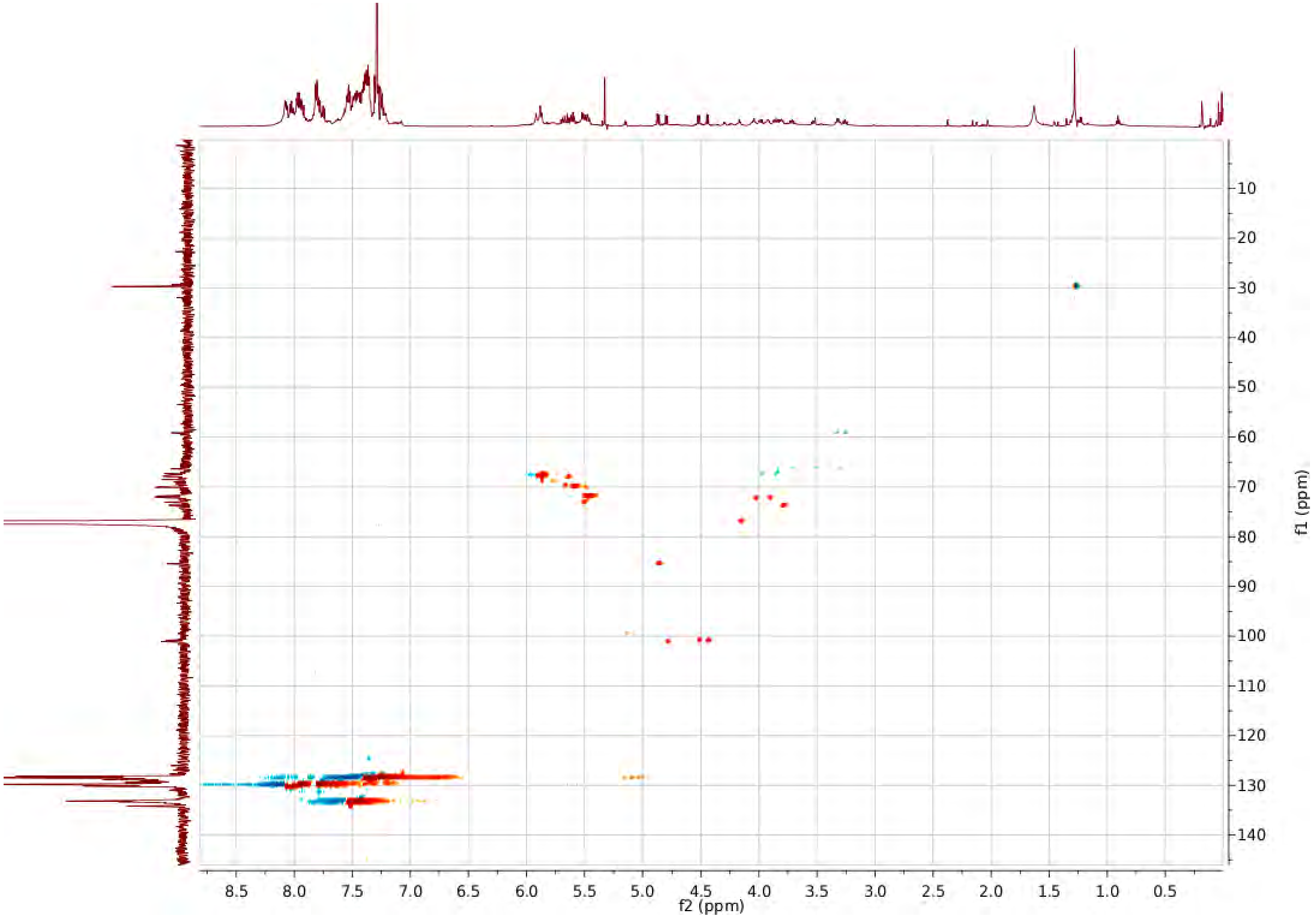

f1 (ppm)
f2 (ppm)
10
20
30
40
50
60
70
80
90
100
110
120
130
140
8.5
8.0
7.5
7.0
6.5
6.0
5.5
5.0
4.5
4.0
3.5
3.0
2.5
2.0
1.5
1.0
0.5

Compound **15$_2$c**

Proton

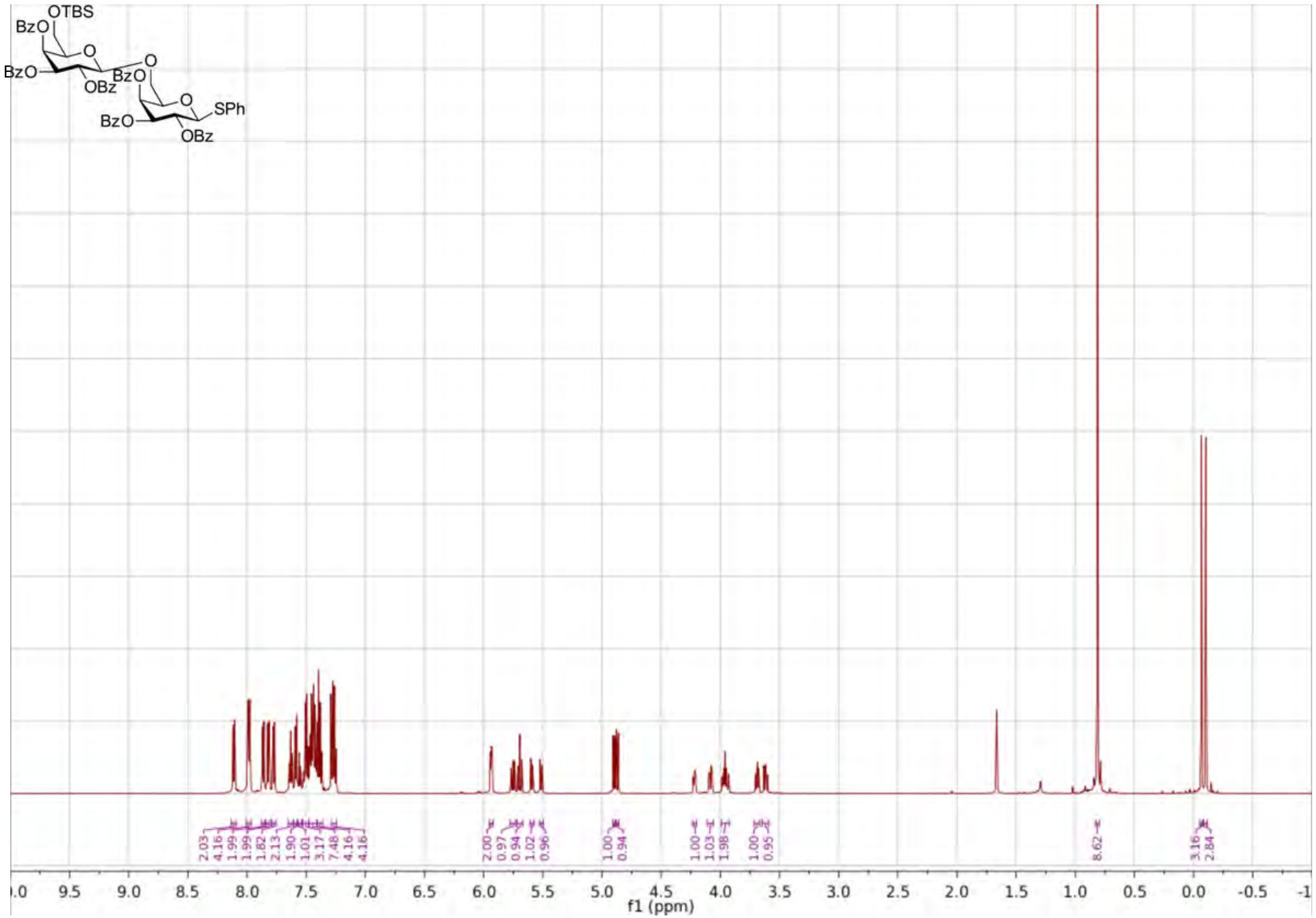

Carbon

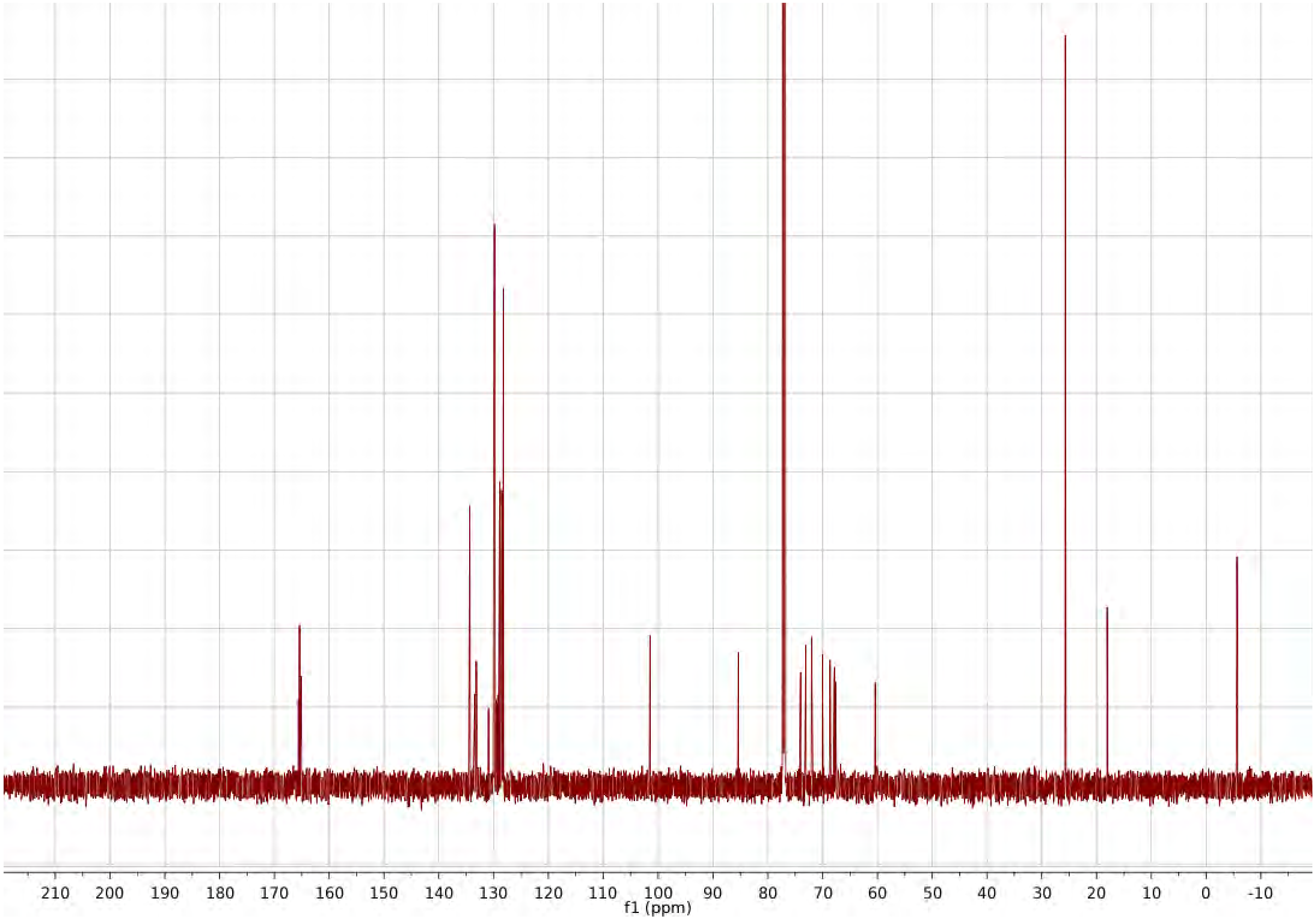
210
200
190
180
170
160
150
140
130
120
110
100
90
80
70
60
50
40
30
20
10
0
-10
f1 (ppm)

COSY

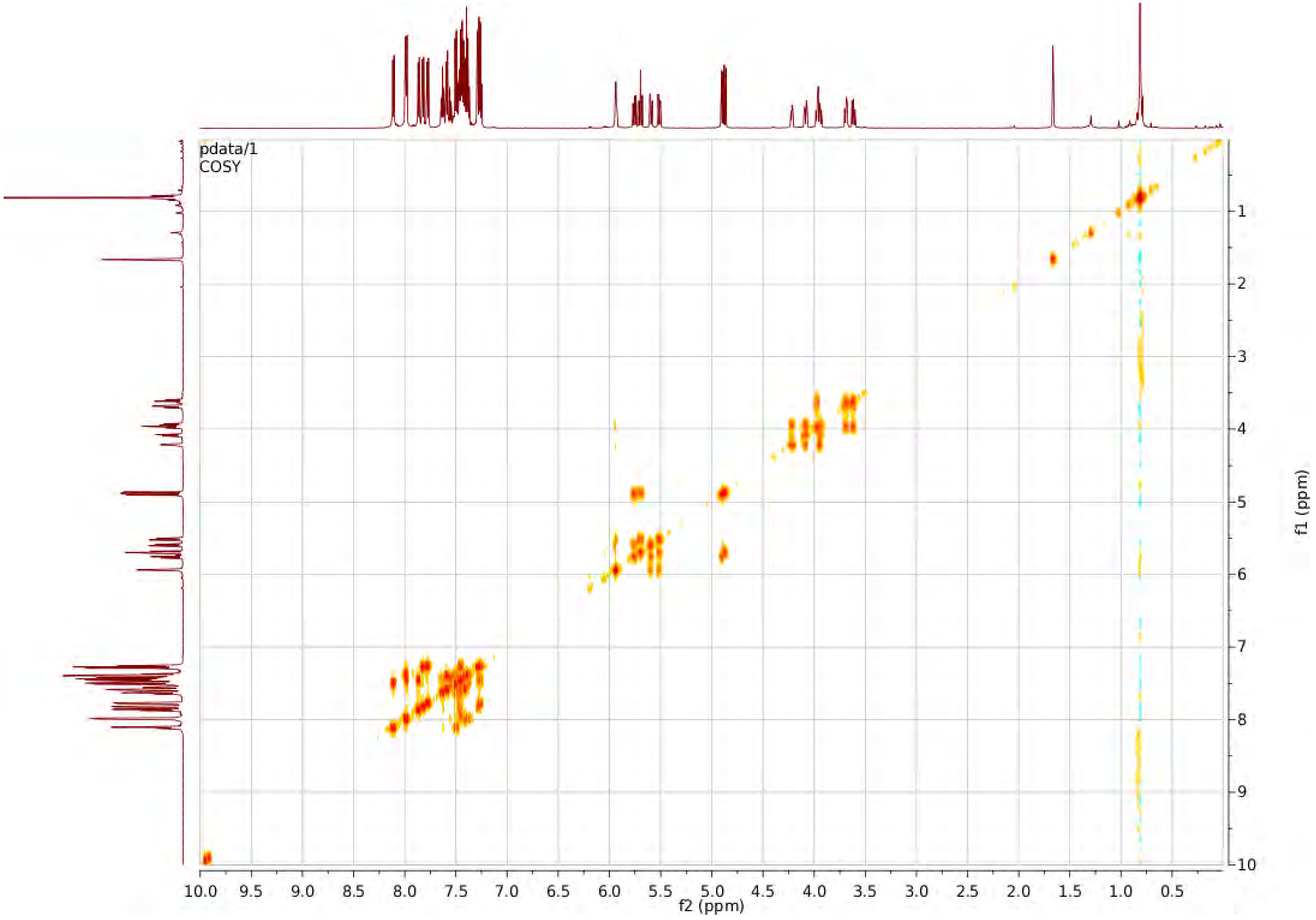

HSQC

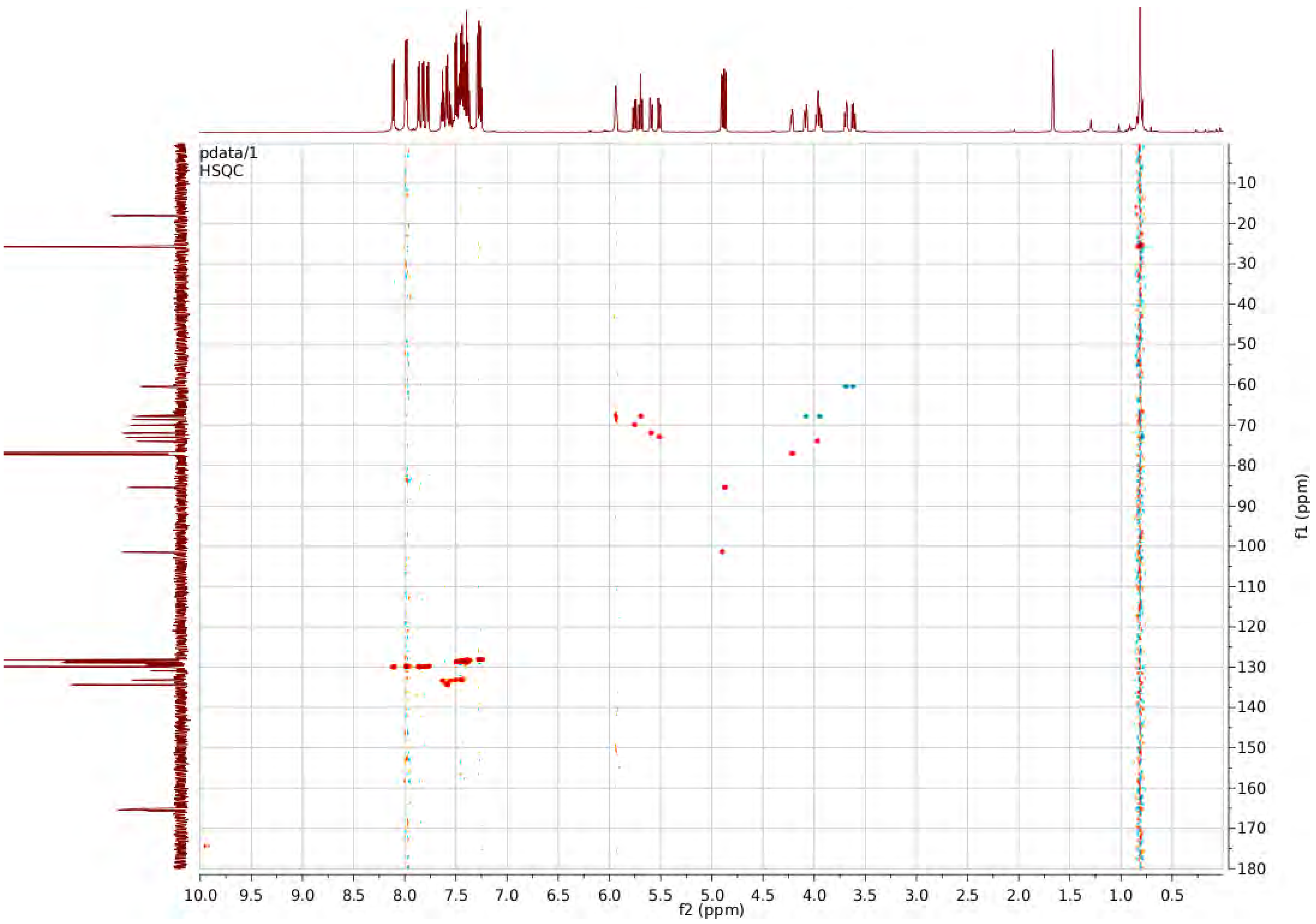
pdata/1
HSQC
f2 (ppm)
f1 (ppm)
10.0 9.5 9.0 8.5 8.0 7.5 7.0 6.5 6.0 5.5 5.0 4.5 4.0 3.5 3.0 2.5 2.0 1.5 1.0 0.5
10 20 30 40 50 60 70 80 90 100 110 120 130 140 150 160 170 180

Compound **15$_3$c**

Proton

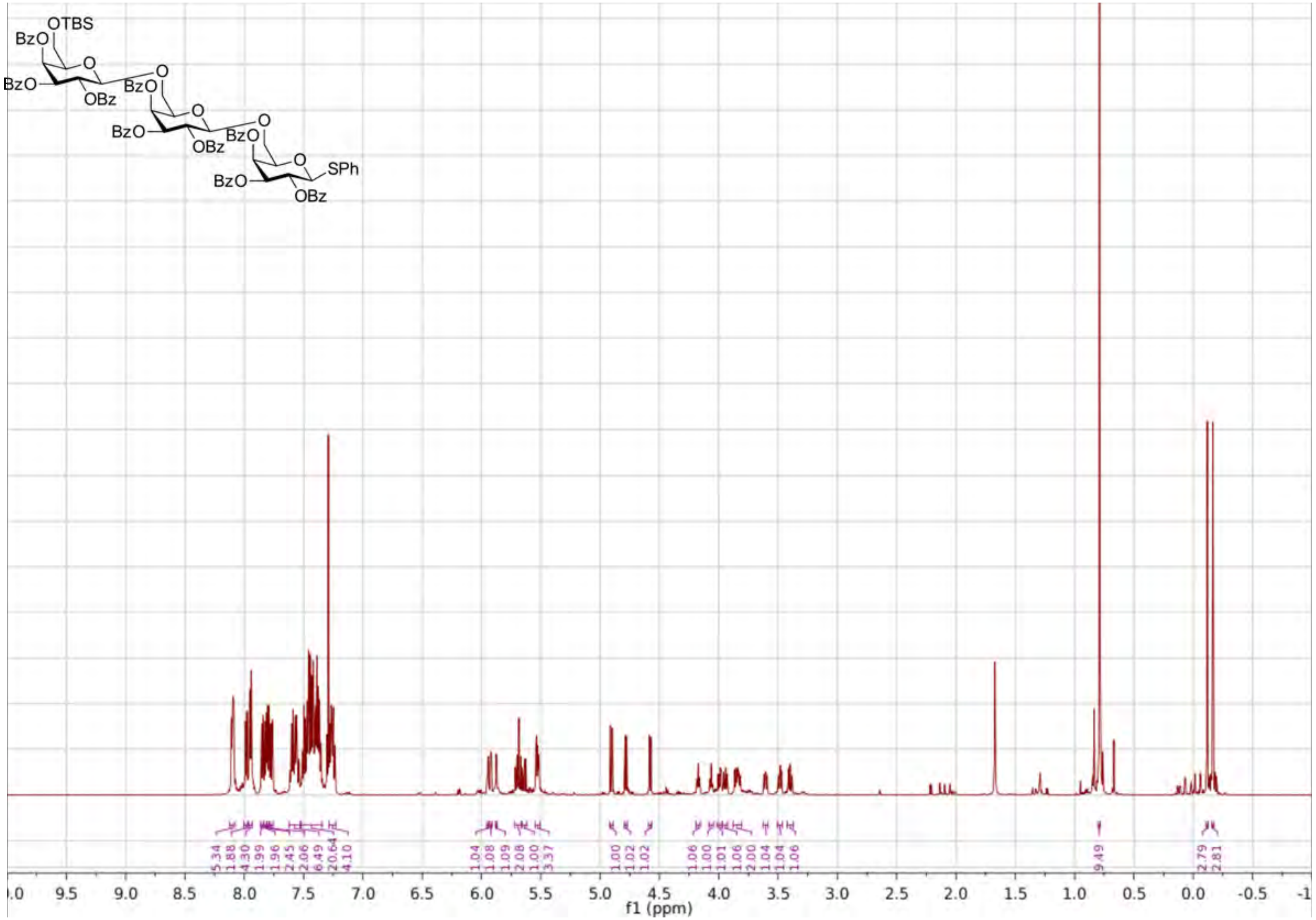

Carbon

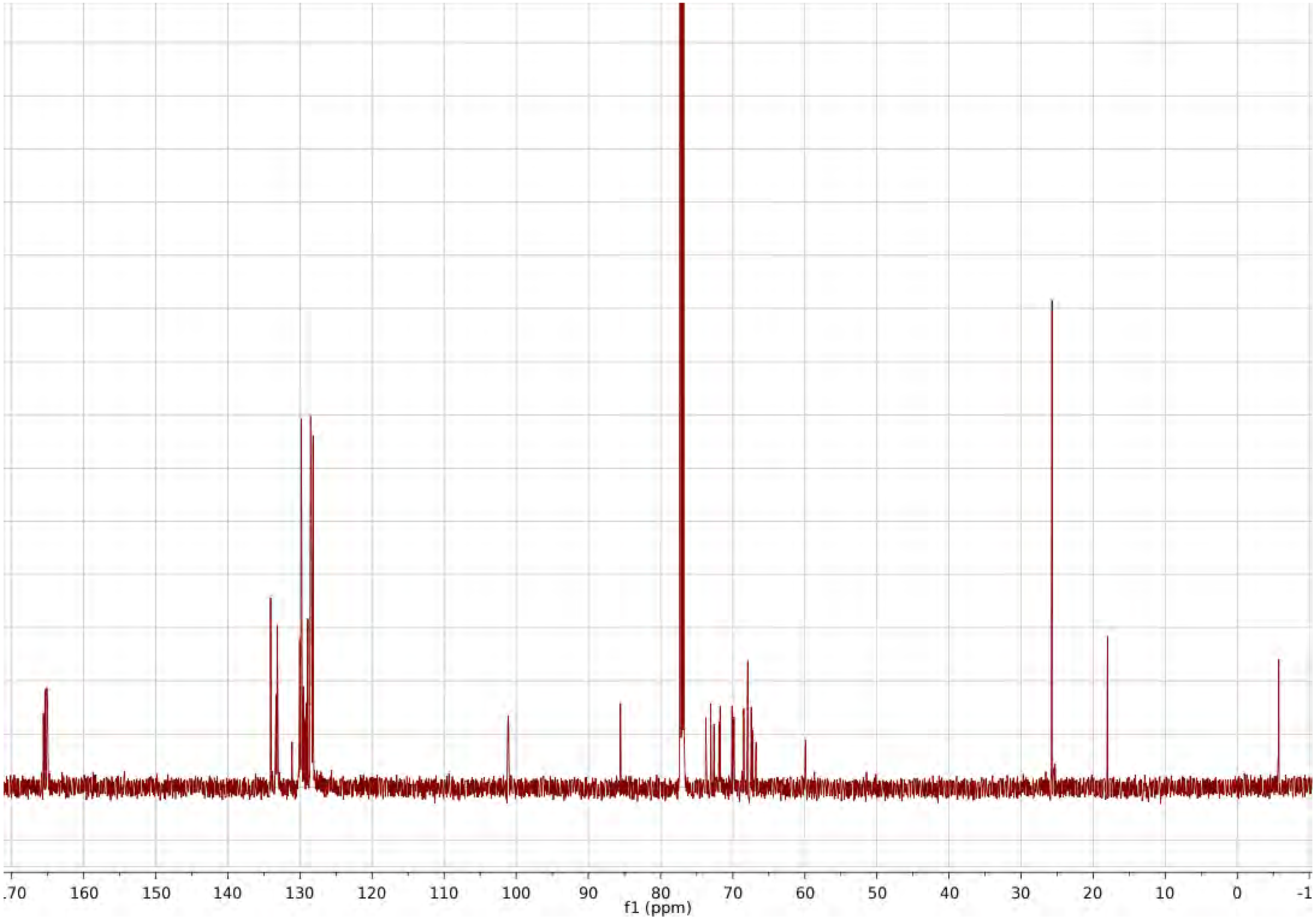

COSY

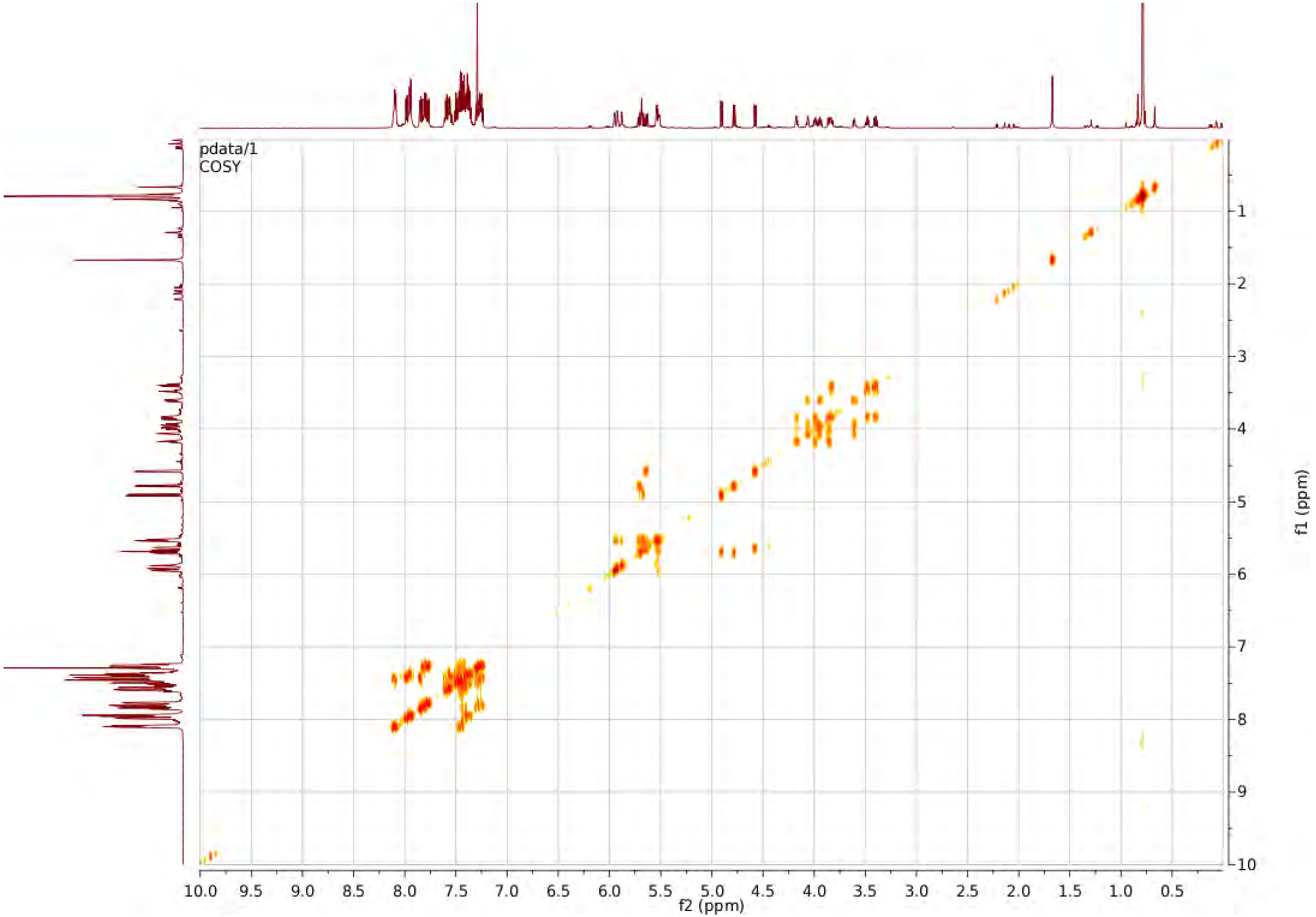
pdata/1
COSY
f2 (ppm)
f1 (ppm)
10.0
9.5
9.0
8.5
8.0
7.5
7.0
6.5
6.0
5.5
5.0
4.5
4.0
3.5
3.0
2.5
2.0
1.5
1.0
0.5
1
2
3
4
5
6
7
8
9
10

HSQC

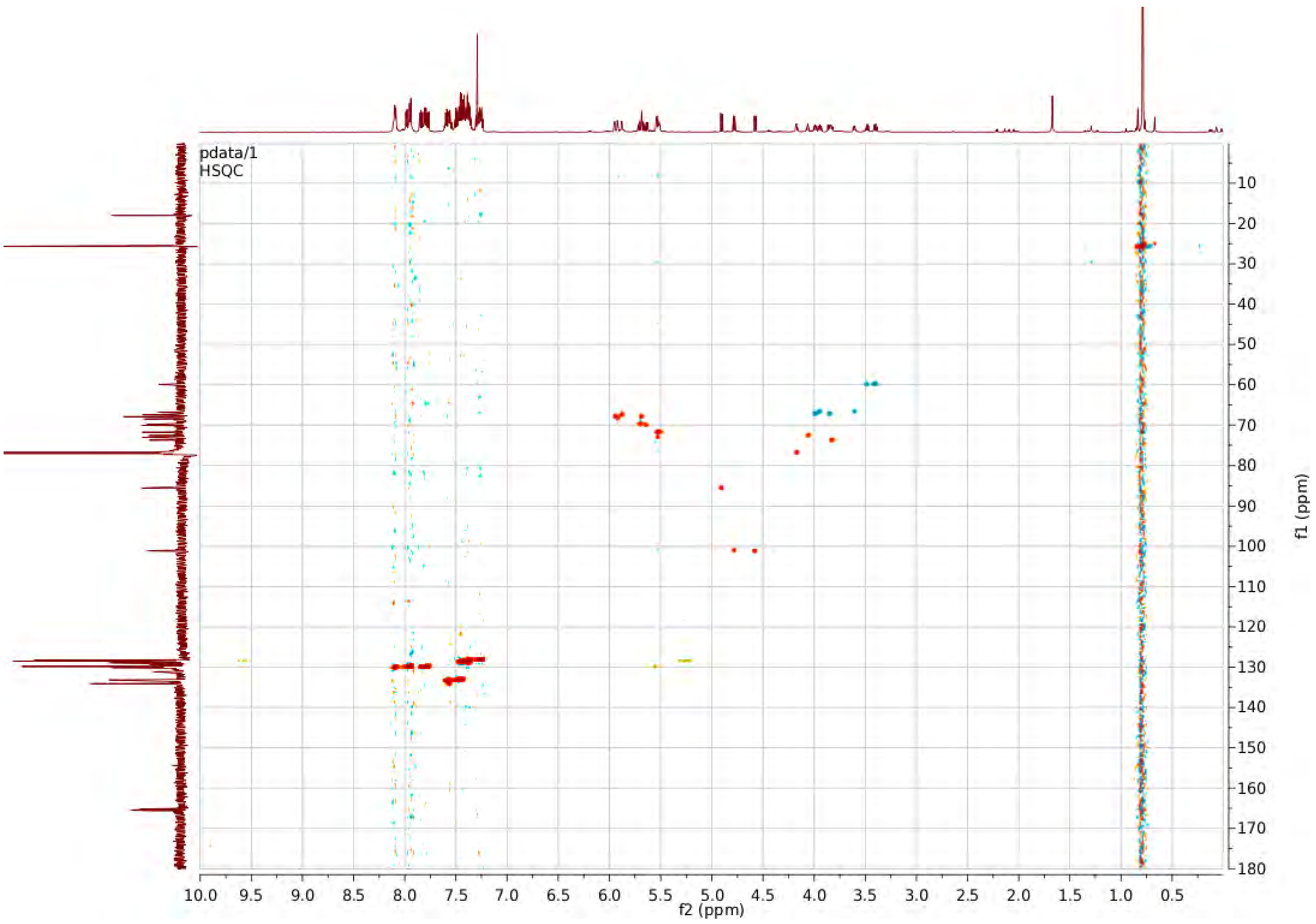

Compound **15$_4$c**

Proton

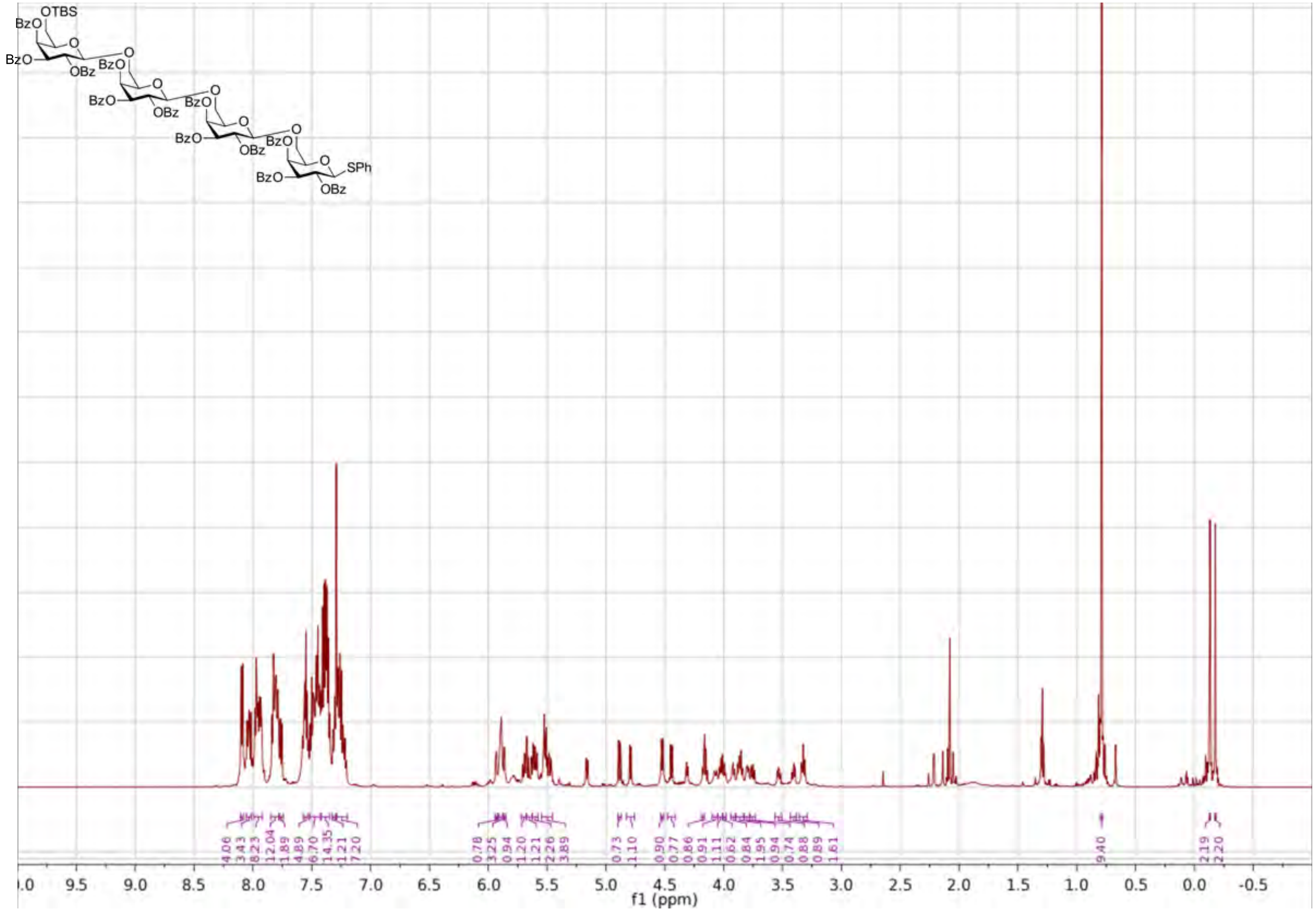

Carbon

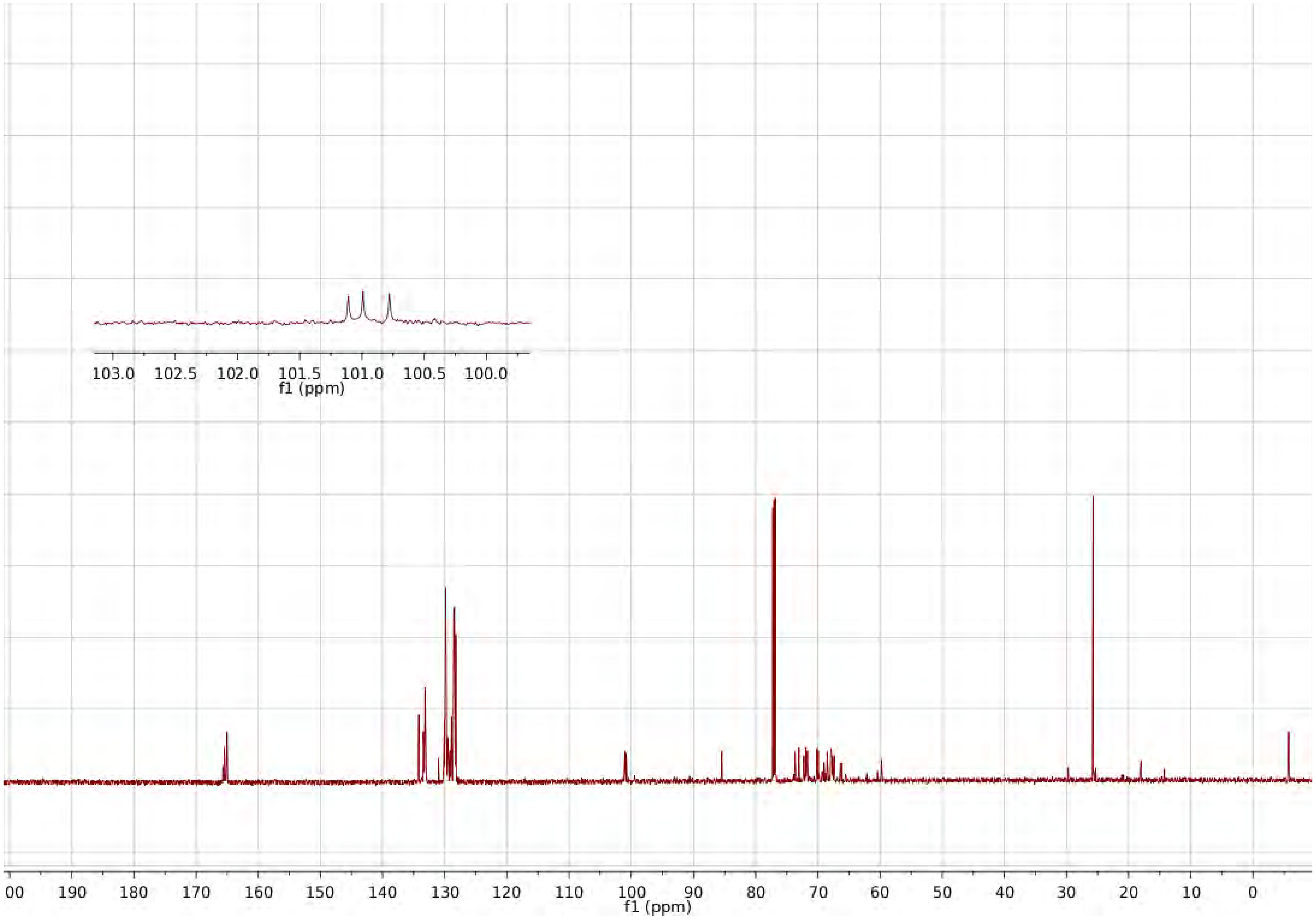
103.0
102.5
102.0
101.5
101.0
100.5
100.0
f1 (ppm)
00
190
180
170
160
150
140
130
120
110
100
90
80
70
60
50
40
30
20
10
0
f1 (ppm)

COSY

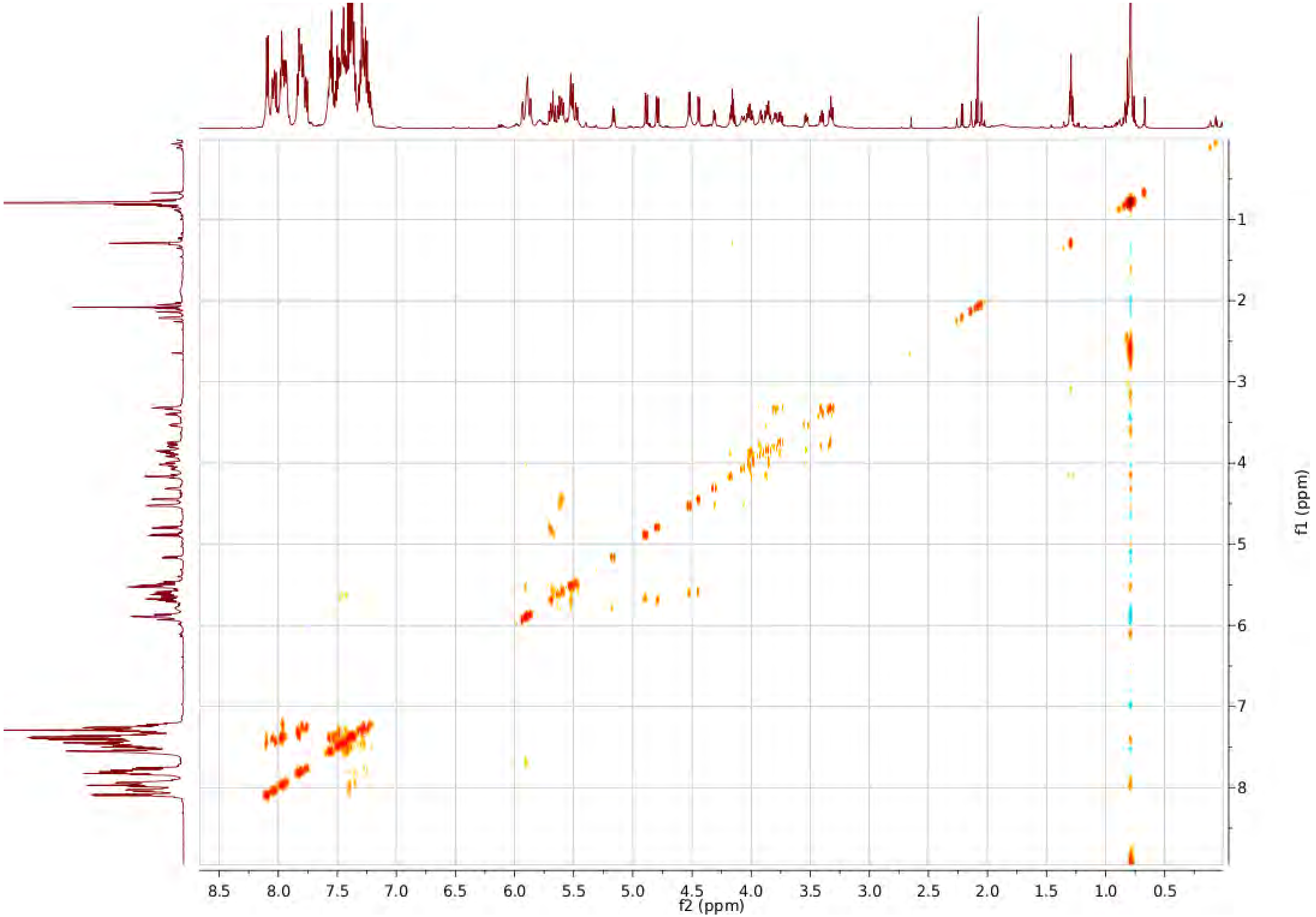

8.5
8.0
7.5
7.0
6.5
6.0
5.5
5.0
4.5
4.0
3.5
3.0
2.5
2.0
1.5
1.0
0.5
f2 (ppm)
1
2
3
4
5
6
7
8
f1 (ppm)

HSQC

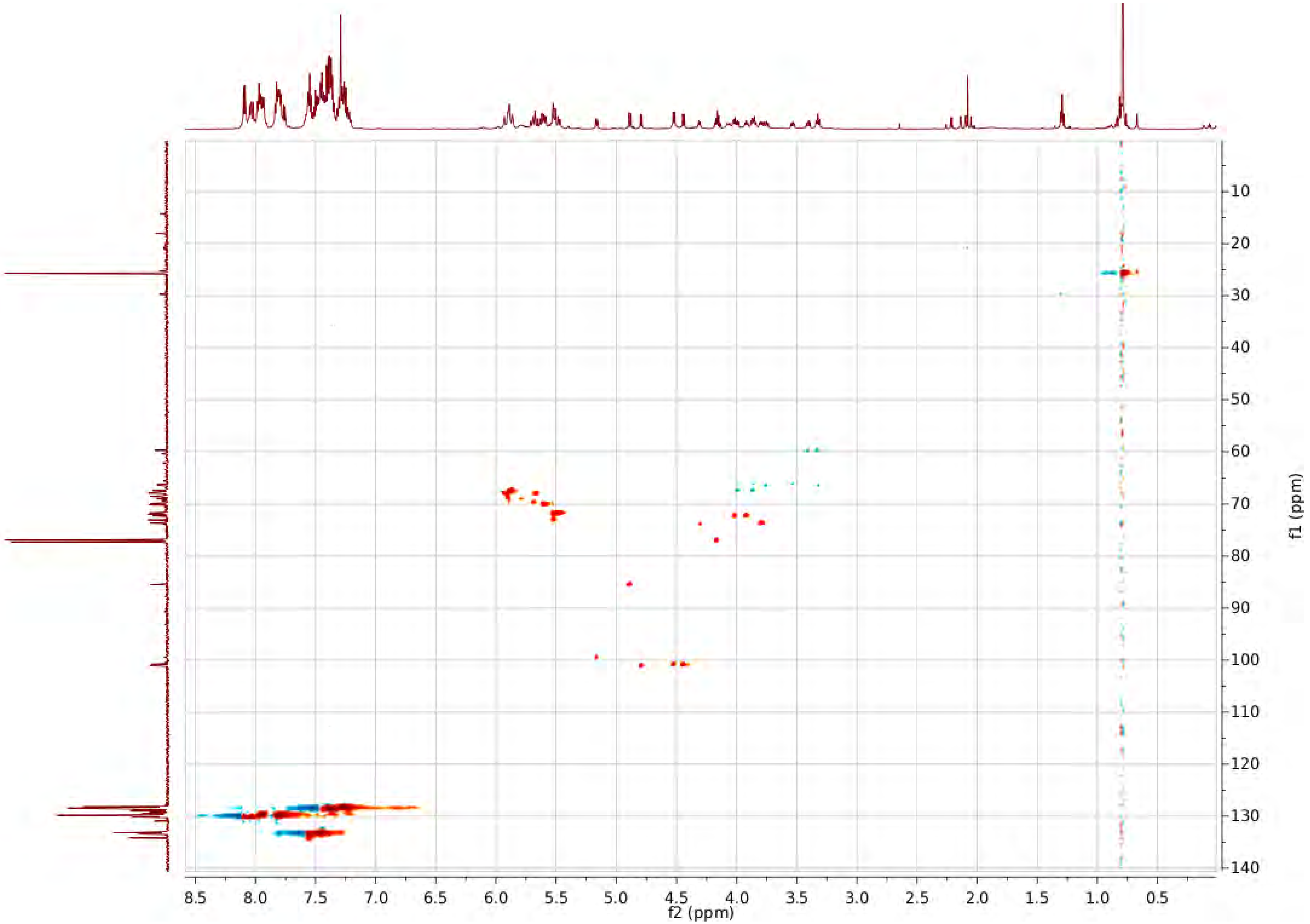

Compound **15₅c**

Proton

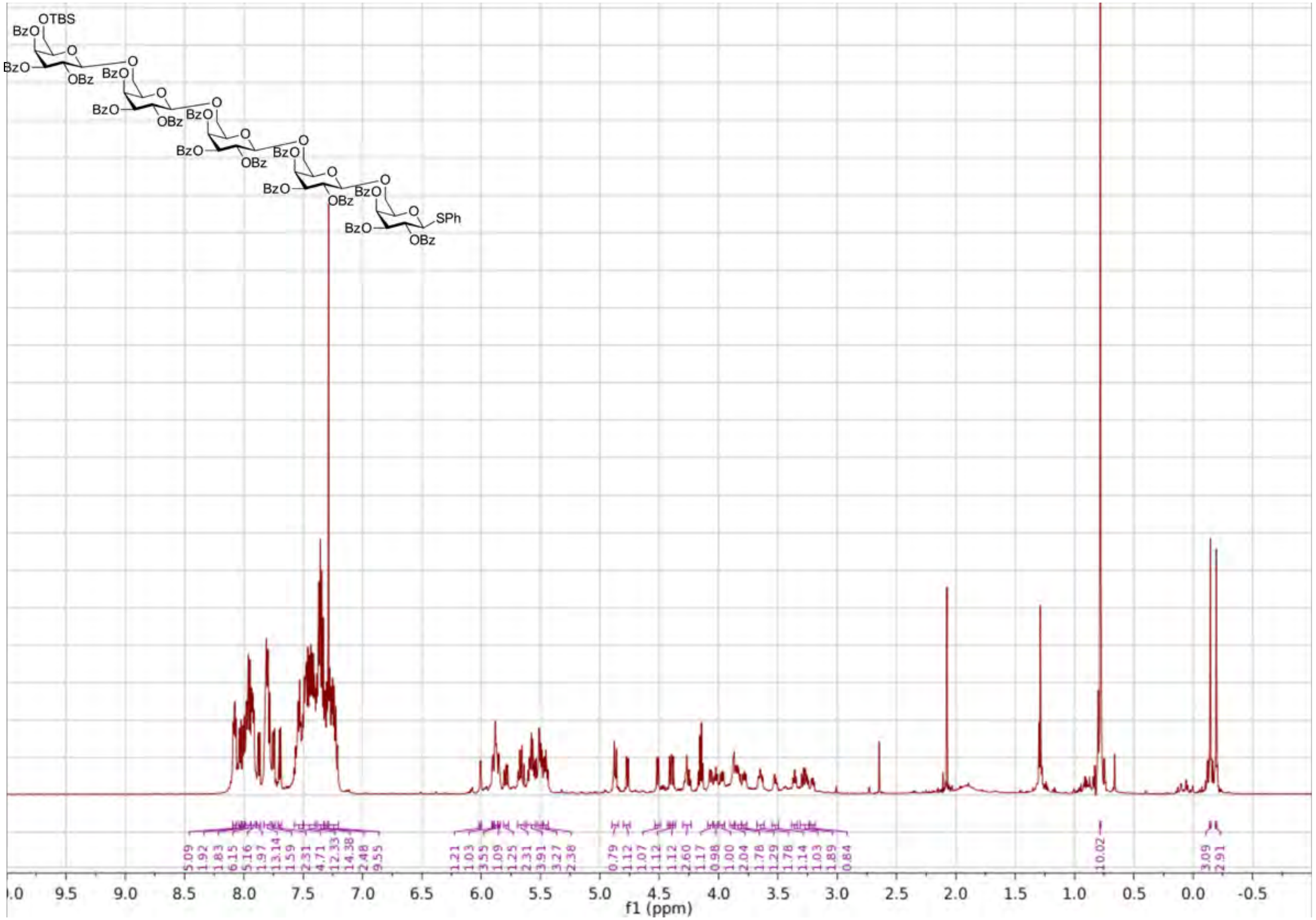

Carbon

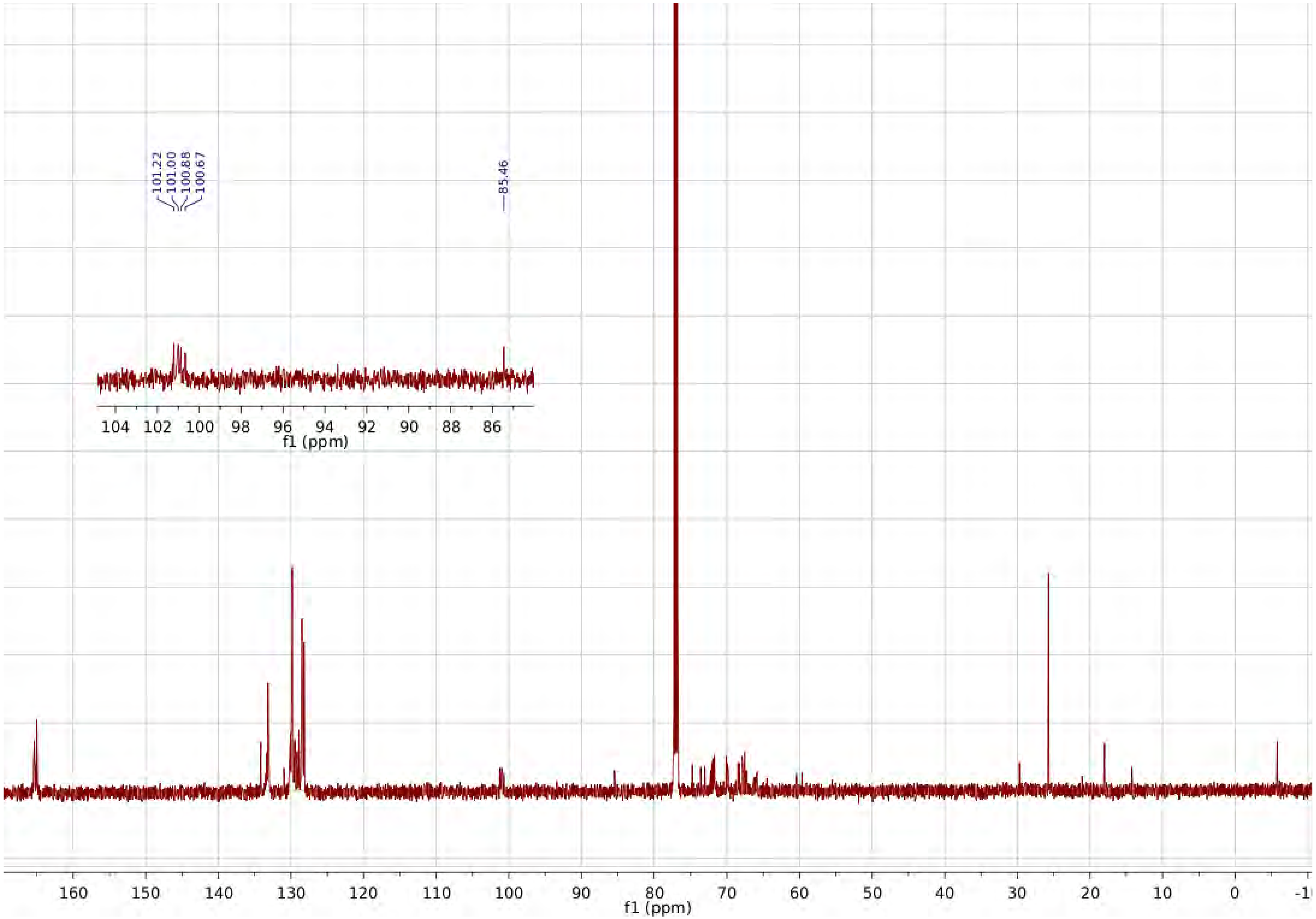

101.22
101.00
100.88
100.67
85.46
104
102
100
98
96
94
92
90
88
86
f1 (ppm)
160
150
140
130
120
110
100
90
80
70
60
50
40
30
20
10
0
f1 (ppm)

Compound **16₁c**

Proton

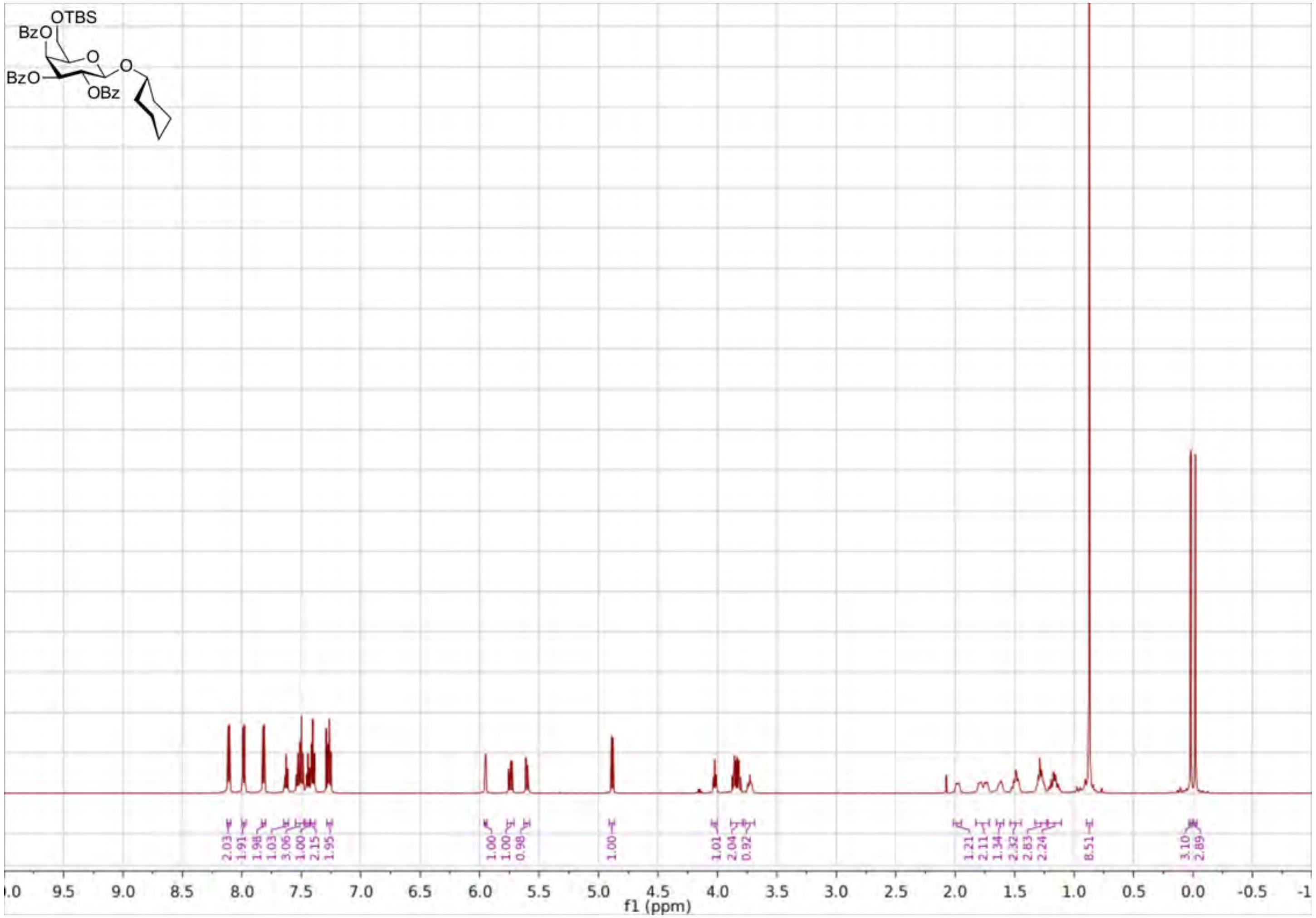

Carbon

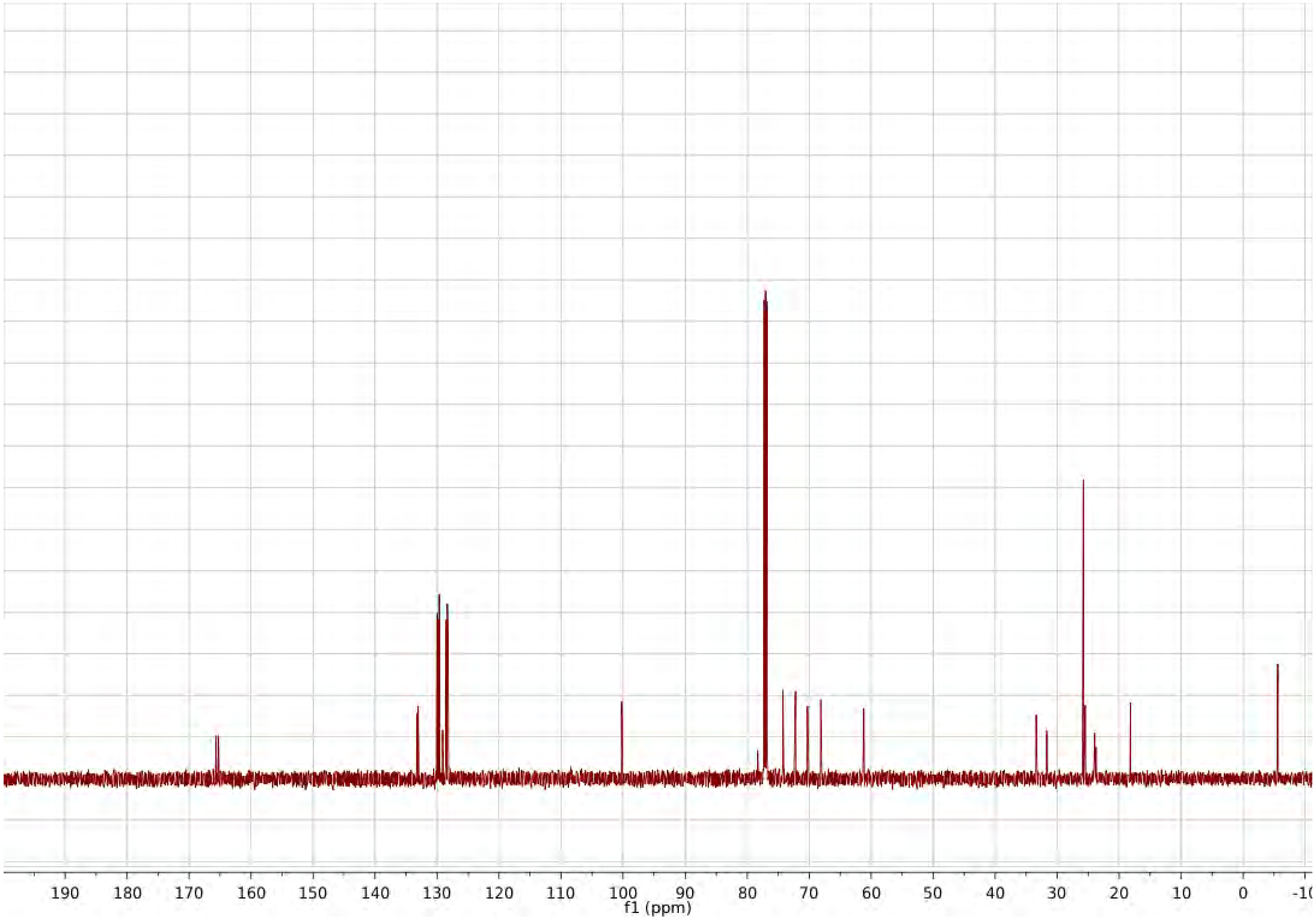
190
180
170
160
150
140
130
120
110
100
90
80
70
60
50
40
30
20
10
0
f1 (ppm)

COSY

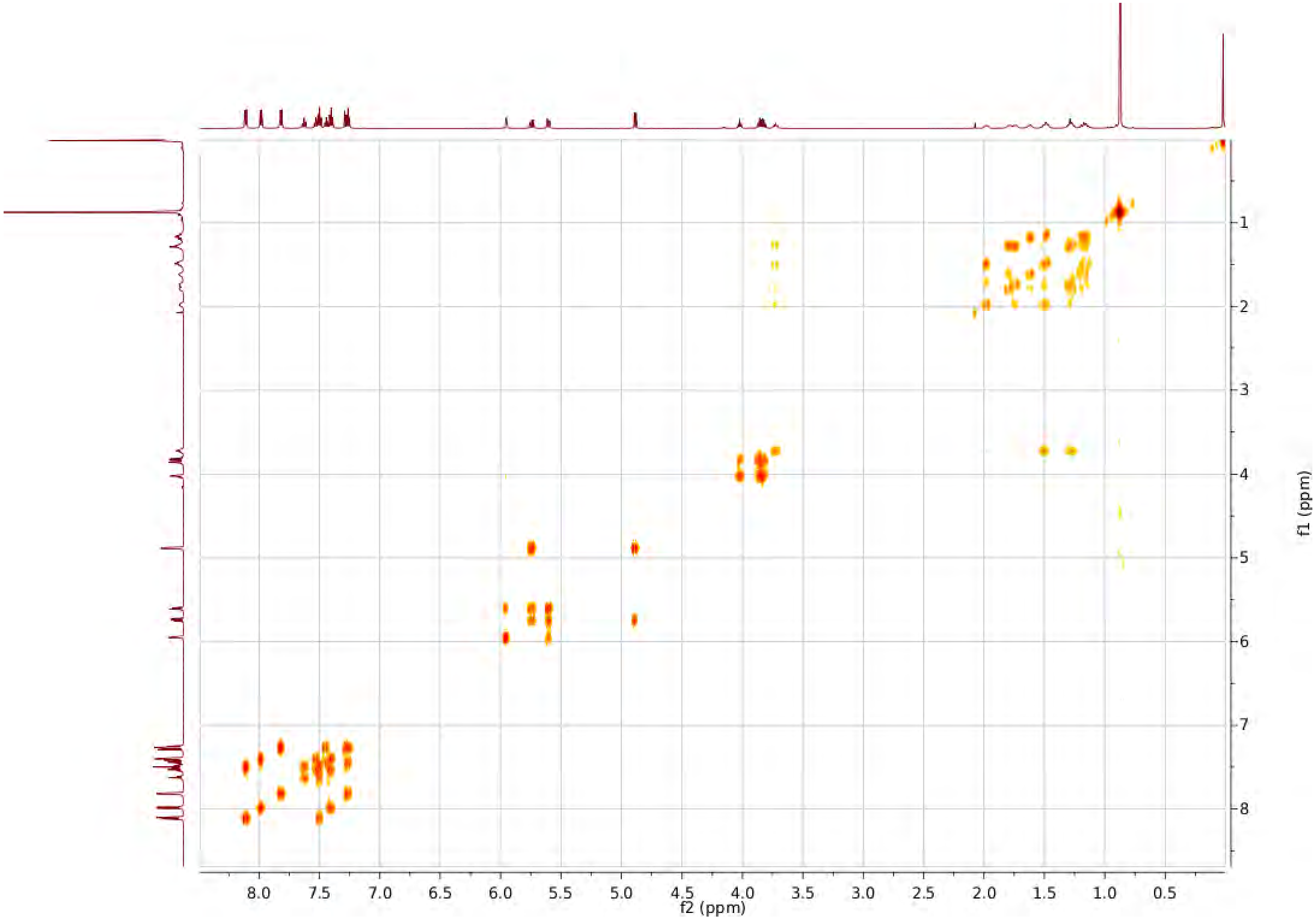
f2 (ppm)
f1 (ppm)
8.0
7.5
7.0
6.5
6.0
5.5
5.0
4.5
4.0
3.5
3.0
2.5
2.0
1.5
1.0
0.5
1
2
3
4
5
6
7
8

HSQC

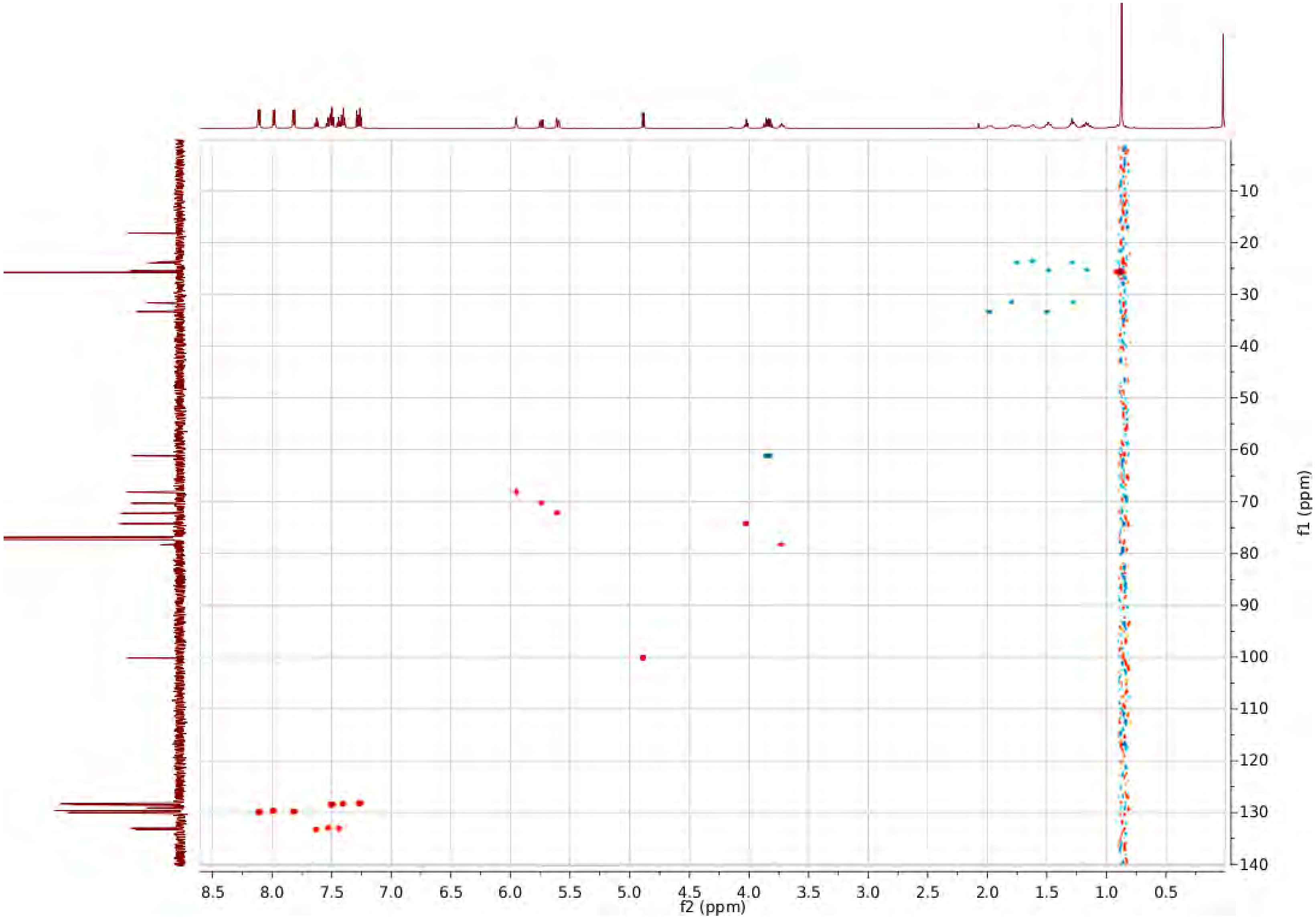

8.5
8.0
7.5
7.0
6.5
6.0
5.5
5.0
4.5
4.0
3.5
3.0
2.5
2.0
1.5
1.0
0.5
f2 (ppm)
10
20
30
40
50
60
70
80
90
100
110
120
130
140
f1 (ppm)

Compound **16₂c**

Proton

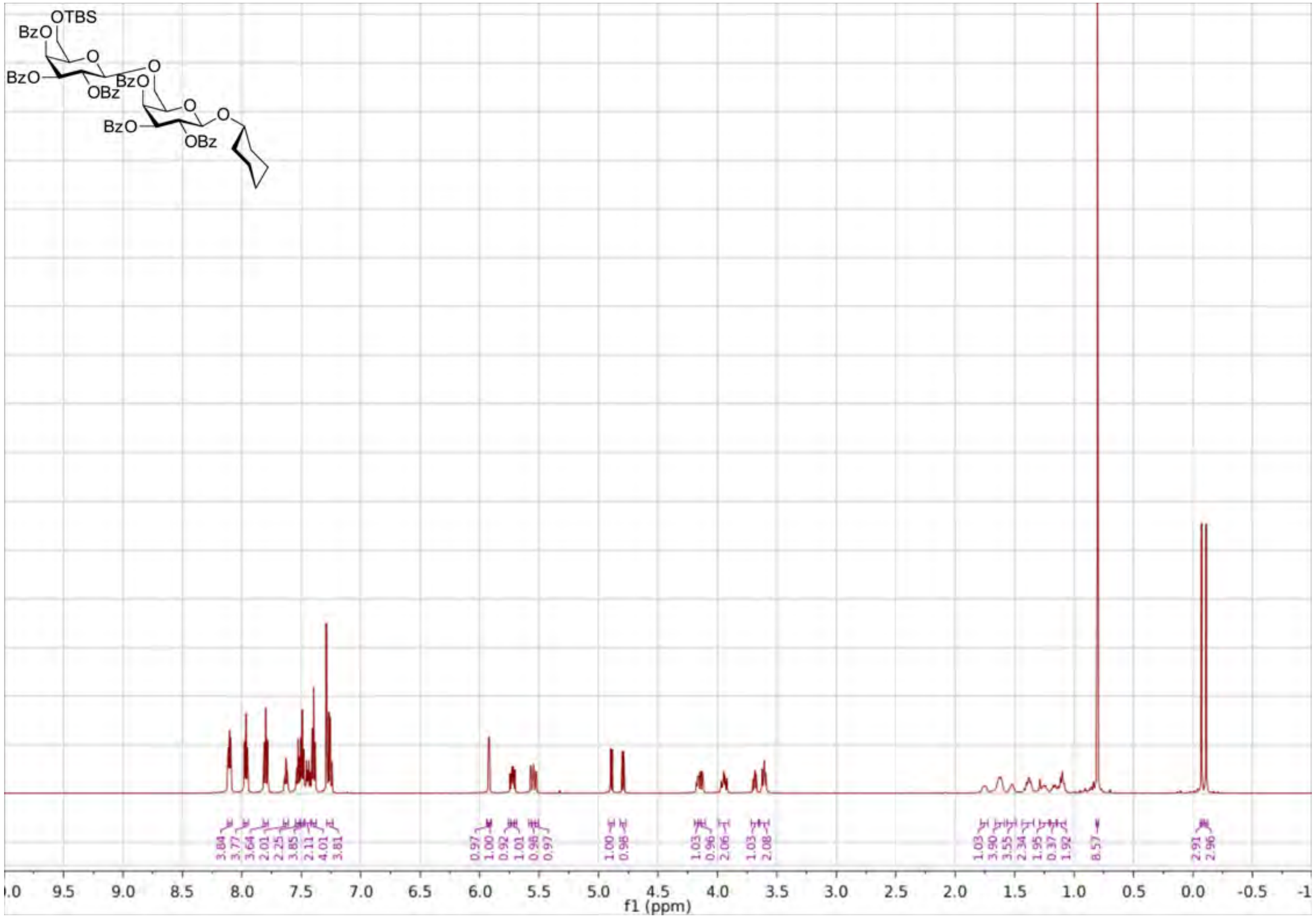

Carbon

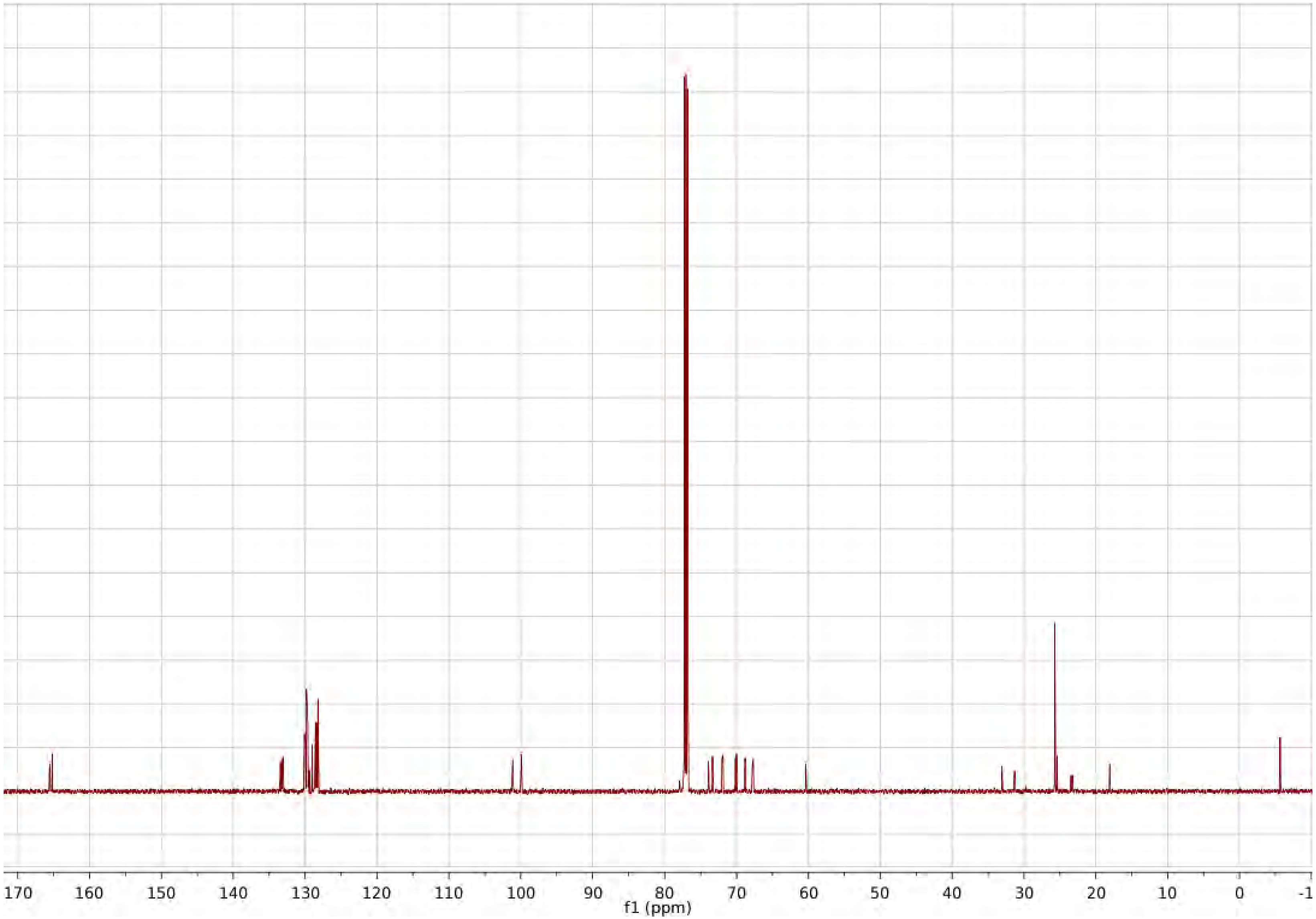

COSY

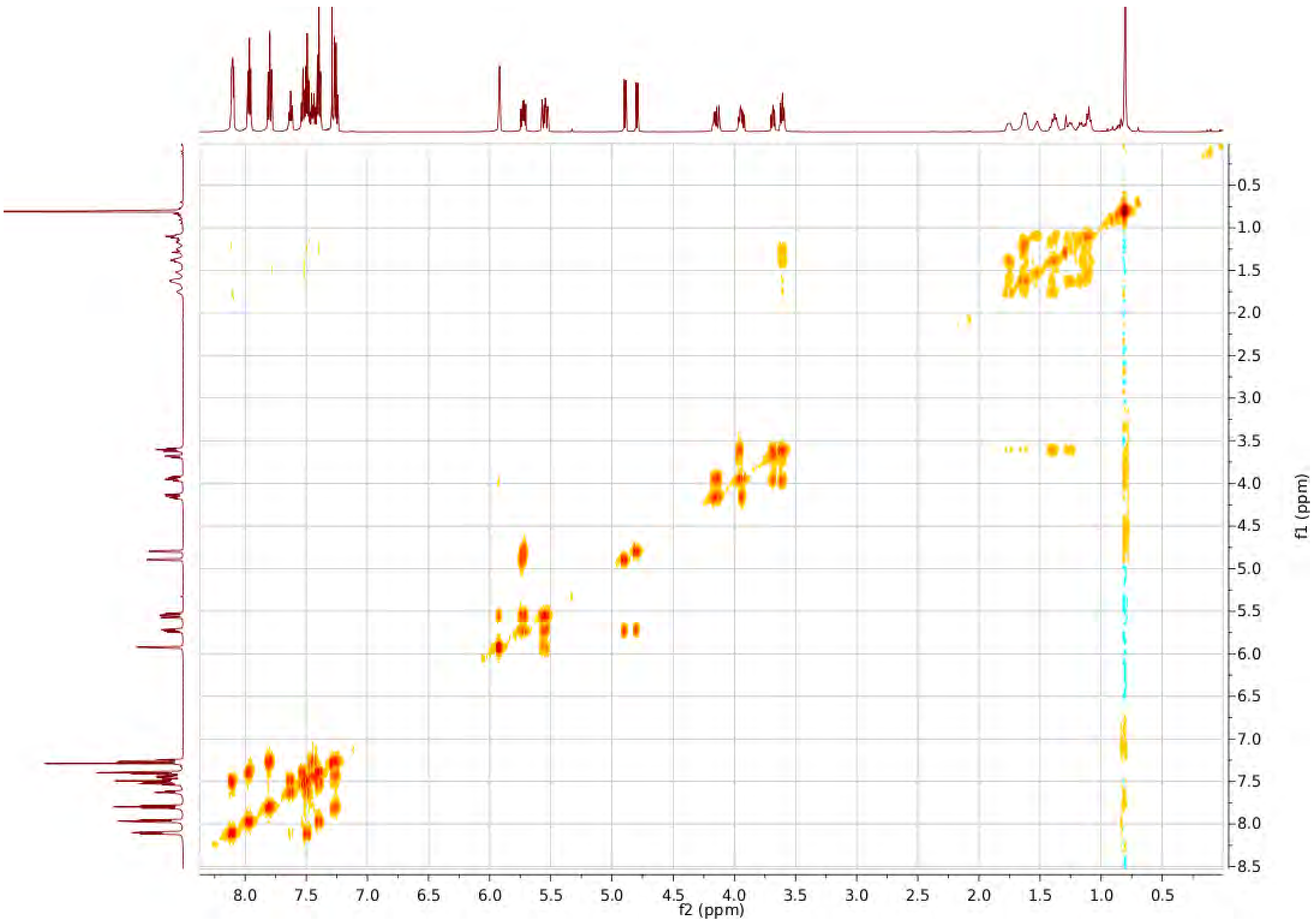

HSQC

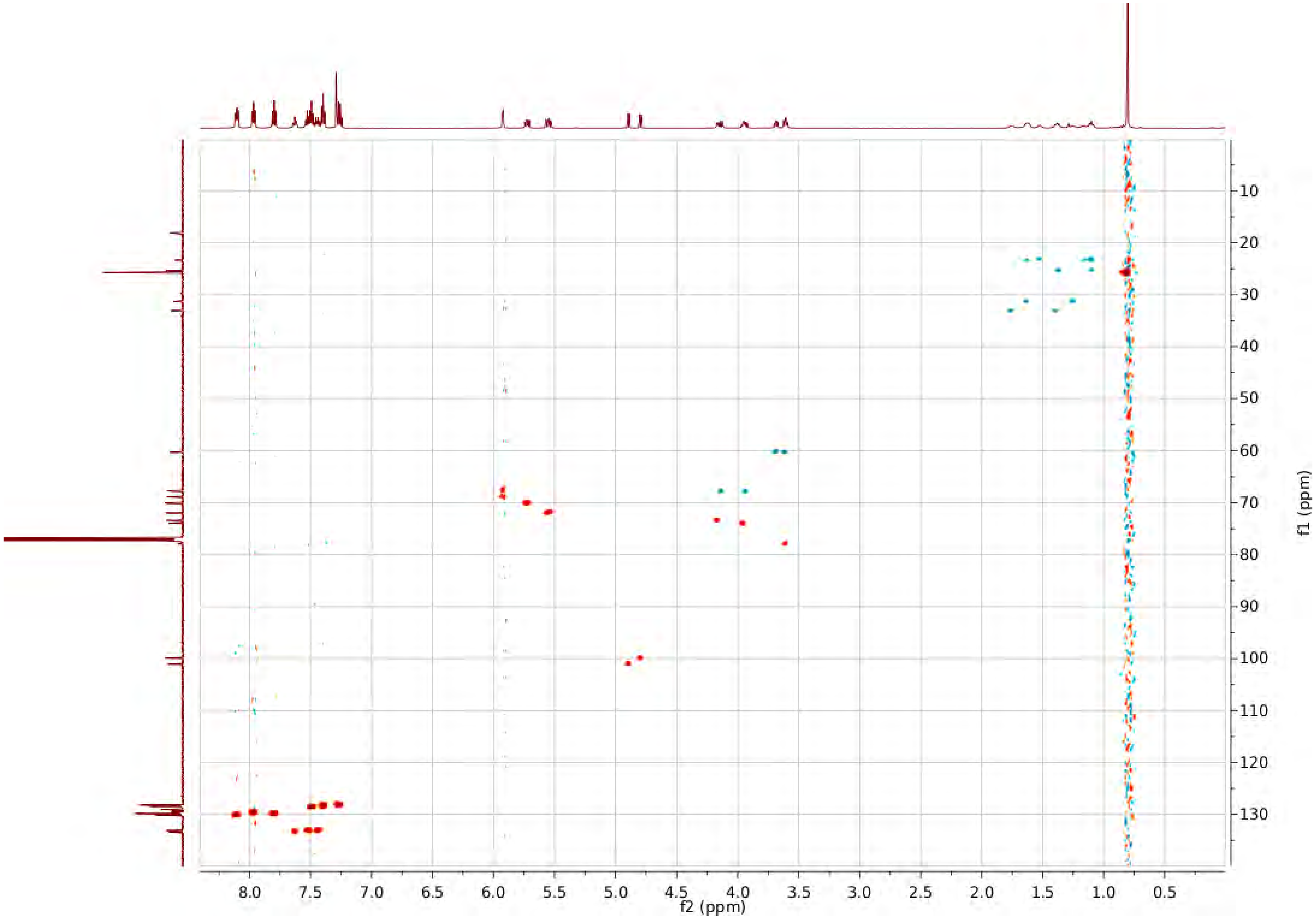

8.0
7.5
7.0
6.5
6.0
5.5
5.0
4.5
4.0
3.5
3.0
2.5
2.0
1.5
1.0
0.5
f2 (ppm)
10
20
30
40
50
60
70
80
90
100
110
120
130
f1 (ppm)

Compound **17$_{1}$b**

Proton

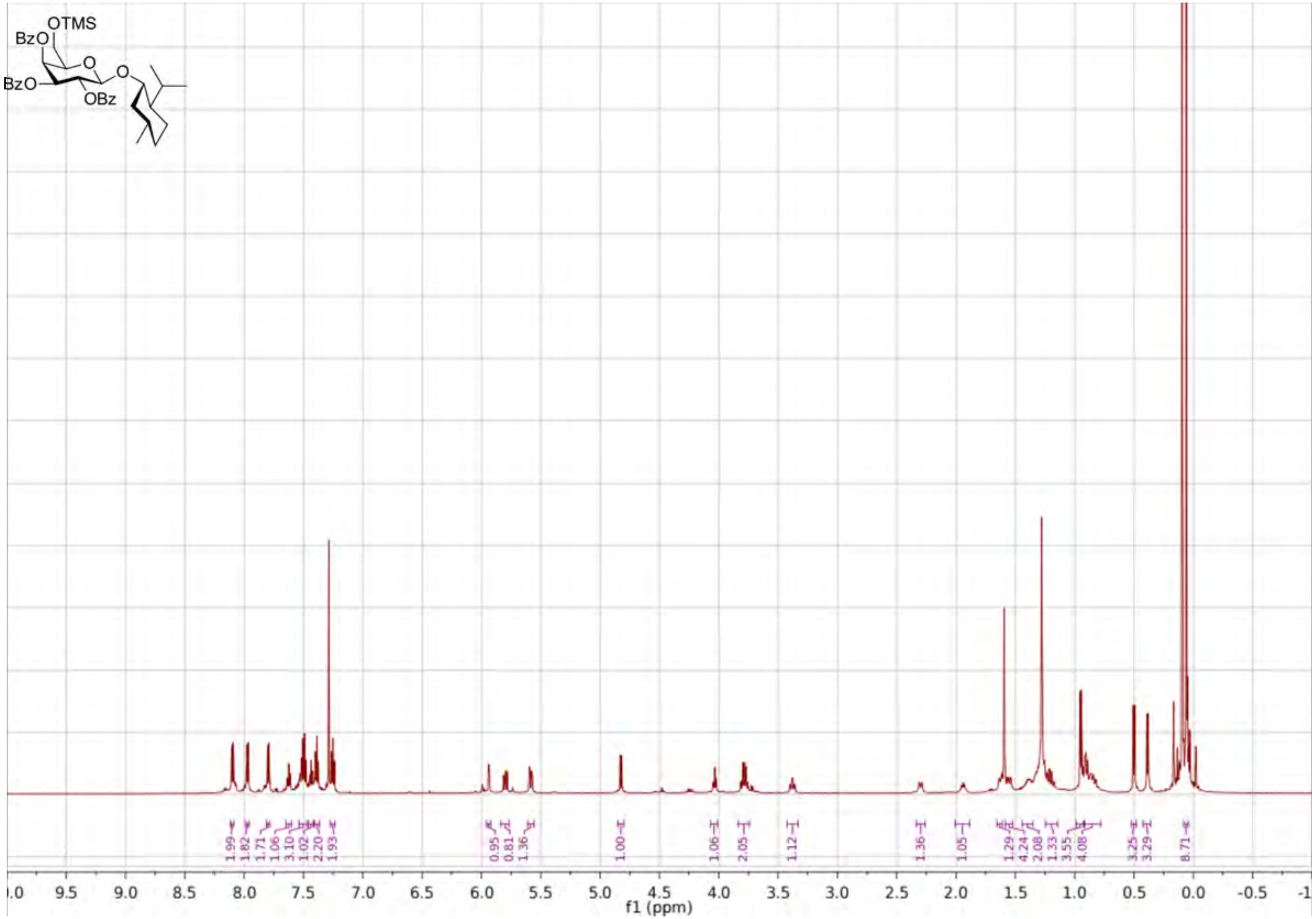

Carbon

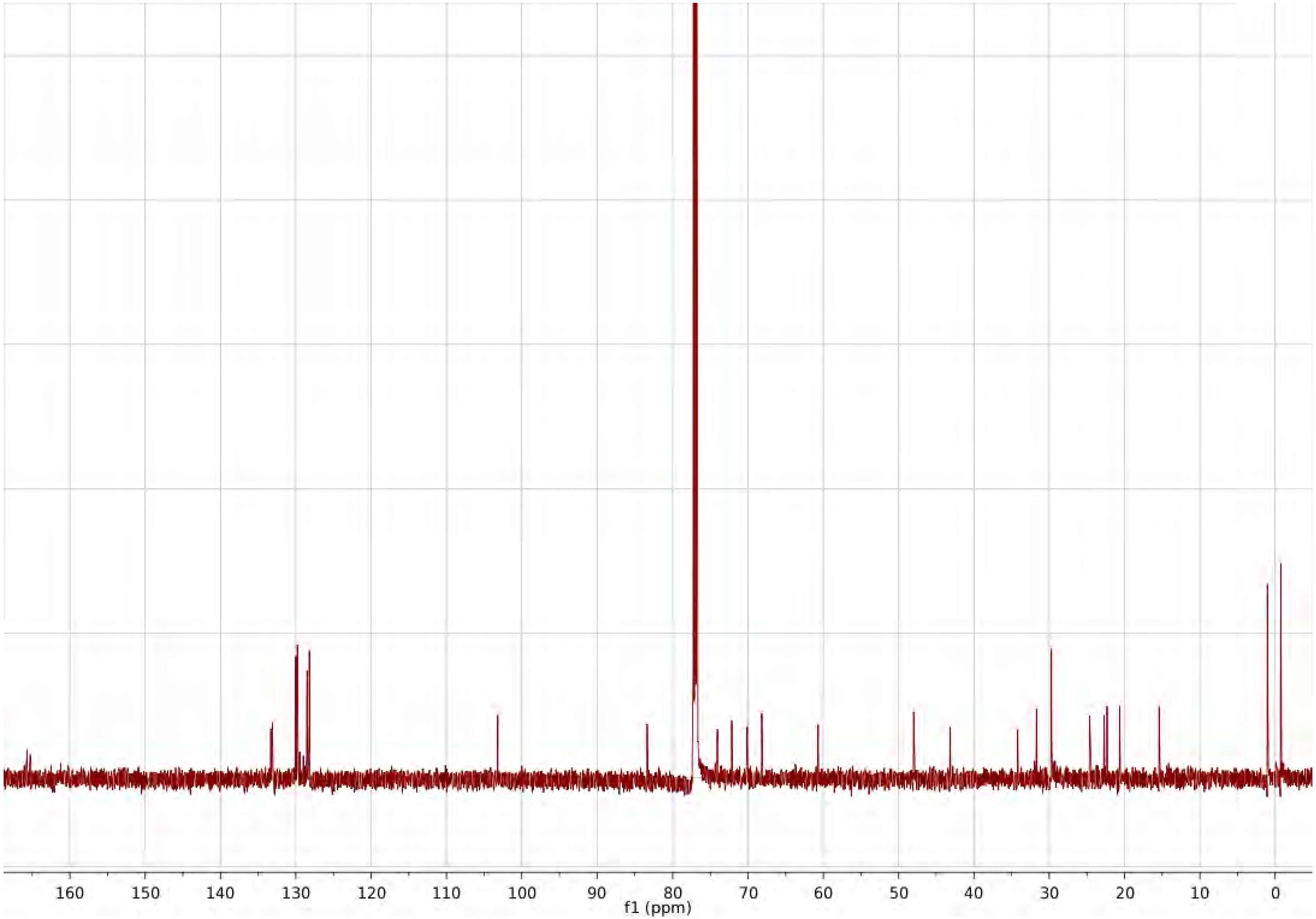

COSY

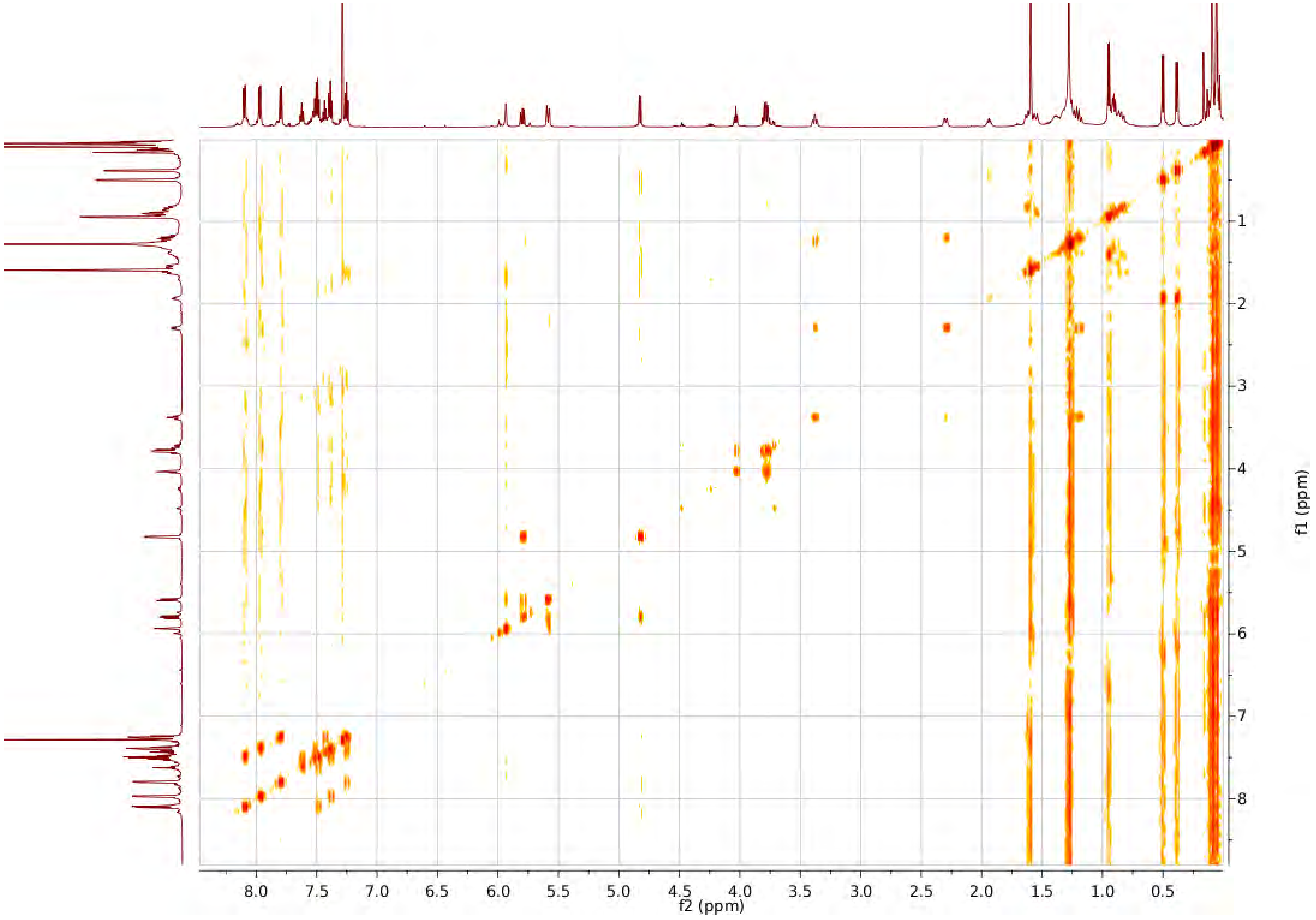

HSQC

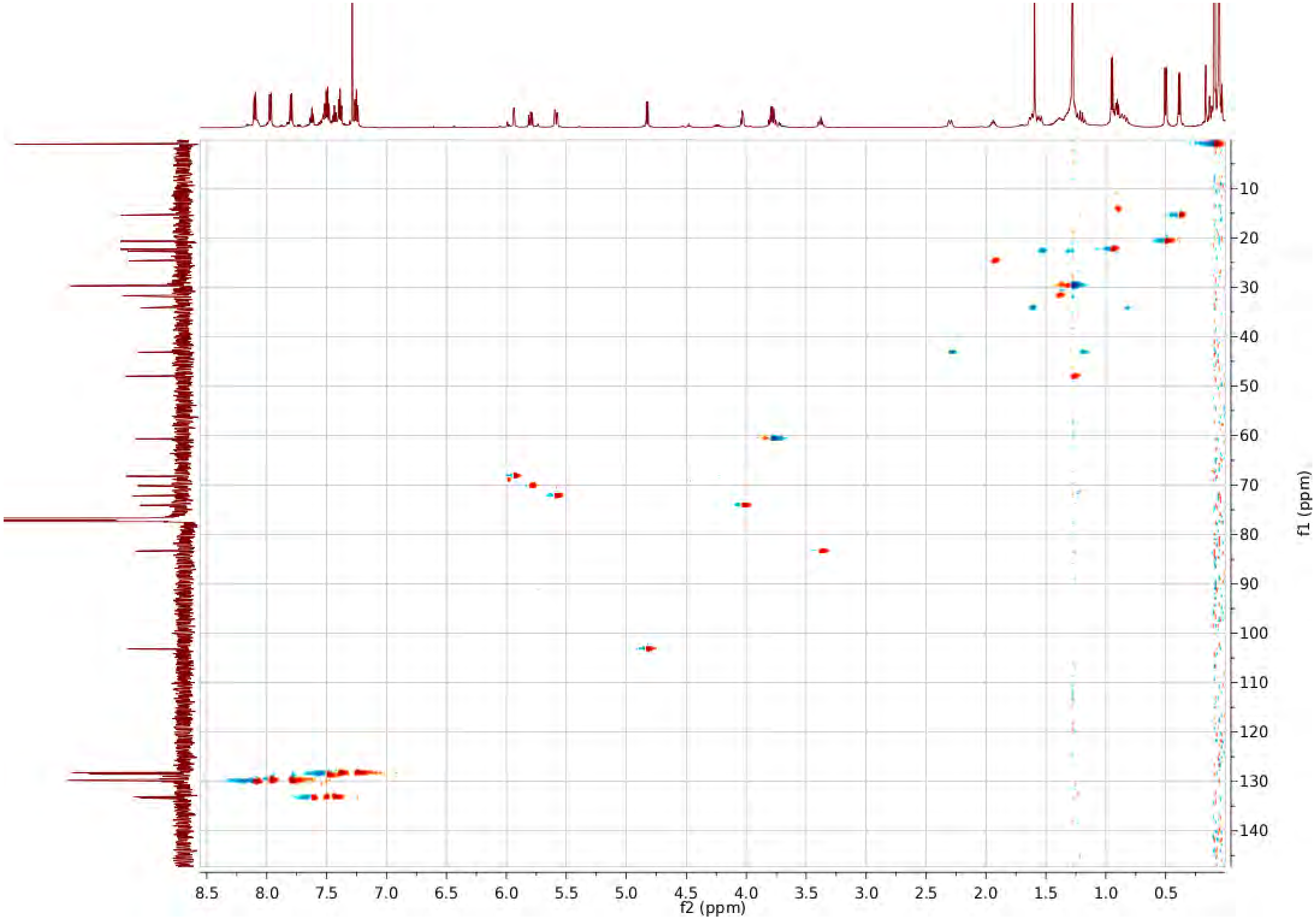

f2 (ppm)
f1 (ppm)
8.5
8.0
7.5
7.0
6.5
6.0
5.5
5.0
4.5
4.0
3.5
3.0
2.5
2.0
1.5
1.0
0.5
10
20
30
40
50
60
70
80
90
100
110
120
130
140

Compound **17_2b**

Proton

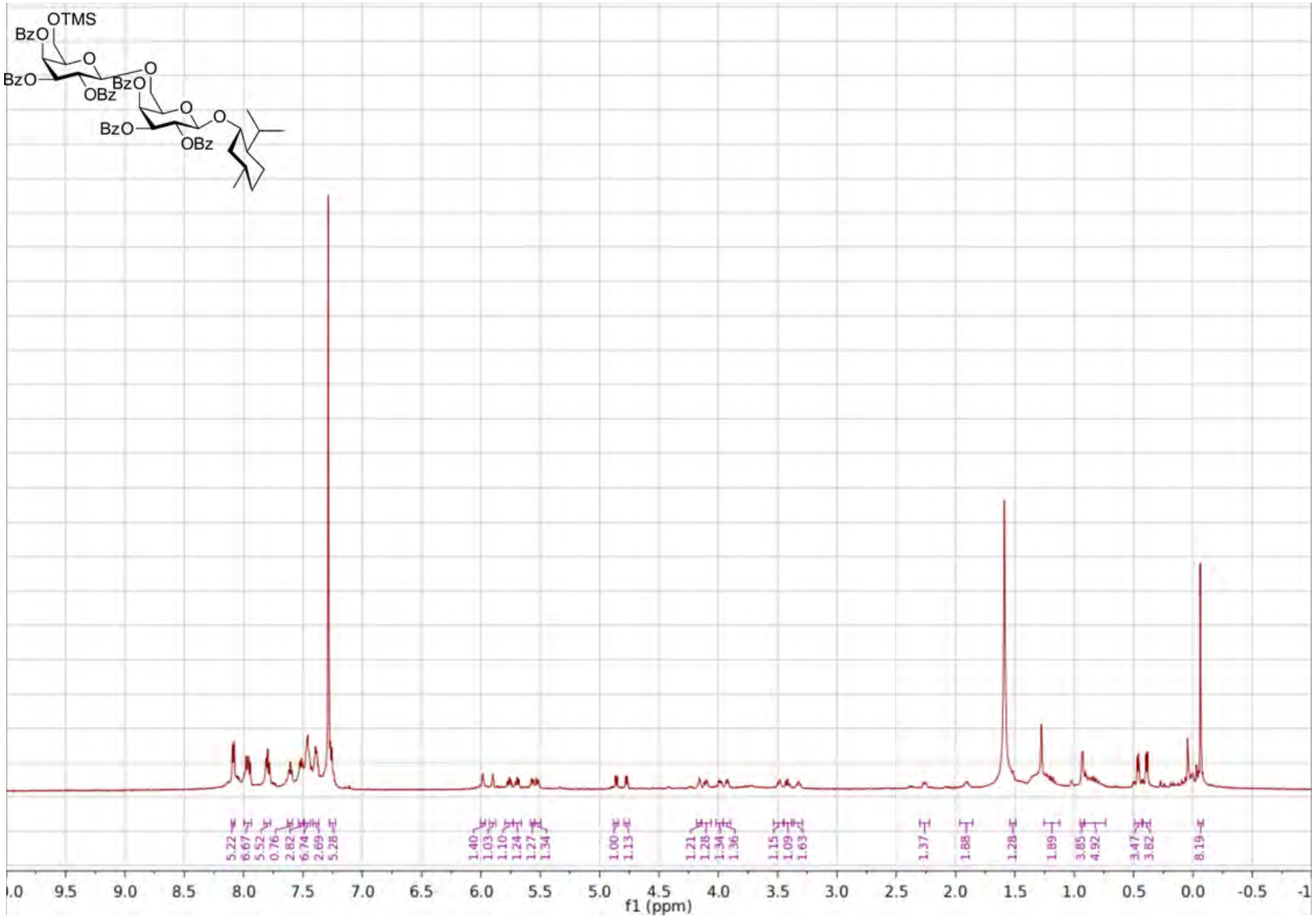

Carbon

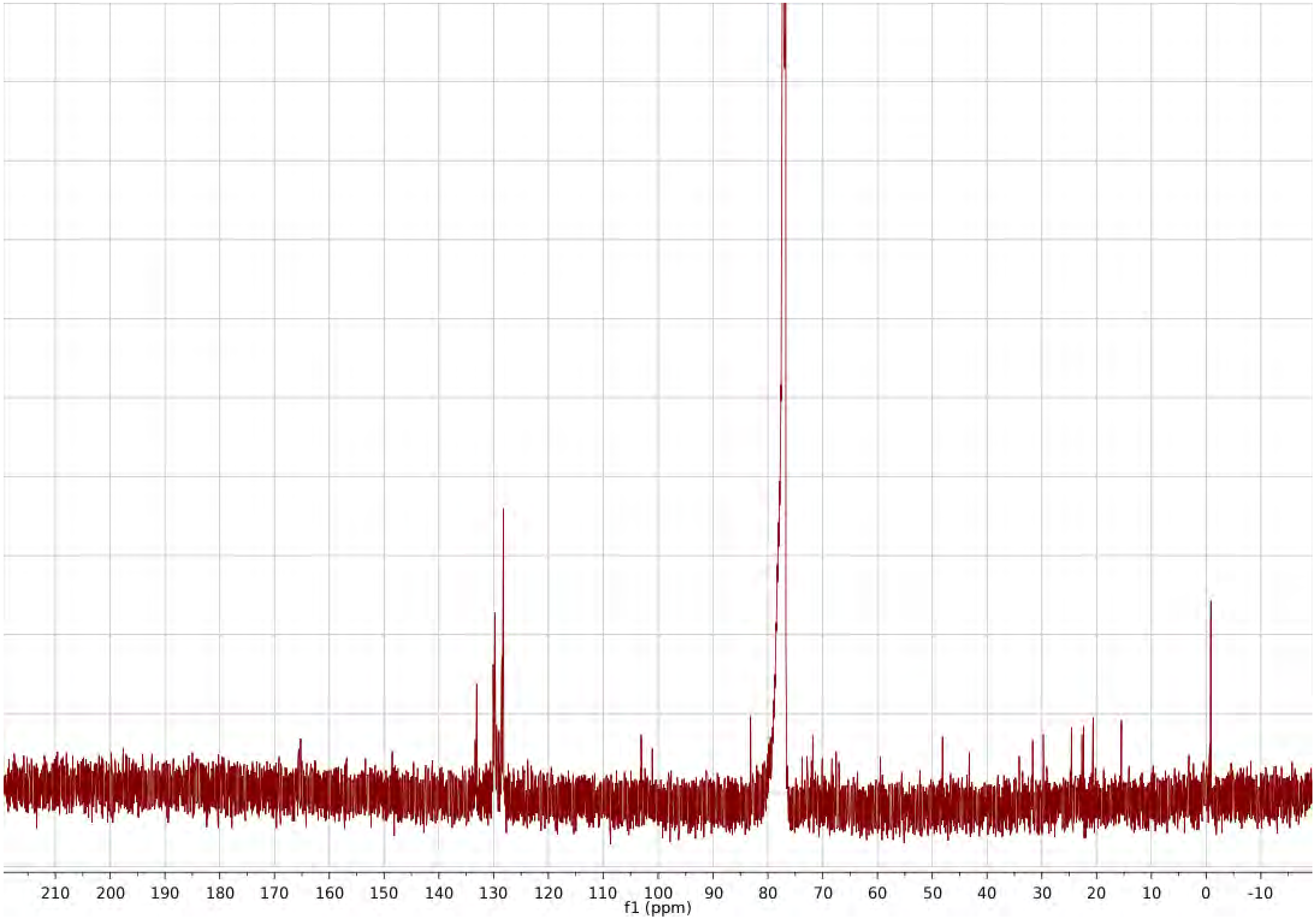

210
200
190
180
170
160
150
140
130
120
110
100
90
80
70
60
50
40
30
20
10
0
-10
f1 (ppm)

COSY

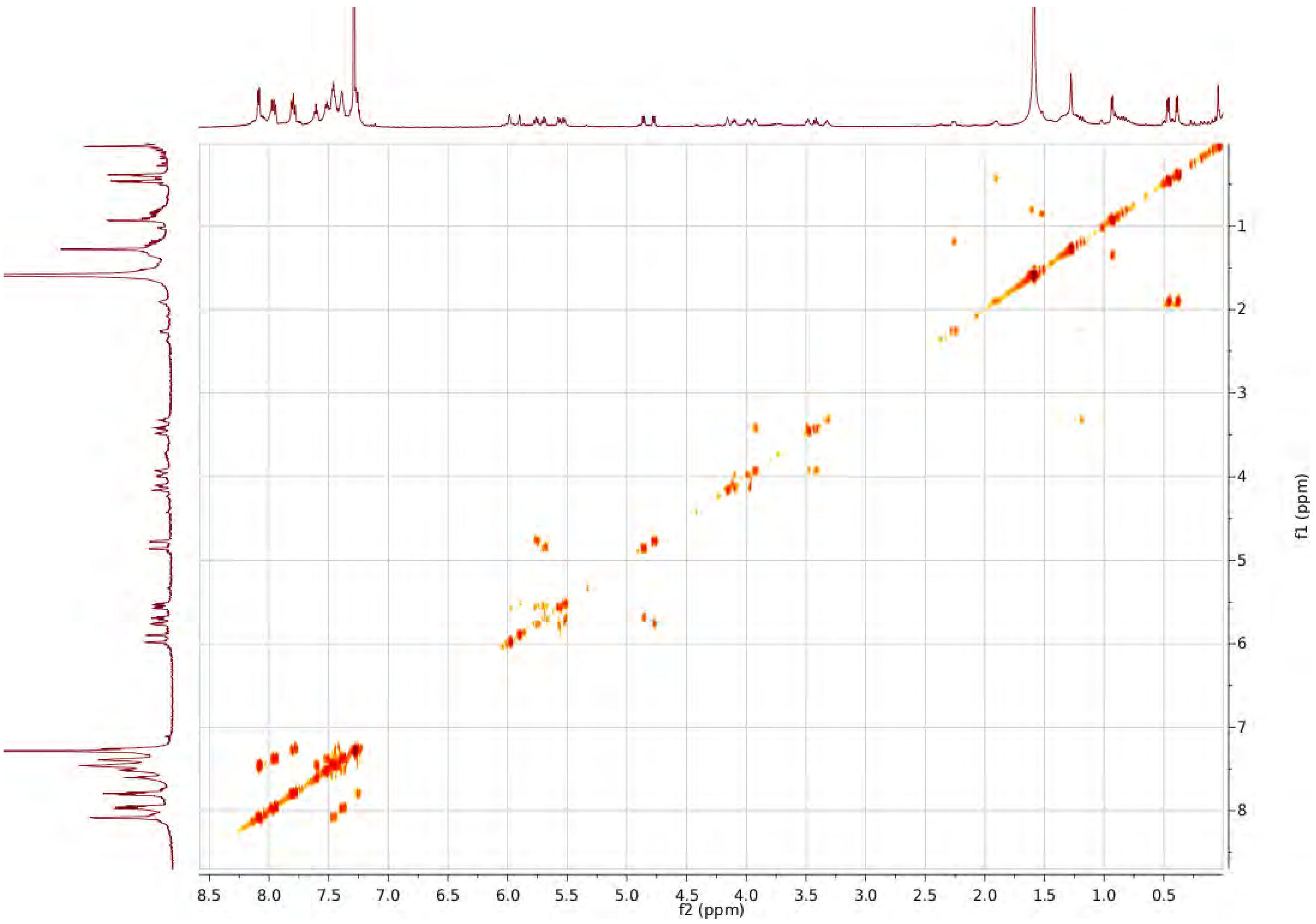

8.5
8.0
7.5
7.0
6.5
6.0
5.5
5.0
4.5
4.0
3.5
3.0
2.5
2.0
1.5
1.0
0.5
f2 (ppm)
1
2
3
4
5
6
7
8
f1 (ppm)

Compound **18₁b**

Proton

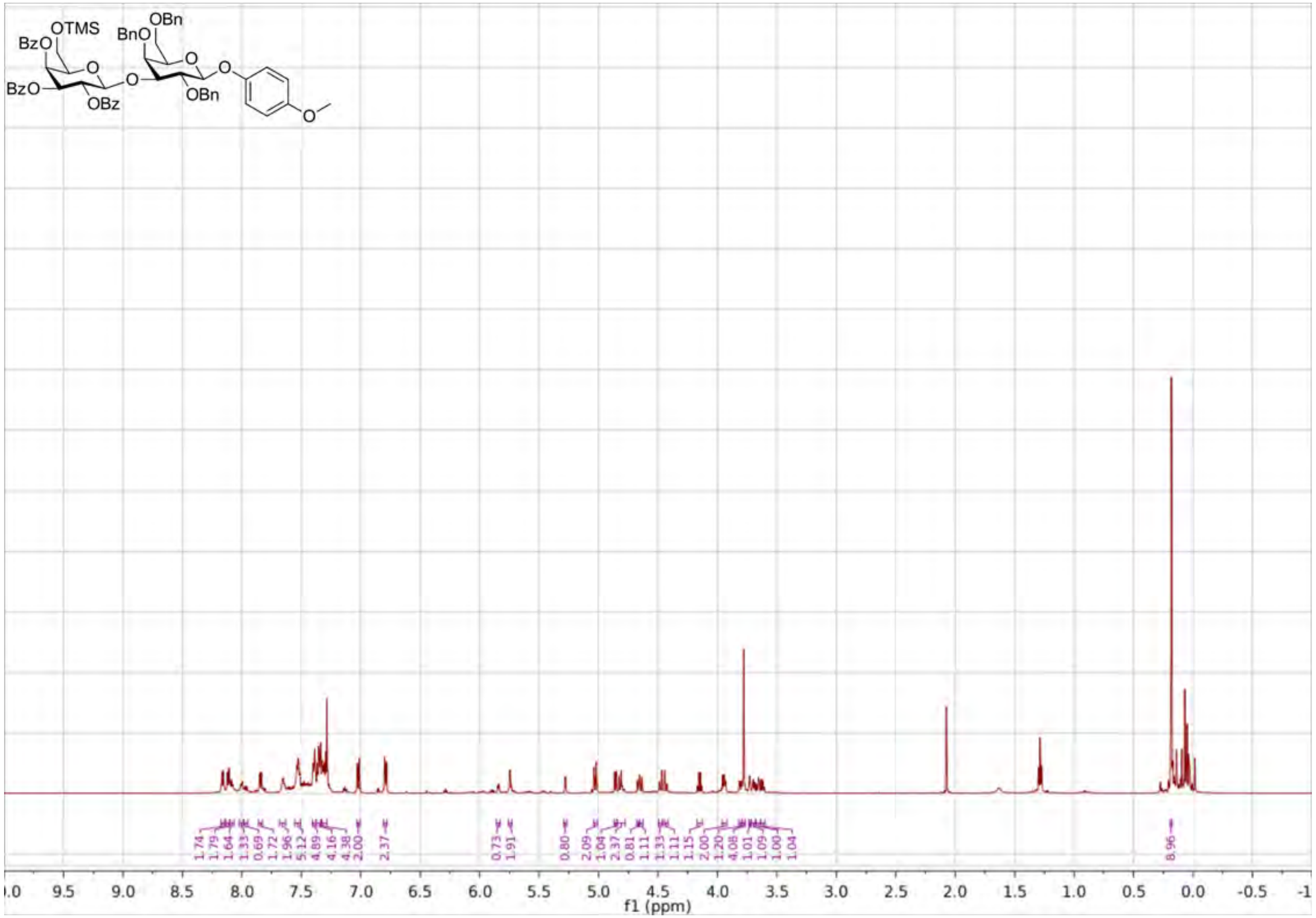

Carbon

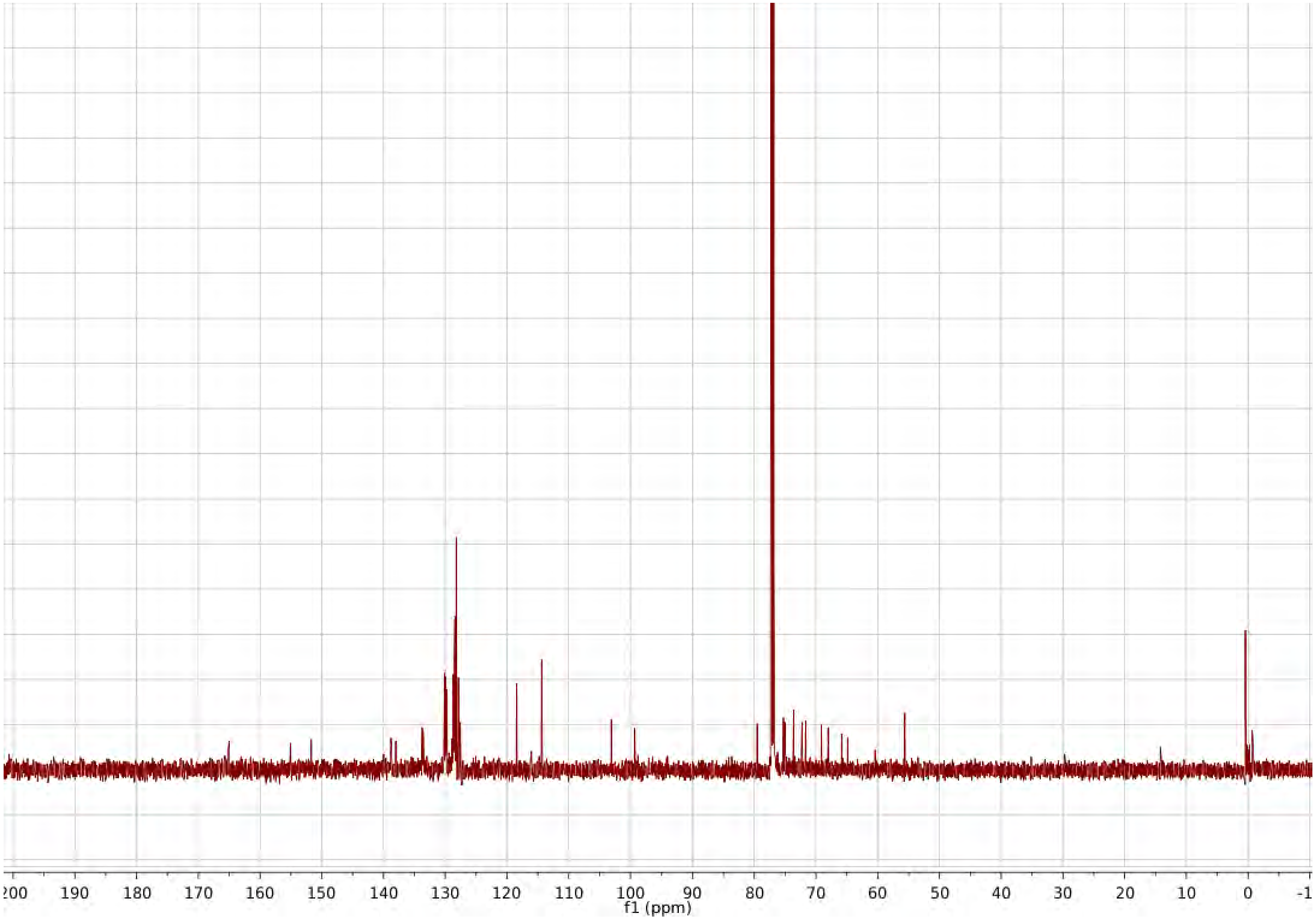

200 190 180 170 160 150 140 130 120 110 100 90 80 70 60 50 40 30 20 10 0 -1
f1 (ppm)

Compound **4$_5$b**

Proton

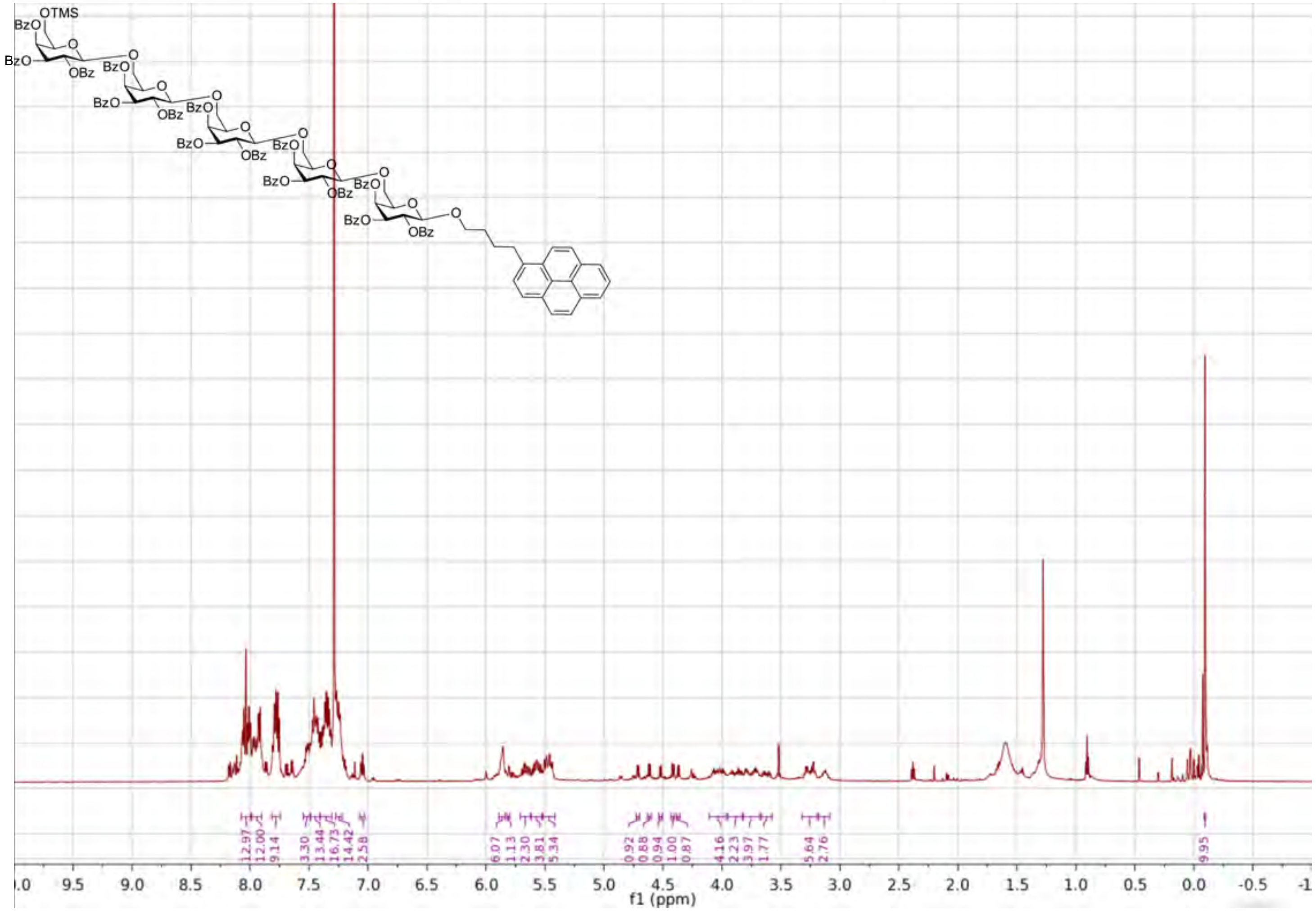

Supplement: File 2 — NMR spectra for all new compounds. [file Beilstein_J_Org_Chem-10-2658-s002.pdf]
